# PAnalyzer: A software tool for protein inference in shotgun proteomics

**DOI:** 10.1186/1471-2105-13-288

**Published:** 2012-11-05

**Authors:** Gorka Prieto, Kerman Aloria, Nerea Osinalde, Asier Fullaondo, Jesus M Arizmendi, Rune Matthiesen

**Affiliations:** 1 Department of Communications Engineering, University of the Basque Country (UPV/EHU), Alda. Urquijo s/n, Bilbao, 48013, Spain; 2 Proteomics Core Facility-SGIKER, University of the Basque Country (UPV/EHU), Barrio Sarriena s/n, Leioa, 48940, Spain; 3 Department of Biochemistry and Molecular Biology, University of the Basque Country (UPV/EHU), Barrio Sarriena s/n, Leioa, 48940, Spain; 4 Department of Genetics, Physical Anthropology and Animal Physiology, University of the Basque Country (UPV/EHU), Barrio Sarriena s/n, Leioa, 48940, Spain; 5 Proteolysis in diseases, IPATIMUP - Institute of Molecular Pathology and Immunology of the University of Porto, Rua Dr. Roberto Frias s/n, Porto, 4200-465, Portugal

## Abstract

**Background:**

Protein inference from peptide identifications in shotgun proteomics must deal with ambiguities that arise due to the presence of peptides shared between different proteins, which is common in higher eukaryotes. Recently data independent acquisition (DIA) approaches have emerged as an alternative to the traditional data dependent acquisition (DDA) in shotgun proteomics experiments. MS^*E *^is the term used to name one of the DIA approaches used in QTOF instruments. MS^*E *^data require specialized software to process acquired spectra and to perform peptide and protein identifications. However the software available at the moment does not group the identified proteins in a transparent way by taking into account peptide evidence categories. Furthermore the inspection, comparison and report of the obtained results require tedious manual intervention. Here we report a software tool to address these limitations for MS^*E *^data.

**Results:**

In this paper we present PAnalyzer, a software tool focused on the protein inference process of shotgun proteomics. Our approach considers all the identified proteins and groups them when necessary indicating their confidence using different evidence categories. PAnalyzer can read protein identification files in the XML output format of the ProteinLynx Global Server (PLGS) software provided by Waters Corporation for their MS^*E *^data, and also in the mzIdentML format recently standardized by HUPO-PSI. Multiple files can also be read simultaneously and are considered as technical replicates. Results are saved to CSV, HTML and mzIdentML (in the case of a single mzIdentML input file) files. An MS^*E *^analysis of a real sample is presented to compare the results of PAnalyzer and ProteinLynx Global Server.

**Conclusions:**

We present a software tool to deal with the ambiguities that arise in the protein inference process. Key contributions are support for MS^*E *^data analysis by ProteinLynx Global Server and technical replicates integration. PAnalyzer is an easy to use multiplatform and free software tool.

## Background

Shotgun proteomics is still the method most widely used for large-scale identification of proteins in complex biological samples [1,2]. This approach involves proteolysis of the proteins followed by chromatographic separation of the resulting peptides coupled to tandem mass spectrometry analysis. Then, a search engine assigns fragmentation spectra to peptide sequences by scoring the similarity between observed spectra and the predicted spectra of peptide sequences in a database. Finally, the assigned peptides are grouped to deduce the list of identified proteins in a process termed protein inference. This process is not trivial, especially in the case of higher eukaryote organisms, where a significant amount of peptides derives from several proteins due to the presence of homologous proteins, isoforms or redundant entries in the sequence database. These shared or degenerate peptides introduce ambiguity in determining the identities of sample proteins, complicating the analysis and biological interpretation of the data obtained [3].

The manner in which different protein inference software tools manage these ambiguities differ (reviewed in [4]). ProteinProphet [5], probably the most widely used software for peptide assembly, infers the most likely protein corresponding to degenerate peptides by apportioning such peptides among their corresponding proteins according to the estimated probabilities of those proteins in the sample, while in turn computing the protein probabilities taking into account those estimated apportionments. IsoformResolver [6] uses a “peptide-centric” approach where the observed peptides are directly referenced against a peptide database with the context of all possible proteins from which they can derive, which are then clustered in the same group. PeptideClassifier [7,8], besides MS data, considers data from other sources to facilitate peptide assembly. Each peptide is classified according to its information content with respect to protein sequences and gene models, and shared peptides are distinguished depending on whether the implied proteins could be encoded either by the same or by distinct gene models.

In the typical LC-MS/MS experiment, co-eluting peptides get into the mass spectrometer together and are selected for fragmentation on the basis of their abundance (data dependent acquisition or DDA). As an alternative, the mass spectrometer can be operated to fragment the entire range of co-eluting peptide ions without prior selection. Data-independent acquisition (DIA) based proteomics from complex mixtures of proteins has mainly been performed on Waters’ QTOF instruments where it has been termed MS^*E *^[9].

The commercial software tool ProteinLynx Global Server (PLGS) developed by Waters Corporation is the only one that deals with MS^*E *^data. PLGS renders a list of all proteins identified, including those identified only with shared peptides, and is also able to group proteins that share peptides into groups or hits. However, the principles used by this software to group the proteins and to select the protein that will name the group are not always based on peptide evidence. Therefore, the report of the results and the comparison of technical replicates are difficult, and besides manual inspection, PLGS does not provide a way to classify the identified proteins based on peptide evidence. Furthermore, none of the protein inference tools can read the output provided by PLGS.

Here we report PAnalyzer, a simple software tool to group and report the list of identified proteins into four simple categories following the recommendations proposed by Nesvizhskii & Aebersold [3]. PAnalyzer is the only software available that directly imports data files generated by PLGS after the database search using MS^*E*^ data and it can also import results generated by other tools in the standardized mzIdentML format [10]. The assigned peptides used to infer the proteins can be filtered according to their score. Moreover, multirun analysis is supported and a single list of identified proteins can be created from several replicate runs. Results can be exported as comma separated values files, as HTML reports and also as mzIdentML in the case of a single mzIdentML input file.

## Implementation

### Protein classification

PAnalyzer classifies proteins according to their evidence using three types of peptides: 

• Unique: peptides that can only be assigned to a single protein.

• Discriminating: shared peptides which presence is explained by a set of proteins without unique peptides.

• Non-discriminating: shared peptides which presence is explained by proteins with unique or discriminating peptides.

Using these three types of peptides, proteins matching a correct peptide identification can be classified into four evidence groups: 

• Conclusive: proteins with unique peptides.

• Non-conclusive: proteins containing only non-discriminating peptides.

• Indistinguishable: proteins that have discriminating peptides only shared by them and which also share the rest of the peptides.

• Ambiguous group: a group of proteins that explain the presence of a group of discriminating peptides.

**Figure 1:**
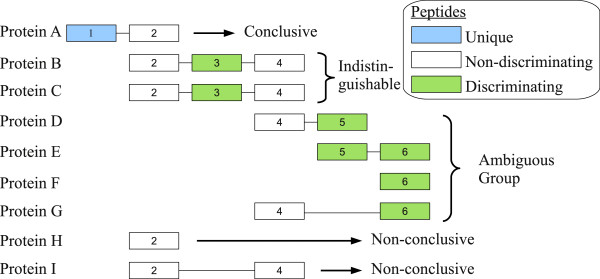
**Classification of proteins into four evidence groups.** 9 different proteins (Protein A to Protein I) comprising 6 different peptides (boxes 1 to 6) have been used to illustrate all possible scenarios. Conclusive proteins have at least one unique peptide; indistinguishable proteins have the same peptides including at least one discriminating peptide; ambiguous group is formed by proteins sharing discriminating peptides; non-conclusive proteins only have non-discriminating peptides.

A theoretical case where the three types of peptides and the four types of proteins are described is depicted in Figure 1.

### Classification algorithm

**Figure 2:**
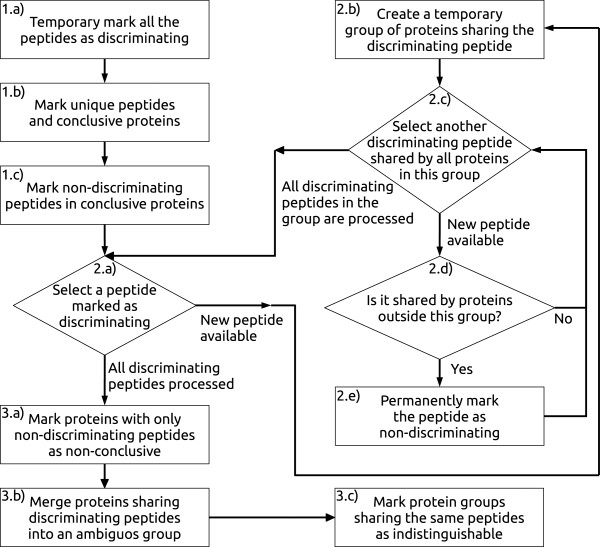
**Peptide classification algorithm.** Algorithm used by PAnalyzer to classify peptides before grouping the proteins into the different evidence groups.

Initially PAnalyzer loads all the protein and peptide identifications and builds a peptide list for each protein and a protein list for each peptide. Then the algorithm depicted in Figure 2 is followed. This algorithm can be separated in the following steps: 

1. An initial peptide classification is carried out to further refine it through iteration in the next step. Initially all the peptides are marked as discriminating. Then, peptides with just one entry in their protein list are marked as unique. Next, proteins with unique peptides in their peptide list are marked as conclusive. Finally, peptides not marked as unique and with conclusive proteins in their protein list and are marked as non-discriminating.

2. An iterative process is carried out to find the non-discriminating peptides temporary marked as discriminating in the previous step. The entries in the protein list of the first discriminating peptide are considered as a temporary group. Any other of the remaining discriminating peptides shared by all of the proteins in this group and also by other proteins outside the group are marked as non-discriminating. This process is repeated until it reaches the last peptide marked as discriminating.

3. Once all the peptides are classified, the protein classification and grouping process is completed. Conclusive proteins had already been classified in the first step. Proteins containing only non-discriminating peptides are marked as non-conclusive. Then proteins sharing discriminating peptides are merged into an ambiguous group. Finally groups with proteins sharing the same peptides are marked as indistinguishable.

### Integration of replicate runs

It is a common practice to perform different replicate runs of a sample in order to account for artifacts or to analyze it in a more exhaustive way. PAnalyzer accounts for this practice by providing the user the option to specify the minimum number of replicates (runs threshold) in which a peptide must be present to be considered in the protein inference step. This feature allows the users to be as flexible as they desire on the criteria adopted to accept the presence of a peptide in the sample.

Depending on the value selected by the user different approaches can be used. Two particular cases of interest are the following: 

• Peptide voting: runs threshold equals half plus one of the replicate runs. This is a conservative approach since only peptides present in at least half plus one of the replicate runs are considered for protein inference.

• Peptide merging: runs threshold equals one. This is a less conservative approach since all the identified peptides in any of the runs are considered as valid, so all the peptides are merged before protein inference.

**Figure 3:**
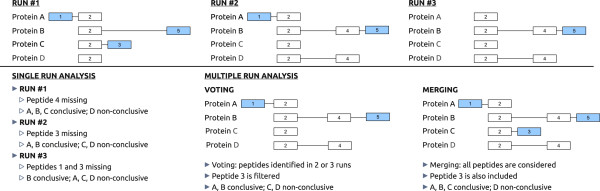
**Replicate run example.** 4 different proteins (A to D) comprising 5 peptides (1 to 5) have been used to illustrate the peptide voting and peptide merging approaches in a multiple run analysis example. The result of the individual samples (single run analysis) is also indicated.

Figure 3 shows an example of three replicate runs where different situations that affect the results of protein inference are considered. The same protein can be assigned to different evidence categories depending on which of its peptides are identified in a particular replicate. For example, protein A is conclusive in the first two runs and non-conclusive in the last run because peptide 1 is missing. The multirun approach accounts for this kind of fluctuations. Peptide 1 is present in two of the runs and protein A is considered as conclusive in both peptide voting and peptide merging approaches. Peptide 3 is only present in one of the runs so it is filtered in the voting approach and consequently protein C is considered as non-conclusive. On the contrary in the merging approach the same peptide is considered valid and makes protein C conclusive. As it is evident from this example the merging approach will result into more conclusive proteins whereas the voting approach will lead to more non-conclusive proteins and ambiguity groups.

### Software technology

**Figure 4:**
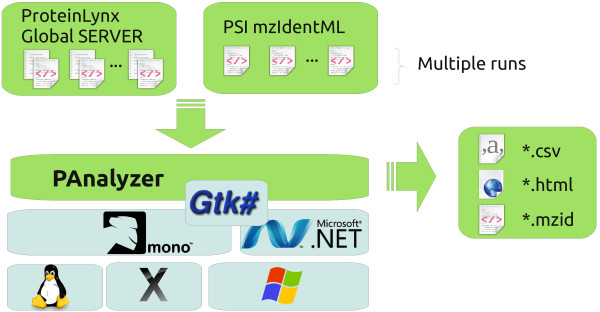
**Block diagram for running PAnalyzer.** PAnalyzer can read one or more ProteinLynx Global Server output files and mzIdentML files and exports the reorganized protein list to CSV, HTML and mzIdentML. The tool runs in every platform where a .NET version 4 compliant CLR is available.

Figure 4 depicts the different software technologies used by PAnalyzer.

Multiplatform support is provided by .NET using the Microsoft implementation for Windows or the Mono free software implementation. This last one has been ported to different platforms, such as GNU/Linux, MacOS X and also Windows. GUI portability is assured by using the free software GTK# toolkit.

Input files can be in the output format of ProteinLynx Global Server, which is the software provided by Waters Corporation for their MS^*E *^data, or in the mzIdentML format recently standardized by the HUPO-PSI [10]. After performing the protein classification, results can be saved into comma-separated values (CSV), mzIdentML and HTML files as shown in the results section.

## Results and discussion

In order to validate the proposed methodology and software implementation a shotgun proteomics experiment has been carried out using protein extracts of Human Embryonic Kidney 293T (HEK 293T) cells. The results obtained by PAnalyzer are compared with those provided by ProteinLynx Global Server.

### Software tool

PAnalyzer is an easy to use tool to handle with the protein inference problem from peptide identifications. Input file formats supported are the output XML files of protein identifications generated by ProteinLynx Global Server (PLGS) versions 2.4 and 2.5, and the new HUPO-PSI standardized format mzIdentML version 1.0.0 and version 1.1.0. In the case of using PLGS files as input, peptide threshold can be specified as “green”, “yellow” or “red” and the associated numerical values are read from the log files generated by PLGS. In the case of mzIdentML files, peptides are filtered according to the “passThreshold” attribute of their corresponding “SpectrumIdentificationItem”. In any case, multiple input files can be selected and are interpreted as different replicate runs, allowing the user to choose the value of the replicate runs threshold.

A simple peptide and protein browser is provided in order to have a quick look at the resulting peptides and inferred proteins before exporting them to an output file. The results can be saved to CSV, HTML and mzIdentML.

CSV output is provided as a convenient format for researches that only want to see the basic data in a spreadsheet or for those who want to perform basic scripting. An example is provided in Additional file 1.

**Figure 5:**
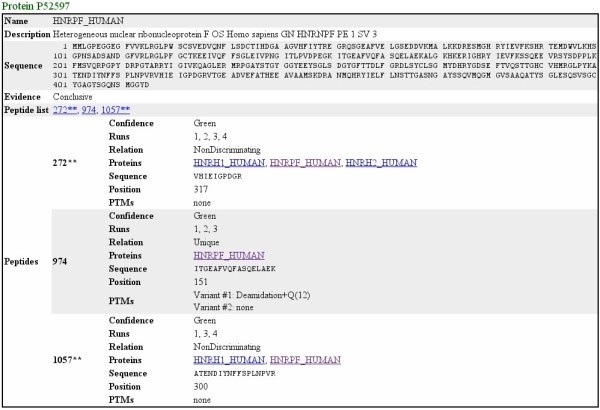
**HTML output.** Example of the protein details section of the HTML output. This conclusive protein has one unique peptide and two non-discriminating peptides. The unique peptide has been detected in 3 out of the 5 replicates (run numbers 2, 3 and 4). In peptides number 459 and 1567 no PTMs have been detected while in peptide number 2621 two peptide variants (different PTMs) have been identified. A number is used for unique peptides, a “*” is appended to discriminating peptides, and a “**” to non-discriminating peptides.

HTML output displays the information in a more convenient way and uses hyperlinks to browse within the different sections of the file. The first section of the report presents the analysis configuration and stats on the number of proteins within the different evidence categories. A second section provides a summary list of identified proteins and groups of proteins with their peptides and evidence type. Clicking in any of the proteins redirects to the protein details section where all the protein, peptide and PTM details are displayed. An example is provided in Additional file 2 and a screenshot of the protein details section in Figure 5.

mzIdentML output is only generated if the input data is also in this format and it is a single run analysis. The inferred proteins replace the “ProteinDectectionList” with new “ProteinAmbiguityGroup” entries. These groups can be composed of one or more inferred proteins according to their shared peptides. But this scheme is not enough to account for the protein evidence categories previously presented. PAnalyzer solves this by using the possibility that mzIdentML has to include additional information from a controlled vocabulary. A new entry (accession number MS:1001600) named “Protein Inference Confidence Category” has been specifically included in the PSI mass spectrometry ontology (PSI-MS, version 2.40) and PAnalyzer makes use of it in every “ProteinDetectionHypothesis” within the “ProteinAmbiguityGroup” to specify whether it consists on a conclusive protein, non-conclusive protein, ambiguous group or indistinguishable group. An example is provided in Additional file 3.

### Sample analysis

#### Sample preparation

Proteins were extracted from Human Embryonic Kidney 293T (HEK 293T) cell line by incubating in lysis buffer (10 mM TrisHCl pH 7.4, 1% Triton X-100, 0.5% NP-40, 150 mM NaCl, 1 mM EDTA, 1 mM EGTA, 0.2 mM ortovanadate).Samples were centrifuged at 13000 rpm for 15 min, supernatant was recovered and protein content was measured by Bradford assay (Sigma Aldrich, St. Louis, MO, USA). Protein extracts (50 *μ*g) were precipitated with 2-D Clean-Up kit (GE Healthcare) resuspended in 0.1% RapiGest SF (Waters, Milford, MA, USA) and digested with trypsin (Roche Diagnostics, Penzberg, Germany) following standard digestion protocol. LC-MS^*E*^ analysis was performed with a nanoAcquity UPLC System interfaced to a SYNAPT HDMS mass spectrometer (Waters). 1.5 *μ*g of protein was loaded onto a Symmetry 300 C18, 180 *μ*m x 20 mm precolumn (Waters). The precolumn was connected to a BEH130 C18, 75 *μ*m x 200 mm, 1.7 *μ*m (Waters) column and peptides were directly eluted onto a homemade emitter.

Mass spectra were acquired using data independent acquisition mode (MS^*E*^) described by Geromanos et al. [11] with minor modifications. Spectra were processed with ProteinLynx Global Server 2.4 (Waters). Protein identification was obtained with the embedded database search algorithm of the program [12] and a human UniProtKB/Swiss-Prot database (version 2012_02) was used. For protein identification the following parameters were adopted: carbamidomethylation of C as fixed modification; N-terminal acetylation, N and Q deamidation and M oxidation, as variable modifications; 1 missed cleavage and automatic precursor and fragment error tolerance. A maximum protein false discovery rate of 5% was allowed.

#### Sample results

Protein extracts from 5 biological replicates of HEK 293T cultures were prepared and analyzed as described in the sample preparation section, and protein identification was performed with ProteinLynx Global Server (Table 1).

**Table 1:** Comparison of results reported by PLGS and PAnalyzer

**Single Run**		**#1**	**#2**	**#3**	**#4**	**#5**
**PLGS**	Hit	143	161	144	129	134
	Protein	305	338	324	293	307
	Conclusive	173	178	179	161	157
	Indistinguishable	77 (29)	94 (39)	82 (32)	63 (26)	78 (31)
**PAnalyzer**	Ambiguous group	0	4 (1)	12 (3)	0	18 (2)
	Non-conclusive	55	62	51	69	54
	Filtered	0	0	0	0	0
**Multiple Run**		Th1	Th2	Th3	Th4	Th5
**PLGS**	Hit	-	-	-	-	-
	Protein	-	-	-	-	-
	Conclusive	335	161	100	69	54
	Indistinguishable	74 (37)	103 (38)	106 (33)	104 (31)	84 (28)
**PAnalyzer**	Ambiguous group	11 (3)	9 (2)	12 (2)	11 (2)	24 (2)
	Non-conclusive	98	81	82	66	65
	Filtered	0	164	218	268	291

Total protein extracts from HEK 293T cells have been analyzed in 5 replicate runs. In single run analysis, the number at the top row indicates the run identifier, while in multiple run analysis the number indicates the runs threshold. ProteinLynx Global Server rows show the number of hits and proteins reported, while the PAnalyzer rows show the number of proteins reported in the different evidence categories presented in the paper. The number of groups are indicated in parenthesis in the case of indistinguishable and ambiguous proteins. ProteinLynx Global Server (PLGS) cannot perform multiple run analysis. The peptide score filter is low confidence (red).

PLGS groups the identified proteins in hits. A hit is a group of proteins (or a single protein) that share peptides and one of the members has at least one unique and high confidence peptide. Furthermore, the top scoring protein of the group names the hit. This simple grouping cannot address the different scenarios that occur in large-scale identification experiments. For example when a protein shares peptides with more than one protein that have unique and high scoring peptides or when several proteins share all the peptides and have the same score. Moreover, manual inspection of each hit is the only way to know exactly the proteins that belong to a specific group.

As previously described, PAnalyzer groups the identified proteins following the recommendations proposed by Nesvizhskii & Aebersold [3]. The results obtained for the 5 biological replicates of HEK 293T samples by PLGS and PAnalyzer are shown in Table 1. Approximately 55% of the identified proteins are conclusive, meaning that they have at least 1 unique peptide and are definitively present in the sample. Nearly 20% of the proteins are non-conclusive and their presence in the sample is not supported by peptide evidence. The rest of the identified proteins belong to groups of proteins, more than 90% to indistinguishable groups with an average of 2.5 proteins per group.

As the peptide filtering criteria is increased to medium and high confidence peptides the percentage of conclusive protein decreases (see tables in Additional files 4 and 5).

PAnalyzer can also process simultaneously results from multiple replicate analysis. Protein classification can be performed based on peptide score criteria or/and replicate criteria (Table 1 and tables in Additional files 4 and 5). In the results shown in Table 1 no peptide filtering has been applied and only replicate criteria (presence in a minimum number of replicates) has been considered. Increase in the replicate criteria or replicate threshold entails a clear decrease in the number of conclusive proteins but it does not produce such a big effect in the number of indistinguishable, ambiguous and non-conclusive proteins.

All these results can be easily visualized and stored in CSV, HTML and mzIdentML (when mzIdentML files are processed) formats for further data analysis (Additional files 1, 2 and 3).

## Conclusions

A software tool for protein inference has been developed according to the protein classification of Nesvizhskii & Aebersold [3]. This tool defines three kinds of peptides (unique, discriminating and non-discriminating) to classify the proteins into four evidence groups: conclusive, non-conclusive, indistinguishable and ambiguous group. It is important to make this protein classification since many studies use proteins that cannot be guaranteed to be present on a sample or, on the contrary, do not consider proteins that could be present in the sample because the protein inference tool based on the Occam’s razor simplification discards them. Conclusive proteins can be considered to be present in the sample, indistinguishable proteins and ambiguous groups usually relate to protein isoforms and it cannot be assured which protein combination is present. Finally non-conclusive proteins are filtered by different software tools according to the Occam’s razor principle, since the observed peptides can already be explained by the presence of the rest of the proteins. But non-conclusive proteins can be present in the sample and instead of being discarded should be tagged as non-conclusive for further analysis.

Multiple replicate run support has also been implemented using a threshold to define the minimum number of runs in which a peptide must be present in order to be considered in the protein inference process.

PAnalyzer is a simple and easy to use software tool to group and report proteins identified in a shotgun proteomics experiment, specially useful when data are obtained by MS^*E *^acquisition mode.

## Availability and requirements

**Project name:** PAnalyzer

**Project home page:**http://code.google.com/p/ehu-bio/wiki/PAnalyzer

**Operating systems:** Platform independent

**Programming language:** C#

**Other requirements:** .NET 4 or higher, GTK# 2.12 or higher

**License:** GNU GPL

**Any restrictions to use by non-academics:** None

## Competing interests

The authors declare that they have not competing interests.

## Authors’ contributions

JMA, KA and AF conceived of the study, and participated in its design and coordination and helped to draft the manuscript. GP designed the algorithms, wrote the software code and the final version of the manuscript. RM reviewed the algorithms using his own implementation in VEMS software and commented the manuscript. NO prepared the cell samples and reviewed the manuscript. KA also tested the software and validated the results. All authors read and approved the final manuscript.

## Supplementary Material

Additional file 1 **Sample CSV output file.** The output report in CSV format for the HEK 293T sample analysis using as input five replicate runs. Peptide score threshold has been selected as red (low confidence) and runs threshold as 3 (identification in at least 3 replicates). Click here for file

Additional file 2 **Sample HTML output file.** The output report in HTML format for the HEK 293T sample analysis using as input five replicate runs. Peptide score threshold has been selected as red (low confidence) and runs threshold as 3 (identification in at least 3 replicates). Click here for file

Additional file 3 **Sample mzIdentML output file.** The output report in mzIdentML v1.1.0 format for the Mascot MS/MS analysis example provided by the HUPO-PSI group. Click here for file

Additional file 4 **Results reported by PLGS and PAnalyzer filtering low confidence peptides.** Total protein extracts from HEK 293T cells have been analyzed in 5 replicate runs. In single run analysis, the number at the top row indicates the run identifier, while in multiple run analysis the number indicates the runs threshold. ProteinLynx Global Server rows show the number of hits and proteins reported, while the PAnalyzer rows show the number of proteins reported in the different evidence categories presented in the paper. The number of groups are indicated in parenthesis in the case of indistinguishable and ambiguous proteins. ProteinLynx Global Server (PLGS) cannot perform multiple run analysis. The peptide score filter is medium confidence (yellow). Click here for file

Additional file 5 **Additional file 5: Results reported by PLGS and PAnalyzer with only high confidence peptides.** Total protein extracts from HEK 293T cells have been analyzed in 5 replicate runs. In single run analysis, the number at the top row indicates the run identifier, while in multiple run analysis the number indicates the runs threshold. ProteinLynx Global Server rows show the number of hits and proteins reported, while the PAnalyzer rows show the number of proteins reported in the different evidence categories presented in the paper. The number of groups are indicated in parenthesis in the case of indistinguishable and ambiguous proteins. ProteinLynx Global Server (PLGS) cannot perform multiple run analysis. The peptide score filter is high confidence (green). Click here for file
