# Supplementary material for: PAnalyzer: A software tool for protein inference in shotgun proteomics
Source: BMC Bioinformatics. 2012 Nov 5;13:288. doi: 10.1186/1471-2105-13-288 (PMC3548767; doi:10.1186/1471-2105-13-288)
Supplement: Additional file 2 — Sample HTML output file. The output report in HTML format for the HEK 293T sample analysis using as input five replicate runs. Peptide score threshold has been selected as red (low confidence) and runs threshold as 3 (identification in at least 3 replicates). [file 1471-2105-13-288-S2.html]

PAnalyzer: Protein identification report for AdditionalFile


## PAnalyzer: Protein identification report for AdditionalFile

---

Content:

1. Analysis Configuration- Protein Summary- Protein List- Protein Details

  

Analysis Configuration

| Software | PAnalyzer (v0.13) |
| Analysis type | Multirun analysis |
| Number of runs | 4 |
| Runs threshold | 3 |
| Input files | NEREA\_1\_3\_100610\_001\_IA\_workflow.xml  NEREA\_1\_4\_100610\_001\_IA\_workflow.xml  NEREA\_1\_5\_100610\_001\_IA\_workflow.xml  NEREA\_1\_6\_100610\_001\_IA\_workflow.xml |
| Input file type | Waters PLGS |
| Peptide threshold | Yellow |

  

Protein Summary

| Maximum | 265 |
| Conclusive | 79 |
| Indistinguishable | 101 in 31 groups |
| Ambiguous groups | 15 in 3 groups |
| Non conclusive | 70 |
| Filtered | 215 |

  

Protein List

| Name | Evidence | Peptide list (unique, meaningful\*, meaningless\*\*) | | Description |
| H4\_HUMAN | Conclusive | 45, 317, 481, 698, 894 | | Histone H4 OS Homo sapiens GN HIST1H4A PE 1 SV 2 |
| RLA2\_HUMAN | Conclusive | 488, 770, 774 | | 60S acidic ribosomal protein P2 OS Homo sapiens GN RPLP2 PE 1 SV 1 |
| ACTG\_HUMAN | Conclusive | 51\*\*, 82\*\*, 121\*\*, 134\*\*, 140\*\*, 141\*\*, 216\*\*, 288\*\*, 431\*\*, 441, 614\*\*, 766\*\*, 857\*\*, 896\*\*, 1049\*\*, 1050\*\* | | Actin cytoplasmic 2 OS Homo sapiens GN ACTG1 PE 1 SV 1 |
| HSP71\_HUMAN | Conclusive | 20\*\*, 59\*\*, 86, 103\*\*, 178\*\*, 232\*\*, 379, 502, 506\*\*, 519, 573, 773\*\*, 777, 781\*\*, 812\*\*, 876, 914, 939, 1064, 1352\*\*, 1434 | | Heat shock 70 kDa protein 1A 1B OS Homo sapiens GN HSPA1A PE 1 SV 5 |
| CH10\_HUMAN | Conclusive | 203, 371, 456, 821 | | 10 kDa heat shock protein mitochondrial OS Homo sapiens GN HSPE1 PE 1 SV 2 |
| HS90B\_HUMAN | Conclusive | 50, 99, 147\*\*, 157\*\*, 183\*\*, 236\*\*, 243\*\*, 249\*\*, 407\*\*, 432\*\*, 535\*\*, 554, 595, 619\*\*, 638\*\*, 689\*\*, 706\*\*, 748, 933\*\*, 1302\*\* | | Heat shock protein HSP 90 beta OS Homo sapiens GN HSP90AB1 PE 1 SV 4 |
| TBB5\_HUMAN | Conclusive | 42\*\*, 337, 368\*\*, 596\*\*, 640\*\*, 796\*\*, 906\*\*, 941, 995\*\*, 1034\*\*, 1080\*\* | | Tubulin beta chain OS Homo sapiens GN TUBB PE 1 SV 2 |
| TBB4B\_HUMAN | Conclusive | 42\*\*, 368\*\*, 596\*\*, 640\*\*, 796\*\*, 906\*\*, 963\*\*, 995\*\*, 1034\*\*, 1080\*\*, 1304 | | Tubulin beta 4B chain OS Homo sapiens GN TUBB4B PE 1 SV 1 |
| RLA1\_HUMAN | Conclusive | 917, 1066 | | 60S acidic ribosomal protein P1 OS Homo sapiens GN RPLP1 PE 1 SV 1 |
| HSP7C\_HUMAN | Conclusive | 19, 59\*\*, 68\*\*, 178\*\*, 321\*\*, 360\*\*, 457\*\*, 506\*\*, 543\*\*, 716\*\*, 720\*\*, 749\*\*, 794\*\*, 840\*\*, 853, 1042\*\* | | Heat shock cognate 71 kDa protein OS Homo sapiens GN HSPA8 PE 1 SV 1 |
| LDHB\_HUMAN | Conclusive | 180\*\*, 184, 213\*\*, 312, 335, 464, 613, 800, 1095 | | L lactate dehydrogenase B chain OS Homo sapiens GN LDHB PE 1 SV 2 |
| RAN\_HUMAN | Conclusive | 324, 560, 632 | | GTP binding nuclear protein Ran OS Homo sapiens GN RAN PE 1 SV 3 |
| HSP76\_HUMAN | Conclusive | 59\*\*, 103\*\*, 232\*\*, 506\*\*, 773\*\*, 781\*\*, 983, 1352\*\*, 1364, 1414 | | Heat shock 70 kDa protein 6 OS Homo sapiens GN HSPA6 PE 1 SV 2 |
| TBB4A\_HUMAN | Conclusive | 596\*\*, 640\*\*, 738, 796\*\*, 906\*\*, 963\*\*, 995\*\*, 1080\*\* | | Tubulin beta 4A chain OS Homo sapiens GN TUBB4A PE 1 SV 2 |
| ROA2\_HUMAN | Conclusive | 78\*\*, 269\*\*, 341\*\*, 393\*\*, 395, 680\*\*, 756\*\* | | Heterogeneous nuclear ribonucleoproteins A2 B1 OS Homo sapiens GN HNRNPA2B1 PE 1 SV 2 |
| TRYP\_PIG | Conclusive | 73, 176, 327, 708, 755, 874, 923, 1370 | | Trypsin OS Sus scrofa PE 1 SV 1 |
| PRDX1\_HUMAN | Conclusive | 70\*\*, 126, 304, 346, 353, 463, 534\*\*, 710\*\* | | Peroxiredoxin 1 OS Homo sapiens GN PRDX1 PE 1 SV 1 |
| G3P\_HUMAN | Conclusive | 367, 581, 615, 815, 829, 947 | | Glyceraldehyde 3 phosphate dehydrogenase OS Homo sapiens GN GAPDH PE 1 SV 3 |
| CH60\_HUMAN | Conclusive | 54, 84, 107, 156, 160, 454, 475, 662, 711, 714, 818, 833, 892, 1155 | | 60 kDa heat shock protein mitochondrial OS Homo sapiens GN HSPD1 PE 1 SV 2 |
| PROF1\_HUMAN | Conclusive | 94, 196, 451, 562, 961, 1477 | | Profilin 1 OS Homo sapiens GN PFN1 PE 1 SV 2 |
| 1433T\_HUMAN | Conclusive | 57\*\*, 88\*\*, 224, 332, 538\*\*, 1030\*\*, 1089 | | 14 3 3 protein theta OS Homo sapiens GN YWHAQ PE 1 SV 1 |
| 1433Z\_HUMAN | Conclusive | 57\*\*, 127, 175, 538\*\*, 1030\*\*, 1096, 1123 | | 14 3 3 protein zeta delta OS Homo sapiens GN YWHAZ PE 1 SV 1 |
| HS71L\_HUMAN | Conclusive | 20\*\*, 59\*\*, 178\*\*, 232\*\*, 380, 506\*\*, 749\*\*, 812\*\*, 1352\*\* | | Heat shock 70 kDa protein 1 like OS Homo sapiens GN HSPA1L PE 1 SV 2 |
| KCRB\_HUMAN | Conclusive | 234, 283, 608, 690, 746, 768, 865, 1046, 1551 | | Creatine kinase B type OS Homo sapiens GN CKB PE 1 SV 1 |
| ENOA\_HUMAN | Conclusive | 56, 80\*\*, 227\*\*, 300, 489\*\*, 498\*\*, 783\*\*, 870\*\*, 949\*\*, 1269\*\*, 1675\*\* | | Alpha enolase OS Homo sapiens GN ENO1 PE 1 SV 2 |
| H2AJ\_HUMAN | Conclusive | 298\*\*, 551\*\*, 731 | | Isoform 2 of Histone H2A J OS Homo sapiens GN H2AFJ |
| RS7\_HUMAN | Conclusive | 1052, 1111 | | 40S ribosomal protein S7 OS Homo sapiens GN RPS7 PE 1 SV 1 |
| 1433F\_HUMAN | Conclusive | 88\*\*, 262, 382, 538\*\*, 1030\*\* | | 14 3 3 protein eta OS Homo sapiens GN YWHAH PE 1 SV 4 |
| PPIA\_HUMAN | Conclusive | 566, 673, 742\*\*, 789, 1013 | | Peptidyl prolyl cis trans isomerase A OS Homo sapiens GN PPIA PE 1 SV 2 |
| RSSA\_HUMAN | Conclusive | 275, 365, 729, 930 | | 40S ribosomal protein SA OS Homo sapiens GN RPSA PE 1 SV 4 |
| PRDX2\_HUMAN | Conclusive | 534\*\*, 1043 | | Peroxiredoxin 2 OS Homo sapiens GN PRDX2 PE 1 SV 5 |
| MDHM\_HUMAN | Conclusive | 624, 905, 912, 1092 | | Malate dehydrogenase mitochondrial OS Homo sapiens GN MDH2 PE 1 SV 3 |
| 1433G\_HUMAN | Conclusive | 88\*\*, 538\*\*, 1030\*\*, 1153 | | 14 3 3 protein gamma OS Homo sapiens GN YWHAG PE 1 SV 2 |
| VIME\_HUMAN | Conclusive | 91, 210, 289, 342\*\*, 375, 403, 791, 893, 1410\*\*, 1431 | | Vimentin OS Homo sapiens GN VIM PE 1 SV 4 |
| EF2\_HUMAN | Conclusive | 60, 165, 233\*\*, 307, 401, 416, 419, 462, 520, 530, 604, 946, 1084, 1152, 1599 | | Elongation factor 2 OS Homo sapiens GN EEF2 PE 1 SV 4 |
| ALDOA\_HUMAN | Conclusive | 28, 100, 106, 132, 137, 385, 418, 527, 899, 1249 | | Fructose bisphosphate aldolase A OS Homo sapiens GN ALDOA PE 1 SV 2 |
| PGAM1\_HUMAN | Conclusive | 179\*\*, 271, 633, 1022\*\*, 1194\*\* | | Phosphoglycerate mutase 1 OS Homo sapiens GN PGAM1 PE 1 SV 2 |
| GRP78\_HUMAN | Conclusive | 178\*\*, 499, 606, 607, 625, 715, 749\*\* | | 78 kDa glucose regulated protein OS Homo sapiens GN HSPA5 PE 1 SV 2 |
| RLA0\_HUMAN | Conclusive | 52\*\*, 553\*\*, 735\*\*, 953, 960\*\*, 976\*\* | | 60S acidic ribosomal protein P0 OS Homo sapiens GN RPLP0 PE 1 SV 1 |
| EF1G\_HUMAN | Conclusive | 295, 357, 364, 417, 460, 739, 822, 925, 1015 | | Elongation factor 1 gamma OS Homo sapiens GN EEF1G PE 1 SV 3 |
| THIO\_HUMAN | Conclusive | 48, 600 | | Thioredoxin OS Homo sapiens GN TXN PE 1 SV 3 |
| PGK1\_HUMAN | Conclusive | 53\*\*, 318, 594, 863, 885, 929, 934 | | Phosphoglycerate kinase 1 OS Homo sapiens GN PGK1 PE 1 SV 3 |
| COF1\_HUMAN | Conclusive | 910 | | Cofilin 1 OS Homo sapiens GN CFL1 PE 1 SV 3 |
| SERA\_HUMAN | Conclusive | 410, 521, 745, 1040, 1065, 1120, 1261 | | D 3 phosphoglycerate dehydrogenase OS Homo sapiens GN PHGDH PE 1 SV 4 |
| PRDX6\_HUMAN | Conclusive | 544, 611, 695, 805, 913 | | Peroxiredoxin 6 OS Homo sapiens GN PRDX6 PE 1 SV 3 |
| PCBP2\_HUMAN | Conclusive | 173\*\*, 526\*\*, 850, 1004 | | Poly rC binding protein 2 OS Homo sapiens GN PCBP2 PE 1 SV 1 |
| GSTP1\_HUMAN | Conclusive | 878 | | Glutathione S transferase P OS Homo sapiens GN GSTP1 PE 1 SV 2 |
| ENPL\_HUMAN | Conclusive | 32, 404, 432\*\*, 571, 694 | | Endoplasmin OS Homo sapiens GN HSP90B1 PE 1 SV 1 |
| KPYM\_HUMAN | Conclusive | 136\*\*, 311\*\*, 504\*\*, 725\*\*, 907, 922\*\*, 1012\*\* | | Pyruvate kinase isozymes M1 M2 OS Homo sapiens GN PKM2 PE 1 SV 4 |
| IF4A1\_HUMAN | Conclusive | 93\*\*, 349\*\*, 359\*\*, 402, 753\*\*, 1643 | | Eukaryotic initiation factor 4A I OS Homo sapiens GN EIF4A1 PE 1 SV 1 |
| HNRH1\_HUMAN | Conclusive | 185\*\*, 272\*\*, 550, 724, 880\*\*, 1057\*\* | | Heterogeneous nuclear ribonucleoprotein H OS Homo sapiens GN HNRNPH1 PE 1 SV 4 |
| ANXA5\_HUMAN | Conclusive | 479, 855, 1109 | | Annexin A5 OS Homo sapiens GN ANXA5 PE 1 SV 2 |
| SET\_HUMAN | Conclusive | 211\*\*, 378\*\*, 1169 | | Isoform 2 of Protein SET OS Homo sapiens GN SET |
| ENO1\_YEAST | Conclusive | 438, 486, 659, 668 | | Enolase 1 OS Saccharomyces cerevisiae GN ENO1 PE 1 SV 2 |
| PDIA3\_HUMAN | Conclusive | 158, 212, 221, 389, 984, 1267 | | Protein disulfide isomerase A3 OS Homo sapiens GN PDIA3 PE 1 SV 4 |
| ATPA\_HUMAN | Conclusive | 253, 452, 546, 635, 1146 | | ATP synthase subunit alpha mitochondrial OS Homo sapiens GN ATP5A1 PE 1 SV 1 |
| HNRPF\_HUMAN | Conclusive | 272\*\*, 974, 1057\*\* | | Heterogeneous nuclear ribonucleoprotein F OS Homo sapiens GN HNRNPF PE 1 SV 3 |
| NUCL\_HUMAN | Conclusive | 39, 113, 217, 423, 474, 509 | | Nucleolin OS Homo sapiens GN NCL PE 1 SV 3 |
| ALBU\_HUMAN | Conclusive | 133\*\*, 153\*\*, 205\*\*, 446, 511\*\* | | Serum albumin OS Homo sapiens GN ALB PE 1 SV 2 |
| TCPA\_HUMAN | Conclusive | 95, 323, 682 | | T complex protein 1 subunit alpha OS Homo sapiens GN TCP1 PE 1 SV 1 |
| UBA1\_HUMAN | Conclusive | 305, 428, 589, 832, 881, 924, 1009, 1032 | | Ubiquitin like modifier activating enzyme 1 OS Homo sapiens GN UBA1 PE 1 SV 3 |
| G6PI\_HUMAN | Conclusive | 261, 373, 1006 | | Glucose 6 phosphate isomerase OS Homo sapiens GN GPI PE 1 SV 4 |
| FETUA\_HUMAN | Conclusive | 15, 43, 193 | | Alpha 2 HS glycoprotein OS Homo sapiens GN AHSG PE 1 SV 1 |
| ADT2\_HUMAN | Conclusive | 118\*\*, 528\*\*, 754, 864\*\*, 980\*\* | | ADP ATP translocase 2 OS Homo sapiens GN SLC25A5 PE 1 SV 7 |
| TCPQ\_HUMAN | Conclusive | 660, 757 | | T complex protein 1 subunit theta OS Homo sapiens GN CCT8 PE 1 SV 4 |
| HSP74\_HUMAN | Conclusive | 1033, 1071 | | Heat shock 70 kDa protein 4 OS Homo sapiens GN HSPA4 PE 1 SV 4 |
| EFTU\_HUMAN | Conclusive | 820, 1018 | | Elongation factor Tu mitochondrial OS Homo sapiens GN TUFM PE 1 SV 2 |
| PLST\_HUMAN | Conclusive | 21\*\*, 717, 1112, 1150, 1543\*\* | | Plastin 3 OS Homo sapiens GN PLS3 PE 1 SV 4 |
| IMB1\_HUMAN | Conclusive | 1062, 1106, 1149, 1229 | | Importin subunit beta 1 OS Homo sapiens GN KPNB1 PE 1 SV 2 |
| STIP1\_HUMAN | Conclusive | 138, 215, 645 | | Stress induced phosphoprotein 1 OS Homo sapiens GN STIP1 PE 1 SV 1 |
| PARP1\_HUMAN | Conclusive | 277, 875 | | Poly ADP ribose polymerase 1 OS Homo sapiens GN PARP1 PE 1 SV 4 |
| DHX9\_HUMAN | Conclusive | 965, 1154\*\* | | ATP dependent RNA helicase A OS Homo sapiens GN DHX9 PE 1 SV 4 |
| TCPZ\_HUMAN | Conclusive | 1079 | | T complex protein 1 subunit zeta OS Homo sapiens GN CCT6A PE 1 SV 3 |
| MATR3\_HUMAN | Conclusive | 1107 | | Matrin 3 OS Homo sapiens GN MATR3 PE 1 SV 2 |
| GRP75\_HUMAN | Conclusive | 27, 653, 1297, 1607 | | Stress 70 protein mitochondrial OS Homo sapiens GN HSPA9 PE 1 SV 2 |
| TCPB\_HUMAN | Conclusive | 1685 | | T complex protein 1 subunit beta OS Homo sapiens GN CCT2 PE 1 SV 4 |
| XRCC5\_HUMAN | Conclusive | 31 | | X ray repair cross complementing protein 5 OS Homo sapiens GN XRCC5 PE 1 SV 3 |
| TCPH\_HUMAN | Conclusive | 74 | | T complex protein 1 subunit eta OS Homo sapiens GN CCT7 PE 1 SV 2 |
| C1QBP\_HUMAN | Conclusive | 1602, 1603 | | Complement component 1 Q subcomponent binding protein mitochondrial OS Homo sapiens GN C1QBP PE 1 S |
| GROUP001 | Indistinguishable | UBC\_HUMAN: | 24\*, 238\*, 439\*, 487\*, 565\* | Polyubiquitin C OS Homo sapiens GN UBC PE 1 SV 2 |
| RL40\_HUMAN: | 24\*, 238\*, 439\*, 487\*, 565\* | Ubiquitin 60S ribosomal protein L40 OS Homo sapiens GN UBA52 PE 1 SV 2 |
| UBB\_HUMAN: | 24\*, 238\*, 439\*, 487\*, 565\* | Polyubiquitin B OS Homo sapiens GN UBB PE 1 SV 1 |
| RS27A\_HUMAN: | 24\*, 238\*, 439\*, 487\*, 565\* | Ubiquitin 40S ribosomal protein S27a OS Homo sapiens GN RPS27A PE 1 SV 2 |
| GROUP002 | Indistinguishable | ACTA\_HUMAN: | 51\*\*, 121\*\*, 134\*\*, 140\*\*, 141\*\*, 431\*\*, 700\*, 766\*\* | Actin aortic smooth muscle OS Homo sapiens GN ACTA2 PE 1 SV 1 |
| ACTC\_HUMAN: | 51\*\*, 121\*\*, 134\*\*, 140\*\*, 141\*\*, 431\*\*, 700\*, 766\*\* | Actin alpha cardiac muscle 1 OS Homo sapiens GN ACTC1 PE 1 SV 1 |
| ACTS\_HUMAN: | 51\*\*, 121\*\*, 134\*\*, 140\*\*, 141\*\*, 431\*\*, 700\*, 766\*\* | Actin alpha skeletal muscle OS Homo sapiens GN ACTA1 PE 1 SV 1 |
| ACTH\_HUMAN: | 51\*\*, 121\*\*, 134\*\*, 140\*\*, 141\*\*, 431\*\*, 700\*, 766\*\* | Actin gamma enteric smooth muscle OS Homo sapiens GN ACTG2 PE 1 SV 1 |
| GROUP003 | Indistinguishable | POTEF\_HUMAN: | 121\*\*, 140\*\*, 216\*\*, 539\*, 766\*\* | POTE ankyrin domain family member F OS Homo sapiens GN POTEF PE 1 SV 2 |
| POTEE\_HUMAN: | 121\*\*, 140\*\*, 216\*\*, 539\*, 766\*\*, 1050\*\* | POTE ankyrin domain family member E OS Homo sapiens GN POTEE PE 1 SV 3 |
| POTEJ\_HUMAN: | 121\*\*, 140\*\*, 216\*\*, 539\* | POTE ankyrin domain family member J OS Homo sapiens GN POTEJ PE 3 SV 1 |
| POTEI\_HUMAN: | 121\*\*, 140\*\*, 216\*\*, 539\* | POTE ankyrin domain family member I OS Homo sapiens GN POTEI PE 3 SV 1 |
| GROUP004 | Indistinguishable | H2BFS\_HUMAN: | 25\*\*, 46\*\*, 309\*, 652\*\*, 1119\*\* | Histone H2B type F S OS Homo sapiens GN H2BFS PE 1 SV 2 |
| H2B1N\_HUMAN: | 25\*\*, 46\*\*, 309\*, 652\*\*, 1119\*\* | Histone H2B type 1 N OS Homo sapiens GN HIST1H2BN PE 1 SV 3 |
| H2B1M\_HUMAN: | 25\*\*, 46\*\*, 309\*, 652\*\*, 1119\*\* | Histone H2B type 1 M OS Homo sapiens GN HIST1H2BM PE 1 SV 3 |
| H2B2F\_HUMAN: | 25\*\*, 46\*\*, 309\*, 652\*\*, 1119\*\* | Histone H2B type 2 F OS Homo sapiens GN HIST2H2BF PE 1 SV 3 |
| H2B1K\_HUMAN: | 25\*\*, 46\*\*, 309\*, 652\*\*, 1119\*\* | Histone H2B type 1 K OS Homo sapiens GN HIST1H2BK PE 1 SV 3 |
| H2B1L\_HUMAN: | 25\*\*, 46\*\*, 309\*, 652\*\*, 1119\*\* | Histone H2B type 1 L OS Homo sapiens GN HIST1H2BL PE 1 SV 3 |
| H2B1D\_HUMAN: | 25\*\*, 46\*\*, 309\*, 652\*\*, 1119\*\* | Histone H2B type 1 D OS Homo sapiens GN HIST1H2BD PE 1 SV 2 |
| H2B1C\_HUMAN: | 25\*\*, 46\*\*, 309\*, 652\*\*, 1119\*\* | Histone H2B type 1 C E F G I OS Homo sapiens GN HIST1H2BC PE 1 SV 4 |
| H2B1H\_HUMAN: | 25\*\*, 46\*\*, 309\*, 652\*\*, 1119\*\* | Histone H2B type 1 H OS Homo sapiens GN HIST1H2BH PE 1 SV 3 |
| GROUP005 | Indistinguishable | H2B1B\_HUMAN: | 25\*\*, 46\*\*, 507\*, 652\*\*, 1119\*\* | Histone H2B type 1 B OS Homo sapiens GN HIST1H2BB PE 1 SV 2 |
| H2B2E\_HUMAN: | 25\*\*, 46\*\*, 507\*, 652\*\*, 1119\*\* | Histone H2B type 2 E OS Homo sapiens GN HIST2H2BE PE 1 SV 3 |
| H2B1J\_HUMAN: | 25\*\*, 46\*\*, 507\*, 652\*\*, 1119\*\* | Histone H2B type 1 J OS Homo sapiens GN HIST1H2BJ PE 1 SV 3 |
| H2B1O\_HUMAN: | 25\*\*, 46\*\*, 507\*, 652\*\*, 1119\*\* | Histone H2B type 1 O OS Homo sapiens GN HIST1H2BO PE 1 SV 3 |
| H2B3B\_HUMAN: | 25\*\*, 507\*, 652\*\*, 1119\*\* | Histone H2B type 3 B OS Homo sapiens GN HIST3H2BB PE 1 SV 3 |
| H2B2C\_HUMAN: | 507\* | Putative histone H2B type 2 C OS Homo sapiens GN HIST2H2BC PE 5 SV 3 |
| H2B2D\_HUMAN: | 507\* | Putative histone H2B type 2 D OS Homo sapiens GN HIST2H2BD PE 5 SV 3 |
| GROUP006 | Indistinguishable | HS90A\_HUMAN: | 38\*, 146\*, 147\*\*, 183\*\*, 236\*\*, 243\*\*, 255\*\*, 264\*, 598\*\*, 619\*\*, 638\*\*, 651\*, 679\*, 689\*\*, 784\*, 986\*\*, 1302\*\*, 1557\*, 1666\* | Heat shock protein HSP 90 alpha OS Homo sapiens GN HSP90AA1 PE 1 SV 5 |
| HS90A\_HUMAN: | 38\*, 146\*, 147\*\*, 183\*\*, 236\*\*, 243\*\*, 255\*\*, 264\*, 598\*\*, 619\*\*, 638\*\*, 651\*, 679\*, 689\*\*, 784\*, 986\*\*, 1302\*\*, 1557\*, 1666\* | Isoform 2 of Heat shock protein HSP 90 alpha OS Homo sapiens GN HSP90AA1 |
| GROUP007 | Indistinguishable | TBB8\_HUMAN: | 42\*\*, 877\*, 995\*\* | Tubulin beta 8 chain OS Homo sapiens GN TUBB8 PE 1 SV 2 |
| YI016\_HUMAN: | 42\*\*, 877\*, 995\*\* | Putative tubulin beta chain like protein ENSP00000290377 OS Homo sapiens PE 5 SV 2 |
| TBB8B\_HUMAN: | 42\*\*, 877\*, 995\*\* | Tubulin beta 8 chain B OS Homo sapiens PE 1 SV 1 |
| GROUP008 | Indistinguishable | TPIS\_HUMAN: | 17\*, 119\*, 143\*, 150\*, 274\*, 421\*, 858\*, 882\*, 927\*, 1081\* | Triosephosphate isomerase OS Homo sapiens GN TPI1 PE 1 SV 3 |
| TPIS\_HUMAN: | 17\*, 119\*, 143\*, 150\*, 274\*, 421\*, 858\*, 882\*, 927\*, 1081\* | Isoform 2 of Triosephosphate isomerase OS Homo sapiens GN TPI1 |
| GROUP009 | Indistinguishable | H31T\_HUMAN: | 120\*\*, 171\*, 172\*\*, 388\*\* | Histone H3 1t OS Homo sapiens GN HIST3H3 PE 1 SV 3 |
| H32\_HUMAN: | 120\*\*, 171\*, 172\*\*, 388\*\* | Histone H3 2 OS Homo sapiens GN HIST2H3A PE 1 SV 3 |
| H33\_HUMAN: | 120\*\*, 171\*, 172\*\*, 388\*\* | Histone H3 3 OS Homo sapiens GN H3F3A PE 1 SV 2 |
| H31\_HUMAN: | 120\*\*, 171\*, 172\*\*, 388\*\* | Histone H3 1 OS Homo sapiens GN HIST1H3A PE 1 SV 2 |
| GROUP010 | Indistinguishable | LDHA\_HUMAN: | 135\*\*, 151\*\*, 180\*\*, 213\*\*, 425\*\*, 501\*, 576\*\*, 713\*\*, 890\*\* | L lactate dehydrogenase A chain OS Homo sapiens GN LDHA PE 1 SV 2 |
| LDHA\_HUMAN: | 135\*\*, 151\*\*, 180\*\*, 213\*\*, 425\*\*, 501\*, 576\*\*, 713\*\*, 890\*\* | Isoform 3 of L lactate dehydrogenase A chain OS Homo sapiens GN LDHA |
| GROUP011 | Indistinguishable | NDKB\_HUMAN: | 63\*\*, 347\*\*, 480\*\*, 577\*\*, 579\*\*, 688\*\*, 1010\* | Isoform 3 of Nucleoside diphosphate kinase B OS Homo sapiens GN NME2 |
| NDKB\_HUMAN: | 63\*\*, 347\*\*, 480\*\*, 577\*\*, 688\*\*, 1010\* | Nucleoside diphosphate kinase B OS Homo sapiens GN NME2 PE 1 SV 1 |
| GROUP012 | Indistinguishable | NDKA\_HUMAN: | 149\*, 347\*\*, 480\*\*, 577\*\*, 579\*\* | Nucleoside diphosphate kinase A OS Homo sapiens GN NME1 PE 1 SV 1 |
| NDKA\_HUMAN: | 149\*, 347\*\*, 480\*\*, 577\*\*, 579\*\* | Isoform 2 of Nucleoside diphosphate kinase A OS Homo sapiens GN NME1 |
| GROUP013 | Indistinguishable | 1433E\_HUMAN: | 112\*, 297\*, 434\*, 538\*\*, 1005\*, 1030\*\*, 1074\* | 14 3 3 protein epsilon OS Homo sapiens GN YWHAE PE 1 SV 1 |
| 1433E\_HUMAN: | 112\*, 297\*, 434\*, 538\*\*, 1005\*, 1030\*\*, 1074\* | Isoform SV of 14 3 3 protein epsilon OS Homo sapiens GN YWHAE |
| GROUP014 | Indistinguishable | 1433B\_HUMAN: | 57\*\*, 66\*, 88\*\*, 538\*\*, 1030\*\*, 1070\* | 14 3 3 protein beta alpha OS Homo sapiens GN YWHAB PE 1 SV 3 |
| 1433B\_HUMAN: | 57\*\*, 66\*, 88\*\*, 538\*\*, 1030\*\*, 1070\* | Isoform Short of 14 3 3 protein beta alpha OS Homo sapiens GN YWHAB |
| GROUP015 | Indistinguishable | 1433S\_HUMAN: | 538\*\*, 807\*, 1030\*\*, 1253\* | 14 3 3 protein sigma OS Homo sapiens GN SFN PE 1 SV 1 |
| 1433S\_HUMAN: | 538\*\*, 807\*, 1030\*\*, 1253\* | Isoform 2 of 14 3 3 protein sigma OS Homo sapiens GN SFN |
| GROUP016 | Indistinguishable | ROA1\_HUMAN: | 8\*, 13\*\*, 37\*\*, 61\*\*, 269\*\*, 741\*\*, 782\*\* | Heterogeneous nuclear ribonucleoprotein A1 OS Homo sapiens GN HNRNPA1 PE 1 SV 5 |
| ROA1\_HUMAN: | 8\*, 13\*\*, 37\*\*, 61\*\*, 269\*\*, 741\*\*, 782\*\* | Isoform A1 A of Heterogeneous nuclear ribonucleoprotein A1 OS Homo sapiens GN HNRNPA1 |
| GROUP019 | Indistinguishable | HNRPK\_HUMAN: | 144\*\*, 226\*\*, 240\*\*, 314\*\*, 329\*\*, 798\*\*, 967\*\*, 971\*\*, 1038\* | Heterogeneous nuclear ribonucleoprotein K OS Homo sapiens GN HNRNPK PE 1 SV 1 |
| HNRPK\_HUMAN: | 144\*\*, 226\*\*, 240\*\*, 314\*\*, 329\*\*, 798\*\*, 967\*\*, 971\*\*, 1038\* | Isoform 2 of Heterogeneous nuclear ribonucleoprotein K OS Homo sapiens GN HNRNPK |
| GROUP020 | Indistinguishable | H2A1A\_HUMAN: | 298\*\*, 551\*\*, 1055\* | Histone H2A type 1 A OS Homo sapiens GN HIST1H2AA PE 1 SV 3 |
| H2AX\_HUMAN: | 298\*\*, 551\*\*, 1055\* | Histone H2A x OS Homo sapiens GN H2AFX PE 1 SV 2 |
| H2A1J\_HUMAN: | 298\*\*, 551\*\*, 1055\* | Histone H2A type 1 J OS Homo sapiens GN HIST1H2AJ PE 1 SV 3 |
| H2A2A\_HUMAN: | 298\*\*, 551\*\*, 1055\* | Histone H2A type 2 A OS Homo sapiens GN HIST2H2AA3 PE 1 SV 3 |
| H2A3\_HUMAN: | 298\*\*, 551\*\*, 1055\* | Histone H2A type 3 OS Homo sapiens GN HIST3H2A PE 1 SV 3 |
| H2A1B\_HUMAN: | 298\*\*, 551\*\*, 1055\* | Histone H2A type 1 B E OS Homo sapiens GN HIST1H2AB PE 1 SV 2 |
| H2A2C\_HUMAN: | 298\*\*, 551\*\*, 1055\* | Histone H2A type 2 C OS Homo sapiens GN HIST2H2AC PE 1 SV 4 |
| H2A1C\_HUMAN: | 298\*\*, 551\*\*, 1055\* | Histone H2A type 1 C OS Homo sapiens GN HIST1H2AC PE 1 SV 3 |
| H2AJ\_HUMAN: | 298\*\*, 551\*\*, 1055\* | Histone H2A J OS Homo sapiens GN H2AFJ PE 1 SV 1 |
| H2A1\_HUMAN: | 298\*\*, 551\*\*, 1055\* | Histone H2A type 1 OS Homo sapiens GN HIST1H2AG PE 1 SV 2 |
| H2A1D\_HUMAN: | 298\*\*, 551\*\*, 1055\* | Histone H2A type 1 D OS Homo sapiens GN HIST1H2AD PE 1 SV 2 |
| H2A1H\_HUMAN: | 298\*\*, 551\*\*, 1055\* | Histone H2A type 1 H OS Homo sapiens GN HIST1H2AH PE 1 SV 3 |
| H2A2B\_HUMAN: | 551\*\*, 1055\* | Histone H2A type 2 B OS Homo sapiens GN HIST2H2AB PE 1 SV 3 |
| GROUP021 | Indistinguishable | NPM\_HUMAN: | 71\*, 124\*, 195\*, 336\*, 856\*, 1073\* | Nucleophosmin OS Homo sapiens GN NPM1 PE 1 SV 2 |
| NPM\_HUMAN: | 71\*, 124\*, 195\*, 336\*, 856\*, 1073\* | Isoform 2 of Nucleophosmin OS Homo sapiens GN NPM1 |
| GROUP023 | Indistinguishable | HNRPU\_HUMAN: | 248\*, 392\*, 610\*, 814\*, 1008\*, 1277\* | Heterogeneous nuclear ribonucleoprotein U OS Homo sapiens GN HNRNPU PE 1 SV 6 |
| HNRPU\_HUMAN: | 248\*, 392\*, 610\*, 814\*, 1008\*, 1277\* | Isoform Short of Heterogeneous nuclear ribonucleoprotein U OS Homo sapiens GN HNRNPU |
| GROUP024 | Indistinguishable | HNRPC\_HUMAN: | 415\*\*, 692\*\*, 752\*, 835\*\* | Heterogeneous nuclear ribonucleoproteins C1 C2 OS Homo sapiens GN HNRNPC PE 1 SV 4 |
| HNRPC\_HUMAN: | 415\*\*, 692\*\*, 752\*, 835\*\* | Isoform 4 of Heterogeneous nuclear ribonucleoproteins C1 C2 OS Homo sapiens GN HNRNPC |
| HNRPC\_HUMAN: | 415\*\*, 692\*\*, 752\*, 835\*\* | Isoform C1 of Heterogeneous nuclear ribonucleoproteins C1 C2 OS Homo sapiens GN HNRNPC |
| GROUP025 | Indistinguishable | HNRPD\_HUMAN: | 563\*, 887\*, 1629\*\* | Heterogeneous nuclear ribonucleoprotein D0 OS Homo sapiens GN HNRNPD PE 1 SV 1 |
| HNRPD\_HUMAN: | 563\*, 887\*, 1629\*\* | Isoform 4 of Heterogeneous nuclear ribonucleoprotein D0 OS Homo sapiens GN HNRNPD |
| HNRPD\_HUMAN: | 563\*, 887\*, 1629\*\* | Isoform 3 of Heterogeneous nuclear ribonucleoprotein D0 OS Homo sapiens GN HNRNPD |
| HNRPD\_HUMAN: | 563\*, 887\*, 1629\*\* | Isoform 2 of Heterogeneous nuclear ribonucleoprotein D0 OS Homo sapiens GN HNRNPD |
| GROUP026 | Indistinguishable | TCPG\_HUMAN: | 631\*, 854\*, 969\* | T complex protein 1 subunit gamma OS Homo sapiens GN CCT3 PE 1 SV 4 |
| TCPG\_HUMAN: | 631\*, 854\*, 969\* | Isoform 2 of T complex protein 1 subunit gamma OS Homo sapiens GN CCT3 |
| GROUP027 | Indistinguishable | HNRPM\_HUMAN: | 220\*, 886\*, 999\* | Heterogeneous nuclear ribonucleoprotein M OS Homo sapiens GN HNRNPM PE 1 SV 3 |
| HNRPM\_HUMAN: | 220\*, 886\*, 999\* | Isoform 2 of Heterogeneous nuclear ribonucleoprotein M OS Homo sapiens GN HNRNPM |
| GROUP028 | Indistinguishable | DX39A\_HUMAN: | 333\*, 827\* | ATP dependent RNA helicase DDX39A OS Homo sapiens GN DDX39A PE 1 SV 2 |
| DX39B\_HUMAN: | 333\*, 827\* | Spliceosome RNA helicase DDX39B OS Homo sapiens GN DDX39B PE 1 SV 1 |
| DX39B\_HUMAN: | 333\*, 827\* | Isoform 2 of Spliceosome RNA helicase DDX39B OS Homo sapiens GN DDX39B |
| GROUP029 | Indistinguishable | PDIA6\_HUMAN: | 1021\* | Protein disulfide isomerase A6 OS Homo sapiens GN PDIA6 PE 1 SV 1 |
| PDIA6\_HUMAN: | 1021\* | Isoform 2 of Protein disulfide isomerase A6 OS Homo sapiens GN PDIA6 |
| GROUP030 | Indistinguishable | DDX17\_HUMAN: | 267\*, 575\*\* | Probable ATP dependent RNA helicase DDX17 OS Homo sapiens GN DDX17 PE 1 SV 1 |
| DDX17\_HUMAN: | 267\*, 575\*\* | Isoform 4 of Probable ATP dependent RNA helicase DDX17 OS Homo sapiens GN DDX17 |
| DDX17\_HUMAN: | 267\*, 575\*\* | Isoform 2 of Probable ATP dependent RNA helicase DDX17 OS Homo sapiens GN DDX17 |
| GROUP031 | Indistinguishable | AN32A\_HUMAN: | 884\*, 919\* | Acidic leucine rich nuclear phosphoprotein 32 family member A OS Homo sapiens GN ANP32A PE 1 SV 1 |
| AN32B\_HUMAN: | 884\*, 919\* | Isoform 2 of Acidic leucine rich nuclear phosphoprotein 32 family member B OS Homo sapiens GN ANP32B |
| AN32B\_HUMAN: | 884\*, 919\* | Acidic leucine rich nuclear phosphoprotein 32 family member B OS Homo sapiens GN ANP32B PE 1 SV 1 |
| GROUP032 | Indistinguishable | PUR6\_HUMAN: | 23\*, 1416\* | Multifunctional protein ADE2 OS Homo sapiens GN PAICS PE 1 SV 3 |
| PUR6\_HUMAN: | 23\*, 1416\* | Isoform 2 of Multifunctional protein ADE2 OS Homo sapiens GN PAICS |
| GROUP033 | Indistinguishable | PTBP1\_HUMAN: | 1035\*, 1054\*, 1579\* | Polypyrimidine tract binding protein 1 OS Homo sapiens GN PTBP1 PE 1 SV 1 |
| PTBP1\_HUMAN: | 1035\*, 1054\*, 1579\* | Isoform 2 of Polypyrimidine tract binding protein 1 OS Homo sapiens GN PTBP1 |
| GROUP034 | Indistinguishable | XPO2\_HUMAN: | 1720\* | Exportin 2 OS Homo sapiens GN CSE1L PE 1 SV 3 |
| XPO2\_HUMAN: | 1720\* | Isoform 2 of Exportin 2 OS Homo sapiens GN CSE1L |
| XPO2\_HUMAN: | 1720\* | Isoform 3 of Exportin 2 OS Homo sapiens GN CSE1L |
| GROUP017 | Group | EF1A2\_HUMAN: | 131\*\*, 273\*\*, 301\*\*, 405\*\*, 476\*\*, 634\* | Elongation factor 1 alpha 2 OS Homo sapiens GN EEF1A2 PE 1 SV 1 |
| EF1A1\_HUMAN: | 131\*\*, 166\*, 273\*\*, 301\*\*, 405\*\*, 476\*\*, 531\*, 634\*, 891\*, 1019\*, 1088\* | Elongation factor 1 alpha 1 OS Homo sapiens GN EEF1A1 PE 1 SV 1 |
| EF1A3\_HUMAN: | 131\*\*, 166\*, 273\*\*, 301\*\*, 405\*\*, 476\*\*, 531\*, 891\*, 1019\*, 1088\* | Putative elongation factor 1 alpha like 3 OS Homo sapiens GN EEF1A1P5 PE 5 SV 1 |
| GROUP018 | Group | TBA3C\_HUMAN: | 11\*\*, 164\*\*, 449\*\*, 473\*\*, 666\*\*, 681\*\*, 846\*\*, 849\*\*, 928\*, 1662\*\* | Isoform 2 of Tubulin alpha 3C D chain OS Homo sapiens GN TUBA3C |
| TBA1A\_HUMAN: | 11\*\*, 164\*\*, 174\*\*, 449\*\*, 473\*\*, 666\*\*, 681\*\*, 846\*\*, 849\*\*, 866\*, 928\*, 937\*, 1662\*\* | Tubulin alpha 1A chain OS Homo sapiens GN TUBA1A PE 1 SV 1 |
| TBA1C\_HUMAN: | 11\*\*, 164\*\*, 174\*\*, 449\*\*, 473\*\*, 666\*\*, 681\*\*, 846\*\*, 849\*\*, 866\*, 928\*, 937\*, 1114\*\*, 1662\*\* | Tubulin alpha 1C chain OS Homo sapiens GN TUBA1C PE 1 SV 1 |
| TBA3C\_HUMAN: | 11\*\*, 164\*\*, 449\*\*, 473\*\*, 666\*\*, 681\*\*, 846\*\*, 849\*\*, 928\*, 1662\*\* | Tubulin alpha 3C D chain OS Homo sapiens GN TUBA3C PE 1 SV 3 |
| TBA3E\_HUMAN: | 449\*\*, 473\*\*, 666\*\*, 681\*\*, 846\*\*, 849\*\*, 928\*, 1662\*\* | Tubulin alpha 3E chain OS Homo sapiens GN TUBA3E PE 1 SV 2 |
| TBA8\_HUMAN: | 11\*\*, 473\*\*, 666\*\*, 681\*\*, 928\*, 1114\*\*, 1662\*\* | Tubulin alpha 8 chain OS Homo sapiens GN TUBA8 PE 1 SV 1 |
| TBA1B\_HUMAN: | 11\*\*, 164\*\*, 174\*\*, 449\*\*, 473\*\*, 666\*\*, 681\*\*, 846\*\*, 849\*\*, 866\*, 920\*, 937\*, 1114\*\*, 1662\*\* | Tubulin alpha 1B chain OS Homo sapiens GN TUBA1B PE 1 SV 1 |
| TBA4A\_HUMAN: | 11\*\*, 164\*\*, 174\*\*, 473\*\*, 666\*\*, 681\*\*, 846\*\*, 920\*, 1114\*\*, 1662\*\* | Tubulin alpha 4A chain OS Homo sapiens GN TUBA4A PE 1 SV 1 |
| GROUP022 | Group | IF5A1\_HUMAN: | 130\*, 142\* | Eukaryotic translation initiation factor 5A 1 OS Homo sapiens GN EIF5A PE 1 SV 2 |
| IF5A1\_HUMAN: | 130\*, 142\* | Isoform 2 of Eukaryotic translation initiation factor 5A 1 OS Homo sapiens GN EIF5A |
| IF5A2\_HUMAN: | 130\* | Eukaryotic translation initiation factor 5A 2 OS Homo sapiens GN EIF5A2 PE 1 SV 3 |
| IF5AL\_HUMAN: | 142\* | Eukaryotic translation initiation factor 5A 1 like OS Homo sapiens GN EIF5AL1 PE 1 SV 2 |
| ACTBL\_HUMAN | NonConclusive | 82\*\*, 134\*\*, 140\*\*, 141\*\*, 766\*\* | | Beta actin like protein 2 OS Homo sapiens GN ACTBL2 PE 1 SV 2 |
| ACTB\_HUMAN | NonConclusive | 51\*\*, 82\*\*, 121\*\*, 134\*\*, 140\*\*, 141\*\*, 216\*\*, 288\*\*, 431\*\*, 614\*\*, 766\*\*, 857\*\*, 896\*\*, 1049\*\*, 1050\*\* | | Actin cytoplasmic 1 OS Homo sapiens GN ACTB PE 1 SV 1 |
| HSP77\_HUMAN | NonConclusive | 103\*\*, 232\*\*, 506\*\*, 781\*\* | | Putative heat shock 70 kDa protein 7 OS Homo sapiens GN HSPA7 PE 5 SV 2 |
| HSP7C\_HUMAN | NonConclusive | 68\*\*, 178\*\*, 321\*\*, 360\*\*, 457\*\*, 506\*\*, 543\*\*, 716\*\*, 720\*\*, 749\*\*, 794\*\*, 840\*\*, 1042\*\* | | Isoform 2 of Heat shock cognate 71 kDa protein OS Homo sapiens GN HSPA8 |
| TBB1\_HUMAN | NonConclusive | 42\*\* | | Tubulin beta 1 chain OS Homo sapiens GN TUBB1 PE 1 SV 1 |
| TBB2B\_HUMAN | NonConclusive | 42\*\*, 368\*\*, 596\*\*, 906\*\*, 995\*\*, 1034\*\*, 1080\*\* | | Tubulin beta 2B chain OS Homo sapiens GN TUBB2B PE 1 SV 1 |
| H3C\_HUMAN | NonConclusive | 120\*\*, 172\*\*, 388\*\* | | Histone H3 3C OS Homo sapiens GN H3F3C PE 1 SV 3 |
| H2B1A\_HUMAN | NonConclusive | 25\*\*, 46\*\*, 652\*\* | | Histone H2B type 1 A OS Homo sapiens GN HIST1H2BA PE 1 SV 3 |
| LDHA\_HUMAN | NonConclusive | 135\*\*, 151\*\*, 180\*\*, 213\*\*, 425\*\*, 576\*\*, 713\*\*, 890\*\* | | Isoform 2 of L lactate dehydrogenase A chain OS Homo sapiens GN LDHA |
| LDHC\_HUMAN | NonConclusive | 180\*\* | | L lactate dehydrogenase C chain OS Homo sapiens GN LDHC PE 2 SV 4 |
| NDK8\_HUMAN | NonConclusive | 63\*\*, 347\*\*, 480\*\*, 688\*\* | | Putative nucleoside diphosphate kinase OS Homo sapiens GN NME2P1 PE 5 SV 1 |
| TBB2A\_HUMAN | NonConclusive | 42\*\*, 368\*\*, 596\*\*, 906\*\*, 995\*\*, 1034\*\*, 1080\*\* | | Tubulin beta 2A chain OS Homo sapiens GN TUBB2A PE 1 SV 1 |
| H90B3\_HUMAN | NonConclusive | 147\*\*, 157\*\*, 236\*\*, 243\*\*, 249\*\*, 432\*\*, 535\*\*, 689\*\*, 933\*\* | | Putative heat shock protein HSP 90 beta 3 OS Homo sapiens GN HSP90AB3P PE 5 SV 1 |
| ROA2\_HUMAN | NonConclusive | 78\*\*, 269\*\*, 341\*\*, 393\*\*, 680\*\*, 756\*\* | | Isoform A2 of Heterogeneous nuclear ribonucleoproteins A2 B1 OS Homo sapiens GN HNRNPA2B1 |
| PRDX4\_HUMAN | NonConclusive | 70\*\*, 710\*\* | | Peroxiredoxin 4 OS Homo sapiens GN PRDX4 PE 1 SV 1 |
| TBA4B\_HUMAN | NonConclusive | 473\*\*, 1114\*\* | | Putative tubulin like protein alpha 4B OS Homo sapiens GN TUBA4B PE 5 SV 2 |
| HSP72\_HUMAN | NonConclusive | 59\*\*, 68\*\*, 178\*\*, 506\*\*, 749\*\*, 794\*\* | | Heat shock related 70 kDa protein 2 OS Homo sapiens GN HSPA2 PE 1 SV 1 |
| ENOA\_HUMAN | NonConclusive | 80\*\*, 227\*\*, 489\*\*, 498\*\*, 783\*\*, 949\*\*, 1269\*\*, 1675\*\* | | Isoform MBP 1 of Alpha enolase OS Homo sapiens GN ENO1 |
| ENOB\_HUMAN | NonConclusive | 870\*\* | | Beta enolase OS Homo sapiens GN ENO3 PE 1 SV 4 |
| ENOB\_HUMAN | NonConclusive | 870\*\* | | Isoform 2 of Beta enolase OS Homo sapiens GN ENO3 |
| ENOB\_HUMAN | NonConclusive | 870\*\* | | Isoform 3 of Beta enolase OS Homo sapiens GN ENO3 |
| HNRPK\_HUMAN | NonConclusive | 144\*\*, 226\*\*, 240\*\*, 314\*\*, 329\*\*, 798\*\*, 967\*\*, 971\*\* | | Isoform 3 of Heterogeneous nuclear ribonucleoprotein K OS Homo sapiens GN HNRNPK |
| ACTBM\_HUMAN | NonConclusive | 140\*\*, 216\*\*, 766\*\* | | Putative beta actin like protein 3 OS Homo sapiens GN POTEKP PE 5 SV 1 |
| ROA1\_HUMAN | NonConclusive | 13\*\*, 37\*\*, 61\*\*, 269\*\*, 741\*\*, 782\*\* | | Isoform 2 of Heterogeneous nuclear ribonucleoprotein A1 OS Homo sapiens GN HNRNPA1 |
| H2AV\_HUMAN | NonConclusive | 298\*\*, 551\*\* | | Histone H2A V OS Homo sapiens GN H2AFV PE 1 SV 3 |
| H2AZ\_HUMAN | NonConclusive | 298\*\*, 551\*\* | | Histone H2A Z OS Homo sapiens GN H2AFZ PE 1 SV 2 |
| H90B2\_HUMAN | NonConclusive | 147\*\*, 157\*\*, 183\*\*, 407\*\*, 432\*\*, 638\*\*, 706\*\* | | Putative heat shock protein HSP 90 beta 2 OS Homo sapiens GN HSP90AB2P PE 1 SV 2 |
| RA1L2\_HUMAN | NonConclusive | 61\*\*, 269\*\*, 741\*\*, 782\*\* | | Heterogeneous nuclear ribonucleoprotein A1 like 2 OS Homo sapiens GN HNRNPA1L2 PE 2 SV 2 |
| H90B4\_HUMAN | NonConclusive | 147\*\*, 236\*\*, 243\*\*, 432\*\* | | Putative heat shock protein HSP 90 beta 4 OS Homo sapiens GN HSP90AB4P PE 5 SV 1 |
| DESM\_HUMAN | NonConclusive | 342\*\*, 1410\*\* | | Desmin OS Homo sapiens GN DES PE 1 SV 3 |
| U5S1\_HUMAN | NonConclusive | 233\*\* | | 116 kDa U5 small nuclear ribonucleoprotein component OS Homo sapiens GN EFTUD2 PE 1 SV 1 |
| HS902\_HUMAN | NonConclusive | 147\*\*, 183\*\*, 236\*\*, 598\*\*, 638\*\* | | Putative heat shock protein HSP 90 alpha A2 OS Homo sapiens GN HSP90AA2 PE 1 SV 2 |
| LDH6A\_HUMAN | NonConclusive | 180\*\*, 425\*\* | | L lactate dehydrogenase A like 6A OS Homo sapiens GN LDHAL6A PE 2 SV 1 |
| PGAM4\_HUMAN | NonConclusive | 179\*\*, 1022\*\* | | Probable phosphoglycerate mutase 4 OS Homo sapiens GN PGAM4 PE 1 SV 1 |
| TBB3\_HUMAN | NonConclusive | 640\*\*, 906\*\*, 963\*\*, 1034\*\* | | Tubulin beta 3 chain OS Homo sapiens GN TUBB3 PE 1 SV 2 |
| TBB6\_HUMAN | NonConclusive | 906\*\*, 995\*\* | | Tubulin beta 6 chain OS Homo sapiens GN TUBB6 PE 1 SV 1 |
| PCBP3\_HUMAN | NonConclusive | 173\*\*, 526\*\* | | Poly rC binding protein 3 OS Homo sapiens GN PCBP3 PE 1 SV 2 |
| PCBP3\_HUMAN | NonConclusive | 173\*\*, 526\*\* | | Isoform 2 of Poly rC binding protein 3 OS Homo sapiens GN PCBP3 |
| PCBP3\_HUMAN | NonConclusive | 173\*\*, 526\*\* | | Isoform 4 of Poly rC binding protein 3 OS Homo sapiens GN PCBP3 |
| PCBP3\_HUMAN | NonConclusive | 173\*\*, 526\*\* | | Isoform 5 of Poly rC binding protein 3 OS Homo sapiens GN PCBP3 |
| PGAM2\_HUMAN | NonConclusive | 179\*\*, 1022\*\*, 1194\*\* | | Phosphoglycerate mutase 2 OS Homo sapiens GN PGAM2 PE 1 SV 3 |
| KPYM\_HUMAN | NonConclusive | 136\*\*, 311\*\*, 504\*\*, 725\*\*, 922\*\*, 1012\*\* | | Isoform M1 of Pyruvate kinase isozymes M1 M2 OS Homo sapiens GN PKM2 |
| IF4A2\_HUMAN | NonConclusive | 93\*\*, 349\*\*, 359\*\*, 753\*\* | | Eukaryotic initiation factor 4A II OS Homo sapiens GN EIF4A2 PE 1 SV 2 |
| IF4A2\_HUMAN | NonConclusive | 93\*\*, 349\*\*, 359\*\*, 753\*\* | | Isoform 2 of Eukaryotic initiation factor 4A II OS Homo sapiens GN EIF4A2 |
| HNRH2\_HUMAN | NonConclusive | 185\*\*, 272\*\*, 880\*\* | | Heterogeneous nuclear ribonucleoprotein H2 OS Homo sapiens GN HNRNPH2 PE 1 SV 1 |
| RLA0L\_HUMAN | NonConclusive | 52\*\*, 553\*\*, 735\*\*, 960\*\*, 976\*\* | | 60S acidic ribosomal protein P0 like OS Homo sapiens GN RPLP0P6 PE 5 SV 1 |
| SET\_HUMAN | NonConclusive | 211\*\*, 378\*\* | | Protein SET OS Homo sapiens GN SET PE 1 SV 3 |
| ENOG\_HUMAN | NonConclusive | 870\*\* | | Gamma enolase OS Homo sapiens GN ENO2 PE 1 SV 3 |
| HNRCL\_HUMAN | NonConclusive | 415\*\*, 692\*\*, 835\*\* | | Heterogeneous nuclear ribonucleoprotein C like 1 OS Homo sapiens GN HNRNPCL1 PE 1 SV 1 |
| HNRPC\_HUMAN | NonConclusive | 692\*\*, 835\*\* | | Isoform 3 of Heterogeneous nuclear ribonucleoproteins C1 C2 OS Homo sapiens GN HNRNPC |
| HS905\_HUMAN | NonConclusive | 255\*\*, 986\*\* | | Putative heat shock protein HSP 90 alpha A5 OS Homo sapiens GN HSP90AA5P PE 1 SV 1 |
| ALBU\_HUMAN | NonConclusive | 133\*\*, 153\*\*, 205\*\*, 511\*\* | | Isoform 2 of Serum albumin OS Homo sapiens GN ALB |
| PCBP1\_HUMAN | NonConclusive | 173\*\* | | Poly rC binding protein 1 OS Homo sapiens GN PCBP1 PE 1 SV 2 |
| ADT1\_HUMAN | NonConclusive | 118\*\*, 528\*\*, 980\*\* | | ADP ATP translocase 1 OS Homo sapiens GN SLC25A4 PE 1 SV 4 |
| ADT3\_HUMAN | NonConclusive | 118\*\*, 528\*\*, 864\*\*, 980\*\* | | ADP ATP translocase 3 OS Homo sapiens GN SLC25A6 PE 1 SV 4 |
| ADT4\_HUMAN | NonConclusive | 118\*\*, 980\*\* | | ADP ATP translocase 4 OS Homo sapiens GN SLC25A31 PE 1 SV 1 |
| PCBP3\_HUMAN | NonConclusive | 173\*\*, 526\*\* | | Isoform 3 of Poly rC binding protein 3 OS Homo sapiens GN PCBP3 |
| PLSL\_HUMAN | NonConclusive | 21\*\*, 1543\*\* | | Plastin 2 OS Homo sapiens GN LCP1 PE 1 SV 6 |
| DDX5\_HUMAN | NonConclusive | 575\*\* | | Probable ATP dependent RNA helicase DDX5 OS Homo sapiens GN DDX5 PE 1 SV 1 |
| DDX17\_HUMAN | NonConclusive | 575\*\* | | Isoform 3 of Probable ATP dependent RNA helicase DDX17 OS Homo sapiens GN DDX17 |
| GBF1\_HUMAN | NonConclusive | 1302\*\* | | Golgi specific brefeldin A resistance guanine nucleotide exchange factor 1 OS Homo sapiens GN GBF1 P |
| TBAL3\_HUMAN | NonConclusive | 11\*\* | | Tubulin alpha chain like 3 OS Homo sapiens GN TUBAL3 PE 1 SV 2 |
| TBAL3\_HUMAN | NonConclusive | 11\*\* | | Isoform 2 of Tubulin alpha chain like 3 OS Homo sapiens GN TUBAL3 |
| PGK2\_HUMAN | NonConclusive | 53\*\* | | Phosphoglycerate kinase 2 OS Homo sapiens GN PGK2 PE 1 SV 3 |
| DHX9\_HUMAN | NonConclusive | 1154\*\* | | Isoform 2 of ATP dependent RNA helicase A OS Homo sapiens GN DHX9 |
| PLSI\_HUMAN | NonConclusive | 1543\*\* | | Plastin 1 OS Homo sapiens GN PLS1 PE 1 SV 2 |
| HNRDL\_HUMAN | NonConclusive | 1629\*\* | | Heterogeneous nuclear ribonucleoprotein D like OS Homo sapiens GN HNRPDL PE 1 SV 3 |
| HNRDL\_HUMAN | NonConclusive | 1629\*\* | | Isoform 2 of Heterogeneous nuclear ribonucleoprotein D like OS Homo sapiens GN HNRPDL |
| PAL4A\_HUMAN | NonConclusive | 742\*\* | | Peptidyl prolyl cis trans isomerase A like 4A B C OS Homo sapiens GN PPIAL4A PE 1 SV 1 |
| HNRDL\_HUMAN | NonConclusive | 1629\*\* | | Isoform 3 of Heterogeneous nuclear ribonucleoprotein D like OS Homo sapiens GN HNRPDL |
| SUMO3\_HUMAN | Filtered |  | | Small ubiquitin related modifier 3 OS Homo sapiens GN SUMO3 PE 1 SV 2 |
| SUMO2\_HUMAN | Filtered |  | | Small ubiquitin related modifier 2 OS Homo sapiens GN SUMO2 PE 1 SV 3 |
| SUMO2\_HUMAN | Filtered |  | | Isoform 2 of Small ubiquitin related modifier 2 OS Homo sapiens GN SUMO2 |
| SUMO4\_HUMAN | Filtered |  | | Small ubiquitin related modifier 4 OS Homo sapiens GN SUMO4 PE 1 SV 2 |
| RL12\_HUMAN | Filtered |  | | 60S ribosomal protein L12 OS Homo sapiens GN RPL12 PE 1 SV 1 |
| RL12\_HUMAN | Filtered |  | | Isoform 2 of 60S ribosomal protein L12 OS Homo sapiens GN RPL12 |
| RS20\_HUMAN | Filtered |  | | 40S ribosomal protein S20 OS Homo sapiens GN RPS20 PE 1 SV 1 |
| KPYR\_HUMAN | Filtered |  | | Pyruvate kinase isozymes R L OS Homo sapiens GN PKLR PE 1 SV 2 |
| KPYR\_HUMAN | Filtered |  | | Isoform L type of Pyruvate kinase isozymes R L OS Homo sapiens GN PKLR |
| RS5\_HUMAN | Filtered |  | | 40S ribosomal protein S5 OS Homo sapiens GN RPS5 PE 1 SV 4 |
| RS3\_HUMAN | Filtered |  | | 40S ribosomal protein S3 OS Homo sapiens GN RPS3 PE 1 SV 2 |
| MDHC\_HUMAN | Filtered |  | | Malate dehydrogenase cytoplasmic OS Homo sapiens GN MDH1 PE 1 SV 4 |
| RS18\_HUMAN | Filtered |  | | 40S ribosomal protein S18 OS Homo sapiens GN RPS18 PE 1 SV 3 |
| ILF2\_HUMAN | Filtered |  | | Interleukin enhancer binding factor 2 OS Homo sapiens GN ILF2 PE 1 SV 2 |
| MARCS\_HUMAN | Filtered |  | | Myristoylated alanine rich C kinase substrate OS Homo sapiens GN MARCKS PE 1 SV 4 |
| HGB1A\_HUMAN | Filtered |  | | Putative high mobility group protein B1 like 1 OS Homo sapiens GN HMGB1P1 PE 5 SV 1 |
| GBLP\_HUMAN | Filtered |  | | Guanine nucleotide binding protein subunit beta 2 like 1 OS Homo sapiens GN GNB2L1 PE 1 SV 3 |
| 4F2\_HUMAN | Filtered |  | | 4F2 cell surface antigen heavy chain OS Homo sapiens GN SLC3A2 PE 1 SV 3 |
| 4F2\_HUMAN | Filtered |  | | Isoform 2 of 4F2 cell surface antigen heavy chain OS Homo sapiens GN SLC3A2 |
| 4F2\_HUMAN | Filtered |  | | Isoform 3 of 4F2 cell surface antigen heavy chain OS Homo sapiens GN SLC3A2 |
| 4F2\_HUMAN | Filtered |  | | Isoform 4 of 4F2 cell surface antigen heavy chain OS Homo sapiens GN SLC3A2 |
| CYC\_HUMAN | Filtered |  | | Cytochrome c OS Homo sapiens GN CYCS PE 1 SV 2 |
| PHB2\_HUMAN | Filtered |  | | Prohibitin 2 OS Homo sapiens GN PHB2 PE 1 SV 2 |
| ALDR\_HUMAN | Filtered |  | | Aldose reductase OS Homo sapiens GN AKR1B1 PE 1 SV 3 |
| TERA\_HUMAN | Filtered |  | | Transitional endoplasmic reticulum ATPase OS Homo sapiens GN VCP PE 1 SV 4 |
| UB2L3\_HUMAN | Filtered |  | | Ubiquitin conjugating enzyme E2 L3 OS Homo sapiens GN UBE2L3 PE 1 SV 1 |
| HS105\_HUMAN | Filtered |  | | Heat shock protein 105 kDa OS Homo sapiens GN HSPH1 PE 1 SV 1 |
| HS105\_HUMAN | Filtered |  | | Isoform Beta of Heat shock protein 105 kDa OS Homo sapiens GN HSPH1 |
| FUBP2\_HUMAN | Filtered |  | | Far upstream element binding protein 2 OS Homo sapiens GN KHSRP PE 1 SV 4 |
| FUBP2\_HUMAN | Filtered |  | | Isoform 2 of Far upstream element binding protein 2 OS Homo sapiens GN KHSRP |
| RS4X\_HUMAN | Filtered |  | | 40S ribosomal protein S4 X isoform OS Homo sapiens GN RPS4X PE 1 SV 2 |
| PUR9\_HUMAN | Filtered |  | | Bifunctional purine biosynthesis protein PURH OS Homo sapiens GN ATIC PE 1 SV 3 |
| SLMO1\_HUMAN | Filtered |  | | Protein slowmo homolog 1 OS Homo sapiens GN SLMO1 PE 2 SV 1 |
| RUVB1\_HUMAN | Filtered |  | | RuvB like 1 OS Homo sapiens GN RUVBL1 PE 1 SV 1 |
| PUF60\_HUMAN | Filtered |  | | Poly U binding splicing factor PUF60 OS Homo sapiens GN PUF60 PE 1 SV 1 |
| PUF60\_HUMAN | Filtered |  | | Isoform 2 of Poly U binding splicing factor PUF60 OS Homo sapiens GN PUF60 |
| PUF60\_HUMAN | Filtered |  | | Isoform 3 of Poly U binding splicing factor PUF60 OS Homo sapiens GN PUF60 |
| PUF60\_HUMAN | Filtered |  | | Isoform 4 of Poly U binding splicing factor PUF60 OS Homo sapiens GN PUF60 |
| PUF60\_HUMAN | Filtered |  | | Isoform 5 of Poly U binding splicing factor PUF60 OS Homo sapiens GN PUF60 |
| PUF60\_HUMAN | Filtered |  | | Isoform 6 of Poly U binding splicing factor PUF60 OS Homo sapiens GN PUF60 |
| VDAC1\_HUMAN | Filtered |  | | Voltage dependent anion selective channel protein 1 OS Homo sapiens GN VDAC1 PE 1 SV 2 |
| FUS\_HUMAN | Filtered |  | | RNA binding protein FUS OS Homo sapiens GN FUS PE 1 SV 1 |
| FUS\_HUMAN | Filtered |  | | Isoform Short of RNA binding protein FUS OS Homo sapiens GN FUS |
| PSA4\_HUMAN | Filtered |  | | Proteasome subunit alpha type 4 OS Homo sapiens GN PSMA4 PE 1 SV 1 |
| PSMD6\_HUMAN | Filtered |  | | 26S proteasome non ATPase regulatory subunit 6 OS Homo sapiens GN PSMD6 PE 1 SV 1 |
| CLH1\_HUMAN | Filtered |  | | Clathrin heavy chain 1 OS Homo sapiens GN CLTC PE 1 SV 5 |
| CLH1\_HUMAN | Filtered |  | | Isoform 2 of Clathrin heavy chain 1 OS Homo sapiens GN CLTC |
| CI141\_HUMAN | Filtered |  | | Putative uncharacterized protein C9orf141 OS Homo sapiens GN C9orf141 PE 2 SV 1 |
| CLIC1\_HUMAN | Filtered |  | | Chloride intracellular channel protein 1 OS Homo sapiens GN CLIC1 PE 1 SV 4 |
| BMP10\_HUMAN | Filtered |  | | Bone morphogenetic protein 10 OS Homo sapiens GN BMP10 PE 2 SV 1 |
| PRDX3\_HUMAN | Filtered |  | | Thioredoxin dependent peroxide reductase mitochondrial OS Homo sapiens GN PRDX3 PE 1 SV 3 |
| AT1A1\_HUMAN | Filtered |  | | Sodium potassium transporting ATPase subunit alpha 1 OS Homo sapiens GN ATP1A1 PE 1 SV 1 |
| AT1A1\_HUMAN | Filtered |  | | Isoform Short of Sodium potassium transporting ATPase subunit alpha 1 OS Homo sapiens GN ATP1A1 |
| AT1A2\_HUMAN | Filtered |  | | Sodium potassium transporting ATPase subunit alpha 2 OS Homo sapiens GN ATP1A2 PE 1 SV 1 |
| AT1A3\_HUMAN | Filtered |  | | Sodium potassium transporting ATPase subunit alpha 3 OS Homo sapiens GN ATP1A3 PE 1 SV 3 |
| AT1A4\_HUMAN | Filtered |  | | Sodium potassium transporting ATPase subunit alpha 4 OS Homo sapiens GN ATP1A4 PE 1 SV 3 |
| RS3A\_HUMAN | Filtered |  | | 40S ribosomal protein S3a OS Homo sapiens GN RPS3A PE 1 SV 2 |
| MAN1\_HUMAN | Filtered |  | | Inner nuclear membrane protein Man1 OS Homo sapiens GN LEMD3 PE 1 SV 2 |
| ACTN1\_HUMAN | Filtered |  | | Alpha actinin 1 OS Homo sapiens GN ACTN1 PE 1 SV 2 |
| ACTN1\_HUMAN | Filtered |  | | Isoform 2 of Alpha actinin 1 OS Homo sapiens GN ACTN1 |
| ACTN4\_HUMAN | Filtered |  | | Alpha actinin 4 OS Homo sapiens GN ACTN4 PE 1 SV 2 |
| GSHR\_HUMAN | Filtered |  | | Glutathione reductase mitochondrial OS Homo sapiens GN GSR PE 1 SV 2 |
| GSHR\_HUMAN | Filtered |  | | Isoform Cytoplasmic of Glutathione reductase mitochondrial OS Homo sapiens GN GSR |
| SYYC\_HUMAN | Filtered |  | | Tyrosine tRNA ligase cytoplasmic OS Homo sapiens GN YARS PE 1 SV 4 |
| LA\_HUMAN | Filtered |  | | Lupus La protein OS Homo sapiens GN SSB PE 1 SV 2 |
| IF4B\_HUMAN | Filtered |  | | Eukaryotic translation initiation factor 4B OS Homo sapiens GN EIF4B PE 1 SV 2 |
| CBPC6\_HUMAN | Filtered |  | | Cytosolic carboxypeptidase 6 OS Homo sapiens GN AGBL4 PE 2 SV 3 |
| CBPC6\_HUMAN | Filtered |  | | Isoform 2 of Cytosolic carboxypeptidase 6 OS Homo sapiens GN AGBL4 |
| RBMX\_HUMAN | Filtered |  | | RNA binding motif protein X chromosome OS Homo sapiens GN RBMX PE 1 SV 3 |
| AKD1\_HUMAN | Filtered |  | | Adenylate kinase domain containing protein 1 OS Homo sapiens GN AKD1 PE 1 SV 2 |
| AKD1\_HUMAN | Filtered |  | | Isoform 5 of Adenylate kinase domain containing protein 1 OS Homo sapiens GN AKD1 |
| AKD1\_HUMAN | Filtered |  | | Isoform 6 of Adenylate kinase domain containing protein 1 OS Homo sapiens GN AKD1 |
| IMA2\_HUMAN | Filtered |  | | Importin subunit alpha 2 OS Homo sapiens GN KPNA2 PE 1 SV 1 |
| SRGP2\_HUMAN | Filtered |  | | SLIT ROBO Rho GTPase activating protein 3 OS Homo sapiens GN SRGAP3 PE 1 SV 3 |
| SRGP2\_HUMAN | Filtered |  | | Isoform 2 of SLIT ROBO Rho GTPase activating protein 3 OS Homo sapiens GN SRGAP3 |
| SRGP2\_HUMAN | Filtered |  | | Isoform 3 of SLIT ROBO Rho GTPase activating protein 3 OS Homo sapiens GN SRGAP3 |
| PAP1L\_HUMAN | Filtered |  | | Polyadenylate binding protein 1 like OS Homo sapiens GN PABPC1L PE 2 SV 1 |
| RAB1A\_HUMAN | Filtered |  | | Ras related protein Rab 1A OS Homo sapiens GN RAB1A PE 1 SV 3 |
| RAB1B\_HUMAN | Filtered |  | | Ras related protein Rab 1B OS Homo sapiens GN RAB1B PE 1 SV 1 |
| C144B\_HUMAN | Filtered |  | | Coiled coil domain containing protein 144B OS Homo sapiens GN CCDC144B PE 2 SV 1 |
| IP3KB\_HUMAN | Filtered |  | | Inositol trisphosphate 3 kinase B OS Homo sapiens GN ITPKB PE 1 SV 5 |
| IP3KB\_HUMAN | Filtered |  | | Isoform 2 of Inositol trisphosphate 3 kinase B OS Homo sapiens GN ITPKB |
| YR010\_HUMAN | Filtered |  | | Putative uncharacterized protein ENSP00000344348 OS Homo sapiens PE 2 SV 2 |
| C1TC\_HUMAN | Filtered |  | | C 1 tetrahydrofolate synthase cytoplasmic OS Homo sapiens GN MTHFD1 PE 1 SV 3 |
| LRC41\_HUMAN | Filtered |  | | Isoform 3 of Leucine rich repeat containing protein 41 OS Homo sapiens GN LRRC41 |
| DDX3X\_HUMAN | Filtered |  | | ATP dependent RNA helicase DDX3X OS Homo sapiens GN DDX3X PE 1 SV 3 |
| RPN1\_HUMAN | Filtered |  | | Dolichyl diphosphooligosaccharide protein glycosyltransferase subunit 1 OS Homo sapiens GN RPN1 PE |
| PAIRB\_HUMAN | Filtered |  | | Plasminogen activator inhibitor 1 RNA binding protein OS Homo sapiens GN SERBP1 PE 1 SV 2 |
| PAIRB\_HUMAN | Filtered |  | | Isoform 2 of Plasminogen activator inhibitor 1 RNA binding protein OS Homo sapiens GN SERBP1 |
| PAIRB\_HUMAN | Filtered |  | | Isoform 3 of Plasminogen activator inhibitor 1 RNA binding protein OS Homo sapiens GN SERBP1 |
| PAIRB\_HUMAN | Filtered |  | | Isoform 4 of Plasminogen activator inhibitor 1 RNA binding protein OS Homo sapiens GN SERBP1 |
| K2C1B\_HUMAN | Filtered |  | | Keratin type II cytoskeletal 1b OS Homo sapiens GN KRT77 PE 1 SV 3 |
| SRSF3\_HUMAN | Filtered |  | | Serine arginine rich splicing factor 3 OS Homo sapiens GN SRSF3 PE 1 SV 1 |
| SRSF7\_HUMAN | Filtered |  | | Serine arginine rich splicing factor 7 OS Homo sapiens GN SRSF7 PE 1 SV 1 |
| SRSF7\_HUMAN | Filtered |  | | Isoform 2 of Serine arginine rich splicing factor 7 OS Homo sapiens GN SRSF7 |
| SRSF7\_HUMAN | Filtered |  | | Isoform 3 of Serine arginine rich splicing factor 7 OS Homo sapiens GN SRSF7 |
| K2C1\_HUMAN | Filtered |  | | Keratin type II cytoskeletal 1 OS Homo sapiens GN KRT1 PE 1 SV 6 |
| K22E\_HUMAN | Filtered |  | | Keratin type II cytoskeletal 2 epidermal OS Homo sapiens GN KRT2 PE 1 SV 2 |
| K22O\_HUMAN | Filtered |  | | Keratin type II cytoskeletal 2 oral OS Homo sapiens GN KRT76 PE 1 SV 2 |
| K2C6A\_HUMAN | Filtered |  | | Keratin type II cytoskeletal 6A OS Homo sapiens GN KRT6A PE 1 SV 3 |
| K2C6B\_HUMAN | Filtered |  | | Keratin type II cytoskeletal 6B OS Homo sapiens GN KRT6B PE 1 SV 5 |
| K2C6C\_HUMAN | Filtered |  | | Keratin type II cytoskeletal 6C OS Homo sapiens GN KRT6C PE 1 SV 3 |
| K2C75\_HUMAN | Filtered |  | | Keratin type II cytoskeletal 75 OS Homo sapiens GN KRT75 PE 1 SV 2 |
| RS2\_HUMAN | Filtered |  | | 40S ribosomal protein S2 OS Homo sapiens GN RPS2 PE 1 SV 2 |
| IF2B1\_HUMAN | Filtered |  | | Insulin like growth factor 2 mRNA binding protein 1 OS Homo sapiens GN IGF2BP1 PE 1 SV 2 |
| SODC\_HUMAN | Filtered |  | | Superoxide dismutase Cu Zn OS Homo sapiens GN SOD1 PE 1 SV 2 |
| PA2G4\_HUMAN | Filtered |  | | Proliferation associated protein 2G4 OS Homo sapiens GN PA2G4 PE 1 SV 3 |
| SFPQ\_HUMAN | Filtered |  | | Splicing factor proline and glutamine rich OS Homo sapiens GN SFPQ PE 1 SV 2 |
| SFPQ\_HUMAN | Filtered |  | | Isoform Short of Splicing factor proline and glutamine rich OS Homo sapiens GN SFPQ |
| HPRT\_HUMAN | Filtered |  | | Hypoxanthine guanine phosphoribosyltransferase OS Homo sapiens GN HPRT1 PE 1 SV 2 |
| GDIR1\_HUMAN | Filtered |  | | Rho GDP dissociation inhibitor 1 OS Homo sapiens GN ARHGDIA PE 1 SV 3 |
| FA64A\_HUMAN | Filtered |  | | Protein FAM64A OS Homo sapiens GN FAM64A PE 1 SV 1 |
| YBOX1\_HUMAN | Filtered |  | | Nuclease sensitive element binding protein 1 OS Homo sapiens GN YBX1 PE 1 SV 3 |
| HBB\_HUMAN | Filtered |  | | Hemoglobin subunit beta OS Homo sapiens GN HBB PE 1 SV 2 |
| IPYR\_HUMAN | Filtered |  | | Inorganic pyrophosphatase OS Homo sapiens GN PPA1 PE 1 SV 2 |
| ATPB\_HUMAN | Filtered |  | | ATP synthase subunit beta mitochondrial OS Homo sapiens GN ATP5B PE 1 SV 3 |
| RBMX\_HUMAN | Filtered |  | | Isoform 2 of RNA binding motif protein X chromosome OS Homo sapiens GN RBMX |
| NUAK1\_HUMAN | Filtered |  | | Isoform 2 of NUAK family SNF1 like kinase 1 OS Homo sapiens GN NUAK1 |
| MMRN1\_HUMAN | Filtered |  | | Multimerin 1 OS Homo sapiens GN MMRN1 PE 1 SV 3 |
| MMRN1\_HUMAN | Filtered |  | | Isoform 2 of Multimerin 1 OS Homo sapiens GN MMRN1 |
| K2C3\_HUMAN | Filtered |  | | Keratin type II cytoskeletal 3 OS Homo sapiens GN KRT3 PE 1 SV 3 |
| GDIA\_HUMAN | Filtered |  | | Rab GDP dissociation inhibitor alpha OS Homo sapiens GN GDI1 PE 1 SV 2 |
| GDIB\_HUMAN | Filtered |  | | Rab GDP dissociation inhibitor beta OS Homo sapiens GN GDI2 PE 1 SV 2 |
| K2C8\_HUMAN | Filtered |  | | Keratin type II cytoskeletal 8 OS Homo sapiens GN KRT8 PE 1 SV 7 |
| NP1L1\_HUMAN | Filtered |  | | Nucleosome assembly protein 1 like 1 OS Homo sapiens GN NAP1L1 PE 1 SV 1 |
| SRSF1\_HUMAN | Filtered |  | | Serine arginine rich splicing factor 1 OS Homo sapiens GN SRSF1 PE 1 SV 2 |
| TCPE\_HUMAN | Filtered |  | | T complex protein 1 subunit epsilon OS Homo sapiens GN CCT5 PE 1 SV 1 |
| INVO\_HUMAN | Filtered |  | | Involucrin OS Homo sapiens GN IVL PE 1 SV 2 |
| TEKT5\_HUMAN | Filtered |  | | Tektin 5 OS Homo sapiens GN TEKT5 PE 2 SV 1 |
| CACO2\_HUMAN | Filtered |  | | Calcium binding and coiled coil domain containing protein 2 OS Homo sapiens GN CALCOCO2 PE 1 SV 1 |
| FUBP1\_HUMAN | Filtered |  | | Far upstream element binding protein 1 OS Homo sapiens GN FUBP1 PE 1 SV 3 |
| FUBP1\_HUMAN | Filtered |  | | Isoform 2 of Far upstream element binding protein 1 OS Homo sapiens GN FUBP1 |
| SPICE\_HUMAN | Filtered |  | | Spindle and centriole associated protein 1 OS Homo sapiens GN SPICE1 PE 1 SV 1 |
| CC166\_HUMAN | Filtered |  | | Coiled coil domain containing protein 166 OS Homo sapiens GN CCDC166 PE 4 SV 1 |
| GRAP1\_HUMAN | Filtered |  | | GRIP1 associated protein 1 OS Homo sapiens GN GRIPAP1 PE 1 SV 1 |
| GRAP1\_HUMAN | Filtered |  | | Isoform 4 of GRIP1 associated protein 1 OS Homo sapiens GN GRIPAP1 |
| ASNS\_HUMAN | Filtered |  | | Asparagine synthetase glutamine hydrolyzing OS Homo sapiens GN ASNS PE 1 SV 4 |
| LIMA1\_HUMAN | Filtered |  | | LIM domain and actin binding protein 1 OS Homo sapiens GN LIMA1 PE 1 SV 1 |
| LIMA1\_HUMAN | Filtered |  | | Isoform 4 of LIM domain and actin binding protein 1 OS Homo sapiens GN LIMA1 |
| KLP6\_HUMAN | Filtered |  | | Kinesin like protein KLP6 OS Homo sapiens GN KLP6 PE 3 SV 2 |
| ARFP2\_HUMAN | Filtered |  | | Arfaptin 2 OS Homo sapiens GN ARFIP2 PE 1 SV 1 |
| PCBP4\_HUMAN | Filtered |  | | Poly rC binding protein 4 OS Homo sapiens GN PCBP4 PE 2 SV 1 |
| PCBP4\_HUMAN | Filtered |  | | Isoform 2 of Poly rC binding protein 4 OS Homo sapiens GN PCBP4 |
| RANG\_HUMAN | Filtered |  | | Ran specific GTPase activating protein OS Homo sapiens GN RANBP1 PE 1 SV 1 |
| TCPD\_HUMAN | Filtered |  | | T complex protein 1 subunit delta OS Homo sapiens GN CCT4 PE 1 SV 4 |
| EF1B\_HUMAN | Filtered |  | | Elongation factor 1 beta OS Homo sapiens GN EEF1B2 PE 1 SV 3 |
| PHB\_HUMAN | Filtered |  | | Prohibitin OS Homo sapiens GN PHB PE 1 SV 1 |
| F10A1\_HUMAN | Filtered |  | | Hsc70 interacting protein OS Homo sapiens GN ST13 PE 1 SV 2 |
| F10A5\_HUMAN | Filtered |  | | Putative protein FAM10A5 OS Homo sapiens GN ST13P5 PE 5 SV 1 |
| ST134\_HUMAN | Filtered |  | | Putative protein FAM10A4 OS Homo sapiens GN ST13P4 PE 5 SV 1 |
| CALR\_HUMAN | Filtered |  | | Calreticulin OS Homo sapiens GN CALR PE 1 SV 1 |
| FGF11\_HUMAN | Filtered |  | | Fibroblast growth factor 11 OS Homo sapiens GN FGF11 PE 2 SV 1 |
| 2AAA\_HUMAN | Filtered |  | | Serine threonine protein phosphatase 2A 65 kDa regulatory subunit A alpha isoform OS Homo sapiens GN |
| TAD2A\_HUMAN | Filtered |  | | Transcriptional adapter 2 alpha OS Homo sapiens GN TADA2A PE 1 SV 3 |
| TAD2A\_HUMAN | Filtered |  | | Isoform 2 of Transcriptional adapter 2 alpha OS Homo sapiens GN TADA2A |
| TKT\_HUMAN | Filtered |  | | Transketolase OS Homo sapiens GN TKT PE 1 SV 3 |
| XRCC6\_HUMAN | Filtered |  | | X ray repair cross complementing protein 6 OS Homo sapiens GN XRCC6 PE 1 SV 2 |
| NFIB\_HUMAN | Filtered |  | | Isoform 3 of Nuclear factor 1 B type OS Homo sapiens GN NFIB |
| PABP1\_HUMAN | Filtered |  | | Polyadenylate binding protein 1 OS Homo sapiens GN PABPC1 PE 1 SV 2 |
| PABP1\_HUMAN | Filtered |  | | Isoform 2 of Polyadenylate binding protein 1 OS Homo sapiens GN PABPC1 |
| PABP3\_HUMAN | Filtered |  | | Polyadenylate binding protein 3 OS Homo sapiens GN PABPC3 PE 1 SV 2 |
| TPM3\_HUMAN | Filtered |  | | Tropomyosin alpha 3 chain OS Homo sapiens GN TPM3 PE 1 SV 1 |
| SLMAP\_HUMAN | Filtered |  | | Sarcolemmal membrane associated protein OS Homo sapiens GN SLMAP PE 1 SV 1 |
| SLMAP\_HUMAN | Filtered |  | | Isoform 2 of Sarcolemmal membrane associated protein OS Homo sapiens GN SLMAP |
| SLMAP\_HUMAN | Filtered |  | | Isoform 3 of Sarcolemmal membrane associated protein OS Homo sapiens GN SLMAP |
| SLMAP\_HUMAN | Filtered |  | | Isoform 6 of Sarcolemmal membrane associated protein OS Homo sapiens GN SLMAP |
| SLMAP\_HUMAN | Filtered |  | | Isoform 7 of Sarcolemmal membrane associated protein OS Homo sapiens GN SLMAP |
| IPO5\_HUMAN | Filtered |  | | Importin 5 OS Homo sapiens GN IPO5 PE 1 SV 4 |
| IPO5\_HUMAN | Filtered |  | | Isoform 2 of Importin 5 OS Homo sapiens GN IPO5 |
| IPO5\_HUMAN | Filtered |  | | Isoform 3 of Importin 5 OS Homo sapiens GN IPO5 |
| SYAC\_HUMAN | Filtered |  | | Alanine tRNA ligase cytoplasmic OS Homo sapiens GN AARS PE 1 SV 2 |
| CPSF6\_HUMAN | Filtered |  | | Cleavage and polyadenylation specificity factor subunit 6 OS Homo sapiens GN CPSF6 PE 1 SV 2 |
| CPSF6\_HUMAN | Filtered |  | | Isoform 2 of Cleavage and polyadenylation specificity factor subunit 6 OS Homo sapiens GN CPSF6 |
| CPSF6\_HUMAN | Filtered |  | | Isoform 3 of Cleavage and polyadenylation specificity factor subunit 6 OS Homo sapiens GN CPSF6 |
| PUR2\_HUMAN | Filtered |  | | Trifunctional purine biosynthetic protein adenosine 3 OS Homo sapiens GN GART PE 1 SV 1 |
| PUR2\_HUMAN | Filtered |  | | Isoform Short of Trifunctional purine biosynthetic protein adenosine 3 OS Homo sapiens GN GART |
| SYG\_HUMAN | Filtered |  | | Glycine tRNA ligase OS Homo sapiens GN GARS PE 1 SV 3 |
| RS13\_HUMAN | Filtered |  | | 40S ribosomal protein S13 OS Homo sapiens GN RPS13 PE 1 SV 2 |
| RL11\_HUMAN | Filtered |  | | 60S ribosomal protein L11 OS Homo sapiens GN RPL11 PE 1 SV 2 |
| RL11\_HUMAN | Filtered |  | | Isoform 2 of 60S ribosomal protein L11 OS Homo sapiens GN RPL11 |
| VDAC2\_HUMAN | Filtered |  | | Voltage dependent anion selective channel protein 2 OS Homo sapiens GN VDAC2 PE 1 SV 2 |
| VDAC2\_HUMAN | Filtered |  | | Isoform 1 of Voltage dependent anion selective channel protein 2 OS Homo sapiens GN VDAC2 |
| VDAC2\_HUMAN | Filtered |  | | Isoform 2 of Voltage dependent anion selective channel protein 2 OS Homo sapiens GN VDAC2 |
| MSRA\_HUMAN | Filtered |  | | Mitochondrial peptide methionine sulfoxide reductase OS Homo sapiens GN MSRA PE 1 SV 1 |
| MSRA\_HUMAN | Filtered |  | | Isoform 3 of Mitochondrial peptide methionine sulfoxide reductase OS Homo sapiens GN MSRA |
| MSRA\_HUMAN | Filtered |  | | Isoform 5 of Mitochondrial peptide methionine sulfoxide reductase OS Homo sapiens GN MSRA |
| RRAGC\_HUMAN | Filtered |  | | Ras related GTP binding protein C OS Homo sapiens GN RRAGC PE 1 SV 1 |
| UCHL1\_HUMAN | Filtered |  | | Ubiquitin carboxyl terminal hydrolase isozyme L1 OS Homo sapiens GN UCHL1 PE 1 SV 2 |
| U2AF2\_HUMAN | Filtered |  | | Splicing factor U2AF 65 kDa subunit OS Homo sapiens GN U2AF2 PE 1 SV 4 |
| PDIA1\_HUMAN | Filtered |  | | Protein disulfide isomerase OS Homo sapiens GN P4HB PE 1 SV 3 |
| RBP56\_HUMAN | Filtered |  | | TATA binding protein associated factor 2N OS Homo sapiens GN TAF15 PE 1 SV 1 |
| RBP56\_HUMAN | Filtered |  | | Isoform Short of TATA binding protein associated factor 2N OS Homo sapiens GN TAF15 |
| RAB32\_HUMAN | Filtered |  | | Ras related protein Rab 32 OS Homo sapiens GN RAB32 PE 1 SV 3 |
| K1C10\_HUMAN | Filtered |  | | Keratin type I cytoskeletal 10 OS Homo sapiens GN KRT10 PE 1 SV 6 |
| MA6D1\_HUMAN | Filtered |  | | MAP6 domain containing protein 1 OS Homo sapiens GN MAP6D1 PE 1 SV 1 |
| OCTC\_HUMAN | Filtered |  | | Peroxisomal carnitine O octanoyltransferase OS Homo sapiens GN CROT PE 1 SV 2 |
| XIRP1\_HUMAN | Filtered |  | | Xin actin binding repeat containing protein 1 OS Homo sapiens GN XIRP1 PE 1 SV 1 |
| INHBA\_HUMAN | Filtered |  | | Inhibin beta A chain OS Homo sapiens GN INHBA PE 1 SV 2 |
| SIN3B\_HUMAN | Filtered |  | | Paired amphipathic helix protein Sin3b OS Homo sapiens GN SIN3B PE 1 SV 2 |
| SIN3B\_HUMAN | Filtered |  | | Isoform 2 of Paired amphipathic helix protein Sin3b OS Homo sapiens GN SIN3B |
| SIN3B\_HUMAN | Filtered |  | | Isoform 3 of Paired amphipathic helix protein Sin3b OS Homo sapiens GN SIN3B |
| PML\_HUMAN | Filtered |  | | Protein PML OS Homo sapiens GN PML PE 1 SV 3 |
| PML\_HUMAN | Filtered |  | | Isoform PML 2 of Protein PML OS Homo sapiens GN PML |
| PML\_HUMAN | Filtered |  | | Isoform PML 3 of Protein PML OS Homo sapiens GN PML |
| PML\_HUMAN | Filtered |  | | Isoform PML 4 of Protein PML OS Homo sapiens GN PML |
| PML\_HUMAN | Filtered |  | | Isoform PML 5 of Protein PML OS Homo sapiens GN PML |
| PML\_HUMAN | Filtered |  | | Isoform PML 6 of Protein PML OS Homo sapiens GN PML |
| PML\_HUMAN | Filtered |  | | Isoform PML 7 of Protein PML OS Homo sapiens GN PML |
| PML\_HUMAN | Filtered |  | | Isoform PML 8 of Protein PML OS Homo sapiens GN PML |
| PML\_HUMAN | Filtered |  | | Isoform PML 11 of Protein PML OS Homo sapiens GN PML |
| PML\_HUMAN | Filtered |  | | Isoform PML 12 of Protein PML OS Homo sapiens GN PML |
| PML\_HUMAN | Filtered |  | | Isoform PML 13 of Protein PML OS Homo sapiens GN PML |
| PML\_HUMAN | Filtered |  | | Isoform PML 14 of Protein PML OS Homo sapiens GN PML |
| GDPD3\_HUMAN | Filtered |  | | Glycerophosphodiester phosphodiesterase domain containing protein 3 OS Homo sapiens GN GDPD3 PE 2 SV |
| PHF19\_HUMAN | Filtered |  | | Isoform 2 of PHD finger protein 19 OS Homo sapiens GN PHF19 |

  


---

Protein P62805

| Name | H4\_HUMAN | | |
| Description | Histone H4 OS Homo sapiens GN HIST1H4A PE 1 SV 2 | | |
| Sequence | ```   1 MSGRGKGGKG LGKGGAKRHR KVLRDNIQGI TKPAIRRLAR RGGVKRISGL IYEETRGVLK VFLENVIRDA VTYTEHAKRK TVTAMDVVYA LKRQGRTLYG 101 FGG ``` | | |
| Evidence | Conclusive | | |
| Peptide list | 45, 317, 481, 698, 894 | | |
| Peptides | 45 | Confidence | Green |
| Runs | 1, 3, 4 |
| Relation | Unique |
| Proteins | H4\_HUMAN |
| Sequence | ``` DAVTYTEHAK ``` |
| Position | 69 |
| PTMs | none |
| 317 | Confidence | Green |
| Runs | 1, 2, 3, 4 |
| Relation | Unique |
| Proteins | H4\_HUMAN |
| Sequence | ``` DNIQGITKPAIR ``` |
| Position | 25 |
| PTMs | none |
| 481 | Confidence | Green |
| Runs | 1, 2, 3, 4 |
| Relation | Unique |
| Proteins | H4\_HUMAN |
| Sequence | ``` ISGLIYEETR ``` |
| Position | 47 |
| PTMs | none |
| 698 | Confidence | Green |
| Runs | 1, 2, 3, 4 |
| Relation | Unique |
| Proteins | H4\_HUMAN |
| Sequence | ``` VFLENVIR ``` |
| Position | 61 |
| PTMs | none |
| 894 | Confidence | Green |
| Runs | 1, 2, 3, 4 |
| Relation | Unique |
| Proteins | H4\_HUMAN |
| Sequence | ``` TVTAMDVVYALK ``` |
| Position | 81 |
| PTMs | none |

  

Protein P05387

| Name | RLA2\_HUMAN | | |
| Description | 60S acidic ribosomal protein P2 OS Homo sapiens GN RPLP2 PE 1 SV 1 | | |
| Sequence | ```   1 MRYVASYLLA ALGGNSSPSA KDIKKILDSV GIEADDDRLN KVISELNGKN IEDVIAQGIG KLASVPAGGA VAVSAAPGSA APAAGSAPAA AEEKKDEKKE 101 ESEESDDDMG FGLFD ``` | | |
| Evidence | Conclusive | | |
| Peptide list | 488, 770, 774 | | |
| Peptides | 488 | Confidence | Green |
| Runs | 1, 2, 3, 4 |
| Relation | Unique |
| Proteins | RLA2\_HUMAN |
| Sequence | ``` LASVPAGGAVAVSAAPGSAAPAAGSAPAAAEEK ``` |
| Position | 62 |
| PTMs | none |
| 770 | Confidence | Green |
| Runs | 1, 2, 3, 4 |
| Relation | Unique |
| Proteins | RLA2\_HUMAN |
| Sequence | ``` NIEDVIAQGIGK ``` |
| Position | 50 |
| PTMs | none |
| 774 | Confidence | Green |
| Runs | 1, 2, 3, 4 |
| Relation | Unique |
| Proteins | RLA2\_HUMAN |
| Sequence | ``` YVASYLLAALGGNSSPSAK ``` |
| Position | 3 |
| PTMs | Variant #1: Deamidation+N(13)  Variant #2: none |

  

Protein P63261

| Name | ACTG\_HUMAN | | |
| Description | Actin cytoplasmic 2 OS Homo sapiens GN ACTG1 PE 1 SV 1 | | |
| Sequence | ```   1 MEEEIAALVI DNGSGMCKAG FAGDDAPRAV FPSIVGRPRH QGVMVGMGQK DSYVGDEAQS KRGILTLKYP IEHGIVTNWD DMEKIWHHTF YNELRVAPEE 101 HPVLLTEAPL NPKANREKMT QIMFETFNTP AMYVAIQAVL SLYASGRTTG IVMDSGDGVT HTVPIYEGYA LPHAILRLDL AGRDLTDYLM KILTERGYSF 201 TTTAEREIVR DIKEKLCYVA LDFEQEMATA ASSSSLEKSY ELPDGQVITI GNERFRCPEA LFQPSFLGME SCGIHETTFN SIMKCDVDIR KDLYANTVLS 301 GGTTMYPGIA DRMQKEITAL APSTMKIKII APPERKYSVW IGGSILASLS TFQQMWISKQ EYDESGPSIV HRKCF ``` | | |
| Evidence | Conclusive | | |
| Peptide list | 51\*\*, 82\*\*, 121\*\*, 134\*\*, 140\*\*, 141\*\*, 216\*\*, 288\*\*, 431\*\*, 441, 614\*\*, 766\*\*, 857\*\*, 896\*\*, 1049\*\*, 1050\*\* | | |
| Peptides | 51\*\* | Confidence | Green |
| Runs | 1, 2, 3, 4 |
| Relation | NonDiscriminating |
| Proteins | ACTG\_HUMAN, ACTB\_HUMAN, ACTC\_HUMAN, ACTS\_HUMAN, ACTH\_HUMAN, ACTA\_HUMAN |
| Sequence | ``` DSYVGDEAQSK ``` |
| Position | 51 |
| PTMs | none |
| 82\*\* | Confidence | Green |
| Runs | 1, 2, 3, 4 |
| Relation | NonDiscriminating |
| Proteins | ACTG\_HUMAN, ACTB\_HUMAN, ACTBL\_HUMAN |
| Sequence | ``` CDVDIR ``` |
| Position | 285 |
| PTMs | Carbamidomethyl+C(1) |
| 121\*\* | Confidence | Green |
| Runs | 1, 2, 3, 4 |
| Relation | NonDiscriminating |
| Proteins | ACTG\_HUMAN, ACTB\_HUMAN, POTEE\_HUMAN, POTEF\_HUMAN, POTEJ\_HUMAN, POTEI\_HUMAN, ACTC\_HUMAN, ACTS\_HUMAN, ACTH\_HUMAN, ACTA\_HUMAN |
| Sequence | ``` AGFAGDDAPR ``` |
| Position | 19 |
| PTMs | none |
| 134\*\* | Confidence | Green |
| Runs | 1, 2, 4 |
| Relation | NonDiscriminating |
| Proteins | ACTG\_HUMAN, ACTB\_HUMAN, ACTC\_HUMAN, ACTS\_HUMAN, ACTH\_HUMAN, ACTA\_HUMAN, ACTBL\_HUMAN |
| Sequence | ``` HQGVMVGMGQK ``` |
| Position | 40 |
| PTMs | none |
| 140\*\* | Confidence | Green |
| Runs | 1, 2, 4 |
| Relation | NonDiscriminating |
| Proteins | ACTG\_HUMAN, ACTB\_HUMAN, POTEE\_HUMAN, POTEF\_HUMAN, POTEJ\_HUMAN, POTEI\_HUMAN, ACTC\_HUMAN, ACTS\_HUMAN, ACTBM\_HUMAN, ACTH\_HUMAN, ACTA\_HUMAN, ACTBL\_HUMAN |
| Sequence | ``` LDLAGR ``` |
| Position | 178 |
| PTMs | none |
| 141\*\* | Confidence | Green |
| Runs | 1, 2, 3, 4 |
| Relation | NonDiscriminating |
| Proteins | ACTG\_HUMAN, ACTB\_HUMAN, ACTC\_HUMAN, ACTS\_HUMAN, ACTH\_HUMAN, ACTA\_HUMAN, ACTBL\_HUMAN |
| Sequence | ``` IIAPPER ``` |
| Position | 329 |
| PTMs | none |
| 216\*\* | Confidence | Green |
| Runs | 1, 2, 3 |
| Relation | NonDiscriminating |
| Proteins | ACTG\_HUMAN, ACTB\_HUMAN, POTEE\_HUMAN, POTEF\_HUMAN, POTEJ\_HUMAN, POTEI\_HUMAN, ACTBM\_HUMAN |
| Sequence | ``` QEYDESGPSIVHR ``` |
| Position | 360 |
| PTMs | none |
| 288\*\* | Confidence | Green |
| Runs | 1, 2, 3 |
| Relation | NonDiscriminating |
| Proteins | ACTG\_HUMAN, ACTB\_HUMAN |
| Sequence | ``` GYSFTTTAER ``` |
| Position | 197 |
| PTMs | none |
| 431\*\* | Confidence | Green |
| Runs | 1, 2, 3, 4 |
| Relation | NonDiscriminating |
| Proteins | ACTG\_HUMAN, ACTB\_HUMAN, ACTC\_HUMAN, ACTS\_HUMAN, ACTH\_HUMAN, ACTA\_HUMAN |
| Sequence | ``` EITALAPSTMK ``` |
| Position | 316 |
| PTMs | none |
| 441 | Confidence | Green |
| Runs | 1, 3, 4 |
| Relation | Unique |
| Proteins | ACTG\_HUMAN |
| Sequence | ``` EEEIAALVIDNGSGMCK ``` |
| Position | 2 |
| PTMs | Variant #1: Carbamidomethyl+C(16)  Variant #2: Carbamidomethyl+C(16) Acetyl+N-TERM(1) |
| 614\*\* | Confidence | Green |
| Runs | 1, 2, 3, 4 |
| Relation | NonDiscriminating |
| Proteins | ACTG\_HUMAN, ACTB\_HUMAN |
| Sequence | ``` VAPEEHPVLLTEAPLNPK ``` |
| Position | 96 |
| PTMs | none |
| 766\*\* | Confidence | Green |
| Runs | 1, 2, 3, 4 |
| Relation | NonDiscriminating |
| Proteins | ACTG\_HUMAN, ACTB\_HUMAN, POTEE\_HUMAN, POTEF\_HUMAN, ACTC\_HUMAN, ACTS\_HUMAN, ACTBM\_HUMAN, ACTH\_HUMAN, ACTA\_HUMAN, ACTBL\_HUMAN |
| Sequence | ``` SYELPDGQVITIGNER ``` |
| Position | 239 |
| PTMs | none |
| 857\*\* | Confidence | Green |
| Runs | 1, 2, 4 |
| Relation | NonDiscriminating |
| Proteins | ACTG\_HUMAN, ACTB\_HUMAN |
| Sequence | ``` DLYANTVLSGGTTMYPGIADR ``` |
| Position | 292 |
| PTMs | none |
| 896\*\* | Confidence | Green |
| Runs | 1, 2, 3 |
| Relation | NonDiscriminating |
| Proteins | ACTG\_HUMAN, ACTB\_HUMAN |
| Sequence | ``` TTGIVMDSGDGVTHTVPIYEGYALPHAILR ``` |
| Position | 148 |
| PTMs | none |
| 1049\*\* | Confidence | Green |
| Runs | 1, 2, 4 |
| Relation | NonDiscriminating |
| Proteins | ACTG\_HUMAN, ACTB\_HUMAN |
| Sequence | ``` CPEALFQPSFLGMESCGIHETTFNSIMK ``` |
| Position | 257 |
| PTMs | Carbamidomethyl+C(1) Carbamidomethyl+C(16) |
| 1050\*\* | Confidence | Green |
| Runs | 1, 2, 4 |
| Relation | NonDiscriminating |
| Proteins | ACTG\_HUMAN, ACTB\_HUMAN, POTEE\_HUMAN |
| Sequence | ``` LCYVALDFEQEMATAASSSSLEK ``` |
| Position | 216 |
| PTMs | Carbamidomethyl+C(2) |

  

Protein P08107

| Name | HSP71\_HUMAN | | |
| Description | Heat shock 70 kDa protein 1A 1B OS Homo sapiens GN HSPA1A PE 1 SV 5 | | |
| Sequence | ```   1 MAKAAAIGID LGTTYSCVGV FQHGKVEIIA NDQGNRTTPS YVAFTDTERL IGDAAKNQVA LNPQNTVFDA KRLIGRKFGD PVVQSDMKHW PFQVINDGDK 101 PKVQVSYKGE TKAFYPEEIS SMVLTKMKEI AEAYLGYPVT NAVITVPAYF NDSQRQATKD AGVIAGLNVL RIINEPTAAA IAYGLDRTGK GERNVLIFDL 201 GGGTFDVSIL TIDDGIFEVK ATAGDTHLGG EDFDNRLVNH FVEEFKRKHK KDISQNKRAV RRLRTACERA KRTLSSSTQA SLEIDSLFEG IDFYTSITRA 301 RFEELCSDLF RSTLEPVEKA LRDAKLDKAQ IHDLVLVGGS TRIPKVQKLL QDFFNGRDLN KSINPDEAVA YGAAVQAAIL MGDKSENVQD LLLLDVAPLS 401 LGLETAGGVM TALIKRNSTI PTKQTQIFTT YSDNQPGVLI QVYEGERAMT KDNNLLGRFE LSGIPPAPRG VPQIEVTFDI DANGILNVTA TDKSTGKANK 501 ITITNDKGRL SKEEIERMVQ EAEKYKAEDE VQRERVSAKN ALESYAFNMK SAVEDEGLKG KISEADKKKV LDKCQEVISW LDANTLAEKD EFEHKRKELE 601 QVCNPIISGL YQGAGGPGPG GFGAQGPKGG SGSGPTIEEV D ``` | | |
| Evidence | Conclusive | | |
| Peptide list | 20\*\*, 59\*\*, 86, 103\*\*, 178\*\*, 232\*\*, 379, 502, 506\*\*, 519, 573, 773\*\*, 777, 781\*\*, 812\*\*, 876, 914, 939, 1064, 1352\*\*, 1434 | | |
| Peptides | 20\*\* | Confidence | Green |
| Runs | 1, 2, 3, 4 |
| Relation | NonDiscriminating |
| Proteins | HSP71\_HUMAN, HS71L\_HUMAN |
| Sequence | ``` YKAEDEVQR ``` |
| Position | 525 |
| PTMs | none |
| 59\*\* | Confidence | Green |
| Runs | 1, 2, 3, 4 |
| Relation | NonDiscriminating |
| Proteins | HSP71\_HUMAN, HSP7C\_HUMAN, HSP76\_HUMAN, HS71L\_HUMAN, HSP72\_HUMAN |
| Sequence | ``` ITITNDK ``` |
| Position | 501 |
| PTMs | none |
| 86 | Confidence | Green |
| Runs | 1, 2, 3, 4 |
| Relation | Unique |
| Proteins | HSP71\_HUMAN |
| Sequence | ``` SAVEDEGLK ``` |
| Position | 551 |
| PTMs | none |
| 103\*\* | Confidence | Green |
| Runs | 1, 2, 3, 4 |
| Relation | NonDiscriminating |
| Proteins | HSP71\_HUMAN, HSP76\_HUMAN, HSP77\_HUMAN |
| Sequence | ``` STLEPVEK ``` |
| Position | 312 |
| PTMs | none |
| 178\*\* | Confidence | Green |
| Runs | 1, 2, 3, 4 |
| Relation | NonDiscriminating |
| Proteins | HSP71\_HUMAN, HSP7C\_HUMAN, HSP7C\_HUMAN, HS71L\_HUMAN, HSP72\_HUMAN, GRP78\_HUMAN |
| Sequence | ``` VEIIANDQGNR ``` |
| Position | 26 |
| PTMs | none |
| 232\*\* | Confidence | Green |
| Runs | 1, 2, 3, 4 |
| Relation | NonDiscriminating |
| Proteins | HSP71\_HUMAN, HSP76\_HUMAN, HS71L\_HUMAN, HSP77\_HUMAN |
| Sequence | ``` ATAGDTHLGGEDFDNR ``` |
| Position | 221 |
| PTMs | none |
| 379 | Confidence | Green |
| Runs | 1, 2, 3, 4 |
| Relation | Unique |
| Proteins | HSP71\_HUMAN |
| Sequence | ``` FGDPVVQSDMK ``` |
| Position | 78 |
| PTMs | none |
| 502 | Confidence | Green |
| Runs | 1, 2, 3, 4 |
| Relation | Unique |
| Proteins | HSP71\_HUMAN |
| Sequence | ``` AQIHDLVLVGGSTR ``` |
| Position | 329 |
| PTMs | none |
| 506\*\* | Confidence | Green |
| Runs | 1, 2, 3, 4 |
| Relation | NonDiscriminating |
| Proteins | HSP71\_HUMAN, HSP7C\_HUMAN, HSP7C\_HUMAN, HSP76\_HUMAN, HS71L\_HUMAN, HSP77\_HUMAN, HSP72\_HUMAN |
| Sequence | ``` TTPSYVAFTDTER ``` |
| Position | 37 |
| PTMs | none |
| 519 | Confidence | Green |
| Runs | 1, 2, 3, 4 |
| Relation | Unique |
| Proteins | HSP71\_HUMAN |
| Sequence | ``` LVNHFVEEFK ``` |
| Position | 237 |
| PTMs | none |
| 573 | Confidence | Green |
| Runs | 1, 2, 3, 4 |
| Relation | Unique |
| Proteins | HSP71\_HUMAN |
| Sequence | ``` NQVALNPQNTVFDAK ``` |
| Position | 57 |
| PTMs | none |
| 773\*\* | Confidence | Green |
| Runs | 1, 2, 3, 4 |
| Relation | NonDiscriminating |
| Proteins | HSP71\_HUMAN, HSP76\_HUMAN |
| Sequence | ``` IINEPTAAAIAYGLDR ``` |
| Position | 172 |
| PTMs | none |
| 777 | Confidence | Green |
| Runs | 1, 2, 3, 4 |
| Relation | Unique |
| Proteins | HSP71\_HUMAN |
| Sequence | ``` LLQDFFNGR ``` |
| Position | 349 |
| PTMs | Variant #1: Deamidation+N(7)  Variant #2: none |
| 781\*\* | Confidence | Green |
| Runs | 1, 2, 3, 4 |
| Relation | NonDiscriminating |
| Proteins | HSP71\_HUMAN, HSP76\_HUMAN, HSP77\_HUMAN |
| Sequence | ``` FEELCSDLFR ``` |
| Position | 302 |
| PTMs | Carbamidomethyl+C(5) |
| 812\*\* | Confidence | Green |
| Runs | 1, 2, 3, 4 |
| Relation | NonDiscriminating |
| Proteins | HSP71\_HUMAN, HS71L\_HUMAN |
| Sequence | ``` DAGVIAGLNVLR ``` |
| Position | 160 |
| PTMs | none |
| 876 | Confidence | Green |
| Runs | 1, 2, 3 |
| Relation | Unique |
| Proteins | HSP71\_HUMAN |
| Sequence | ``` CQEVISWLDANTLAEKDEFEHK ``` |
| Position | 574 |
| PTMs | Carbamidomethyl+C(1) |
| 914 | Confidence | Green |
| Runs | 1, 2, 4 |
| Relation | Unique |
| Proteins | HSP71\_HUMAN |
| Sequence | ``` CQEVISWLDANTLAEK ``` |
| Position | 574 |
| PTMs | Carbamidomethyl+C(1) |
| 939 | Confidence | Green |
| Runs | 1, 2, 3, 4 |
| Relation | Unique |
| Proteins | HSP71\_HUMAN |
| Sequence | ``` ELEQVCNPIISGLYQGAGGPGPGGFGAQGPK ``` |
| Position | 598 |
| PTMs | Carbamidomethyl+C(6) |
| 1064 | Confidence | Green |
| Runs | 1, 2, 3, 4 |
| Relation | Unique |
| Proteins | HSP71\_HUMAN |
| Sequence | ``` EIAEAYLGYPVTNAVITVPAYFNDSQR ``` |
| Position | 129 |
| PTMs | Variant #1: none Variant #2: Deamidation+N(13) |
| 1352\*\* | Confidence | Green |
| Runs | 2, 3, 4 |
| Relation | NonDiscriminating |
| Proteins | HSP71\_HUMAN, HSP76\_HUMAN, HS71L\_HUMAN |
| Sequence | ``` AMTKDNNLLGR ``` |
| Position | 448 |
| PTMs | Variant #1: none Variant #2: Deamidation+N(6) |
| 1434 | Confidence | Green |
| Runs | 2, 3, 4 |
| Relation | Unique |
| Proteins | HSP71\_HUMAN |
| Sequence | ``` HWPFQVINDGDKPK ``` |
| Position | 89 |
| PTMs | none |

  

Protein P61604

| Name | CH10\_HUMAN | | |
| Description | 10 kDa heat shock protein mitochondrial OS Homo sapiens GN HSPE1 PE 1 SV 2 | | |
| Sequence | ```   1 MAGQAFRKFL PLFDRVLVER SAAETVTKGG IMLPEKSQGK VLQATVVAVG SGSKGKGGEI QPVSVKVGDK VLLPEYGGTK VVLDDKDYFL FRDGDILGKY 101 VD ``` | | |
| Evidence | Conclusive | | |
| Peptide list | 203, 371, 456, 821 | | |
| Peptides | 203 | Confidence | Green |
| Runs | 1, 2, 3, 4 |
| Relation | Unique |
| Proteins | CH10\_HUMAN |
| Sequence | ``` GGEIQPVSVK ``` |
| Position | 57 |
| PTMs | none |
| 371 | Confidence | Green |
| Runs | 1, 2, 3, 4 |
| Relation | Unique |
| Proteins | CH10\_HUMAN |
| Sequence | ``` VLQATVVAVGSGSK ``` |
| Position | 41 |
| PTMs | none |
| 456 | Confidence | Green |
| Runs | 1, 2, 3, 4 |
| Relation | Unique |
| Proteins | CH10\_HUMAN |
| Sequence | ``` VLLPEYGGTK ``` |
| Position | 71 |
| PTMs | none |
| 821 | Confidence | Green |
| Runs | 1, 2, 3, 4 |
| Relation | Unique |
| Proteins | CH10\_HUMAN |
| Sequence | ``` FLPLFDR ``` |
| Position | 9 |
| PTMs | none |

  

Protein P08238

| Name | HS90B\_HUMAN | | |
| Description | Heat shock protein HSP 90 beta OS Homo sapiens GN HSP90AB1 PE 1 SV 4 | | |
| Sequence | ```   1 MPEEVHHGEE EVETFAFQAE IAQLMSLIIN TFYSNKEIFL RELISNASDA LDKIRYESLT DPSKLDSGKE LKIDIIPNPQ ERTLTLVDTG IGMTKADLIN 101 NLGTIAKSGT KAFMEALQAG ADISMIGQFG VGFYSAYLVA EKVVVITKHN DDEQYAWESS AGGSFTVRAD HGEPIGRGTK VILHLKEDQT EYLEERRVKE 201 VVKKHSQFIG YPITLYLEKE REKEISDDEA EEEKGEKEEE DKDDEEKPKI EDVGSDEEDD SGKDKKKKTK KIKEKYIDQE ELNKTKPIWT RNPDDITQEE 301 YGEFYKSLTN DWEDHLAVKH FSVEGQLEFR ALLFIPRRAP FDLFENKKKK NNIKLYVRRV FIMDSCDELI PEYLNFIRGV VDSEDLPLNI SREMLQQSKI 401 LKVIRKNIVK KCLELFSELA EDKENYKKFY EAFSKNLKLG IHEDSTNRRR LSELLRYHTS QSGDEMTSLS EYVSRMKETQ KSIYYITGES KEQVANSAFV 501 ERVRKRGFEV VYMTEPIDEY CVQQLKEFDG KSLVSVTKEG LELPEDEEEK KKMEESKAKF ENLCKLMKEI LDKKVEKVTI SNRLVSSPCC IVTSTYGWTA 601 NMERIMKAQA LRDNSTMGYM MAKKHLEINP DHPIVETLRQ KAEADKNDKA VKDLVVLLFE TALLSSGFSL EDPQTHSNRI YRMIKLGLGI DEDEVAAEEP 701 NAAVPDEIPP LEGDEDASRM EEVD ``` | | |
| Evidence | Conclusive | | |
| Peptide list | 50, 99, 147\*\*, 157\*\*, 183\*\*, 236\*\*, 243\*\*, 249\*\*, 407\*\*, 432\*\*, 535\*\*, 554, 595, 619\*\*, 638\*\*, 689\*\*, 706\*\*, 748, 933\*\*, 1302\*\* | | |
| Peptides | 50 | Confidence | Green |
| Runs | 1, 2, 3, 4 |
| Relation | Unique |
| Proteins | HS90B\_HUMAN |
| Sequence | ``` LGIHEDSTNR ``` |
| Position | 439 |
| PTMs | none |
| 99 | Confidence | Green |
| Runs | 1, 2, 3, 4 |
| Relation | Unique |
| Proteins | HS90B\_HUMAN |
| Sequence | ``` VVVITK ``` |
| Position | 143 |
| PTMs | none |
| 147\*\* | Confidence | Green |
| Runs | 1, 2, 3, 4 |
| Relation | NonDiscriminating |
| Proteins | HS90B\_HUMAN, HS90A\_HUMAN, HS90A\_HUMAN, H90B3\_HUMAN, H90B2\_HUMAN, H90B4\_HUMAN, HS902\_HUMAN |
| Sequence | ``` YESLTDPSK ``` |
| Position | 56 |
| PTMs | none |
| 157\*\* | Confidence | Green |
| Runs | 1, 3, 4 |
| Relation | NonDiscriminating |
| Proteins | HS90B\_HUMAN, H90B3\_HUMAN, H90B2\_HUMAN |
| Sequence | ``` SLVSVTK ``` |
| Position | 532 |
| PTMs | none |
| 183\*\* | Confidence | Green |
| Runs | 1, 2, 4 |
| Relation | NonDiscriminating |
| Proteins | HS90B\_HUMAN, HS90A\_HUMAN, HS90A\_HUMAN, H90B2\_HUMAN, HS902\_HUMAN |
| Sequence | ``` YIDQEELNK ``` |
| Position | 276 |
| PTMs | none |
| 236\*\* | Confidence | Green |
| Runs | 1, 2, 3, 4 |
| Relation | NonDiscriminating |
| Proteins | HS90B\_HUMAN, HS90A\_HUMAN, HS90A\_HUMAN, H90B3\_HUMAN, H90B4\_HUMAN, HS902\_HUMAN |
| Sequence | ``` VILHLK ``` |
| Position | 181 |
| PTMs | none |
| 243\*\* | Confidence | Green |
| Runs | 1, 2, 3, 4 |
| Relation | NonDiscriminating |
| Proteins | HS90B\_HUMAN, HS90A\_HUMAN, HS90A\_HUMAN, H90B3\_HUMAN, H90B4\_HUMAN |
| Sequence | ``` EDQTEYLEER ``` |
| Position | 187 |
| PTMs | none |
| 249\*\* | Confidence | Green |
| Runs | 1, 2, 3, 4 |
| Relation | NonDiscriminating |
| Proteins | HS90B\_HUMAN, H90B3\_HUMAN |
| Sequence | ``` EQVANSAFVER ``` |
| Position | 492 |
| PTMs | none |
| 407\*\* | Confidence | Green |
| Runs | 1, 2, 3 |
| Relation | NonDiscriminating |
| Proteins | HS90B\_HUMAN, H90B2\_HUMAN |
| Sequence | ``` SIYYITGESK ``` |
| Position | 482 |
| PTMs | none |
| 432\*\* | Confidence | Green |
| Runs | 1, 2, 3, 4 |
| Relation | NonDiscriminating |
| Proteins | HS90B\_HUMAN, H90B3\_HUMAN, H90B2\_HUMAN, H90B4\_HUMAN, ENPL\_HUMAN |
| Sequence | ``` ELISNASDALDK ``` |
| Position | 42 |
| PTMs | none |
| 535\*\* | Confidence | Green |
| Runs | 1, 2, 3 |
| Relation | NonDiscriminating |
| Proteins | HS90B\_HUMAN, H90B3\_HUMAN |
| Sequence | ``` IDIIPNPQER ``` |
| Position | 73 |
| PTMs | none |
| 554 | Confidence | Green |
| Runs | 1, 2, 3, 4 |
| Relation | Unique |
| Proteins | HS90B\_HUMAN |
| Sequence | ``` HLEINPDHPIVETLR ``` |
| Position | 625 |
| PTMs | none |
| 595 | Confidence | Green |
| Runs | 1, 2, 3, 4 |
| Relation | Unique |
| Proteins | HS90B\_HUMAN |
| Sequence | ``` YHTSQSGDEMTSLSEYVSR ``` |
| Position | 457 |
| PTMs | none |
| 619\*\* | Confidence | Green |
| Runs | 1, 3, 4 |
| Relation | NonDiscriminating |
| Proteins | HS90B\_HUMAN, HS90A\_HUMAN, HS90A\_HUMAN |
| Sequence | ``` SLTNDWEDHLAVK ``` |
| Position | 307 |
| PTMs | Variant #1: none Variant #2: Deamidation+N(4) |
| 638\*\* | Confidence | Green |
| Runs | 1, 2, 3, 4 |
| Relation | NonDiscriminating |
| Proteins | HS90B\_HUMAN, HS90A\_HUMAN, HS90A\_HUMAN, H90B2\_HUMAN, HS902\_HUMAN |
| Sequence | ``` ADLINNLGTIAK ``` |
| Position | 96 |
| PTMs | Variant #1: Deamidation+N(5)  Variant #2: none |
| 689\*\* | Confidence | Green |
| Runs | 1, 2, 3, 4 |
| Relation | NonDiscriminating |
| Proteins | HS90B\_HUMAN, HS90A\_HUMAN, HS90A\_HUMAN, H90B3\_HUMAN |
| Sequence | ``` GVVDSEDLPLNISR ``` |
| Position | 379 |
| PTMs | none |
| 706\*\* | Confidence | Green |
| Runs | 1, 2, 3, 4 |
| Relation | NonDiscriminating |
| Proteins | HS90B\_HUMAN, H90B2\_HUMAN |
| Sequence | ``` TLTLVDTGIGMTK ``` |
| Position | 83 |
| PTMs | none |
| 748 | Confidence | Green |
| Runs | 1, 2, 3 |
| Relation | Unique |
| Proteins | HS90B\_HUMAN |
| Sequence | ``` ALLFIPR ``` |
| Position | 331 |
| PTMs | none |
| 933\*\* | Confidence | Green |
| Runs | 1, 2, 3, 4 |
| Relation | NonDiscriminating |
| Proteins | HS90B\_HUMAN, H90B3\_HUMAN |
| Sequence | ``` HSQFIGYPITLYLEK ``` |
| Position | 205 |
| PTMs | none |
| 1302\*\* | Confidence | Green |
| Runs | 2, 3, 4 |
| Relation | NonDiscriminating |
| Proteins | HS90B\_HUMAN, HS90A\_HUMAN, HS90A\_HUMAN, GBF1\_HUMAN |
| Sequence | ``` LSELLR ``` |
| Position | 451 |
| PTMs | none |

  

Protein P07437

| Name | TBB5\_HUMAN | | |
| Description | Tubulin beta chain OS Homo sapiens GN TUBB PE 1 SV 2 | | |
| Sequence | ```   1 MREIVHIQAG QCGNQIGAKF WEVISDEHGI DPTGTYHGDS DLQLDRISVY YNEATGGKYV PRAILVDLEP GTMDSVRSGP FGQIFRPDNF VFGQSGAGNN 101 WAKGHYTEGA ELVDSVLDVV RKEAESCDCL QGFQLTHSLG GGTGSGMGTL LISKIREEYP DRIMNTFSVV PSPKVSDTVV EPYNATLSVH QLVENTDETY 201 CIDNEALYDI CFRTLKLTTP TYGDLNHLVS ATMSGVTTCL RFPGQLNADL RKLAVNMVPF PRLHFFMPGF APLTSRGSQQ YRALTVPELT QQVFDAKNMM 301 AACDPRHGRY LTVAAVFRGR MSMKEVDEQM LNVQNKNSSY FVEWIPNNVK TAVCDIPPRG LKMAVTFIGN STAIQELFKR ISEQFTAMFR RKAFLHWYTG 401 EGMDEMEFTE AESNMNDLVS EYQQYQDATA EEEEDFGEEA EEEA ``` | | |
| Evidence | Conclusive | | |
| Peptide list | 42\*\*, 337, 368\*\*, 596\*\*, 640\*\*, 796\*\*, 906\*\*, 941, 995\*\*, 1034\*\*, 1080\*\* | | |
| Peptides | 42\*\* | Confidence | Green |
| Runs | 1, 2, 3, 4 |
| Relation | NonDiscriminating |
| Proteins | TBB5\_HUMAN, TBB4B\_HUMAN, TBB2A\_HUMAN, TBB2B\_HUMAN, TBB8\_HUMAN, YI016\_HUMAN, TBB8B\_HUMAN, TBB1\_HUMAN |
| Sequence | ``` IREEYPDR ``` |
| Position | 155 |
| PTMs | none |
| 337 | Confidence | Green |
| Runs | 1, 2, 3, 4 |
| Relation | Unique |
| Proteins | TBB5\_HUMAN |
| Sequence | ``` ISVYYNEATGGK ``` |
| Position | 47 |
| PTMs | none |
| 368\*\* | Confidence | Green |
| Runs | 1, 2, 3, 4 |
| Relation | NonDiscriminating |
| Proteins | TBB5\_HUMAN, TBB4B\_HUMAN, TBB2A\_HUMAN, TBB2B\_HUMAN |
| Sequence | ``` EVDEQMLNVQNK ``` |
| Position | 325 |
| PTMs | none |
| 596\*\* | Confidence | Green |
| Runs | 1, 2, 4 |
| Relation | NonDiscriminating |
| Proteins | TBB5\_HUMAN, TBB4B\_HUMAN, TBB2A\_HUMAN, TBB2B\_HUMAN, TBB4A\_HUMAN |
| Sequence | ``` SGPFGQIFRPDNFVFGQSGAGNNWAK ``` |
| Position | 78 |
| PTMs | Variant #1: Deamidation+N(12) Deamidation+N(22)  Variant #2: none Variant #3: Deamidation+Q(17) |
| 640\*\* | Confidence | Green |
| Runs | 1, 2, 3, 4 |
| Relation | NonDiscriminating |
| Proteins | TBB5\_HUMAN, TBB4B\_HUMAN, TBB4A\_HUMAN, TBB3\_HUMAN |
| Sequence | ``` IMNTFSVVPSPK ``` |
| Position | 163 |
| PTMs | none |
| 796\*\* | Confidence | Green |
| Runs | 1, 2, 3, 4 |
| Relation | NonDiscriminating |
| Proteins | TBB5\_HUMAN, TBB4B\_HUMAN, TBB4A\_HUMAN |
| Sequence | ``` YLTVAAVFR ``` |
| Position | 310 |
| PTMs | none |
| 906\*\* | Confidence | Green |
| Runs | 1, 2, 3, 4 |
| Relation | NonDiscriminating |
| Proteins | TBB5\_HUMAN, TBB4B\_HUMAN, TBB2A\_HUMAN, TBB2B\_HUMAN, TBB4A\_HUMAN, TBB3\_HUMAN, TBB6\_HUMAN |
| Sequence | ``` NSSYFVEWIPNNVK ``` |
| Position | 337 |
| PTMs | none |
| 941 | Confidence | Green |
| Runs | 1, 2, 3, 4 |
| Relation | Unique |
| Proteins | TBB5\_HUMAN |
| Sequence | ``` ALTVPELTQQVFDAK ``` |
| Position | 283 |
| PTMs | Variant #1: none Variant #2: Deamidation+Q(9) |
| 995\*\* | Confidence | Green |
| Runs | 1, 2, 3, 4 |
| Relation | NonDiscriminating |
| Proteins | TBB5\_HUMAN, TBB4B\_HUMAN, TBB2A\_HUMAN, TBB2B\_HUMAN, TBB4A\_HUMAN, TBB8\_HUMAN, YI016\_HUMAN, TBB8B\_HUMAN, TBB6\_HUMAN |
| Sequence | ``` LHFFMPGFAPLTSR ``` |
| Position | 263 |
| PTMs | none |
| 1034\*\* | Confidence | Green |
| Runs | 1, 2, 3, 4 |
| Relation | NonDiscriminating |
| Proteins | TBB5\_HUMAN, TBB4B\_HUMAN, TBB2A\_HUMAN, TBB2B\_HUMAN, TBB3\_HUMAN |
| Sequence | ``` GHYTEGAELVDSVLDVVR ``` |
| Position | 104 |
| PTMs | none |
| 1080\*\* | Confidence | Green |
| Runs | 1, 2, 3, 4 |
| Relation | NonDiscriminating |
| Proteins | TBB5\_HUMAN, TBB4B\_HUMAN, TBB2A\_HUMAN, TBB2B\_HUMAN, TBB4A\_HUMAN |
| Sequence | ``` LTTPTYGDLNHLVSATMSGVTTCLR ``` |
| Position | 217 |
| PTMs | Variant #1: Carbamidomethyl+C(23)  Variant #2: Carbamidomethyl+C(23) Deamidation+N(10) |

  

Protein P68371

| Name | TBB4B\_HUMAN | | |
| Description | Tubulin beta 4B chain OS Homo sapiens GN TUBB4B PE 1 SV 1 | | |
| Sequence | ```   1 MREIVHLQAG QCGNQIGAKF WEVISDEHGI DPTGTYHGDS DLQLERINVY YNEATGGKYV PRAVLVDLEP GTMDSVRSGP FGQIFRPDNF VFGQSGAGNN 101 WAKGHYTEGA ELVDSVLDVV RKEAESCDCL QGFQLTHSLG GGTGSGMGTL LISKIREEYP DRIMNTFSVV PSPKVSDTVV EPYNATLSVH QLVENTDETY 201 CIDNEALYDI CFRTLKLTTP TYGDLNHLVS ATMSGVTTCL RFPGQLNADL RKLAVNMVPF PRLHFFMPGF APLTSRGSQQ YRALTVPELT QQMFDAKNMM 301 AACDPRHGRY LTVAAVFRGR MSMKEVDEQM LNVQNKNSSY FVEWIPNNVK TAVCDIPPRG LKMSATFIGN STAIQELFKR ISEQFTAMFR RKAFLHWYTG 401 EGMDEMEFTE AESNMNDLVS EYQQYQDATA EEEGEFEEEA EEEVA ``` | | |
| Evidence | Conclusive | | |
| Peptide list | 42\*\*, 368\*\*, 596\*\*, 640\*\*, 796\*\*, 906\*\*, 963\*\*, 995\*\*, 1034\*\*, 1080\*\*, 1304 | | |
| Peptides | 42\*\* | Confidence | Green |
| Runs | 1, 2, 3, 4 |
| Relation | NonDiscriminating |
| Proteins | TBB5\_HUMAN, TBB4B\_HUMAN, TBB2A\_HUMAN, TBB2B\_HUMAN, TBB8\_HUMAN, YI016\_HUMAN, TBB8B\_HUMAN, TBB1\_HUMAN |
| Sequence | ``` IREEYPDR ``` |
| Position | 155 |
| PTMs | none |
| 368\*\* | Confidence | Green |
| Runs | 1, 2, 3, 4 |
| Relation | NonDiscriminating |
| Proteins | TBB5\_HUMAN, TBB4B\_HUMAN, TBB2A\_HUMAN, TBB2B\_HUMAN |
| Sequence | ``` EVDEQMLNVQNK ``` |
| Position | 325 |
| PTMs | none |
| 596\*\* | Confidence | Green |
| Runs | 1, 2, 4 |
| Relation | NonDiscriminating |
| Proteins | TBB5\_HUMAN, TBB4B\_HUMAN, TBB2A\_HUMAN, TBB2B\_HUMAN, TBB4A\_HUMAN |
| Sequence | ``` SGPFGQIFRPDNFVFGQSGAGNNWAK ``` |
| Position | 78 |
| PTMs | Variant #1: Deamidation+N(12) Deamidation+N(22)  Variant #2: none Variant #3: Deamidation+Q(17) |
| 640\*\* | Confidence | Green |
| Runs | 1, 2, 3, 4 |
| Relation | NonDiscriminating |
| Proteins | TBB5\_HUMAN, TBB4B\_HUMAN, TBB4A\_HUMAN, TBB3\_HUMAN |
| Sequence | ``` IMNTFSVVPSPK ``` |
| Position | 163 |
| PTMs | none |
| 796\*\* | Confidence | Green |
| Runs | 1, 2, 3, 4 |
| Relation | NonDiscriminating |
| Proteins | TBB5\_HUMAN, TBB4B\_HUMAN, TBB4A\_HUMAN |
| Sequence | ``` YLTVAAVFR ``` |
| Position | 310 |
| PTMs | none |
| 906\*\* | Confidence | Green |
| Runs | 1, 2, 3, 4 |
| Relation | NonDiscriminating |
| Proteins | TBB5\_HUMAN, TBB4B\_HUMAN, TBB2A\_HUMAN, TBB2B\_HUMAN, TBB4A\_HUMAN, TBB3\_HUMAN, TBB6\_HUMAN |
| Sequence | ``` NSSYFVEWIPNNVK ``` |
| Position | 337 |
| PTMs | none |
| 963\*\* | Confidence | Green |
| Runs | 1, 2, 3, 4 |
| Relation | NonDiscriminating |
| Proteins | TBB4B\_HUMAN, TBB4A\_HUMAN, TBB3\_HUMAN |
| Sequence | ``` ALTVPELTQQMFDAK ``` |
| Position | 283 |
| PTMs | none |
| 995\*\* | Confidence | Green |
| Runs | 1, 2, 3, 4 |
| Relation | NonDiscriminating |
| Proteins | TBB5\_HUMAN, TBB4B\_HUMAN, TBB2A\_HUMAN, TBB2B\_HUMAN, TBB4A\_HUMAN, TBB8\_HUMAN, YI016\_HUMAN, TBB8B\_HUMAN, TBB6\_HUMAN |
| Sequence | ``` LHFFMPGFAPLTSR ``` |
| Position | 263 |
| PTMs | none |
| 1034\*\* | Confidence | Green |
| Runs | 1, 2, 3, 4 |
| Relation | NonDiscriminating |
| Proteins | TBB5\_HUMAN, TBB4B\_HUMAN, TBB2A\_HUMAN, TBB2B\_HUMAN, TBB3\_HUMAN |
| Sequence | ``` GHYTEGAELVDSVLDVVR ``` |
| Position | 104 |
| PTMs | none |
| 1080\*\* | Confidence | Green |
| Runs | 1, 2, 3, 4 |
| Relation | NonDiscriminating |
| Proteins | TBB5\_HUMAN, TBB4B\_HUMAN, TBB2A\_HUMAN, TBB2B\_HUMAN, TBB4A\_HUMAN |
| Sequence | ``` LTTPTYGDLNHLVSATMSGVTTCLR ``` |
| Position | 217 |
| PTMs | Variant #1: Carbamidomethyl+C(23)  Variant #2: Carbamidomethyl+C(23) Deamidation+N(10) |
| 1304 | Confidence | Green |
| Runs | 2, 3, 4 |
| Relation | Unique |
| Proteins | TBB4B\_HUMAN |
| Sequence | ``` INVYYNEATGGK ``` |
| Position | 47 |
| PTMs | none |

  

Protein P05386

| Name | RLA1\_HUMAN | | |
| Description | 60S acidic ribosomal protein P1 OS Homo sapiens GN RPLP1 PE 1 SV 1 | | |
| Sequence | ```   1 MASVSELACI YSALILHDDE VTVTEDKINA LIKAAGVNVE PFWPGLFAKA LANVNIGSLI CNVGAGGPAP AAGAAPAGGP APSTAAAPAE EKKVEAKKEE 101 SEESDDDMGF GLFD ``` | | |
| Evidence | Conclusive | | |
| Peptide list | 917, 1066 | | |
| Peptides | 917 | Confidence | Green |
| Runs | 1, 2, 3, 4 |
| Relation | Unique |
| Proteins | RLA1\_HUMAN |
| Sequence | ``` ALANVNIGSLICNVGAGGPAPAAGAAPAGGPAPSTAAAPAEEK ``` |
| Position | 50 |
| PTMs | Carbamidomethyl+C(12) |
| 1066 | Confidence | Green |
| Runs | 1, 3, 4 |
| Relation | Unique |
| Proteins | RLA1\_HUMAN |
| Sequence | ``` AAGVNVEPFWPGLFAK ``` |
| Position | 34 |
| PTMs | none |

  

Protein P11142

| Name | HSP7C\_HUMAN | | |
| Description | Heat shock cognate 71 kDa protein OS Homo sapiens GN HSPA8 PE 1 SV 1 | | |
| Sequence | ```   1 MSKGPAVGID LGTTYSCVGV FQHGKVEIIA NDQGNRTTPS YVAFTDTERL IGDAAKNQVA MNPTNTVFDA KRLIGRRFDD AVVQSDMKHW PFMVVNDAGR 101 PKVQVEYKGE TKSFYPEEVS SMVLTKMKEI AEAYLGKTVT NAVVTVPAYF NDSQRQATKD AGTIAGLNVL RIINEPTAAA IAYGLDKKVG AERNVLIFDL 201 GGGTFDVSIL TIEDGIFEVK STAGDTHLGG EDFDNRMVNH FIAEFKRKHK KDISENKRAV RRLRTACERA KRTLSSSTQA SIEIDSLYEG IDFYTSITRA 301 RFEELNADLF RGTLDPVEKA LRDAKLDKSQ IHDIVLVGGS TRIPKIQKLL QDFFNGKELN KSINPDEAVA YGAAVQAAIL SGDKSENVQD LLLLDVTPLS 401 LGIETAGGVM TVLIKRNTTI PTKQTQTFTT YSDNQPGVLI QVYEGERAMT KDNNLLGKFE LTGIPPAPRG VPQIEVTFDI DANGILNVSA VDKSTGKENK 501 ITITNDKGRL SKEDIERMVQ EAEKYKAEDE KQRDKVSSKN SLESYAFNMK ATVEDEKLQG KINDEDKQKI LDKCNEIINW LDKNQTAEKE EFEHQQKELE 601 KVCNPIITKL YQSAGGMPGG MPGGFPGGGA PPSGGASSGP TIEEVD ``` | | |
| Evidence | Conclusive | | |
| Peptide list | 19, 59\*\*, 68\*\*, 178\*\*, 321\*\*, 360\*\*, 457\*\*, 506\*\*, 543\*\*, 716\*\*, 720\*\*, 749\*\*, 794\*\*, 840\*\*, 853, 1042\*\* | | |
| Peptides | 19 | Confidence | Green |
| Runs | 1, 2, 3, 4 |
| Relation | Unique |
| Proteins | HSP7C\_HUMAN |
| Sequence | ``` NQTAEKEEFEHQQK ``` |
| Position | 584 |
| PTMs | none |
| 59\*\* | Confidence | Green |
| Runs | 1, 2, 3, 4 |
| Relation | NonDiscriminating |
| Proteins | HSP71\_HUMAN, HSP7C\_HUMAN, HSP76\_HUMAN, HS71L\_HUMAN, HSP72\_HUMAN |
| Sequence | ``` ITITNDK ``` |
| Position | 501 |
| PTMs | none |
| 68\*\* | Confidence | Green |
| Runs | 1, 3, 4 |
| Relation | NonDiscriminating |
| Proteins | HSP7C\_HUMAN, HSP7C\_HUMAN, HSP72\_HUMAN |
| Sequence | ``` VQVEYK ``` |
| Position | 103 |
| PTMs | none |
| 178\*\* | Confidence | Green |
| Runs | 1, 2, 3, 4 |
| Relation | NonDiscriminating |
| Proteins | HSP71\_HUMAN, HSP7C\_HUMAN, HSP7C\_HUMAN, HS71L\_HUMAN, HSP72\_HUMAN, GRP78\_HUMAN |
| Sequence | ``` VEIIANDQGNR ``` |
| Position | 26 |
| PTMs | none |
| 321\*\* | Confidence | Green |
| Runs | 1, 2, 4 |
| Relation | NonDiscriminating |
| Proteins | HSP7C\_HUMAN, HSP7C\_HUMAN |
| Sequence | ``` RFDDAVVQSDMK ``` |
| Position | 77 |
| PTMs | none |
| 360\*\* | Confidence | Green |
| Runs | 1, 2, 3, 4 |
| Relation | NonDiscriminating |
| Proteins | HSP7C\_HUMAN, HSP7C\_HUMAN |
| Sequence | ``` EIAEAYLGK ``` |
| Position | 129 |
| PTMs | none |
| 457\*\* | Confidence | Green |
| Runs | 1, 2, 3, 4 |
| Relation | NonDiscriminating |
| Proteins | HSP7C\_HUMAN, HSP7C\_HUMAN |
| Sequence | ``` SQIHDIVLVGGSTR ``` |
| Position | 329 |
| PTMs | none |
| 506\*\* | Confidence | Green |
| Runs | 1, 2, 3, 4 |
| Relation | NonDiscriminating |
| Proteins | HSP71\_HUMAN, HSP7C\_HUMAN, HSP7C\_HUMAN, HSP76\_HUMAN, HS71L\_HUMAN, HSP77\_HUMAN, HSP72\_HUMAN |
| Sequence | ``` TTPSYVAFTDTER ``` |
| Position | 37 |
| PTMs | none |
| 543\*\* | Confidence | Green |
| Runs | 1, 3, 4 |
| Relation | NonDiscriminating |
| Proteins | HSP7C\_HUMAN, HSP7C\_HUMAN |
| Sequence | ``` NQVAMNPTNTVFDAK ``` |
| Position | 57 |
| PTMs | none |
| 716\*\* | Confidence | Green |
| Runs | 1, 2, 3, 4 |
| Relation | NonDiscriminating |
| Proteins | HSP7C\_HUMAN, HSP7C\_HUMAN |
| Sequence | ``` DAGTIAGLNVLR ``` |
| Position | 160 |
| PTMs | none |
| 720\*\* | Confidence | Green |
| Runs | 1, 2, 3, 4 |
| Relation | NonDiscriminating |
| Proteins | HSP7C\_HUMAN, HSP7C\_HUMAN |
| Sequence | ``` TVTNAVVTVPAYFNDSQR ``` |
| Position | 138 |
| PTMs | none |
| 749\*\* | Confidence | Green |
| Runs | 1, 2, 3, 4 |
| Relation | NonDiscriminating |
| Proteins | HSP7C\_HUMAN, HSP7C\_HUMAN, HS71L\_HUMAN, HSP72\_HUMAN, GRP78\_HUMAN |
| Sequence | ``` IINEPTAAAIAYGLDK ``` |
| Position | 172 |
| PTMs | none |
| 794\*\* | Confidence | Green |
| Runs | 1, 2, 3, 4 |
| Relation | NonDiscriminating |
| Proteins | HSP7C\_HUMAN, HSP7C\_HUMAN, HSP72\_HUMAN |
| Sequence | ``` FEELNADLFR ``` |
| Position | 302 |
| PTMs | none |
| 840\*\* | Confidence | Green |
| Runs | 1, 2, 3, 4 |
| Relation | NonDiscriminating |
| Proteins | HSP7C\_HUMAN, HSP7C\_HUMAN |
| Sequence | ``` SFYPEEVSSMVLTK ``` |
| Position | 113 |
| PTMs | none |
| 853 | Confidence | Green |
| Runs | 1, 2, 3, 4 |
| Relation | Unique |
| Proteins | HSP7C\_HUMAN |
| Sequence | ``` CNEIINWLDK ``` |
| Position | 574 |
| PTMs | Carbamidomethyl+C(1) |
| 1042\*\* | Confidence | Green |
| Runs | 1, 2, 3, 4 |
| Relation | NonDiscriminating |
| Proteins | HSP7C\_HUMAN, HSP7C\_HUMAN |
| Sequence | ``` SINPDEAVAYGAAVQAAILSGDK ``` |
| Position | 362 |
| PTMs | none |

  

Protein P07195

| Name | LDHB\_HUMAN | | |
| Description | L lactate dehydrogenase B chain OS Homo sapiens GN LDHB PE 1 SV 2 | | |
| Sequence | ```   1 MATLKEKLIA PVAEEEATVP NNKITVVGVG QVGMACAISI LGKSLADELA LVDVLEDKLK GEMMDLQHGS LFLQTPKIVA DKDYSVTANS KIVVVTAGVR 101 QQEGESRLNL VQRNVNVFKF IIPQIVKYSP DCIIIVVSNP VDILTYVTWK LSGLPKHRVI GSGCNLDSAR FRYLMAEKLG IHPSSCHGWI LGEHGDSSVA 201 VWSGVNVAGV SLQELNPEMG TDNDSENWKE VHKMVVESAY EVIKLKGYTN WAIGLSVADL IESMLKNLSR IHPVSTMVKG MYGIENEVFL SLPCILNARG 301 LTSVINQKLK DDEVAQLKKS ADTLWDIQKD LKDL ``` | | |
| Evidence | Conclusive | | |
| Peptide list | 180\*\*, 184, 213\*\*, 312, 335, 464, 613, 800, 1095 | | |
| Peptides | 180\*\* | Confidence | Green |
| Runs | 1, 2, 3, 4 |
| Relation | NonDiscriminating |
| Proteins | LDHB\_HUMAN, LDHA\_HUMAN, LDHA\_HUMAN, LDHA\_HUMAN, LDH6A\_HUMAN, LDHC\_HUMAN |
| Sequence | ``` VIGSGCNLDSAR ``` |
| Position | 159 |
| PTMs | Carbamidomethyl+C(6) |
| 184 | Confidence | Green |
| Runs | 1, 2, 3, 4 |
| Relation | Unique |
| Proteins | LDHB\_HUMAN |
| Sequence | ``` LKDDEVAQLK ``` |
| Position | 309 |
| PTMs | none |
| 213\*\* | Confidence | Green |
| Runs | 1, 2, 3 |
| Relation | NonDiscriminating |
| Proteins | LDHB\_HUMAN, LDHA\_HUMAN, LDHA\_HUMAN, LDHA\_HUMAN |
| Sequence | ``` LNLVQR ``` |
| Position | 108 |
| PTMs | none |
| 312 | Confidence | Green |
| Runs | 1, 2, 3, 4 |
| Relation | Unique |
| Proteins | LDHB\_HUMAN |
| Sequence | ``` GLTSVINQK ``` |
| Position | 300 |
| PTMs | none |
| 335 | Confidence | Green |
| Runs | 1, 2, 3, 4 |
| Relation | Unique |
| Proteins | LDHB\_HUMAN |
| Sequence | ``` IVVVTAGVR ``` |
| Position | 92 |
| PTMs | none |
| 464 | Confidence | Green |
| Runs | 1, 2, 3, 4 |
| Relation | Unique |
| Proteins | LDHB\_HUMAN |
| Sequence | ``` LIAPVAEEEATVPNNK ``` |
| Position | 8 |
| PTMs | Variant #1: none Variant #2: Deamidation+N(14) |
| 613 | Confidence | Green |
| Runs | 1, 2, 3, 4 |
| Relation | Unique |
| Proteins | LDHB\_HUMAN |
| Sequence | ``` MVVESAYEVIK ``` |
| Position | 234 |
| PTMs | none |
| 800 | Confidence | Green |
| Runs | 1, 2, 3, 4 |
| Relation | Unique |
| Proteins | LDHB\_HUMAN |
| Sequence | ``` FIIPQIVK ``` |
| Position | 120 |
| PTMs | none |
| 1095 | Confidence | Green |
| Runs | 1, 2, 3, 4 |
| Relation | Unique |
| Proteins | LDHB\_HUMAN |
| Sequence | ``` SLADELALVDVLEDK ``` |
| Position | 44 |
| PTMs | none |

  

Protein P62826

| Name | RAN\_HUMAN | | |
| Description | GTP binding nuclear protein Ran OS Homo sapiens GN RAN PE 1 SV 3 | | |
| Sequence | ```   1 MAAQGEPQVQ FKLVLVGDGG TGKTTFVKRH LTGEFEKKYV ATLGVEVHPL VFHTNRGPIK FNVWDTAGQE KFGGLRDGYY IQAQCAIIMF DVTSRVTYKN 101 VPNWHRDLVR VCENIPIVLC GNKVDIKDRK VKAKSIVFHR KKNLQYYDIS AKSNYNFEKP FLWLARKLIG DPNLEFVAMP ALAPPEVVMD PALAAQYEHD 201 LEVAQTTALP DEDDDL ``` | | |
| Evidence | Conclusive | | |
| Peptide list | 324, 560, 632 | | |
| Peptides | 324 | Confidence | Green |
| Runs | 1, 2, 3, 4 |
| Relation | Unique |
| Proteins | RAN\_HUMAN |
| Sequence | ``` LVLVGDGGTGK ``` |
| Position | 13 |
| PTMs | none |
| 560 | Confidence | Green |
| Runs | 1, 2, 4 |
| Relation | Unique |
| Proteins | RAN\_HUMAN |
| Sequence | ``` FNVWDTAGQEK ``` |
| Position | 61 |
| PTMs | none |
| 632 | Confidence | Green |
| Runs | 1, 2, 3, 4 |
| Relation | Unique |
| Proteins | RAN\_HUMAN |
| Sequence | ``` VCENIPIVLCGNK ``` |
| Position | 111 |
| PTMs | Carbamidomethyl+C(2) Carbamidomethyl+C(10) |

  

Protein P17066

| Name | HSP76\_HUMAN | | |
| Description | Heat shock 70 kDa protein 6 OS Homo sapiens GN HSPA6 PE 1 SV 2 | | |
| Sequence | ```   1 MQAPRELAVG IDLGTTYSCV GVFQQGRVEI LANDQGNRTT PSYVAFTDTE RLVGDAAKSQ AALNPHNTVF DAKRLIGRKF ADTTVQSDMK HWPFRVVSEG 101 GKPKVRVCYR GEDKTFYPEE ISSMVLSKMK ETAEAYLGQP VKHAVITVPA YFNDSQRQAT KDAGAIAGLN VLRIINEPTA AAIAYGLDRR GAGERNVLIF 201 DLGGGTFDVS VLSIDAGVFE VKATAGDTHL GGEDFDNRLV NHFMEEFRRK HGKDLSGNKR ALRRLRTACE RAKRTLSSST QATLEIDSLF EGVDFYTSIT 301 RARFEELCSD LFRSTLEPVE KALRDAKLDK AQIHDVVLVG GSTRIPKVQK LLQDFFNGKE LNKSINPDEA VAYGAAVQAA VLMGDKCEKV QDLLLLDVAP 401 LSLGLETAGG VMTTLIQRNA TIPTKQTQTF TTYSDNQPGV FIQVYEGERA MTKDNNLLGR FELSGIPPAP RGVPQIEVTF DIDANGILSV TATDRSTGKA 501 NKITITNDKG RLSKEEVERM VHEAEQYKAE DEAQRDRVAA KNSLEAHVFH VKGSLQEESL RDKIPEEDRR KMQDKCREVL AWLEHNQLAE KEEYEHQKRE 601 LEQICRPIFS RLYGGPGVPG GSSCGTQARQ GDPSTGPIIE EVD ``` | | |
| Evidence | Conclusive | | |
| Peptide list | 59\*\*, 103\*\*, 232\*\*, 506\*\*, 773\*\*, 781\*\*, 983, 1352\*\*, 1364, 1414 | | |
| Peptides | 59\*\* | Confidence | Green |
| Runs | 1, 2, 3, 4 |
| Relation | NonDiscriminating |
| Proteins | HSP71\_HUMAN, HSP7C\_HUMAN, HSP76\_HUMAN, HS71L\_HUMAN, HSP72\_HUMAN |
| Sequence | ``` ITITNDK ``` |
| Position | 503 |
| PTMs | none |
| 103\*\* | Confidence | Green |
| Runs | 1, 2, 3, 4 |
| Relation | NonDiscriminating |
| Proteins | HSP71\_HUMAN, HSP76\_HUMAN, HSP77\_HUMAN |
| Sequence | ``` STLEPVEK ``` |
| Position | 314 |
| PTMs | none |
| 232\*\* | Confidence | Green |
| Runs | 1, 2, 3, 4 |
| Relation | NonDiscriminating |
| Proteins | HSP71\_HUMAN, HSP76\_HUMAN, HS71L\_HUMAN, HSP77\_HUMAN |
| Sequence | ``` ATAGDTHLGGEDFDNR ``` |
| Position | 223 |
| PTMs | none |
| 506\*\* | Confidence | Green |
| Runs | 1, 2, 3, 4 |
| Relation | NonDiscriminating |
| Proteins | HSP71\_HUMAN, HSP7C\_HUMAN, HSP7C\_HUMAN, HSP76\_HUMAN, HS71L\_HUMAN, HSP77\_HUMAN, HSP72\_HUMAN |
| Sequence | ``` TTPSYVAFTDTER ``` |
| Position | 39 |
| PTMs | none |
| 773\*\* | Confidence | Green |
| Runs | 1, 2, 3, 4 |
| Relation | NonDiscriminating |
| Proteins | HSP71\_HUMAN, HSP76\_HUMAN |
| Sequence | ``` IINEPTAAAIAYGLDR ``` |
| Position | 174 |
| PTMs | none |
| 781\*\* | Confidence | Green |
| Runs | 1, 2, 3, 4 |
| Relation | NonDiscriminating |
| Proteins | HSP71\_HUMAN, HSP76\_HUMAN, HSP77\_HUMAN |
| Sequence | ``` FEELCSDLFR ``` |
| Position | 304 |
| PTMs | Carbamidomethyl+C(5) |
| 983 | Confidence | Green |
| Runs | 1, 3, 4 |
| Relation | Unique |
| Proteins | HSP76\_HUMAN |
| Sequence | ``` SINPDEAVAYGAAVQAAVLMGDK ``` |
| Position | 364 |
| PTMs | Variant #1: Oxidation+M(20)  Variant #2: none |
| 1352\*\* | Confidence | Green |
| Runs | 2, 3, 4 |
| Relation | NonDiscriminating |
| Proteins | HSP71\_HUMAN, HSP76\_HUMAN, HS71L\_HUMAN |
| Sequence | ``` AMTKDNNLLGR ``` |
| Position | 450 |
| PTMs | Variant #1: none Variant #2: Deamidation+N(6) |
| 1364 | Confidence | Green |
| Runs | 2, 3, 4 |
| Relation | Unique |
| Proteins | HSP76\_HUMAN |
| Sequence | ``` HAVITVPAYFNDSQR ``` |
| Position | 143 |
| PTMs | Variant #1: Deamidation+Q(14)  Variant #2: none |
| 1414 | Confidence | Green |
| Runs | 2, 3, 4 |
| Relation | Unique |
| Proteins | HSP76\_HUMAN |
| Sequence | ``` QGDPSTGPIIEEVD ``` |
| Position | 630 |
| PTMs | none |

  

Protein P04350

| Name | TBB4A\_HUMAN | | |
| Description | Tubulin beta 4A chain OS Homo sapiens GN TUBB4A PE 1 SV 2 | | |
| Sequence | ```   1 MREIVHLQAG QCGNQIGAKF WEVISDEHGI DPTGTYHGDS DLQLERINVY YNEATGGNYV PRAVLVDLEP GTMDSVRSGP FGQIFRPDNF VFGQSGAGNN 101 WAKGHYTEGA ELVDAVLDVV RKEAESCDCL QGFQLTHSLG GGTGSGMGTL LISKIREEFP DRIMNTFSVV PSPKVSDTVV EPYNATLSVH QLVENTDETY 201 CIDNEALYDI CFRTLKLTTP TYGDLNHLVS ATMSGVTTCL RFPGQLNADL RKLAVNMVPF PRLHFFMPGF APLTSRGSQQ YRALTVPELT QQMFDAKNMM 301 AACDPRHGRY LTVAAVFRGR MSMKEVDEQM LSVQSKNSSY FVEWIPNNVK TAVCDIPPRG LKMAATFIGN STAIQELFKR ISEQFTAMFR RKAFLHWYTG 401 EGMDEMEFTE AESNMNDLVS EYQQYQDATA EEGEFEEEAE EEVA ``` | | |
| Evidence | Conclusive | | |
| Peptide list | 596\*\*, 640\*\*, 738, 796\*\*, 906\*\*, 963\*\*, 995\*\*, 1080\*\* | | |
| Peptides | 596\*\* | Confidence | Green |
| Runs | 1, 2, 4 |
| Relation | NonDiscriminating |
| Proteins | TBB5\_HUMAN, TBB4B\_HUMAN, TBB2A\_HUMAN, TBB2B\_HUMAN, TBB4A\_HUMAN |
| Sequence | ``` SGPFGQIFRPDNFVFGQSGAGNNWAK ``` |
| Position | 78 |
| PTMs | Variant #1: Deamidation+N(12) Deamidation+N(22)  Variant #2: none Variant #3: Deamidation+Q(17) |
| 640\*\* | Confidence | Green |
| Runs | 1, 2, 3, 4 |
| Relation | NonDiscriminating |
| Proteins | TBB5\_HUMAN, TBB4B\_HUMAN, TBB4A\_HUMAN, TBB3\_HUMAN |
| Sequence | ``` IMNTFSVVPSPK ``` |
| Position | 163 |
| PTMs | none |
| 738 | Confidence | Green |
| Runs | 1, 2, 3, 4 |
| Relation | Unique |
| Proteins | TBB4A\_HUMAN |
| Sequence | ``` INVYYNEATGGNYVPR ``` |
| Position | 47 |
| PTMs | none |
| 796\*\* | Confidence | Green |
| Runs | 1, 2, 3, 4 |
| Relation | NonDiscriminating |
| Proteins | TBB5\_HUMAN, TBB4B\_HUMAN, TBB4A\_HUMAN |
| Sequence | ``` YLTVAAVFR ``` |
| Position | 310 |
| PTMs | none |
| 906\*\* | Confidence | Green |
| Runs | 1, 2, 3, 4 |
| Relation | NonDiscriminating |
| Proteins | TBB5\_HUMAN, TBB4B\_HUMAN, TBB2A\_HUMAN, TBB2B\_HUMAN, TBB4A\_HUMAN, TBB3\_HUMAN, TBB6\_HUMAN |
| Sequence | ``` NSSYFVEWIPNNVK ``` |
| Position | 337 |
| PTMs | none |
| 963\*\* | Confidence | Green |
| Runs | 1, 2, 3, 4 |
| Relation | NonDiscriminating |
| Proteins | TBB4B\_HUMAN, TBB4A\_HUMAN, TBB3\_HUMAN |
| Sequence | ``` ALTVPELTQQMFDAK ``` |
| Position | 283 |
| PTMs | none |
| 995\*\* | Confidence | Green |
| Runs | 1, 2, 3, 4 |
| Relation | NonDiscriminating |
| Proteins | TBB5\_HUMAN, TBB4B\_HUMAN, TBB2A\_HUMAN, TBB2B\_HUMAN, TBB4A\_HUMAN, TBB8\_HUMAN, YI016\_HUMAN, TBB8B\_HUMAN, TBB6\_HUMAN |
| Sequence | ``` LHFFMPGFAPLTSR ``` |
| Position | 263 |
| PTMs | none |
| 1080\*\* | Confidence | Green |
| Runs | 1, 2, 3, 4 |
| Relation | NonDiscriminating |
| Proteins | TBB5\_HUMAN, TBB4B\_HUMAN, TBB2A\_HUMAN, TBB2B\_HUMAN, TBB4A\_HUMAN |
| Sequence | ``` LTTPTYGDLNHLVSATMSGVTTCLR ``` |
| Position | 217 |
| PTMs | Variant #1: Carbamidomethyl+C(23)  Variant #2: Carbamidomethyl+C(23) Deamidation+N(10) |

  

Protein P22626

| Name | ROA2\_HUMAN | | |
| Description | Heterogeneous nuclear ribonucleoproteins A2 B1 OS Homo sapiens GN HNRNPA2B1 PE 1 SV 2 | | |
| Sequence | ```   1 MEKTLETVPL ERKKREKEQF RKLFIGGLSF ETTEESLRNY YEQWGKLTDC VVMRDPASKR SRGFGFVTFS SMAEVDAAMA ARPHSIDGRV VEPKRAVARE 101 ESGKPGAHVT VKKLFVGGIK EDTEEHHLRD YFEEYGKIDT IEIITDRQSG KKRGFGFVTF DDHDPVDKIV LQKYHTINGH NAEVRKALSR QEMQEVQSSR 201 SGRGGNFGFG DSRGGGGNFG PGPGSNFRGG SDGYGSGRGF GDGYNGYGGG PGGGNFGGSP GYGGGRGGYG GGGPGYGNQG GGYGGGYDNY GGGNYGSGNY 301 NDFGNYNQQP SNYGPMKSGN FGGSRNMGGP YGGGNYGPGG SGGSGGYGGR SRY ``` | | |
| Evidence | Conclusive | | |
| Peptide list | 78\*\*, 269\*\*, 341\*\*, 393\*\*, 395, 680\*\*, 756\*\* | | |
| Peptides | 78\*\* | Confidence | Green |
| Runs | 1, 2, 3, 4 |
| Relation | NonDiscriminating |
| Proteins | ROA2\_HUMAN, ROA2\_HUMAN |
| Sequence | ``` YHTINGHNAEVR ``` |
| Position | 174 |
| PTMs | Variant #1: Deamidation+N(5)  Variant #2: none |
| 269\*\* | Confidence | Green |
| Runs | 1, 3, 4 |
| Relation | NonDiscriminating |
| Proteins | ROA2\_HUMAN, ROA2\_HUMAN, ROA1\_HUMAN, ROA1\_HUMAN, ROA1\_HUMAN, RA1L2\_HUMAN |
| Sequence | ``` LTDCVVMR ``` |
| Position | 47 |
| PTMs | Carbamidomethyl+C(4) |
| 341\*\* | Confidence | Green |
| Runs | 1, 2, 3, 4 |
| Relation | NonDiscriminating |
| Proteins | ROA2\_HUMAN, ROA2\_HUMAN |
| Sequence | ``` GGNFGFGDSR ``` |
| Position | 204 |
| PTMs | none |
| 393\*\* | Confidence | Green |
| Runs | 1, 2, 4 |
| Relation | NonDiscriminating |
| Proteins | ROA2\_HUMAN, ROA2\_HUMAN |
| Sequence | ``` LFVGGIK ``` |
| Position | 114 |
| PTMs | none |
| 395 | Confidence | Green |
| Runs | 1, 2, 3 |
| Relation | Unique |
| Proteins | ROA2\_HUMAN |
| Sequence | ``` TLETVPLER ``` |
| Position | 4 |
| PTMs | none |
| 680\*\* | Confidence | Green |
| Runs | 1, 2, 3 |
| Relation | NonDiscriminating |
| Proteins | ROA2\_HUMAN, ROA2\_HUMAN |
| Sequence | ``` IDTIEIITDR ``` |
| Position | 138 |
| PTMs | none |
| 756\*\* | Confidence | Green |
| Runs | 1, 2, 3, 4 |
| Relation | NonDiscriminating |
| Proteins | ROA2\_HUMAN, ROA2\_HUMAN |
| Sequence | ``` GFGFVTFDDHDPVDK ``` |
| Position | 154 |
| PTMs | none |

  

Protein P00761

| Name | TRYP\_PIG | | |
| Description | Trypsin OS Sus scrofa PE 1 SV 1 | | |
| Sequence | ```   1 FPTDDDDKIV GGYTCAANSI PYQVSLNSGS HFCGGSLINS QWVVSAAHCY KSRIQVRLGE HNIDVLEGNE QFINAAKIIT HPNFNGNTLD NDIMLIKLSS 101 PATLNSRVAT VSLPRSCAAA GTECLISGWG NTKSSGSSYP SLLQCLKAPV LSDSSCKSSY PGQITGNMIC VGFLEGGKDS CQGDSGGPVV CNGQLQGIVS 201 WGYGCAQKNK PGVYTKVCNY VNWIQQTIAA N ``` | | |
| Evidence | Conclusive | | |
| Peptide list | 73, 176, 327, 708, 755, 874, 923, 1370 | | |
| Peptides | 73 | Confidence | Green |
| Runs | 1, 2, 3, 4 |
| Relation | Unique |
| Proteins | TRYP\_PIG |
| Sequence | ``` APVLSDSSCK ``` |
| Position | 148 |
| PTMs | Carbamidomethyl+C(9) |
| 176 | Confidence | Green |
| Runs | 1, 2, 3, 4 |
| Relation | Unique |
| Proteins | TRYP\_PIG |
| Sequence | ``` LSSPATLNSR ``` |
| Position | 98 |
| PTMs | none |
| 327 | Confidence | Green |
| Runs | 1, 2, 3, 4 |
| Relation | Unique |
| Proteins | TRYP\_PIG |
| Sequence | ``` VATVSLPR ``` |
| Position | 108 |
| PTMs | none |
| 708 | Confidence | Green |
| Runs | 1, 2, 3 |
| Relation | Unique |
| Proteins | TRYP\_PIG |
| Sequence | ``` LGEHNIDVLEGNEQFINAAK ``` |
| Position | 58 |
| PTMs | Variant #1: none Variant #2: Deamidation+N(17) |
| 755 | Confidence | Green |
| Runs | 1, 2, 3 |
| Relation | Unique |
| Proteins | TRYP\_PIG |
| Sequence | ``` SSGSSYPSLLQCLK ``` |
| Position | 134 |
| PTMs | Carbamidomethyl+C(12) |
| 874 | Confidence | Green |
| Runs | 1, 2, 3, 4 |
| Relation | Unique |
| Proteins | TRYP\_PIG |
| Sequence | ``` IITHPNFNGNTLDNDIMLIK ``` |
| Position | 78 |
| PTMs | Variant #1: none Variant #2: Deamidation+N(8)  Variant #3: Deamidation+N(10) Deamidation+N(14)  Variant #4: Deamidation+N(6) |
| 923 | Confidence | Green |
| Runs | 1, 2, 3 |
| Relation | Unique |
| Proteins | TRYP\_PIG |
| Sequence | ``` NKPGVYTKVCNYVNWIQQTIAAN ``` |
| Position | 209 |
| PTMs | Variant #1: Carbamidomethyl+C(10) Deamidation+N(23)  Variant #2: Carbamidomethyl+C(10) |
| 1370 | Confidence | Green |
| Runs | 2, 3, 4 |
| Relation | Unique |
| Proteins | TRYP\_PIG |
| Sequence | ``` DSCQGDSGGPVVCNGQLQGIVSWGYGCAQK ``` |
| Position | 179 |
| PTMs | Carbamidomethyl+C(3) Carbamidomethyl+C(13) Carbamidomethyl+C(27) |

  

Protein Q06830

| Name | PRDX1\_HUMAN | | |
| Description | Peroxiredoxin 1 OS Homo sapiens GN PRDX1 PE 1 SV 1 | | |
| Sequence | ```   1 MSSGNAKIGH PAPNFKATAV MPDGQFKDIS LSDYKGKYVV FFFYPLDFTF VCPTEIIAFS DRAEEFKKLN CQVIGASVDS HFCHLAWVNT PKKQGGLGPM 101 NIPLVSDPKR TIAQDYGVLK ADEGISFRGL FIIDDKGILR QITVNDLPVG RSVDETLRLV QAFQFTDKHG EVCPAGWKPG SDTIKPDVQK SKEYFSKQK ``` | | |
| Evidence | Conclusive | | |
| Peptide list | 70\*\*, 126, 304, 346, 353, 463, 534\*\*, 710\*\* | | |
| Peptides | 70\*\* | Confidence | Green |
| Runs | 1, 2, 3, 4 |
| Relation | NonDiscriminating |
| Proteins | PRDX1\_HUMAN, PRDX4\_HUMAN |
| Sequence | ``` SVDETLR ``` |
| Position | 152 |
| PTMs | none |
| 126 | Confidence | Green |
| Runs | 1, 2, 3, 4 |
| Relation | Unique |
| Proteins | PRDX1\_HUMAN |
| Sequence | ``` IGHPAPNFK ``` |
| Position | 8 |
| PTMs | none |
| 304 | Confidence | Green |
| Runs | 1, 2, 3, 4 |
| Relation | Unique |
| Proteins | PRDX1\_HUMAN |
| Sequence | ``` ADEGISFR ``` |
| Position | 121 |
| PTMs | none |
| 346 | Confidence | Green |
| Runs | 1, 2, 4 |
| Relation | Unique |
| Proteins | PRDX1\_HUMAN |
| Sequence | ``` HGEVCPAGWKPGSDTIKPDVQK ``` |
| Position | 169 |
| PTMs | Carbamidomethyl+C(5) |
| 353 | Confidence | Green |
| Runs | 1, 2, 3, 4 |
| Relation | Unique |
| Proteins | PRDX1\_HUMAN |
| Sequence | ``` ATAVMPDGQFK ``` |
| Position | 17 |
| PTMs | none |
| 463 | Confidence | Green |
| Runs | 1, 2, 3, 4 |
| Relation | Unique |
| Proteins | PRDX1\_HUMAN |
| Sequence | ``` TIAQDYGVLK ``` |
| Position | 111 |
| PTMs | none |
| 534\*\* | Confidence | Green |
| Runs | 1, 2, 3, 4 |
| Relation | NonDiscriminating |
| Proteins | PRDX1\_HUMAN, PRDX2\_HUMAN |
| Sequence | ``` QITVNDLPVGR ``` |
| Position | 141 |
| PTMs | none |
| 710\*\* | Confidence | Green |
| Runs | 1, 2, 3, 4 |
| Relation | NonDiscriminating |
| Proteins | PRDX1\_HUMAN, PRDX4\_HUMAN |
| Sequence | ``` GLFIIDDK ``` |
| Position | 129 |
| PTMs | none |

  

Protein P04406

| Name | G3P\_HUMAN | | |
| Description | Glyceraldehyde 3 phosphate dehydrogenase OS Homo sapiens GN GAPDH PE 1 SV 3 | | |
| Sequence | ```   1 MGKVKVGVNG FGRIGRLVTR AAFNSGKVDI VAINDPFIDL NYMVYMFQYD STHGKFHGTV KAENGKLVIN GNPITIFQER DPSKIKWGDA GAEYVVESTG 101 VFTTMEKAGA HLQGGAKRVI ISAPSADAPM FVMGVNHEKY DNSLKIISNA SCTTNCLAPL AKVIHDNFGI VEGLMTTVHA ITATQKTVDG PSGKLWRDGR 201 GALQNIIPAS TGAAKAVGKV IPELNGKLTG MAFRVPTANV SVVDLTCRLE KPAKYDDIKK VVKQASEGPL KGILGYTEHQ VVSSDFNSDT HSSTFDAGAG 301 IALNDHFVKL ISWYDNEFGY SNRVVDLMAH MASKE ``` | | |
| Evidence | Conclusive | | |
| Peptide list | 367, 581, 615, 815, 829, 947 | | |
| Peptides | 367 | Confidence | Green |
| Runs | 1, 2, 3, 4 |
| Relation | Unique |
| Proteins | G3P\_HUMAN |
| Sequence | ``` LTGMAFR ``` |
| Position | 228 |
| PTMs | none |
| 581 | Confidence | Green |
| Runs | 1, 2, 3, 4 |
| Relation | Unique |
| Proteins | G3P\_HUMAN |
| Sequence | ``` GALQNIIPASTGAAK ``` |
| Position | 201 |
| PTMs | none |
| 615 | Confidence | Green |
| Runs | 1, 2, 3, 4 |
| Relation | Unique |
| Proteins | G3P\_HUMAN |
| Sequence | ``` VPTANVSVVDLTCR ``` |
| Position | 235 |
| PTMs | Carbamidomethyl+C(13) |
| 815 | Confidence | Green |
| Runs | 1, 2, 3, 4 |
| Relation | Unique |
| Proteins | G3P\_HUMAN |
| Sequence | ``` VIISAPSADAPMFVMGVNHEK ``` |
| Position | 119 |
| PTMs | Variant #1: none Variant #2: Deamidation+N(18)  Variant #3: Oxidation+M(12) |
| 829 | Confidence | Green |
| Runs | 1, 2, 3, 4 |
| Relation | Unique |
| Proteins | G3P\_HUMAN |
| Sequence | ``` LISWYDNEFGYSNR ``` |
| Position | 310 |
| PTMs | none |
| 947 | Confidence | Green |
| Runs | 1, 2, 3, 4 |
| Relation | Unique |
| Proteins | G3P\_HUMAN |
| Sequence | ``` LVINGNPITIFQER ``` |
| Position | 67 |
| PTMs | Deamidation+N(4) |

  

Protein P10809

| Name | CH60\_HUMAN | | |
| Description | 60 kDa heat shock protein mitochondrial OS Homo sapiens GN HSPD1 PE 1 SV 2 | | |
| Sequence | ```   1 MLRLPTVFRQ MRPVSRVLAP HLTRAYAKDV KFGADARALM LQGVDLLADA VAVTMGPKGR TVIIEQSWGS PKVTKDGVTV AKSIDLKDKY KNIGAKLVQD 101 VANNTNEEAG DGTTTATVLA RSIAKEGFEK ISKGANPVEI RRGVMLAVDA VIAELKKQSK PVTTPEEIAQ VATISANGDK EIGNIISDAM KKVGRKGVIT 201 VKDGKTLNDE LEIIEGMKFD RGYISPYFIN TSKGQKCEFQ DAYVLLSEKK ISSIQSIVPA LEIANAHRKP LVIIAEDVDG EALSTLVLNR LKVGLQVVAV 301 KAPGFGDNRK NQLKDMAIAT GGAVFGEEGL TLNLEDVQPH DLGKVGEVIV TKDDAMLLKG KGDKAQIEKR IQEIIEQLDV TTSEYEKEKL NERLAKLSDG 401 VAVLKVGGTS DVEVNEKKDR VTDALNATRA AVEEGIVLGG GCALLRCIPA LDSLTPANED QKIGIEIIKR TLKIPAMTIA KNAGVEGSLI VEKIMQSSSE 501 VGYDAMAGDF VNMVEKGIID PTKVVRTALL DAAGVASLLT TAEVVVTEIP KEEKDPGMGA MGGMGGGMGG GMF ``` | | |
| Evidence | Conclusive | | |
| Peptide list | 54, 84, 107, 156, 160, 454, 475, 662, 711, 714, 818, 833, 892, 1155 | | |
| Peptides | 54 | Confidence | Green |
| Runs | 1, 2, 3, 4 |
| Relation | Unique |
| Proteins | CH60\_HUMAN |
| Sequence | ``` APGFGDNR ``` |
| Position | 302 |
| PTMs | none |
| 84 | Confidence | Green |
| Runs | 1, 2, 3, 4 |
| Relation | Unique |
| Proteins | CH60\_HUMAN |
| Sequence | ``` VGGTSDVEVNEK ``` |
| Position | 406 |
| PTMs | none |
| 107 | Confidence | Green |
| Runs | 1, 2, 3, 4 |
| Relation | Unique |
| Proteins | CH60\_HUMAN |
| Sequence | ``` VTDALNATR ``` |
| Position | 421 |
| PTMs | none |
| 156 | Confidence | Green |
| Runs | 1, 2, 3, 4 |
| Relation | Unique |
| Proteins | CH60\_HUMAN |
| Sequence | ``` GANPVEIR ``` |
| Position | 134 |
| PTMs | none |
| 160 | Confidence | Green |
| Runs | 1, 2, 3, 4 |
| Relation | Unique |
| Proteins | CH60\_HUMAN |
| Sequence | ``` VGEVIVTK ``` |
| Position | 345 |
| PTMs | none |
| 454 | Confidence | Green |
| Runs | 1, 2, 3, 4 |
| Relation | Unique |
| Proteins | CH60\_HUMAN |
| Sequence | ``` VGLQVVAVK ``` |
| Position | 293 |
| PTMs | none |
| 475 | Confidence | Green |
| Runs | 1, 3, 4 |
| Relation | Unique |
| Proteins | CH60\_HUMAN |
| Sequence | ``` LVQDVANNTNEEAGDGTTTATVLAR ``` |
| Position | 97 |
| PTMs | none |
| 662 | Confidence | Green |
| Runs | 1, 2, 3, 4 |
| Relation | Unique |
| Proteins | CH60\_HUMAN |
| Sequence | ``` CIPALDSLTPANEDQK ``` |
| Position | 447 |
| PTMs | Carbamidomethyl+C(1) |
| 711 | Confidence | Green |
| Runs | 1, 2, 3, 4 |
| Relation | Unique |
| Proteins | CH60\_HUMAN |
| Sequence | ``` EIGNIISDAMK ``` |
| Position | 181 |
| PTMs | none |
| 714 | Confidence | Green |
| Runs | 1, 3, 4 |
| Relation | Unique |
| Proteins | CH60\_HUMAN |
| Sequence | ``` TLNDELEIIEGMK ``` |
| Position | 206 |
| PTMs | Variant #1: Oxidation+M(12)  Variant #2: none |
| 818 | Confidence | Green |
| Runs | 1, 2, 3, 4 |
| Relation | Unique |
| Proteins | CH60\_HUMAN |
| Sequence | ``` AAVEEGIVLGGGCALLR ``` |
| Position | 430 |
| PTMs | Carbamidomethyl+C(13) |
| 833 | Confidence | Green |
| Runs | 1, 2, 3, 4 |
| Relation | Unique |
| Proteins | CH60\_HUMAN |
| Sequence | ``` CEFQDAYVLLSEK ``` |
| Position | 237 |
| PTMs | Carbamidomethyl+C(1) |
| 892 | Confidence | Green |
| Runs | 1, 2, 3, 4 |
| Relation | Unique |
| Proteins | CH60\_HUMAN |
| Sequence | ``` ISSIQSIVPALEIANAHR ``` |
| Position | 251 |
| PTMs | none |
| 1155 | Confidence | Green |
| Runs | 1, 2, 3, 4 |
| Relation | Unique |
| Proteins | CH60\_HUMAN |
| Sequence | ``` TALLDAAGVASLLTTAEVVVTEIPK ``` |
| Position | 527 |
| PTMs | none |

  

Protein P07737

| Name | PROF1\_HUMAN | | |
| Description | Profilin 1 OS Homo sapiens GN PFN1 PE 1 SV 2 | | |
| Sequence | ```   1 MAGWNAYIDN LMADGTCQDA AIVGYKDSPS VWAAVPGKTF VNITPAEVGV LVGKDRSSFY VNGLTLGGQK CSVIRDSLLQ DGEFSMDLRT KSTGGAPTFN 101 VTVTKTDKTL VLLMGKEGVH GGLINKKCYE MASHLRRSQY ``` | | |
| Evidence | Conclusive | | |
| Peptide list | 94, 196, 451, 562, 961, 1477 | | |
| Peptides | 94 | Confidence | Green |
| Runs | 1, 2, 3, 4 |
| Relation | Unique |
| Proteins | PROF1\_HUMAN |
| Sequence | ``` EGVHGGLINK ``` |
| Position | 117 |
| PTMs | none |
| 196 | Confidence | Green |
| Runs | 1, 2, 3 |
| Relation | Unique |
| Proteins | PROF1\_HUMAN |
| Sequence | ``` CYEMASHLR ``` |
| Position | 128 |
| PTMs | Carbamidomethyl+C(1) |
| 451 | Confidence | Green |
| Runs | 1, 2, 3, 4 |
| Relation | Unique |
| Proteins | PROF1\_HUMAN |
| Sequence | ``` STGGAPTFNVTVTK ``` |
| Position | 92 |
| PTMs | none |
| 562 | Confidence | Green |
| Runs | 1, 3, 4 |
| Relation | Unique |
| Proteins | PROF1\_HUMAN |
| Sequence | ``` DSPSVWAAVPGK ``` |
| Position | 27 |
| PTMs | none |
| 961 | Confidence | Green |
| Runs | 1, 2, 3, 4 |
| Relation | Unique |
| Proteins | PROF1\_HUMAN |
| Sequence | ``` TFVNITPAEVGVLVGK ``` |
| Position | 39 |
| PTMs | none |
| 1477 | Confidence | Green |
| Runs | 2, 3, 4 |
| Relation | Unique |
| Proteins | PROF1\_HUMAN |
| Sequence | ``` SSFYVNGLTLGGQK ``` |
| Position | 57 |
| PTMs | Variant #1: Deamidation+N(6)  Variant #2: none |

  

Protein P27348

| Name | 1433T\_HUMAN | | |
| Description | 14 3 3 protein theta OS Homo sapiens GN YWHAQ PE 1 SV 1 | | |
| Sequence | ```   1 MEKTELIQKA KLAEQAERYD DMATCMKAVT EQGAELSNEE RNLLSVAYKN VVGGRRSAWR VISSIEQKTD TSDKKLQLIK DYREKVESEL RSICTTVLEL 101 LDKYLIANAT NPESKVFYLK MKGDYFRYLA EVACGDDRKQ TIDNSQGAYQ EAFDISKKEM QPTHPIRLGL ALNFSVFYYE ILNNPELACT LAKTAFDEAI 201 AELDTLNEDS YKDSTLIMQL LRDNLTLWTS DSAGEECDAA EGAEN ``` | | |
| Evidence | Conclusive | | |
| Peptide list | 57\*\*, 88\*\*, 224, 332, 538\*\*, 1030\*\*, 1089 | | |
| Peptides | 57\*\* | Confidence | Green |
| Runs | 1, 2, 4 |
| Relation | NonDiscriminating |
| Proteins | 1433T\_HUMAN, 1433Z\_HUMAN, 1433B\_HUMAN, 1433B\_HUMAN |
| Sequence | ``` EMQPTHPIR ``` |
| Position | 159 |
| PTMs | none |
| 88\*\* | Confidence | Green |
| Runs | 1, 2, 3, 4 |
| Relation | NonDiscriminating |
| Proteins | 1433T\_HUMAN, 1433B\_HUMAN, 1433B\_HUMAN, 1433F\_HUMAN, 1433G\_HUMAN |
| Sequence | ``` VISSIEQK ``` |
| Position | 61 |
| PTMs | none |
| 224 | Confidence | Green |
| Runs | 1, 2, 3 |
| Relation | Unique |
| Proteins | 1433T\_HUMAN |
| Sequence | ``` QTIDNSQGAYQEAFDISK ``` |
| Position | 140 |
| PTMs | Variant #1: Deamidation+Q(7)  Variant #2: none |
| 332 | Confidence | Green |
| Runs | 1, 2, 4 |
| Relation | Unique |
| Proteins | 1433T\_HUMAN |
| Sequence | ``` YLIANATNPESK ``` |
| Position | 104 |
| PTMs | none |
| 538\*\* | Confidence | Green |
| Runs | 1, 2, 3, 4 |
| Relation | NonDiscriminating |
| Proteins | 1433E\_HUMAN, 1433E\_HUMAN, 1433T\_HUMAN, 1433Z\_HUMAN, 1433B\_HUMAN, 1433B\_HUMAN, 1433F\_HUMAN, 1433G\_HUMAN, 1433S\_HUMAN, 1433S\_HUMAN |
| Sequence | ``` NLLSVAYK ``` |
| Position | 42 |
| PTMs | none |
| 1030\*\* | Confidence | Green |
| Runs | 1, 2, 3, 4 |
| Relation | NonDiscriminating |
| Proteins | 1433E\_HUMAN, 1433E\_HUMAN, 1433T\_HUMAN, 1433Z\_HUMAN, 1433B\_HUMAN, 1433B\_HUMAN, 1433F\_HUMAN, 1433G\_HUMAN, 1433S\_HUMAN, 1433S\_HUMAN |
| Sequence | ``` DSTLIMQLLR ``` |
| Position | 213 |
| PTMs | none |
| 1089 | Confidence | Green |
| Runs | 1, 2, 3, 4 |
| Relation | Unique |
| Proteins | 1433T\_HUMAN |
| Sequence | ``` SICTTVLELLDK ``` |
| Position | 92 |
| PTMs | Carbamidomethyl+C(3) |

  

Protein P63104

| Name | 1433Z\_HUMAN | | |
| Description | 14 3 3 protein zeta delta OS Homo sapiens GN YWHAZ PE 1 SV 1 | | |
| Sequence | ```   1 MDKNELVQKA KLAEQAERYD DMAACMKSVT EQGAELSNEE RNLLSVAYKN VVGARRSSWR VVSSIEQKTE GAEKKQQMAR EYREKIETEL RDICNDVLSL 101 LEKFLIPNAS QAESKVFYLK MKGDYYRYLA EVAAGDDKKG IVDQSQQAYQ EAFEISKKEM QPTHPIRLGL ALNFSVFYYE ILNSPEKACS LAKTAFDEAI 201 AELDTLSEES YKDSTLIMQL LRDNLTLWTS DTQGDEAEAG EGGEN ``` | | |
| Evidence | Conclusive | | |
| Peptide list | 57\*\*, 127, 175, 538\*\*, 1030\*\*, 1096, 1123 | | |
| Peptides | 57\*\* | Confidence | Green |
| Runs | 1, 2, 4 |
| Relation | NonDiscriminating |
| Proteins | 1433T\_HUMAN, 1433Z\_HUMAN, 1433B\_HUMAN, 1433B\_HUMAN |
| Sequence | ``` EMQPTHPIR ``` |
| Position | 159 |
| PTMs | none |
| 127 | Confidence | Green |
| Runs | 1, 3, 4 |
| Relation | Unique |
| Proteins | 1433Z\_HUMAN |
| Sequence | ``` EKIETELR ``` |
| Position | 84 |
| PTMs | none |
| 175 | Confidence | Green |
| Runs | 1, 2, 3, 4 |
| Relation | Unique |
| Proteins | 1433Z\_HUMAN |
| Sequence | ``` SVTEQGAELSNEER ``` |
| Position | 28 |
| PTMs | none |
| 538\*\* | Confidence | Green |
| Runs | 1, 2, 3, 4 |
| Relation | NonDiscriminating |
| Proteins | 1433E\_HUMAN, 1433E\_HUMAN, 1433T\_HUMAN, 1433Z\_HUMAN, 1433B\_HUMAN, 1433B\_HUMAN, 1433F\_HUMAN, 1433G\_HUMAN, 1433S\_HUMAN, 1433S\_HUMAN |
| Sequence | ``` NLLSVAYK ``` |
| Position | 42 |
| PTMs | none |
| 1030\*\* | Confidence | Green |
| Runs | 1, 2, 3, 4 |
| Relation | NonDiscriminating |
| Proteins | 1433E\_HUMAN, 1433E\_HUMAN, 1433T\_HUMAN, 1433Z\_HUMAN, 1433B\_HUMAN, 1433B\_HUMAN, 1433F\_HUMAN, 1433G\_HUMAN, 1433S\_HUMAN, 1433S\_HUMAN |
| Sequence | ``` DSTLIMQLLR ``` |
| Position | 213 |
| PTMs | none |
| 1096 | Confidence | Green |
| Runs | 1, 2, 3, 4 |
| Relation | Unique |
| Proteins | 1433Z\_HUMAN |
| Sequence | ``` DICNDVLSLLEK ``` |
| Position | 92 |
| PTMs | Carbamidomethyl+C(3) |
| 1123 | Confidence | Green |
| Runs | 1, 2, 3, 4 |
| Relation | Unique |
| Proteins | 1433Z\_HUMAN |
| Sequence | ``` TAFDEAIAELDTLSEESYK ``` |
| Position | 194 |
| PTMs | none |

  

Protein P34931

| Name | HS71L\_HUMAN | | |
| Description | Heat shock 70 kDa protein 1 like OS Homo sapiens GN HSPA1L PE 1 SV 2 | | |
| Sequence | ```   1 MATAKGIAIG IDLGTTYSCV GVFQHGKVEI IANDQGNRTT PSYVAFTDTE RLIGDAAKNQ VAMNPQNTVF DAKRLIGRKF NDPVVQADMK LWPFQVINEG 101 GKPKVLVSYK GENKAFYPEE ISSMVLTKLK ETAEAFLGHP VTNAVITVPA YFNDSQRQAT KDAGVIAGLN VLRIINEPTA AAIAYGLDKG GQGERHVLIF 201 DLGGGTFDVS ILTIDDGIFE VKATAGDTHL GGEDFDNRLV SHFVEEFKRK HKKDISQNKR AVRRLRTACE RAKRTLSSST QANLEIDSLY EGIDFYTSIT 301 RARFEELCAD LFRGTLEPVE KALRDAKMDK AKIHDIVLVG GSTRIPKVQR LLQDYFNGRD LNKSINPDEA VAYGAAVQAA ILMGDKSEKV QDLLLLDVAP 401 LSLGLETAGG VMTALIKRNS TIPTKQTQIF TTYSDNQPGV LIQVYEGERA MTKDNNLLGR FDLTGIPPAP RGVPQIEVTF DIDANGILNV TATDKSTGKV 501 NKITITNDKG RLSKEEIERM VLDAEKYKAE DEVQREKIAA KNALESYAFN MKSVVSDEGL KGKISESDKN KILDKCNELL SWLEVNQLAE KDEFDHKRKE 601 LEQMCNPIIT KLYQGGCTGP ACGTGYVPGR PATGPTIEEV D ``` | | |
| Evidence | Conclusive | | |
| Peptide list | 20\*\*, 59\*\*, 178\*\*, 232\*\*, 380, 506\*\*, 749\*\*, 812\*\*, 1352\*\* | | |
| Peptides | 20\*\* | Confidence | Green |
| Runs | 1, 2, 3, 4 |
| Relation | NonDiscriminating |
| Proteins | HSP71\_HUMAN, HS71L\_HUMAN |
| Sequence | ``` YKAEDEVQR ``` |
| Position | 527 |
| PTMs | none |
| 59\*\* | Confidence | Green |
| Runs | 1, 2, 3, 4 |
| Relation | NonDiscriminating |
| Proteins | HSP71\_HUMAN, HSP7C\_HUMAN, HSP76\_HUMAN, HS71L\_HUMAN, HSP72\_HUMAN |
| Sequence | ``` ITITNDK ``` |
| Position | 503 |
| PTMs | none |
| 178\*\* | Confidence | Green |
| Runs | 1, 2, 3, 4 |
| Relation | NonDiscriminating |
| Proteins | HSP71\_HUMAN, HSP7C\_HUMAN, HSP7C\_HUMAN, HS71L\_HUMAN, HSP72\_HUMAN, GRP78\_HUMAN |
| Sequence | ``` VEIIANDQGNR ``` |
| Position | 28 |
| PTMs | none |
| 232\*\* | Confidence | Green |
| Runs | 1, 2, 3, 4 |
| Relation | NonDiscriminating |
| Proteins | HSP71\_HUMAN, HSP76\_HUMAN, HS71L\_HUMAN, HSP77\_HUMAN |
| Sequence | ``` ATAGDTHLGGEDFDNR ``` |
| Position | 223 |
| PTMs | none |
| 380 | Confidence | Green |
| Runs | 1, 2, 4 |
| Relation | Unique |
| Proteins | HS71L\_HUMAN |
| Sequence | ``` NQVAMNPQNTVFDAK ``` |
| Position | 59 |
| PTMs | none |
| 506\*\* | Confidence | Green |
| Runs | 1, 2, 3, 4 |
| Relation | NonDiscriminating |
| Proteins | HSP71\_HUMAN, HSP7C\_HUMAN, HSP7C\_HUMAN, HSP76\_HUMAN, HS71L\_HUMAN, HSP77\_HUMAN, HSP72\_HUMAN |
| Sequence | ``` TTPSYVAFTDTER ``` |
| Position | 39 |
| PTMs | none |
| 749\*\* | Confidence | Green |
| Runs | 1, 2, 3, 4 |
| Relation | NonDiscriminating |
| Proteins | HSP7C\_HUMAN, HSP7C\_HUMAN, HS71L\_HUMAN, HSP72\_HUMAN, GRP78\_HUMAN |
| Sequence | ``` IINEPTAAAIAYGLDK ``` |
| Position | 174 |
| PTMs | none |
| 812\*\* | Confidence | Green |
| Runs | 1, 2, 3, 4 |
| Relation | NonDiscriminating |
| Proteins | HSP71\_HUMAN, HS71L\_HUMAN |
| Sequence | ``` DAGVIAGLNVLR ``` |
| Position | 162 |
| PTMs | none |
| 1352\*\* | Confidence | Green |
| Runs | 2, 3, 4 |
| Relation | NonDiscriminating |
| Proteins | HSP71\_HUMAN, HSP76\_HUMAN, HS71L\_HUMAN |
| Sequence | ``` AMTKDNNLLGR ``` |
| Position | 450 |
| PTMs | Variant #1: none Variant #2: Deamidation+N(6) |

  

Protein P12277

| Name | KCRB\_HUMAN | | |
| Description | Creatine kinase B type OS Homo sapiens GN CKB PE 1 SV 1 | | |
| Sequence | ```   1 MPFSNSHNAL KLRFPAEDEF PDLSAHNNHM AKVLTPELYA ELRAKSTPSG FTLDDVIQTG VDNPGHPYIM TVGCVAGDEE SYEVFKDLFD PIIEDRHGGY 101 KPSDEHKTDL NPDNLQGGDD LDPNYVLSSR VRTGRSIRGF CLPPHCSRGE RRAIEKLAVE ALSSLDGDLA GRYYALKSMT EAEQQQLIDD HFLFDKPVSP 201 LLLASGMARD WPDARGIWHN DNKTFLVWVN EEDHLRVISM QKGGNMKEVF TRFCTGLTQI ETLFKSKDYE FMWNPHLGYI LTCPSNLGTG LRAGVHIKLP 301 NLGKHEKFSE VLKRLRLQKR GTGGVDTAAV GGVFDVSNAD RLGFSEVELV QMVVDGVKLL IEMEQRLEQG QAIDDLMPAQ K ``` | | |
| Evidence | Conclusive | | |
| Peptide list | 234, 283, 608, 690, 746, 768, 865, 1046, 1551 | | |
| Peptides | 234 | Confidence | Green |
| Runs | 1, 2, 4 |
| Relation | Unique |
| Proteins | KCRB\_HUMAN |
| Sequence | ``` FSEVLK ``` |
| Position | 308 |
| PTMs | none |
| 283 | Confidence | Green |
| Runs | 1, 3, 4 |
| Relation | Unique |
| Proteins | KCRB\_HUMAN |
| Sequence | ``` PFSNSHNALK ``` |
| Position | 2 |
| PTMs | none |
| 608 | Confidence | Green |
| Runs | 1, 2, 3 |
| Relation | Unique |
| Proteins | KCRB\_HUMAN |
| Sequence | ``` LEQGQAIDDLMPAQK ``` |
| Position | 367 |
| PTMs | Variant #1: none Variant #2: Deamidation+Q(3) Deamidation+Q(14) |
| 690 | Confidence | Green |
| Runs | 1, 2, 3, 4 |
| Relation | Unique |
| Proteins | KCRB\_HUMAN |
| Sequence | ``` GTGGVDTAAVGGVFDVSNADR ``` |
| Position | 321 |
| PTMs | none |
| 746 | Confidence | Green |
| Runs | 1, 2, 3, 4 |
| Relation | Unique |
| Proteins | KCRB\_HUMAN |
| Sequence | ``` VLTPELYAELR ``` |
| Position | 33 |
| PTMs | none |
| 768 | Confidence | Green |
| Runs | 1, 2, 3, 4 |
| Relation | Unique |
| Proteins | KCRB\_HUMAN |
| Sequence | ``` TDLNPDNLQGGDDLDPNYVLSSR ``` |
| Position | 108 |
| PTMs | Variant #1: none Variant #2: Deamidation+N(4)  Variant #3: Deamidation+N(17) Deamidation+Q(9) |
| 865 | Confidence | Green |
| Runs | 1, 2, 3, 4 |
| Relation | Unique |
| Proteins | KCRB\_HUMAN |
| Sequence | ``` DLFDPIIEDR ``` |
| Position | 87 |
| PTMs | none |
| 1046 | Confidence | Green |
| Runs | 1, 2, 3, 4 |
| Relation | Unique |
| Proteins | KCRB\_HUMAN |
| Sequence | ``` FCTGLTQIETLFK ``` |
| Position | 253 |
| PTMs | Carbamidomethyl+C(2) |
| 1551 | Confidence | Green |
| Runs | 2, 3, 4 |
| Relation | Unique |
| Proteins | KCRB\_HUMAN |
| Sequence | ``` LAVEALSSLDGDLAGR ``` |
| Position | 157 |
| PTMs | none |

  

Protein P06733

| Name | ENOA\_HUMAN | | |
| Description | Alpha enolase OS Homo sapiens GN ENO1 PE 1 SV 2 | | |
| Sequence | ```   1 MSILKIHARE IFDSRGNPTV EVDLFTSKGL FRAAVPSGAS TGIYEALELR DNDKTRYMGK GVSKAVEHIN KTIAPALVSK KLNVTEQEKI DKLMIEMDGT 101 ENKSKFGANA ILGVSLAVCK AGAVEKGVPL YRHIADLAGN SEVILPVPAF NVINGGSHAG NKLAMQEFMI LPVGAANFRE AMRIGAEVYH NLKNVIKEKY 201 GKDATNVGDE GGFAPNILEN KEGLELLKTA IGKAGYTDKV VIGMDVAASE FFRSGKYDLD FKSPDDPSRY ISPDQLADLY KSFIKDYPVV SIEDPFDQDD 301 WGAWQKFTAS AGIQVVGDDL TVTNPKRIAK AVNEKSCNCL LLKVNQIGSV TESLQACKLA QANGWGVMVS HRSGETEDTF IADLVVGLCT GQIKTGAPCR 401 SERLAKYNQL LRIEEELGSK AKFAGRNFRN PLAK ``` | | |
| Evidence | Conclusive | | |
| Peptide list | 56, 80\*\*, 227\*\*, 300, 489\*\*, 498\*\*, 783\*\*, 870\*\*, 949\*\*, 1269\*\*, 1675\*\* | | |
| Peptides | 56 | Confidence | Green |
| Runs | 1, 2, 3 |
| Relation | Unique |
| Proteins | ENOA\_HUMAN |
| Sequence | ``` LNVTEQEK ``` |
| Position | 82 |
| PTMs | none |
| 80\*\* | Confidence | Green |
| Runs | 1, 2, 3 |
| Relation | NonDiscriminating |
| Proteins | ENOA\_HUMAN, ENOA\_HUMAN |
| Sequence | ``` IEEELGSK ``` |
| Position | 413 |
| PTMs | none |
| 227\*\* | Confidence | Green |
| Runs | 1, 2, 3, 4 |
| Relation | NonDiscriminating |
| Proteins | ENOA\_HUMAN, ENOA\_HUMAN |
| Sequence | ``` IGAEVYHNLK ``` |
| Position | 184 |
| PTMs | none |
| 300 | Confidence | Green |
| Runs | 1, 2, 3, 4 |
| Relation | Unique |
| Proteins | ENOA\_HUMAN |
| Sequence | ``` TIAPALVSK ``` |
| Position | 72 |
| PTMs | none |
| 489\*\* | Confidence | Green |
| Runs | 1, 2, 4 |
| Relation | NonDiscriminating |
| Proteins | ENOA\_HUMAN, ENOA\_HUMAN |
| Sequence | ``` YGKDATNVGDEGGFAPNILENK ``` |
| Position | 200 |
| PTMs | Variant #1: Deamidation+N(17)  Variant #2: none Variant #3: Deamidation+N(7) |
| 498\*\* | Confidence | Green |
| Runs | 1, 2, 3, 4 |
| Relation | NonDiscriminating |
| Proteins | ENOA\_HUMAN, ENOA\_HUMAN |
| Sequence | ``` VNQIGSVTESLQACK ``` |
| Position | 344 |
| PTMs | Carbamidomethyl+C(14) |
| 783\*\* | Confidence | Green |
| Runs | 1, 2, 3, 4 |
| Relation | NonDiscriminating |
| Proteins | ENOA\_HUMAN, ENOA\_HUMAN |
| Sequence | ``` YISPDQLADLYK ``` |
| Position | 270 |
| PTMs | none |
| 870\*\* | Confidence | Green |
| Runs | 1, 2, 4 |
| Relation | NonDiscriminating |
| Proteins | ENOA\_HUMAN, ENOB\_HUMAN, ENOB\_HUMAN, ENOB\_HUMAN, ENOG\_HUMAN |
| Sequence | ``` AAVPSGASTGIYEALELR ``` |
| Position | 33 |
| PTMs | none |
| 949\*\* | Confidence | Green |
| Runs | 1, 2, 3 |
| Relation | NonDiscriminating |
| Proteins | ENOA\_HUMAN, ENOA\_HUMAN |
| Sequence | ``` HIADLAGNSEVILPVPAFNVINGGSHAGNK ``` |
| Position | 133 |
| PTMs | Deamidation+N(22) |
| 1269\*\* | Confidence | Green |
| Runs | 2, 3, 4 |
| Relation | NonDiscriminating |
| Proteins | ENOA\_HUMAN, ENOA\_HUMAN |
| Sequence | ``` YNQLLR ``` |
| Position | 407 |
| PTMs | none |
| 1675\*\* | Confidence | Green |
| Runs | 2, 3, 4 |
| Relation | NonDiscriminating |
| Proteins | ENOA\_HUMAN, ENOA\_HUMAN |
| Sequence | ``` DYPVVSIEDPFDQDDWGAWQK ``` |
| Position | 286 |
| PTMs | none |

  

Protein Q9BTM1-2

| Name | H2AJ\_HUMAN | | |
| Description | Isoform 2 of Histone H2A J OS Homo sapiens GN H2AFJ | | |
| Sequence | ```   1 MSGRGKQGGK VRAKAKSRSS RAGLQFPVGR VHRLLRKGNY AERVGAGAPV YLAAVLEYLT AEILELAGNA ARDNKKTRII PRHLQLAIRN DEELNKLLGK 101 VTIAQGGVLP NIQAVLLPVC EHSGPSSGKI PSDRAELGAG SVCGHIFQKV E ``` | | |
| Evidence | Conclusive | | |
| Peptide list | 298\*\*, 551\*\*, 731 | | |
| Peptides | 298\*\* | Confidence | Green |
| Runs | 1, 2, 3, 4 |
| Relation | NonDiscriminating |
| Proteins | H2A1A\_HUMAN, H2AX\_HUMAN, H2A1J\_HUMAN, H2A2A\_HUMAN, H2A3\_HUMAN, H2A1B\_HUMAN, H2A2C\_HUMAN, H2A1C\_HUMAN, H2AJ\_HUMAN, H2A1\_HUMAN, H2A1D\_HUMAN, H2A1H\_HUMAN, H2AJ\_HUMAN, H2AZ\_HUMAN, H2AV\_HUMAN |
| Sequence | ``` HLQLAIR ``` |
| Position | 83 |
| PTMs | none |
| 551\*\* | Confidence | Green |
| Runs | 1, 2, 3, 4 |
| Relation | NonDiscriminating |
| Proteins | H2A1A\_HUMAN, H2AX\_HUMAN, H2A1J\_HUMAN, H2A2A\_HUMAN, H2A3\_HUMAN, H2A1B\_HUMAN, H2A2C\_HUMAN, H2A1C\_HUMAN, H2AJ\_HUMAN, H2A1\_HUMAN, H2A1D\_HUMAN, H2A1H\_HUMAN, H2A2B\_HUMAN, H2AJ\_HUMAN, H2AZ\_HUMAN, H2AV\_HUMAN |
| Sequence | ``` AGLQFPVGR ``` |
| Position | 22 |
| PTMs | none |
| 731 | Confidence | Green |
| Runs | 1, 2, 3 |
| Relation | Unique |
| Proteins | H2AJ\_HUMAN |
| Sequence | ``` AELGAGSVCGHIFQK ``` |
| Position | 135 |
| PTMs | Variant #1: Carbamidomethyl+C(9) Deamidation+Q(14)  Variant #2: Carbamidomethyl+C(9) |

  

Protein P62081

| Name | RS7\_HUMAN | | |
| Description | 40S ribosomal protein S7 OS Homo sapiens GN RPS7 PE 1 SV 1 | | |
| Sequence | ```   1 MFSSSAKIVK PNGEKPDEFE SGISQALLEL EMNSDLKAQL RELNITAAKE IEVGGGRKAI IIFVPVPQLK SFQKIQVRLV RELEKKFSGK HVVFIAQRRI 101 LPKPTRKSRT KNKQKRPRSR TLTAVHDAIL EDLVFPSEIV GKRIRVKLDG SRLIKVHLDK AQQNNVEHKV ETFSGVYKKL TGKDVNFEFP EFQL ``` | | |
| Evidence | Conclusive | | |
| Peptide list | 1052, 1111 | | |
| Peptides | 1052 | Confidence | Green |
| Runs | 1, 2, 3, 4 |
| Relation | Unique |
| Proteins | RS7\_HUMAN |
| Sequence | ``` AIIIFVPVPQLK ``` |
| Position | 59 |
| PTMs | none |
| 1111 | Confidence | Green |
| Runs | 1, 2, 3, 4 |
| Relation | Unique |
| Proteins | RS7\_HUMAN |
| Sequence | ``` TLTAVHDAILEDLVFPSEIVGK ``` |
| Position | 121 |
| PTMs | none |

  

Protein Q04917

| Name | 1433F\_HUMAN | | |
| Description | 14 3 3 protein eta OS Homo sapiens GN YWHAH PE 1 SV 4 | | |
| Sequence | ```   1 MGDREQLLQR ARLAEQAERY DDMASAMKAV TELNEPLSNE DRNLLSVAYK NVVGARRSSW RVISSIEQKT MADGNEKKLE KVKAYREKIE KELETVCNDV 101 LSLLDKFLIK NCNDFQYESK VFYLKMKGDY YRYLAEVASG EKKNSVVEAS EAAYKEAFEI SKEQMQPTHP IRLGLALNFS VFYYEIQNAP EQACLLAKQA 201 FDDAIAELDT LNEDSYKDST LIMQLLRDNL TLWTSDQQDE EAGEGN ``` | | |
| Evidence | Conclusive | | |
| Peptide list | 88\*\*, 262, 382, 538\*\*, 1030\*\* | | |
| Peptides | 88\*\* | Confidence | Green |
| Runs | 1, 2, 3, 4 |
| Relation | NonDiscriminating |
| Proteins | 1433T\_HUMAN, 1433B\_HUMAN, 1433B\_HUMAN, 1433F\_HUMAN, 1433G\_HUMAN |
| Sequence | ``` VISSIEQK ``` |
| Position | 62 |
| PTMs | none |
| 262 | Confidence | Green |
| Runs | 1, 3, 4 |
| Relation | Unique |
| Proteins | 1433F\_HUMAN |
| Sequence | ``` NSVVEASEAAYK ``` |
| Position | 144 |
| PTMs | none |
| 382 | Confidence | Green |
| Runs | 1, 3, 4 |
| Relation | Unique |
| Proteins | 1433F\_HUMAN |
| Sequence | ``` AVTELNEPLSNEDR ``` |
| Position | 29 |
| PTMs | none |
| 538\*\* | Confidence | Green |
| Runs | 1, 2, 3, 4 |
| Relation | NonDiscriminating |
| Proteins | 1433E\_HUMAN, 1433E\_HUMAN, 1433T\_HUMAN, 1433Z\_HUMAN, 1433B\_HUMAN, 1433B\_HUMAN, 1433F\_HUMAN, 1433G\_HUMAN, 1433S\_HUMAN, 1433S\_HUMAN |
| Sequence | ``` NLLSVAYK ``` |
| Position | 43 |
| PTMs | none |
| 1030\*\* | Confidence | Green |
| Runs | 1, 2, 3, 4 |
| Relation | NonDiscriminating |
| Proteins | 1433E\_HUMAN, 1433E\_HUMAN, 1433T\_HUMAN, 1433Z\_HUMAN, 1433B\_HUMAN, 1433B\_HUMAN, 1433F\_HUMAN, 1433G\_HUMAN, 1433S\_HUMAN, 1433S\_HUMAN |
| Sequence | ``` DSTLIMQLLR ``` |
| Position | 218 |
| PTMs | none |

  

Protein P62937

| Name | PPIA\_HUMAN | | |
| Description | Peptidyl prolyl cis trans isomerase A OS Homo sapiens GN PPIA PE 1 SV 2 | | |
| Sequence | ```   1 MVNPTVFFDI AVDGEPLGRV SFELFADKVP KTAENFRALS TGEKGFGYKG SCFHRIIPGF MCQGGDFTRH NGTGGKSIYG EKFEDENFIL KHTGPGILSM 101 ANAGPNTNGS QFFICTAKTE WLDGKHVVFG KVKEGMNIVE AMERFGSRNG KTSKKITIAD CGQLE ``` | | |
| Evidence | Conclusive | | |
| Peptide list | 566, 673, 742\*\*, 789, 1013 | | |
| Peptides | 566 | Confidence | Green |
| Runs | 1, 2, 4 |
| Relation | Unique |
| Proteins | PPIA\_HUMAN |
| Sequence | ``` HTGPGILSMANAGPNTNGSQFFICTAK ``` |
| Position | 92 |
| PTMs | Variant #1: Carbamidomethyl+C(24)  Variant #2: Carbamidomethyl+C(24) Deamidation+Q(20)  Variant #3: Carbamidomethyl+C(24) Deamidation+N(17) |
| 673 | Confidence | Green |
| Runs | 1, 2, 3, 4 |
| Relation | Unique |
| Proteins | PPIA\_HUMAN |
| Sequence | ``` FEDENFILK ``` |
| Position | 83 |
| PTMs | none |
| 742\*\* | Confidence | Green |
| Runs | 1, 2, 3, 4 |
| Relation | NonDiscriminating |
| Proteins | PPIA\_HUMAN, PAL4A\_HUMAN |
| Sequence | ``` IIPGFMCQGGDFTR ``` |
| Position | 56 |
| PTMs | Carbamidomethyl+C(7) |
| 789 | Confidence | Green |
| Runs | 1, 2, 3, 4 |
| Relation | Unique |
| Proteins | PPIA\_HUMAN |
| Sequence | ``` VSFELFADK ``` |
| Position | 20 |
| PTMs | none |
| 1013 | Confidence | Green |
| Runs | 1, 3, 4 |
| Relation | Unique |
| Proteins | PPIA\_HUMAN |
| Sequence | ``` MVNPTVFFDIAVDGEPLGR ``` |
| Position | 1 |
| PTMs | Variant #1: none Variant #2: Acetyl+N-TERM(1) |

  

Protein P08865

| Name | RSSA\_HUMAN | | |
| Description | 40S ribosomal protein SA OS Homo sapiens GN RPSA PE 1 SV 4 | | |
| Sequence | ```   1 MSGALDVLQM KEEDVLKFLA AGTHLGGTNL DFQMEQYIYK RKSDGIYIIN LKRTWEKLLL AARAIVAIEN PADVSVISSR NTGQRAVLKF AAATGATPIA 101 GRFTPGTFTN QIQAAFREPR LLVVTDPRAD HQPLTEASYV NLPTIALCNT DSPLRYVDIA IPCNNKGAHS VGLMWWMLAR EVLRMRGTIS REHPWEVMPD 201 LYFYRDPEEI EKEEQAAAEK AVTKEEFQGE WTAPAPEFTA TQPEVADWSE GVQVPSVPIQ QFPTEDWSAQ PATEDWSAAP TAQATEWVGA TTDWS ``` | | |
| Evidence | Conclusive | | |
| Peptide list | 275, 365, 729, 930 | | |
| Peptides | 275 | Confidence | Green |
| Runs | 1, 2, 4 |
| Relation | Unique |
| Proteins | RSSA\_HUMAN |
| Sequence | ``` FAAATGATPIAGR ``` |
| Position | 90 |
| PTMs | none |
| 365 | Confidence | Green |
| Runs | 1, 2, 3 |
| Relation | Unique |
| Proteins | RSSA\_HUMAN |
| Sequence | ``` DPEEIEKEEQAAAEK ``` |
| Position | 206 |
| PTMs | none |
| 729 | Confidence | Green |
| Runs | 1, 2, 3, 4 |
| Relation | Unique |
| Proteins | RSSA\_HUMAN |
| Sequence | ``` AIVAIENPADVSVISSR ``` |
| Position | 64 |
| PTMs | none |
| 930 | Confidence | Green |
| Runs | 1, 2, 3, 4 |
| Relation | Unique |
| Proteins | RSSA\_HUMAN |
| Sequence | ``` ADHQPLTEASYVNLPTIALCNTDSPLR ``` |
| Position | 129 |
| PTMs | Carbamidomethyl+C(20) |

  

Protein P32119

| Name | PRDX2\_HUMAN | | |
| Description | Peroxiredoxin 2 OS Homo sapiens GN PRDX2 PE 1 SV 5 | | |
| Sequence | ```   1 MASGNARIGK PAPDFKATAV VDGAFKEVKL SDYKGKYVVL FFYPLDFTFV CPTEIIAFSN RAEDFRKLGC EVLGVSVDSQ FTHLAWINTP RKEGGLGPLN 101 IPLLADVTRR LSEDYGVLKT DEGIAYRGLF IIDGKGVLRQ ITVNDLPVGR SVDEALRLVQ AFQYTDEHGE VCPAGWKPGS DTIKPNVDDS KEYFSKHN ``` | | |
| Evidence | Conclusive | | |
| Peptide list | 534\*\*, 1043 | | |
| Peptides | 534\*\* | Confidence | Green |
| Runs | 1, 2, 3, 4 |
| Relation | NonDiscriminating |
| Proteins | PRDX1\_HUMAN, PRDX2\_HUMAN |
| Sequence | ``` QITVNDLPVGR ``` |
| Position | 140 |
| PTMs | none |
| 1043 | Confidence | Green |
| Runs | 1, 2, 3 |
| Relation | Unique |
| Proteins | PRDX2\_HUMAN |
| Sequence | ``` KEGGLGPLNIPLLADVTR ``` |
| Position | 92 |
| PTMs | none |

  

Protein P40926

| Name | MDHM\_HUMAN | | |
| Description | Malate dehydrogenase mitochondrial OS Homo sapiens GN MDH2 PE 1 SV 3 | | |
| Sequence | ```   1 MLSALARPAS AALRRSFSTS AQNNAKVAVL GASGGIGQPL SLLLKNSPLV SRLTLYDIAH TPGVAADLSH IETKAAVKGY LGPEQLPDCL KGCDVVVIPA 101 GVPRKPGMTR DDLFNTNATI VATLTAACAQ HCPEAMICVI ANPVNSTIPI TAEVFKKHGV YNPNKIFGVT TLDIVRANTF VAELKGLDPA RVNVPVIGGH 201 AGKTIIPLIS QCTPKVDFPQ DQLTALTGRI QEAGTEVVKA KAGAGSATLS MAYAGARFVF SLVDAMNGKE GVVECSFVKS QETECTYFST PLLLGKKGIE 301 KNLGIGKVSS FEEKMISDAI PELKASIKKG EDFVKTLK ``` | | |
| Evidence | Conclusive | | |
| Peptide list | 624, 905, 912, 1092 | | |
| Peptides | 624 | Confidence | Green |
| Runs | 1, 2, 3, 4 |
| Relation | Unique |
| Proteins | MDHM\_HUMAN |
| Sequence | ``` GCDVVVIPAGVPR ``` |
| Position | 92 |
| PTMs | Carbamidomethyl+C(2) |
| 905 | Confidence | Green |
| Runs | 1, 2, 3, 4 |
| Relation | Unique |
| Proteins | MDHM\_HUMAN |
| Sequence | ``` LTLYDIAHTPGVAADLSHIETK ``` |
| Position | 53 |
| PTMs | none |
| 912 | Confidence | Green |
| Runs | 1, 2, 3, 4 |
| Relation | Unique |
| Proteins | MDHM\_HUMAN |
| Sequence | ``` IFGVTTLDIVR ``` |
| Position | 166 |
| PTMs | none |
| 1092 | Confidence | Green |
| Runs | 1, 2, 3, 4 |
| Relation | Unique |
| Proteins | MDHM\_HUMAN |
| Sequence | ``` VAVLGASGGIGQPLSLLLK ``` |
| Position | 27 |
| PTMs | none |

  

Protein P61981

| Name | 1433G\_HUMAN | | |
| Description | 14 3 3 protein gamma OS Homo sapiens GN YWHAG PE 1 SV 2 | | |
| Sequence | ```   1 MVDREQLVQK ARLAEQAERY DDMAAAMKNV TELNEPLSNE ERNLLSVAYK NVVGARRSSW RVISSIEQKT SADGNEKKIE MVRAYREKIE KELEAVCQDV 101 LSLLDNYLIK NCSETQYESK VFYLKMKGDY YRYLAEVATG EKRATVVESS EKAYSEAHEI SKEHMQPTHP IRLGLALNYS VFYYEIQNAP EQACHLAKTA 201 FDDAIAELDT LNEDSYKDST LIMQLLRDNL TLWTSDQQDD DGGEGNN ``` | | |
| Evidence | Conclusive | | |
| Peptide list | 88\*\*, 538\*\*, 1030\*\*, 1153 | | |
| Peptides | 88\*\* | Confidence | Green |
| Runs | 1, 2, 3, 4 |
| Relation | NonDiscriminating |
| Proteins | 1433T\_HUMAN, 1433B\_HUMAN, 1433B\_HUMAN, 1433F\_HUMAN, 1433G\_HUMAN |
| Sequence | ``` VISSIEQK ``` |
| Position | 62 |
| PTMs | none |
| 538\*\* | Confidence | Green |
| Runs | 1, 2, 3, 4 |
| Relation | NonDiscriminating |
| Proteins | 1433E\_HUMAN, 1433E\_HUMAN, 1433T\_HUMAN, 1433Z\_HUMAN, 1433B\_HUMAN, 1433B\_HUMAN, 1433F\_HUMAN, 1433G\_HUMAN, 1433S\_HUMAN, 1433S\_HUMAN |
| Sequence | ``` NLLSVAYK ``` |
| Position | 43 |
| PTMs | none |
| 1030\*\* | Confidence | Green |
| Runs | 1, 2, 3, 4 |
| Relation | NonDiscriminating |
| Proteins | 1433E\_HUMAN, 1433E\_HUMAN, 1433T\_HUMAN, 1433Z\_HUMAN, 1433B\_HUMAN, 1433B\_HUMAN, 1433F\_HUMAN, 1433G\_HUMAN, 1433S\_HUMAN, 1433S\_HUMAN |
| Sequence | ``` DSTLIMQLLR ``` |
| Position | 218 |
| PTMs | none |
| 1153 | Confidence | Green |
| Runs | 1, 3, 4 |
| Relation | Unique |
| Proteins | 1433G\_HUMAN |
| Sequence | ``` ELEAVCQDVLSLLDNYLIK ``` |
| Position | 92 |
| PTMs | Carbamidomethyl+C(6) |

  

Protein P08670

| Name | VIME\_HUMAN | | |
| Description | Vimentin OS Homo sapiens GN VIM PE 1 SV 4 | | |
| Sequence | ```   1 MSTRSVSSSS YRRMFGGPGT ASRPSSSRSY VTTSTRTYSL GSALRPSTSR SLYASSPGGV YATRSSAVRL RSSVPGVRLL QDSVDFSLAD AINTEFKNTR 101 TNEKVELQEL NDRFANYIDK VRFLEQQNKI LLAELEQLKG QGKSRLGDLY EEEMRELRRQ VDQLTNDKAR VEVERDNLAE DIMRLREKLQ EEMLQREEAE 201 NTLQSFRQDV DNASLARLDL ERKVESLQEE IAFLKKLHEE EIQELQAQIQ EQHVQIDVDV SKPDLTAALR DVRQQYESVA AKNLQEAEEW YKSKFADLSE 301 AANRNNDALR QAKQESTEYR RQVQSLTCEV DALKGTNESL ERQMREMEEN FAVEAANYQD TIGRLQDEIQ NMKEEMARHL REYQDLLNVK MALDIEIATY 401 RKLLEGEESR ISLPLPNFSS LNLRETNLDS LPLVDTHSKR TLLIKTVETR DGQVINETSQ HHDDLE ``` | | |
| Evidence | Conclusive | | |
| Peptide list | 91, 210, 289, 342\*\*, 375, 403, 791, 893, 1410\*\*, 1431 | | |
| Peptides | 91 | Confidence | Green |
| Runs | 1, 2, 4 |
| Relation | Unique |
| Proteins | VIME\_HUMAN |
| Sequence | ``` QDVDNASLAR ``` |
| Position | 208 |
| PTMs | none |
| 210 | Confidence | Green |
| Runs | 1, 2, 3, 4 |
| Relation | Unique |
| Proteins | VIME\_HUMAN |
| Sequence | ``` DGQVINETSQHHDDLE ``` |
| Position | 451 |
| PTMs | none |
| 289 | Confidence | Green |
| Runs | 1, 2, 3, 4 |
| Relation | Unique |
| Proteins | VIME\_HUMAN |
| Sequence | ``` FADLSEAANR ``` |
| Position | 295 |
| PTMs | none |
| 342\*\* | Confidence | Green |
| Runs | 1, 2, 3, 4 |
| Relation | NonDiscriminating |
| Proteins | VIME\_HUMAN, DESM\_HUMAN |
| Sequence | ``` VELQELNDR ``` |
| Position | 105 |
| PTMs | none |
| 375 | Confidence | Green |
| Runs | 1, 2, 3, 4 |
| Relation | Unique |
| Proteins | VIME\_HUMAN |
| Sequence | ``` SLYASSPGGVYATR ``` |
| Position | 51 |
| PTMs | none |
| 403 | Confidence | Green |
| Runs | 1, 2, 3 |
| Relation | Unique |
| Proteins | VIME\_HUMAN |
| Sequence | ``` EEAENTLQSFR ``` |
| Position | 197 |
| PTMs | none |
| 791 | Confidence | Green |
| Runs | 1, 2, 3, 4 |
| Relation | Unique |
| Proteins | VIME\_HUMAN |
| Sequence | ``` KVESLQEEIAFLK ``` |
| Position | 223 |
| PTMs | none |
| 893 | Confidence | Green |
| Runs | 1, 2, 3, 4 |
| Relation | Unique |
| Proteins | VIME\_HUMAN |
| Sequence | ``` ILLAELEQLK ``` |
| Position | 130 |
| PTMs | none |
| 1410\*\* | Confidence | Green |
| Runs | 2, 3, 4 |
| Relation | NonDiscriminating |
| Proteins | VIME\_HUMAN, DESM\_HUMAN |
| Sequence | ``` EYQDLLNVK ``` |
| Position | 382 |
| PTMs | none |
| 1431 | Confidence | Green |
| Runs | 2, 3, 4 |
| Relation | Unique |
| Proteins | VIME\_HUMAN |
| Sequence | ``` ETNLDSLPLVDTHSK ``` |
| Position | 425 |
| PTMs | none |

  

Protein P13639

| Name | EF2\_HUMAN | | |
| Description | Elongation factor 2 OS Homo sapiens GN EEF2 PE 1 SV 4 | | |
| Sequence | ```   1 MVNFTVDQIR AIMDKKANIR NMSVIAHVDH GKSTLTDSLV CKAGIIASAR AGETRFTDTR KDEQERCITI KSTAISLFYE LSENDLNFIK QSKDGAGFLI 101 NLIDSPGHVD FSSEVTAALR VTDGALVVVD CVSGVCVQTE TVLRQAIAER IKPVLMMNKM DRALLELQLE PEELYQTFQR IVENVNVIIS TYGEGESGPM 201 GNIMIDPVLG TVGFGSGLHG WAFTLKQFAE MYVAKFAAKG EGQLGPAERA KKVEDMMKKL WGDRYFDPAN GKFSKSATSP EGKKLPRTFC QLILDPIFKV 301 FDAIMNFKKE ETAKLIEKLD IKLDSEDKDK EGKPLLKAVM RRWLPAGDAL LQMITIHLPS PVTAQKYRCE LLYEGPPDDE AAMGIKSCDP KGPLMMYISK 401 MVPTSDKGRF YAFGRVFSGL VSTGLKVRIM GPNYTPGKKE DLYLKPIQRT ILMMGRYVEP IEDVPCGNIV GLVGVDQFLV KTGTITTFEH AHNMRVMKFS 501 VSPVVRVAVE AKNPADLPKL VEGLKRLAKS DPMVQCIIEE SGEHIIAGAG ELHLEICLKD LEEDHACIPI KKSDPVVSYR ETVSEESNVL CLSKSPNKHN 601 RLYMKARPFP DGLAEDIDKG EVSARQELKQ RARYLAEKYE WDVAEARKIW CFGPDGTGPN ILTDITKGVQ YLNEIKDSVV AGFQWATKEG ALCEENMRGV 701 RFDVHDVTLH ADAIHRGGGQ IIPTARRCLY ASVLTAQPRL MEPIYLVEIQ CPEQVVGGIY GVLNRKRGHV FEESQVAGTP MFVVKAYLPV NESFGFTADL 801 RSNTGGQAFP QCVFDHWQIL PGDPFDNSSR PSQVVAETRK RKGLKEGIPA LDNFLDKL ``` | | |
| Evidence | Conclusive | | |
| Peptide list | 60, 165, 233\*\*, 307, 401, 416, 419, 462, 520, 530, 604, 946, 1084, 1152, 1599 | | |
| Peptides | 60 | Confidence | Green |
| Runs | 1, 2, 3, 4 |
| Relation | Unique |
| Proteins | EF2\_HUMAN |
| Sequence | ``` GEGQLGPAER ``` |
| Position | 240 |
| PTMs | none |
| 165 | Confidence | Green |
| Runs | 1, 2, 3, 4 |
| Relation | Unique |
| Proteins | EF2\_HUMAN |
| Sequence | ``` SDPVVSYR ``` |
| Position | 573 |
| PTMs | none |
| 233\*\* | Confidence | Green |
| Runs | 1, 2, 3, 4 |
| Relation | NonDiscriminating |
| Proteins | EF2\_HUMAN, U5S1\_HUMAN |
| Sequence | ``` GGGQIIPTAR ``` |
| Position | 717 |
| PTMs | none |
| 307 | Confidence | Green |
| Runs | 1, 2, 4 |
| Relation | Unique |
| Proteins | EF2\_HUMAN |
| Sequence | ``` KEDLYLKPIQR ``` |
| Position | 439 |
| PTMs | none |
| 401 | Confidence | Green |
| Runs | 1, 2, 3 |
| Relation | Unique |
| Proteins | EF2\_HUMAN |
| Sequence | ``` STLTDSLVCK ``` |
| Position | 33 |
| PTMs | Carbamidomethyl+C(9) |
| 416 | Confidence | Green |
| Runs | 1, 2, 3, 4 |
| Relation | Unique |
| Proteins | EF2\_HUMAN |
| Sequence | ``` EDLYLKPIQR ``` |
| Position | 440 |
| PTMs | Variant #1: none Variant #2: Deamidation+Q(9) |
| 419 | Confidence | Green |
| Runs | 1, 2, 3, 4 |
| Relation | Unique |
| Proteins | EF2\_HUMAN |
| Sequence | ``` FSVSPVVR ``` |
| Position | 499 |
| PTMs | none |
| 462 | Confidence | Green |
| Runs | 1, 2, 3, 4 |
| Relation | Unique |
| Proteins | EF2\_HUMAN |
| Sequence | ``` GVQYLNEIK ``` |
| Position | 668 |
| PTMs | none |
| 520 | Confidence | Green |
| Runs | 1, 2, 3, 4 |
| Relation | Unique |
| Proteins | EF2\_HUMAN |
| Sequence | ``` VNFTVDQIR ``` |
| Position | 2 |
| PTMs | none |
| 530 | Confidence | Green |
| Runs | 1, 2, 4 |
| Relation | Unique |
| Proteins | EF2\_HUMAN |
| Sequence | ``` ETVSEESNVLCLSK ``` |
| Position | 581 |
| PTMs | Carbamidomethyl+C(11) |
| 604 | Confidence | Green |
| Runs | 1, 2, 3, 4 |
| Relation | Unique |
| Proteins | EF2\_HUMAN |
| Sequence | ``` CLYASVLTAQPR ``` |
| Position | 728 |
| PTMs | Carbamidomethyl+C(1) |
| 946 | Confidence | Green |
| Runs | 1, 2, 3 |
| Relation | Unique |
| Proteins | EF2\_HUMAN |
| Sequence | ``` AYLPVNESFGFTADLR ``` |
| Position | 786 |
| PTMs | none |
| 1084 | Confidence | Green |
| Runs | 1, 2, 3, 4 |
| Relation | Unique |
| Proteins | EF2\_HUMAN |
| Sequence | ``` TFCQLILDPIFK ``` |
| Position | 288 |
| PTMs | Carbamidomethyl+C(3) |
| 1152 | Confidence | Green |
| Runs | 1, 2, 3, 4 |
| Relation | Unique |
| Proteins | EF2\_HUMAN |
| Sequence | ``` LMEPIYLVEIQCPEQVVGGIYGVLNR ``` |
| Position | 740 |
| PTMs | Carbamidomethyl+C(12) |
| 1599 | Confidence | Green |
| Runs | 2, 3, 4 |
| Relation | Unique |
| Proteins | EF2\_HUMAN |
| Sequence | ``` EGIPALDNFLDK ``` |
| Position | 846 |
| PTMs | none |

  

Protein P04075

| Name | ALDOA\_HUMAN | | |
| Description | Fructose bisphosphate aldolase A OS Homo sapiens GN ALDOA PE 1 SV 2 | | |
| Sequence | ```   1 MPYQYPALTP EQKKELSDIA HRIVAPGKGI LAADESTGSI AKRLQSIGTE NTEENRRFYR QLLLTADDRV NPCIGGVILF HETLYQKADD GRPFPQVIKS 101 KGGVVGIKVD KGVVPLAGTN GETTTQGLDG LSERCAQYKK DGADFAKWRC VLKIGEHTPS ALAIMENANV LARYASICQQ NGIVPIVEPE ILPDGDHDLK 201 RCQYVTEKVL AAVYKALSDH HIYLEGTLLK PNMVTPGHAC TQKFSHEEIA MATVTALRRT VPPAVTGITF LSGGQSEEEA SINLNAINKC PLLKPWALTF 301 SYGRALQASA LKAWGGKKEN LKAAQEEYVK RALANSLACQ GKYTPSGQAG AAASESLFVS NHAY ``` | | |
| Evidence | Conclusive | | |
| Peptide list | 28, 100, 106, 132, 137, 385, 418, 527, 899, 1249 | | |
| Peptides | 28 | Confidence | Green |
| Runs | 1, 2, 3, 4 |
| Relation | Unique |
| Proteins | ALDOA\_HUMAN |
| Sequence | ``` AAQEEYVK ``` |
| Position | 323 |
| PTMs | none |
| 100 | Confidence | Green |
| Runs | 1, 2, 3 |
| Relation | Unique |
| Proteins | ALDOA\_HUMAN |
| Sequence | ``` LQSIGTENTEENRR ``` |
| Position | 44 |
| PTMs | none |
| 106 | Confidence | Green |
| Runs | 1, 2, 3, 4 |
| Relation | Unique |
| Proteins | ALDOA\_HUMAN |
| Sequence | ``` ELSDIAHR ``` |
| Position | 15 |
| PTMs | none |
| 132 | Confidence | Green |
| Runs | 1, 2, 3 |
| Relation | Unique |
| Proteins | ALDOA\_HUMAN |
| Sequence | ``` ALQASALK ``` |
| Position | 305 |
| PTMs | none |
| 137 | Confidence | Green |
| Runs | 1, 2, 3, 4 |
| Relation | Unique |
| Proteins | ALDOA\_HUMAN |
| Sequence | ``` LQSIGTENTEENR ``` |
| Position | 44 |
| PTMs | none |
| 385 | Confidence | Green |
| Runs | 1, 2, 3, 4 |
| Relation | Unique |
| Proteins | ALDOA\_HUMAN |
| Sequence | ``` GILAADESTGSIAK ``` |
| Position | 29 |
| PTMs | none |
| 418 | Confidence | Green |
| Runs | 1, 2, 3, 4 |
| Relation | Unique |
| Proteins | ALDOA\_HUMAN |
| Sequence | ``` QLLLTADDR ``` |
| Position | 61 |
| PTMs | none |
| 527 | Confidence | Green |
| Runs | 1, 2, 4 |
| Relation | Unique |
| Proteins | ALDOA\_HUMAN |
| Sequence | ``` YTPSGQAGAAASESLFVSNHAY ``` |
| Position | 343 |
| PTMs | none |
| 899 | Confidence | Green |
| Runs | 1, 2, 4 |
| Relation | Unique |
| Proteins | ALDOA\_HUMAN |
| Sequence | ``` YASICQQNGIVPIVEPEILPDGDHDLK ``` |
| Position | 174 |
| PTMs | Carbamidomethyl+C(5) Deamidation+N(8) |
| 1249 | Confidence | Green |
| Runs | 2, 3, 4 |
| Relation | Unique |
| Proteins | ALDOA\_HUMAN |
| Sequence | ``` ALANSLACQGK ``` |
| Position | 332 |
| PTMs | Carbamidomethyl+C(8) |

  

Protein P18669

| Name | PGAM1\_HUMAN | | |
| Description | Phosphoglycerate mutase 1 OS Homo sapiens GN PGAM1 PE 1 SV 2 | | |
| Sequence | ```   1 MAAYKLVLIR HGESAWNLEN RFSGWYDADL SPAGHEEAKR GGQALRDAGY EFDICFTSVQ KRAIRTLWTV LDAIDQMWLP VVRTWRLNER HYGGLTGLNK 101 AETAAKHGEA QVKIWRRSYD VPPPPMEPDH PFYSNISKDR RYADLTEDQL PSCESLKDTI ARALPFWNEE IVPQIKEGKR VLIAAHGNSL RGIVKHLEGL 201 SEEAIMELNL PTGIPIVYEL DKNLKPIKPM QFLGDEETVR KAMEAVAAQG KAKK ``` | | |
| Evidence | Conclusive | | |
| Peptide list | 179\*\*, 271, 633, 1022\*\*, 1194\*\* | | |
| Peptides | 179\*\* | Confidence | Green |
| Runs | 1, 2, 3, 4 |
| Relation | NonDiscriminating |
| Proteins | PGAM1\_HUMAN, PGAM4\_HUMAN, PGAM2\_HUMAN |
| Sequence | ``` HYGGLTGLNK ``` |
| Position | 91 |
| PTMs | none |
| 271 | Confidence | Green |
| Runs | 1, 2, 4 |
| Relation | Unique |
| Proteins | PGAM1\_HUMAN |
| Sequence | ``` HGESAWNLENR ``` |
| Position | 11 |
| PTMs | Variant #1: none Variant #2: Deamidation+N(10) |
| 633 | Confidence | Green |
| Runs | 1, 2, 3, 4 |
| Relation | Unique |
| Proteins | PGAM1\_HUMAN |
| Sequence | ``` FSGWYDADLSPAGHEEAK ``` |
| Position | 22 |
| PTMs | none |
| 1022\*\* | Confidence | Green |
| Runs | 1, 2, 3, 4 |
| Relation | NonDiscriminating |
| Proteins | PGAM1\_HUMAN, PGAM4\_HUMAN, PGAM2\_HUMAN |
| Sequence | ``` ALPFWNEEIVPQIK ``` |
| Position | 163 |
| PTMs | none |
| 1194\*\* | Confidence | Green |
| Runs | 2, 3, 4 |
| Relation | NonDiscriminating |
| Proteins | PGAM1\_HUMAN, PGAM2\_HUMAN |
| Sequence | ``` AMEAVAAQGK ``` |
| Position | 242 |
| PTMs | none |

  

Protein P11021

| Name | GRP78\_HUMAN | | |
| Description | 78 kDa glucose regulated protein OS Homo sapiens GN HSPA5 PE 1 SV 2 | | |
| Sequence | ```   1 MKLSLVAAML LLLSAARAEE EDKKEDVGTV VGIDLGTTYS CVGVFKNGRV EIIANDQGNR ITPSYVAFTP EGERLIGDAA KNQLTSNPEN TVFDAKRLIG 101 RTWNDPSVQQ DIKFLPFKVV EKKTKPYIQV DIGGGQTKTF APEEISAMVL TKMKETAEAY LGKKVTHAVV TVPAYFNDAQ RQATKDAGTI AGLNVMRIIN 201 EPTAAAIAYG LDKREGEKNI LVFDLGGGTF DVSLLTIDNG VFEVVATNGD THLGGEDFDQ RVMEHFIKLY KKKTGKDVRK DNRAVQKLRR EVEKAKRALS 301 SQHQARIEIE SFYEGEDFSE TLTRAKFEEL NMDLFRSTMK PVQKVLEDSD LKKSDIDEIV LVGGSTRIPK IQQLVKEFFN GKEPSRGINP DEAVAYGAAV 401 QAGVLSGDQD TGDLVLLDVC PLTLGIETVG GVMTKLIPRN TVVPTKKSQI FSTASDNQPT VTIKVYEGER PLTKDNHLLG TFDLTGIPPA PRGVPQIEVT 501 FEIDVNGILR VTAEDKGTGN KNKITITNDQ NRLTPEEIER MVNDAEKFAE EDKKLKERID TRNELESYAY SLKNQIGDKE KLGGKLSSED KETMEKAVEE 601 KIEWLESHQD ADIEDFKAKK KELEEIVQPI ISKLYGSAGP PPTGEEDTAE KDEL ``` | | |
| Evidence | Conclusive | | |
| Peptide list | 178\*\*, 499, 606, 607, 625, 715, 749\*\* | | |
| Peptides | 178\*\* | Confidence | Green |
| Runs | 1, 2, 3, 4 |
| Relation | NonDiscriminating |
| Proteins | HSP71\_HUMAN, HSP7C\_HUMAN, HSP7C\_HUMAN, HS71L\_HUMAN, HSP72\_HUMAN, GRP78\_HUMAN |
| Sequence | ``` VEIIANDQGNR ``` |
| Position | 50 |
| PTMs | none |
| 499 | Confidence | Green |
| Runs | 1, 2, 3, 4 |
| Relation | Unique |
| Proteins | GRP78\_HUMAN |
| Sequence | ``` NQLTSNPENTVFDAK ``` |
| Position | 82 |
| PTMs | Variant #1: none Variant #2: Deamidation+N(1) |
| 606 | Confidence | Green |
| Runs | 1, 2, 3 |
| Relation | Unique |
| Proteins | GRP78\_HUMAN |
| Sequence | ``` VTHAVVTVPAYFNDAQR ``` |
| Position | 165 |
| PTMs | Variant #1: none Variant #2: Deamidation+N(13) Deamidation+Q(16) |
| 607 | Confidence | Green |
| Runs | 1, 2, 3 |
| Relation | Unique |
| Proteins | GRP78\_HUMAN |
| Sequence | ``` ITPSYVAFTPEGER ``` |
| Position | 61 |
| PTMs | none |
| 625 | Confidence | Green |
| Runs | 1, 2, 3, 4 |
| Relation | Unique |
| Proteins | GRP78\_HUMAN |
| Sequence | ``` TFAPEEISAMVLTK ``` |
| Position | 139 |
| PTMs | none |
| 715 | Confidence | Green |
| Runs | 1, 2, 3, 4 |
| Relation | Unique |
| Proteins | GRP78\_HUMAN |
| Sequence | ``` ELEEIVQPIISK ``` |
| Position | 622 |
| PTMs | none |
| 749\*\* | Confidence | Green |
| Runs | 1, 2, 3, 4 |
| Relation | NonDiscriminating |
| Proteins | HSP7C\_HUMAN, HSP7C\_HUMAN, HS71L\_HUMAN, HSP72\_HUMAN, GRP78\_HUMAN |
| Sequence | ``` IINEPTAAAIAYGLDK ``` |
| Position | 198 |
| PTMs | none |

  

Protein P05388

| Name | RLA0\_HUMAN | | |
| Description | 60S acidic ribosomal protein P0 OS Homo sapiens GN RPLP0 PE 1 SV 1 | | |
| Sequence | ```   1 MPREDRATWK SNYFLKIIQL LDDYPKCFIV GADNVGSKQM QQIRMSLRGK AVVLMGKNTM MRKAIRGHLE NNPALEKLLP HIRGNVGFVF TKEDLTEIRD 101 MLLANKVPAA ARAGAIAPCE VTVPAQNTGL GPEKTSFFQA LGITTKISRG TIEILSDVQL IKTGDKVGAS EATLLNMLNI SPFSFGLVIQ QVFDNGSIYN 201 PEVLDITEET LHSRFLEGVR NVASVCLQIG YPTVASVPHS IINGYKRVLA LSVETDYTFP LAEKVKAFLA DPSAFVAAAP VAAATTAAPA AAAAPAKVEA 301 KEESEESDED MGFGLFD ``` | | |
| Evidence | Conclusive | | |
| Peptide list | 52\*\*, 553\*\*, 735\*\*, 953, 960\*\*, 976\*\* | | |
| Peptides | 52\*\* | Confidence | Green |
| Runs | 1, 2, 3, 4 |
| Relation | NonDiscriminating |
| Proteins | RLA0\_HUMAN, RLA0L\_HUMAN |
| Sequence | ``` GHLENNPALEK ``` |
| Position | 67 |
| PTMs | Variant #1: none Variant #2: Deamidation+N(5) |
| 553\*\* | Confidence | Green |
| Runs | 1, 2, 4 |
| Relation | NonDiscriminating |
| Proteins | RLA0\_HUMAN, RLA0L\_HUMAN |
| Sequence | ``` AGAIAPCEVTVPAQNTGLGPEK ``` |
| Position | 113 |
| PTMs | Carbamidomethyl+C(7) |
| 735\*\* | Confidence | Green |
| Runs | 1, 2, 3, 4 |
| Relation | NonDiscriminating |
| Proteins | RLA0\_HUMAN, RLA0L\_HUMAN |
| Sequence | ``` IIQLLDDYPK ``` |
| Position | 17 |
| PTMs | none |
| 953 | Confidence | Green |
| Runs | 1, 2, 3, 4 |
| Relation | Unique |
| Proteins | RLA0\_HUMAN |
| Sequence | ``` AFLADPSAFVAAAPVAAATTAAPAAAAAPAK ``` |
| Position | 267 |
| PTMs | none |
| 960\*\* | Confidence | Green |
| Runs | 1, 2, 3, 4 |
| Relation | NonDiscriminating |
| Proteins | RLA0\_HUMAN, RLA0L\_HUMAN |
| Sequence | ``` GTIEILSDVQLIK ``` |
| Position | 150 |
| PTMs | none |
| 976\*\* | Confidence | Green |
| Runs | 1, 2, 3, 4 |
| Relation | NonDiscriminating |
| Proteins | RLA0\_HUMAN, RLA0L\_HUMAN |
| Sequence | ``` TSFFQALGITTK ``` |
| Position | 135 |
| PTMs | none |

  

Protein P26641

| Name | EF1G\_HUMAN | | |
| Description | Elongation factor 1 gamma OS Homo sapiens GN EEF1G PE 1 SV 3 | | |
| Sequence | ```   1 MAAGTLYTYP ENWRAFKALI AAQYSGAQVR VLSAPPHFHF GQTNRTPEFL RKFPAGKVPA FEGDDGFCVF ESNAIAYYVS NEELRGSTPE AAAQVVQWVS 101 FADSDIVPPA STWVFPTLGI MHHNKQATEN AKEEVRRILG LLDAYLKTRT FLVGERVTLA DITVVCTLLW LYKQVLEPSF RQAFPNTNRW FLTCINQPQF 201 RAVLGEVKLC EKMAQFDAKK FAETQPKKDT PRKEKGSREE KQKPQAERKE EKKAAAPAPE EEMDECEQAL AAEPKAKDPF AHLPKSTFVL DEFKRKYSNE 301 DTLSVALPYF WEHFDKDGWS LWYSEYRFPE ELTQTFMSCN LITGMFQRLD KLRKNAFASV ILFGTNNSSS ISGVWVFRGQ ELAFPLSPDW QVDYESYTWR 401 KLDPGSEETQ TLVREYFSWE GAFQHVGKAF NQGKIFK ``` | | |
| Evidence | Conclusive | | |
| Peptide list | 295, 357, 364, 417, 460, 739, 822, 925, 1015 | | |
| Peptides | 295 | Confidence | Green |
| Runs | 1, 2, 3, 4 |
| Relation | Unique |
| Proteins | EF1G\_HUMAN |
| Sequence | ``` KLDPGSEETQTLVR ``` |
| Position | 401 |
| PTMs | Variant #1: none Variant #2: Deamidation+Q(10) |
| 357 | Confidence | Green |
| Runs | 1, 2, 3, 4 |
| Relation | Unique |
| Proteins | EF1G\_HUMAN |
| Sequence | ``` TFLVGER ``` |
| Position | 150 |
| PTMs | none |
| 364 | Confidence | Green |
| Runs | 1, 3, 4 |
| Relation | Unique |
| Proteins | EF1G\_HUMAN |
| Sequence | ``` LDPGSEETQTLVR ``` |
| Position | 402 |
| PTMs | none |
| 417 | Confidence | Green |
| Runs | 1, 2, 3, 4 |
| Relation | Unique |
| Proteins | EF1G\_HUMAN |
| Sequence | ``` ALIAAQYSGAQVR ``` |
| Position | 18 |
| PTMs | none |
| 460 | Confidence | Green |
| Runs | 1, 2, 3 |
| Relation | Unique |
| Proteins | EF1G\_HUMAN |
| Sequence | ``` VLSAPPHFHFGQTNR ``` |
| Position | 31 |
| PTMs | none |
| 739 | Confidence | Green |
| Runs | 1, 2, 3, 4 |
| Relation | Unique |
| Proteins | EF1G\_HUMAN |
| Sequence | ``` STFVLDEFK ``` |
| Position | 286 |
| PTMs | none |
| 822 | Confidence | Green |
| Runs | 1, 2, 3, 4 |
| Relation | Unique |
| Proteins | EF1G\_HUMAN |
| Sequence | ``` EYFSWEGAFQHVGK ``` |
| Position | 415 |
| PTMs | none |
| 925 | Confidence | Green |
| Runs | 1, 2, 3 |
| Relation | Unique |
| Proteins | EF1G\_HUMAN |
| Sequence | ``` WFLTCINQPQFR ``` |
| Position | 190 |
| PTMs | Carbamidomethyl+C(5) |
| 1015 | Confidence | Green |
| Runs | 1, 2, 3, 4 |
| Relation | Unique |
| Proteins | EF1G\_HUMAN |
| Sequence | ``` ILGLLDAYLK ``` |
| Position | 138 |
| PTMs | none |

  

Protein P10599

| Name | THIO\_HUMAN | | |
| Description | Thioredoxin OS Homo sapiens GN TXN PE 1 SV 3 | | |
| Sequence | ```   1 MVKQIESKTA FQEALDAAGD KLVVVDFSAT WCGPCKMIKP FFHSLSEKYS NVIFLEVDVD DCQDVASECE VKCMPTFQFF KKGQKVGEFS GANKEKLEAT 101 INELV ``` | | |
| Evidence | Conclusive | | |
| Peptide list | 48, 600 | | |
| Peptides | 48 | Confidence | Green |
| Runs | 1, 2, 3, 4 |
| Relation | Unique |
| Proteins | THIO\_HUMAN |
| Sequence | ``` VGEFSGANK ``` |
| Position | 86 |
| PTMs | none |
| 600 | Confidence | Green |
| Runs | 1, 2, 3, 4 |
| Relation | Unique |
| Proteins | THIO\_HUMAN |
| Sequence | ``` TAFQEALDAAGDK ``` |
| Position | 9 |
| PTMs | none |

  

Protein P00558

| Name | PGK1\_HUMAN | | |
| Description | Phosphoglycerate kinase 1 OS Homo sapiens GN PGK1 PE 1 SV 3 | | |
| Sequence | ```   1 MSLSNKLTLD KLDVKGKRVV MRVDFNVPMK NNQITNNQRI KAAVPSIKFC LDNGAKSVVL MSHLGRPDGV PMPDKYSLEP VAVELKSLLG KDVLFLKDCV 101 GPEVEKACAN PAAGSVILLE NLRFHVEEEG KGKDASGNKV KAEPAKIEAF RASLSKLGDV YVNDAFGTAH RAHSSMVGVN LPQKAGGFLM KKELNYFAKA 201 LESPERPFLA ILGGAKVADK IQLINNMLDK VNEMIIGGGM AFTFLKVLNN MEIGTSLFDE EGAKIVKDLM SKAEKNGVKI TLPVDFVTAD KFDENAKTGQ 301 ATVASGIPAG WMGLDCGPES SKKYAEAVTR AKQIVWNGPV GVFEWEAFAR GTKALMDEVV KATSRGCITI IGGGDTATCC AKWNTEDKVS HVSTGGGASL 401 ELLEGKVLPG VDALSNI ``` | | |
| Evidence | Conclusive | | |
| Peptide list | 53\*\*, 318, 594, 863, 885, 929, 934 | | |
| Peptides | 53\*\* | Confidence | Green |
| Runs | 1, 2, 3, 4 |
| Relation | NonDiscriminating |
| Proteins | PGK1\_HUMAN, PGK2\_HUMAN |
| Sequence | ``` FHVEEEGK ``` |
| Position | 124 |
| PTMs | none |
| 318 | Confidence | Green |
| Runs | 1, 2, 4 |
| Relation | Unique |
| Proteins | PGK1\_HUMAN |
| Sequence | ``` AHSSMVGVNLPQK ``` |
| Position | 172 |
| PTMs | none |
| 594 | Confidence | Green |
| Runs | 1, 2, 3, 4 |
| Relation | Unique |
| Proteins | PGK1\_HUMAN |
| Sequence | ``` YSLEPVAVELK ``` |
| Position | 76 |
| PTMs | none |
| 863 | Confidence | Green |
| Runs | 1, 2, 3, 4 |
| Relation | Unique |
| Proteins | PGK1\_HUMAN |
| Sequence | ``` ITLPVDFVTADK ``` |
| Position | 280 |
| PTMs | none |
| 885 | Confidence | Green |
| Runs | 1, 2, 3, 4 |
| Relation | Unique |
| Proteins | PGK1\_HUMAN |
| Sequence | ``` ITLPVDFVTADKFDENAK ``` |
| Position | 280 |
| PTMs | none |
| 929 | Confidence | Green |
| Runs | 1, 2, 3, 4 |
| Relation | Unique |
| Proteins | PGK1\_HUMAN |
| Sequence | ``` ACANPAAGSVILLENLR ``` |
| Position | 107 |
| PTMs | Carbamidomethyl+C(2) |
| 934 | Confidence | Green |
| Runs | 1, 2, 3, 4 |
| Relation | Unique |
| Proteins | PGK1\_HUMAN |
| Sequence | ``` ALESPERPFLAILGGAK ``` |
| Position | 200 |
| PTMs | none |

  

Protein P23528

| Name | COF1\_HUMAN | | |
| Description | Cofilin 1 OS Homo sapiens GN CFL1 PE 1 SV 3 | | |
| Sequence | ```   1 MASGVAVSDG VIKVFNDMKV RKSSTPEEVK KRKKAVLFCL SEDKKNIILE EGKEILVGDV GQTVDDPYAT FVKMLPDKDC RYALYDATYE TKESKKEDLV 101 FIFWAPESAP LKSKMIYASS KDAIKKKLTG IKHELQANCY EEVKDRCTLA EKLGGSAVIS LEGKPL ``` | | |
| Evidence | Conclusive | | |
| Peptide list | 910 | | |
| Peptides | 910 | Confidence | Green |
| Runs | 1, 2, 4 |
| Relation | Unique |
| Proteins | COF1\_HUMAN |
| Sequence | ``` EILVGDVGQTVDDPYATFVK ``` |
| Position | 54 |
| PTMs | none |

  

Protein O43175

| Name | SERA\_HUMAN | | |
| Description | D 3 phosphoglycerate dehydrogenase OS Homo sapiens GN PHGDH PE 1 SV 4 | | |
| Sequence | ```   1 MAFANLRKVL ISDSLDPCCR KILQDGGLQV VEKQNLSKEE LIAELQDCEG LIVRSATKVT ADVINAAEKL QVVGRAGTGV DNVDLEAATR KGILVMNTPN 101 GNSLSAAELT CGMIMCLARQ IPQATASMKD GKWERKKFMG TELNGKTLGI LGLGRIGREV ATRMQSFGMK TIGYDPIISP EVSASFGVQQ LPLEEIWPLC 201 DFITVHTPLL PSTTGLLNDN TFAQCKKGVR VVNCARGGIV DEGALLRALQ SGQCAGAALD VFTEEPPRDR ALVDHENVIS CPHLGASTKE AQSRCGEEIA 301 VQFVDMVKGK SLTGVVNAQA LTSAFSPHTK PWIGLAEALG TLMRAWAGSP KGTIQVITQG TSLKNAGNCL SPAVIVGLLK EASKQADVNL VNAKLLVKEA 401 GLNVTTSHSP AAPGEQGFGE CLLAVALAGA PYQAVGLVQG TTPVLQGLNG AVFRPEVPLR RDLPLLLFRT QTSDPAMLPT MIGLLAEAGV RLLSYQTSLV 501 SDGETWHVMG ISSLLPSLEA WKQHVTEAFQ FHF ``` | | |
| Evidence | Conclusive | | |
| Peptide list | 410, 521, 745, 1040, 1065, 1120, 1261 | | |
| Peptides | 410 | Confidence | Green |
| Runs | 1, 3, 4 |
| Relation | Unique |
| Proteins | SERA\_HUMAN |
| Sequence | ``` AGTGVDNVDLEAATR ``` |
| Position | 76 |
| PTMs | none |
| 521 | Confidence | Green |
| Runs | 1, 2, 3, 4 |
| Relation | Unique |
| Proteins | SERA\_HUMAN |
| Sequence | ``` GTIQVITQGTSLK ``` |
| Position | 352 |
| PTMs | none |
| 745 | Confidence | Green |
| Runs | 1, 2, 3, 4 |
| Relation | Unique |
| Proteins | SERA\_HUMAN |
| Sequence | ``` TLGILGLGR ``` |
| Position | 147 |
| PTMs | none |
| 1040 | Confidence | Green |
| Runs | 1, 2, 3, 4 |
| Relation | Unique |
| Proteins | SERA\_HUMAN |
| Sequence | ``` NAGNCLSPAVIVGLLK ``` |
| Position | 365 |
| PTMs | Carbamidomethyl+C(5) |
| 1065 | Confidence | Green |
| Runs | 1, 2, 3, 4 |
| Relation | Unique |
| Proteins | SERA\_HUMAN |
| Sequence | ``` DLPLLLFR ``` |
| Position | 462 |
| PTMs | none |
| 1120 | Confidence | Green |
| Runs | 1, 2, 3, 4 |
| Relation | Unique |
| Proteins | SERA\_HUMAN |
| Sequence | ``` ALQSGQCAGAALDVFTEEPPRDR ``` |
| Position | 248 |
| PTMs | Carbamidomethyl+C(7) |
| 1261 | Confidence | Green |
| Runs | 2, 3, 4 |
| Relation | Unique |
| Proteins | SERA\_HUMAN |
| Sequence | ``` QADVNLVNAK ``` |
| Position | 385 |
| PTMs | Variant #1: none Variant #2: Deamidation+Q(1) |

  

Protein P30041

| Name | PRDX6\_HUMAN | | |
| Description | Peroxiredoxin 6 OS Homo sapiens GN PRDX6 PE 1 SV 3 | | |
| Sequence | ```   1 MPGGLLLGDV APNFEANTTV GRIRFHDFLG DSWGILFSHP RDFTPVCTTE LGRAAKLAPE FAKRNVKLIA LSIDSVEDHL AWSKDINAYN CEEPTEKLPF 101 PIIDDRNREL AILLGMLDPA EKDEKGMPVT ARVVFVFGPD KKLKLSILYP ATTGRNFDEI LRVVISLQLT AEKRVATPVD WKDGDSVMVL PTIPEEEAKK 201 LFPKGVFTKE LPSGKKYLRY TPQP ``` | | |
| Evidence | Conclusive | | |
| Peptide list | 544, 611, 695, 805, 913 | | |
| Peptides | 544 | Confidence | Green |
| Runs | 1, 2, 4 |
| Relation | Unique |
| Proteins | PRDX6\_HUMAN |
| Sequence | ``` DFTPVCTTELGR ``` |
| Position | 42 |
| PTMs | Carbamidomethyl+C(6) |
| 611 | Confidence | Green |
| Runs | 1, 2, 3, 4 |
| Relation | Unique |
| Proteins | PRDX6\_HUMAN |
| Sequence | ``` LSILYPATTGR ``` |
| Position | 145 |
| PTMs | none |
| 695 | Confidence | Green |
| Runs | 1, 2, 3, 4 |
| Relation | Unique |
| Proteins | PRDX6\_HUMAN |
| Sequence | ``` VVFVFGPDK ``` |
| Position | 133 |
| PTMs | none |
| 805 | Confidence | Green |
| Runs | 1, 3, 4 |
| Relation | Unique |
| Proteins | PRDX6\_HUMAN |
| Sequence | ``` LPFPIIDDR ``` |
| Position | 98 |
| PTMs | none |
| 913 | Confidence | Green |
| Runs | 1, 2, 3 |
| Relation | Unique |
| Proteins | PRDX6\_HUMAN |
| Sequence | ``` PGGLLLGDVAPNFEANTTVGR ``` |
| Position | 2 |
| PTMs | Variant #1: none Variant #2: Acetyl+N-TERM(1) |

  

Protein Q15366

| Name | PCBP2\_HUMAN | | |
| Description | Poly rC binding protein 2 OS Homo sapiens GN PCBP2 PE 1 SV 1 | | |
| Sequence | ```   1 MDTGVIEGGL NVTLTIRLLM HGKEVGSIIG KKGESVKKMR EESGARINIS EGNCPERIIT LAGPTNAIFK AFAMIIDKLE EDISSSMTNS TAASRPPVTL 101 RLVVPASQCG SLIGKGGCKI KEIRESTGAQ VQVAGDMLPN STERAITIAG IPQSIIECVK QICVVMLETL SQSPPKGVTI PYRPKPSSSP VIFAGGQDRY 201 STGSDSASFP HTTPSMCLNP DLEGPPLEAY TIQGQYAIPQ PDLTKLHQLA MQQSHFPMTH GNTGFSGIES SSPEVKGYWG LDASAQTTSH ELTIPNDLIG 301 CIIGRQGAKI NEIRQMSGAQ IKIANPVEGS TDRQVTITGS AASISLAQYL INVRLSSETG GMGSS ``` | | |
| Evidence | Conclusive | | |
| Peptide list | 173\*\*, 526\*\*, 850, 1004 | | |
| Peptides | 173\*\* | Confidence | Green |
| Runs | 1, 2, 3, 4 |
| Relation | NonDiscriminating |
| Proteins | PCBP2\_HUMAN, PCBP1\_HUMAN, PCBP3\_HUMAN, PCBP3\_HUMAN, PCBP3\_HUMAN, PCBP3\_HUMAN, PCBP3\_HUMAN |
| Sequence | ``` INISEGNCPER ``` |
| Position | 47 |
| PTMs | Carbamidomethyl+C(8) |
| 526\*\* | Confidence | Green |
| Runs | 1, 2, 3 |
| Relation | NonDiscriminating |
| Proteins | PCBP2\_HUMAN, PCBP3\_HUMAN, PCBP3\_HUMAN, PCBP3\_HUMAN, PCBP3\_HUMAN, PCBP3\_HUMAN |
| Sequence | ``` LVVPASQCGSLIGK ``` |
| Position | 102 |
| PTMs | Carbamidomethyl+C(8) |
| 850 | Confidence | Green |
| Runs | 1, 2, 3, 4 |
| Relation | Unique |
| Proteins | PCBP2\_HUMAN |
| Sequence | ``` IITLAGPTNAIFK ``` |
| Position | 58 |
| PTMs | none |
| 1004 | Confidence | Green |
| Runs | 1, 2, 3, 4 |
| Relation | Unique |
| Proteins | PCBP2\_HUMAN |
| Sequence | ``` AITIAGIPQSIIECVK ``` |
| Position | 145 |
| PTMs | Carbamidomethyl+C(14) |

  

Protein P09211

| Name | GSTP1\_HUMAN | | |
| Description | Glutathione S transferase P OS Homo sapiens GN GSTP1 PE 1 SV 2 | | |
| Sequence | ```   1 MPPYTVVYFP VRGRCAALRM LLADQGQSWK EEVVTVETWQ EGSLKASCLY GQLPKFQDGD LTLYQSNTIL RHLGRTLGLY GKDQQEAALV DMVNDGVEDL 101 RCKYISLIYT NYEAGKDDYV KALPGQLKPF ETLLSQNQGG KTFIVGDQIS FADYNLLDLL LIHEVLAPGC LDAFPLLSAY VGRLSARPKL KAFLASPEYV 201 NLPINGNGKQ ``` | | |
| Evidence | Conclusive | | |
| Peptide list | 878 | | |
| Peptides | 878 | Confidence | Green |
| Runs | 1, 2, 3 |
| Relation | Unique |
| Proteins | GSTP1\_HUMAN |
| Sequence | ``` ALPGQLKPFETLLSQNQGGK ``` |
| Position | 122 |
| PTMs | Variant #1: none Variant #2: Deamidation+Q(5) |

  

Protein P14625

| Name | ENPL\_HUMAN | | |
| Description | Endoplasmin OS Homo sapiens GN HSP90B1 PE 1 SV 1 | | |
| Sequence | ```   1 MRALWVLGLC CVLLTFGSVR ADDEVDVDGT VEEDLGKSRE GSRTDDEVVQ REEEAIQLDG LNASQIRELR EKSEKFAFQA EVNRMMKLII NSLYKNKEIF 101 LRELISNASD ALDKIRLISL TDENALSGNE ELTVKIKCDK EKNLLHVTDT GVGMTREELV KNLGTIAKSG TSEFLNKMTE AQEDGQSTSE LIGQFGVGFY 201 SAFLVADKVI VTSKHNNDTQ HIWESDSNEF SVIADPRGNT LGRGTTITLV LKEEASDYLE LDTIKNLVKK YSQFINFPIY VWSSKTETVE EPMEEEEAAK 301 EEKEESDDEA AVEEEEEEKK PKTKKVEKTV WDWELMNDIK PIWQRPSKEV EEDEYKAFYK SFSKESDDPM AYIHFTAEGE VTFKSILFVP TSAPRGLFDE 401 YGSKKSDYIK LYVRRVFITD DFHDMMPKYL NFVKGVVDSD DLPLNVSRET LQQHKLLKVI RKKLVRKTLD MIKKIADDKY NDTFWKEFGT NIKLGVIEDH 501 SNRTRLAKLL RFQSSHHPTD ITSLDQYVER MKEKQDKIYF MAGSSRKEAE SSPFVERLLK KGYEVIYLTE PVDEYCIQAL PEFDGKRFQN VAKEGVKFDE 601 SEKTKESREA VEKEFEPLLN WMKDKALKDK IEKAVVSQRL TESPCALVAS QYGWSGNMER IMKAQAYQTG KDISTNYYAS QKKTFEINPR HPLIRDMLRR 701 IKEDEDDKTV LDLAVVLFET ATLRSGYLLP DTKAYGDRIE RMLRLSLNID PDAKVEEEPE EEPEETAEDT TEDTEQDEDE EMDVGTDEEE ETAKESTAEK 801 DEL ``` | | |
| Evidence | Conclusive | | |
| Peptide list | 32, 404, 432\*\*, 571, 694 | | |
| Peptides | 32 | Confidence | Green |
| Runs | 1, 3, 4 |
| Relation | Unique |
| Proteins | ENPL\_HUMAN |
| Sequence | ``` EVEEDEYK ``` |
| Position | 349 |
| PTMs | none |
| 404 | Confidence | Green |
| Runs | 1, 2, 4 |
| Relation | Unique |
| Proteins | ENPL\_HUMAN |
| Sequence | ``` SGYLLPDTK ``` |
| Position | 725 |
| PTMs | none |
| 432\*\* | Confidence | Green |
| Runs | 1, 2, 3, 4 |
| Relation | NonDiscriminating |
| Proteins | HS90B\_HUMAN, H90B3\_HUMAN, H90B2\_HUMAN, H90B4\_HUMAN, ENPL\_HUMAN |
| Sequence | ``` ELISNASDALDK ``` |
| Position | 103 |
| PTMs | none |
| 571 | Confidence | Green |
| Runs | 1, 2, 3 |
| Relation | Unique |
| Proteins | ENPL\_HUMAN |
| Sequence | ``` FQSSHHPTDITSLDQYVER ``` |
| Position | 512 |
| PTMs | none |
| 694 | Confidence | Green |
| Runs | 1, 2, 3, 4 |
| Relation | Unique |
| Proteins | ENPL\_HUMAN |
| Sequence | ``` SILFVPTSAPR ``` |
| Position | 385 |
| PTMs | none |

  

Protein P14618

| Name | KPYM\_HUMAN | | |
| Description | Pyruvate kinase isozymes M1 M2 OS Homo sapiens GN PKM2 PE 1 SV 4 | | |
| Sequence | ```   1 MSKPHSEAGT AFIQTQQLHA AMADTFLEHM CRLDIDSPPI TARNTGIICT IGPASRSVET LKEMIKSGMN VARLNFSHGT HEYHAETIKN VRTATESFAS 101 DPILYRPVAV ALDTKGPEIR TGLIKGSGTA EVELKKGATL KITLDNAYME KCDENILWLD YKNICKVVEV GSKIYVDDGL ISLQVKQKGA DFLVTEVENG 201 GSLGSKKGVN LPGAAVDLPA VSEKDIQDLK FGVEQDVDMV FASFIRKASD VHEVRKVLGE KGKNIKIISK IENHEGVRRF DEILEASDGI MVARGDLGIE 301 IPAEKVFLAQ KMMIGRCNRA GKPVICATQM LESMIKKPRP TRAEGSDVAN AVLDGADCIM LSGETAKGDY PLEAVRMQHL IAREAEAAIY HLQLFEELRR 401 LAPITSDPTE ATAVGAVEAS FKCCSGAIIV LTKSGRSAHQ VARYRPRAPI IAVTRNPQTA RQAHLYRGIF PVLCKDPVQE AWAEDVDLRV NFAMNVGKAR 501 GFFKKGDVVI VLTGWRPGSG FTNTMRVVPV P ``` | | |
| Evidence | Conclusive | | |
| Peptide list | 136\*\*, 311\*\*, 504\*\*, 725\*\*, 907, 922\*\*, 1012\*\* | | |
| Peptides | 136\*\* | Confidence | Green |
| Runs | 1, 2, 3, 4 |
| Relation | NonDiscriminating |
| Proteins | KPYM\_HUMAN, KPYM\_HUMAN |
| Sequence | ``` GSGTAEVELK ``` |
| Position | 126 |
| PTMs | none |
| 311\*\* | Confidence | Green |
| Runs | 1, 2, 3, 4 |
| Relation | NonDiscriminating |
| Proteins | KPYM\_HUMAN, KPYM\_HUMAN |
| Sequence | ``` APIIAVTR ``` |
| Position | 448 |
| PTMs | none |
| 504\*\* | Confidence | Green |
| Runs | 1, 3, 4 |
| Relation | NonDiscriminating |
| Proteins | KPYM\_HUMAN, KPYM\_HUMAN |
| Sequence | ``` LDIDSPPITAR ``` |
| Position | 33 |
| PTMs | none |
| 725\*\* | Confidence | Green |
| Runs | 1, 2, 3, 4 |
| Relation | NonDiscriminating |
| Proteins | KPYM\_HUMAN, KPYM\_HUMAN |
| Sequence | ``` GVNLPGAAVDLPAVSEK ``` |
| Position | 208 |
| PTMs | none |
| 907 | Confidence | Green |
| Runs | 1, 2, 3, 4 |
| Relation | Unique |
| Proteins | KPYM\_HUMAN |
| Sequence | ``` LAPITSDPTEATAVGAVEASFK ``` |
| Position | 401 |
| PTMs | none |
| 922\*\* | Confidence | Green |
| Runs | 1, 2, 3, 4 |
| Relation | NonDiscriminating |
| Proteins | KPYM\_HUMAN, KPYM\_HUMAN |
| Sequence | ``` TATESFASDPILYRPVAVALDTK ``` |
| Position | 93 |
| PTMs | none |
| 1012\*\* | Confidence | Green |
| Runs | 1, 2, 3, 4 |
| Relation | NonDiscriminating |
| Proteins | KPYM\_HUMAN, KPYM\_HUMAN |
| Sequence | ``` AEGSDVANAVLDGADCIMLSGETAK ``` |
| Position | 343 |
| PTMs | Variant #1: Carbamidomethyl+C(16)  Variant #2: Carbamidomethyl+C(16) Oxidation+M(18)  Variant #3: Carbamidomethyl+C(16) Deamidation+N(8) |

  

Protein P60842

| Name | IF4A1\_HUMAN | | |
| Description | Eukaryotic initiation factor 4A I OS Homo sapiens GN EIF4A1 PE 1 SV 1 | | |
| Sequence | ```   1 MSASQDSRSR DNGPDGMEPE GVIESNWNEI VDSFDDMNLS ESLLRGIYAY GFEKPSAIQQ RAILPCIKGY DVIAQAQSGT GKTATFAISI LQQIELDLKA 101 TQALVLAPTR ELAQQIQKVV MALGDYMGAS CHACIGGTNV RAEVQKLQME APHIIVGTPG RVFDMLNRRY LSPKYIKMFV LDEADEMLSR GFKDQIYDIF 201 QKLNSNTQVV LLSATMPSDV LEVTKKFMRD PIRILVKKEE LTLEGIRQFY INVEREEWKL DTLCDLYETL TITQAVIFIN TRRKVDWLTE KMHARDFTVS 301 AMHGDMDQKE RDVIMREFRS GSSRVLITTD LLARGIDVQQ VSLVINYDLP TNRENYIHRI GRGGRFGRKG VAINMVTEED KRTLRDIETF YNTSIEEMPL 401 NVADLI ``` | | |
| Evidence | Conclusive | | |
| Peptide list | 93\*\*, 349\*\*, 359\*\*, 402, 753\*\*, 1643 | | |
| Peptides | 93\*\* | Confidence | Green |
| Runs | 1, 2, 3, 4 |
| Relation | NonDiscriminating |
| Proteins | IF4A1\_HUMAN, IF4A2\_HUMAN, IF4A2\_HUMAN |
| Sequence | ``` ELAQQIQK ``` |
| Position | 111 |
| PTMs | none |
| 349\*\* | Confidence | Green |
| Runs | 1, 3, 4 |
| Relation | NonDiscriminating |
| Proteins | IF4A1\_HUMAN, IF4A2\_HUMAN, IF4A2\_HUMAN |
| Sequence | ``` GYDVIAQAQSGTGK ``` |
| Position | 69 |
| PTMs | none |
| 359\*\* | Confidence | Green |
| Runs | 1, 2, 3 |
| Relation | NonDiscriminating |
| Proteins | IF4A1\_HUMAN, IF4A2\_HUMAN, IF4A2\_HUMAN |
| Sequence | ``` KVDWLTEK ``` |
| Position | 284 |
| PTMs | none |
| 402 | Confidence | Green |
| Runs | 1, 2, 3, 4 |
| Relation | Unique |
| Proteins | IF4A1\_HUMAN |
| Sequence | ``` ATQALVLAPTR ``` |
| Position | 100 |
| PTMs | none |
| 753\*\* | Confidence | Green |
| Runs | 1, 2, 3, 4 |
| Relation | NonDiscriminating |
| Proteins | IF4A1\_HUMAN, IF4A2\_HUMAN, IF4A2\_HUMAN |
| Sequence | ``` VLITTDLLAR ``` |
| Position | 325 |
| PTMs | none |
| 1643 | Confidence | Green |
| Runs | 2, 3, 4 |
| Relation | Unique |
| Proteins | IF4A1\_HUMAN |
| Sequence | ``` LNSNTQVVLLSATMPSDVLEVTK ``` |
| Position | 203 |
| PTMs | none |

  

Protein P31943

| Name | HNRH1\_HUMAN | | |
| Description | Heterogeneous nuclear ribonucleoprotein H OS Homo sapiens GN HNRNPH1 PE 1 SV 4 | | |
| Sequence | ```   1 MMLGTEGGEG FVVKVRGLPW SCSADEVQRF FSDCKIQNGA QGIRFIYTRE GRPSGEAFVE LESEDEVKLA LKKDRETMGH RYVEVFKSNN VEMDWVLKHT 101 GPNSPDTAND GFVRLRGLPF GCSKEEIVQF FSGLEIVPNG ITLPVDFQGR STGEAFVQFA SQEIAEKALK KHKERIGHRY IEIFKSSRAE VRTHYDPPRK 201 LMAMQRPGPY DRPGAGRGYN SIGRGAGFER MRRGAYGGGY GGYDDYNGYN DGYGFGSDRF GRDLNYCFSG MSDHRYGDGG STFQSTTGHC VHMRGLPYRA 301 TENDIYNFFS PLNPVRVHIE IGPDGRVTGE ADVEFATHED AVAAMSKDKA NMQHRYVELF LNSTAGASGG AYEHRYVELF LNSTAGASGG AYGSQMMGGM 401 GLSNQSSYGG PASQQLSGGY GGGYGGQSSM SGYDQVLQEN SSDFQSNIA ``` | | |
| Evidence | Conclusive | | |
| Peptide list | 185\*\*, 272\*\*, 550, 724, 880\*\*, 1057\*\* | | |
| Peptides | 185\*\* | Confidence | Green |
| Runs | 1, 2, 3, 4 |
| Relation | NonDiscriminating |
| Proteins | HNRH1\_HUMAN, HNRH2\_HUMAN |
| Sequence | ``` HTGPNSPDTANDGFVR ``` |
| Position | 99 |
| PTMs | none |
| 272\*\* | Confidence | Green |
| Runs | 1, 2, 3, 4 |
| Relation | NonDiscriminating |
| Proteins | HNRH1\_HUMAN, HNRPF\_HUMAN, HNRH2\_HUMAN |
| Sequence | ``` VHIEIGPDGR ``` |
| Position | 317 |
| PTMs | none |
| 550 | Confidence | Green |
| Runs | 1, 2, 3, 4 |
| Relation | Unique |
| Proteins | HNRH1\_HUMAN |
| Sequence | ``` GLPWSCSADEVQR ``` |
| Position | 17 |
| PTMs | Carbamidomethyl+C(6) |
| 724 | Confidence | Yellow |
| Runs | 1, 2, 4 |
| Relation | Unique |
| Proteins | HNRH1\_HUMAN |
| Sequence | ``` YVELFLNSTAGASGGAYEHR ``` |
| Position | 356 |
| PTMs | none |
| 880\*\* | Confidence | Green |
| Runs | 1, 2, 3, 4 |
| Relation | NonDiscriminating |
| Proteins | HNRH1\_HUMAN, HNRH2\_HUMAN |
| Sequence | ``` STGEAFVQFASQEIAEK ``` |
| Position | 151 |
| PTMs | none |
| 1057\*\* | Confidence | Green |
| Runs | 1, 3, 4 |
| Relation | NonDiscriminating |
| Proteins | HNRH1\_HUMAN, HNRPF\_HUMAN |
| Sequence | ``` ATENDIYNFFSPLNPVR ``` |
| Position | 300 |
| PTMs | none |

  

Protein P08758

| Name | ANXA5\_HUMAN | | |
| Description | Annexin A5 OS Homo sapiens GN ANXA5 PE 1 SV 2 | | |
| Sequence | ```   1 MAQVLRGTVT DFPGFDERAD AETLRKAMKG LGTDEESILT LLTSRSNAQR QEISAAFKTL FGRDLLDDLK SELTGKFEKL IVALMKPSRL YDAYELKHAL 101 KGAGTNEKVL TEIIASRTPE ELRAIKQVYE EEYGSSLEDD VVGDTSGYYQ RMLVVLLQAN RDPDAGIDEA QVEQDAQALF QAGELKWGTD EEKFITIFGT 201 RSVSHLRKVF DKYMTISGFQ IEETIDRETS GNLEQLLLAV VKSIRSIPAY LAETLYYAMK GAGTDDHTLI RVMVSRSEID LFNIRKEFRK NFATSLYSMI 301 KGDTSGDYKK ALLLLCGEDD ``` | | |
| Evidence | Conclusive | | |
| Peptide list | 479, 855, 1109 | | |
| Peptides | 479 | Confidence | Green |
| Runs | 1, 2, 3 |
| Relation | Unique |
| Proteins | ANXA5\_HUMAN |
| Sequence | ``` VLTEIIASR ``` |
| Position | 109 |
| PTMs | none |
| 855 | Confidence | Green |
| Runs | 1, 2, 3, 4 |
| Relation | Unique |
| Proteins | ANXA5\_HUMAN |
| Sequence | ``` SEIDLFNIR ``` |
| Position | 277 |
| PTMs | none |
| 1109 | Confidence | Green |
| Runs | 1, 2, 3, 4 |
| Relation | Unique |
| Proteins | ANXA5\_HUMAN |
| Sequence | ``` GLGTDEESILTLLTSR ``` |
| Position | 30 |
| PTMs | none |

  

Protein Q01105-2

| Name | SET\_HUMAN | | |
| Description | Isoform 2 of Protein SET OS Homo sapiens GN SET | | |
| Sequence | ```   1 MSAPAAKVSK KELNSNHDGA DETSEKEQQE AIEHIDEVQN EIDRLNEQAS EEILKVEQKY NKLRQPFFQK RSELIAKIPN FWVTTFVNHP QVSALLGEED 101 EEALHYLTRV EVTEFEDIKS GYRIDFYFDE NPYFENKVLS KEFHLNESGD PSSKSTEIKW KSGKDLTKRS SQTQNKASRK RQHEEPESFF TWFTDHSDAG 201 ADELGEVIKD DIWPNPLQYY LVPDMDDEEG EGEEDDDDDE EEEGLEDIDE EGDEDEGEED EDDDEGEEGE EDEGEDD ``` | | |
| Evidence | Conclusive | | |
| Peptide list | 211\*\*, 378\*\*, 1169 | | |
| Peptides | 211\*\* | Confidence | Green |
| Runs | 1, 2, 4 |
| Relation | NonDiscriminating |
| Proteins | SET\_HUMAN, SET\_HUMAN |
| Sequence | ``` EFHLNESGDPSSK ``` |
| Position | 142 |
| PTMs | none |
| 378\*\* | Confidence | Green |
| Runs | 1, 2, 3, 4 |
| Relation | NonDiscriminating |
| Proteins | SET\_HUMAN, SET\_HUMAN |
| Sequence | ``` LNEQASEEILK ``` |
| Position | 45 |
| PTMs | none |
| 1169 | Confidence | Yellow |
| Runs | 2, 3, 4 |
| Relation | Unique |
| Proteins | SET\_HUMAN |
| Sequence | ``` ELNSNHDGADETSEK ``` |
| Position | 12 |
| PTMs | none |

  

Protein P00924

| Name | ENO1\_YEAST | | |
| Description | Enolase 1 OS Saccharomyces cerevisiae GN ENO1 PE 1 SV 2 | | |
| Sequence | ```   1 MAVSKVYARS VYDSRGNPTV EVELTTEKGV FRSIVPSGAS TGVHEALEMR DGDKSKWMGK GVLHAVKNVN DVIAPAFVKA NIDVKDQKAV DDFLISLDGT 101 ANKSKLGANA ILGVSLAASR AAAAEKNVPL YKHLADLSKS KTSPYVLPVP FLNVLNGGSH AGGALALQEF MIAPTGAKTF AEALRIGSEV YHNLKSLTKK 201 RYGASAGNVG DEGGVAPNIQ TAEEALDLIV DAIKAAGHDG KVKIGLDCAS SEFFKDGKYD LDFKNPNSDK SKWLTGPQLA DLYHSLMKRY PIVSIEDPFA 301 EDDWEAWSHF FKTAGIQIVA DDLTVTNPKR IATAIEKKAA DALLLKVNQI GTLSESIKAA QDSFAAGWGV MVSHRSGETE DTFIADLVVG LRTGQIKTGA 401 PARSERLAKL NQLLRIEEEL GDNAVFAGEN FHHGDKL ``` | | |
| Evidence | Conclusive | | |
| Peptide list | 438, 486, 659, 668 | | |
| Peptides | 438 | Confidence | Green |
| Runs | 1, 2, 4 |
| Relation | Unique |
| Proteins | ENO1\_YEAST |
| Sequence | ``` AADALLLK ``` |
| Position | 339 |
| PTMs | none |
| 486 | Confidence | Green |
| Runs | 1, 2, 3, 4 |
| Relation | Unique |
| Proteins | ENO1\_YEAST |
| Sequence | ``` VNQIGTLSESIK ``` |
| Position | 347 |
| PTMs | none |
| 659 | Confidence | Green |
| Runs | 1, 3, 4 |
| Relation | Unique |
| Proteins | ENO1\_YEAST |
| Sequence | ``` IEEELGDNAVFAGENFHHGDKL ``` |
| Position | 416 |
| PTMs | none |
| 668 | Confidence | Green |
| Runs | 1, 2, 3, 4 |
| Relation | Unique |
| Proteins | ENO1\_YEAST |
| Sequence | ``` NVNDVIAPAFVK ``` |
| Position | 68 |
| PTMs | none |

  

Protein P30101

| Name | PDIA3\_HUMAN | | |
| Description | Protein disulfide isomerase A3 OS Homo sapiens GN PDIA3 PE 1 SV 4 | | |
| Sequence | ```   1 MRLRRLALFP GVALLLAAAR LAAASDVLEL TDDNFESRIS DTGSAGLMLV EFFAPWCGHC KRLAPEYEAA ATRLKGIVPL AKVDCTANTN TCNKYGVSGY 101 PTLKIFRDGE EAGAYDGPRT ADGIVSHLKK QAGPASVPLR TEEEFKKFIS DKDASIVGFF DDSFSEAHSE FLKAASNLRD NYRFAHTNVE SLVNEYDDNG 201 EGIILFRPSH LTNKFEDKTV AYTEQKMTSG KIKKFIQENI FGICPHMTED NKDLIQGKDL LIAYYDVDYE KNAKGSNYWR NRVMMVAKKF LDAGHKLNFA 301 VASRKTFSHE LSDFGLESTA GEIPVVAIRT AKGEKFVMQE EFSRDGKALE RFLQDYFDGN LKRYLKSEPI PESNDGPVKV VVAENFDEIV NNENKDVLIE 401 FYAPWCGHCK NLEPKYKELG EKLSKDPNIV IAKMDATAND VPSPYEVRGF PTIYFSPANK KLNPKKYEGG RELSDFISYL QREATNPPVI QEEKPKKKKK 501 AQEDL ``` | | |
| Evidence | Conclusive | | |
| Peptide list | 158, 212, 221, 389, 984, 1267 | | |
| Peptides | 158 | Confidence | Green |
| Runs | 1, 3, 4 |
| Relation | Unique |
| Proteins | PDIA3\_HUMAN |
| Sequence | ``` EATNPPVIQEEKPK ``` |
| Position | 483 |
| PTMs | none |
| 212 | Confidence | Green |
| Runs | 1, 2, 4 |
| Relation | Unique |
| Proteins | PDIA3\_HUMAN |
| Sequence | ``` LAPEYEAAATR ``` |
| Position | 63 |
| PTMs | none |
| 221 | Confidence | Green |
| Runs | 1, 2, 4 |
| Relation | Unique |
| Proteins | PDIA3\_HUMAN |
| Sequence | ``` QAGPASVPLR ``` |
| Position | 131 |
| PTMs | none |
| 389 | Confidence | Green |
| Runs | 1, 2, 3 |
| Relation | Unique |
| Proteins | PDIA3\_HUMAN |
| Sequence | ``` LNFAVASR ``` |
| Position | 297 |
| PTMs | none |
| 984 | Confidence | Green |
| Runs | 1, 2, 3, 4 |
| Relation | Unique |
| Proteins | PDIA3\_HUMAN |
| Sequence | ``` TFSHELSDFGLESTAGEIPVVAIR ``` |
| Position | 306 |
| PTMs | none |
| 1267 | Confidence | Green |
| Runs | 2, 3, 4 |
| Relation | Unique |
| Proteins | PDIA3\_HUMAN |
| Sequence | ``` SEPIPESNDGPVK ``` |
| Position | 367 |
| PTMs | none |

  

Protein P25705

| Name | ATPA\_HUMAN | | |
| Description | ATP synthase subunit alpha mitochondrial OS Homo sapiens GN ATP5A1 PE 1 SV 1 | | |
| Sequence | ```   1 MLSVRVAAAV VRALPRRAGL VSRNALGSSF IAARNFHASN THLQKTGTAE MSSILEERIL GADTSVDLEE TGRVLSIGDG IARVHGLRNV QAEEMVEFSS 101 GLKGMSLNLE PDNVGVVVFG NDKLIKEGDI VKRTGAIVDV PVGEELLGRV VDALGNAIDG KGPIGSKTRR RVGLKAPGII PRISVREPMQ TGIKAVDSLV 201 PIGRGQRELI IGDRQTGKTS IAIDTIINQK RFNDGSDEKK KLYCIYVAIG QKRSTVAQLV KRLTDADAMK YTIVVSATAS DAAPLQYLAP YSGCSMGEYF 301 RDNGKHALII YDDLSKQAVA YRQMSLLLRR PPGREAYPGD VFYLHSRLLE RAAKMNDAFG GGSLTALPVI ETQAGDVSAY IPTNVISITD GQIFLETELF 401 YKGIRPAINV GLSVSRVGSA AQTRAMKQVA GTMKLELAQY REVAAFAQFG SDLDAATQQL LSRGVRLTEL LKQGQYSPMA IEEQVAVIYA GVRGYLDKLE 501 PSKITKFENA FLSHVVSQHQ ALLGTIRADG KISEQSDAKL KEIVTNFLAG FEA ``` | | |
| Evidence | Conclusive | | |
| Peptide list | 253, 452, 546, 635, 1146 | | |
| Peptides | 253 | Confidence | Green |
| Runs | 1, 2, 3, 4 |
| Relation | Unique |
| Proteins | ATPA\_HUMAN |
| Sequence | ``` STVAQLVK ``` |
| Position | 254 |
| PTMs | none |
| 452 | Confidence | Green |
| Runs | 1, 2, 3, 4 |
| Relation | Unique |
| Proteins | ATPA\_HUMAN |
| Sequence | ``` VVDALGNAIDGK ``` |
| Position | 150 |
| PTMs | none |
| 546 | Confidence | Green |
| Runs | 1, 2, 3, 4 |
| Relation | Unique |
| Proteins | ATPA\_HUMAN |
| Sequence | ``` AVDSLVPIGR ``` |
| Position | 195 |
| PTMs | none |
| 635 | Confidence | Green |
| Runs | 1, 2, 4 |
| Relation | Unique |
| Proteins | ATPA\_HUMAN |
| Sequence | ``` EAYPGDVFYLHSR ``` |
| Position | 335 |
| PTMs | none |
| 1146 | Confidence | Green |
| Runs | 1, 2, 3, 4 |
| Relation | Unique |
| Proteins | ATPA\_HUMAN |
| Sequence | ``` EVAAFAQFGSDLDAATQQLLSR ``` |
| Position | 442 |
| PTMs | none |

  

Protein P52597

| Name | HNRPF\_HUMAN | | |
| Description | Heterogeneous nuclear ribonucleoprotein F OS Homo sapiens GN HNRNPF PE 1 SV 3 | | |
| Sequence | ```   1 MMLGPEGGEG FVVKLRGLPW SCSVEDVQNF LSDCTIHDGA AGVHFIYTRE GRQSGEAFVE LGSEDDVKMA LKKDRESMGH RYIEVFKSHR TEMDWVLKHS 101 GPNSADSAND GFVRLRGLPF GCTKEEIVQF FSGLEIVPNG ITLPVDPEGK ITGEAFVQFA SQELAEKALG KHKERIGHRY IEVFKSSQEE VRSYSDPPLK 201 FMSVQRPGPY DRPGTARRYI GIVKQAGLER MRPGAYSTGY GGYEEYSGLS DGYGFTTDLF GRDLSYCLSG MYDHRYGDSE FTVQSTTGHC VHMRGLPYKA 301 TENDIYNFFS PLNPVRVHIE IGPDGRVTGE ADVEFATHEE AVAAMSKDRA NMQHRYIELF LNSTTGASNG AYSSQVMQGM GVSAAQATYS GLESQSVSGC 401 YGAGYSGQNS MGGYD ``` | | |
| Evidence | Conclusive | | |
| Peptide list | 272\*\*, 974, 1057\*\* | | |
| Peptides | 272\*\* | Confidence | Green |
| Runs | 1, 2, 3, 4 |
| Relation | NonDiscriminating |
| Proteins | HNRH1\_HUMAN, HNRPF\_HUMAN, HNRH2\_HUMAN |
| Sequence | ``` VHIEIGPDGR ``` |
| Position | 317 |
| PTMs | none |
| 974 | Confidence | Green |
| Runs | 1, 2, 3 |
| Relation | Unique |
| Proteins | HNRPF\_HUMAN |
| Sequence | ``` ITGEAFVQFASQELAEK ``` |
| Position | 151 |
| PTMs | Variant #1: Deamidation+Q(12)  Variant #2: none |
| 1057\*\* | Confidence | Green |
| Runs | 1, 3, 4 |
| Relation | NonDiscriminating |
| Proteins | HNRH1\_HUMAN, HNRPF\_HUMAN |
| Sequence | ``` ATENDIYNFFSPLNPVR ``` |
| Position | 300 |
| PTMs | none |

  

Protein P19338

| Name | NUCL\_HUMAN | | |
| Description | Nucleolin OS Homo sapiens GN NCL PE 1 SV 3 | | |
| Sequence | ```   1 MVKLAKAGKN QGDPKKMAPP PKEVEEDSED EEMSEDEEDD SSGEEVVIPQ KKGKKAAATS AKKVVVSPTK KVAVATPAKK AAVTPGKKAA ATPAKKTVTP 101 AKAVTTPGKK GATPGKALVA TPGKKGAAIP AKGAKNGKNA KKEDSDEEED DDSEEDEEDD EDEDEDEDEI EPAAMKAAAA APASEDEDDE DDEDDEDDDD 201 DEEDDSEEEA METTPAKGKK AAKVVPVKAK NVAEDEDEEE DDEDEDDDDD EDDEDDDDED DEEEEEEEEE EPVKEAPGKR KKEMAKQKAA PEAKKQKVEG 301 TEPTTAFNLF VGNLNFNKSA PELKTGISDV FAKNDLAVVD VRIGMTRKFG YVDFESAEDL EKALELTGLK VFGNEIKLEK PKGKDSKKER DARTLLAKNL 401 PYKVTQDELK EVFEDAAEIR LVSKDGKSKG IAYIEFKTEA DAEKTFEEKQ GTEIDGRSIS LYYTGEKGQN QDYRGGKNST WSGESKTLVL SNLSYSATEE 501 TLQEVFEKAT FIKVPQNQNG KSKGYAFIEF ASFEDAKEAL NSCNKREIEG RAIRLELQGP RGSPNARSQP SKTLFVKGLS EDTTEETLKE SFDGSVRARI 601 VTDRETGSSK GFGFVDFNSE EDAKAAKEAM EDGEIDGNKV TLDWAKPKGE GGFGGRGGGR GGFGGRGGGR GGRGGFGGRG RGGFGGRGGF RGGRGGGGDH 701 KPQGKKTKFE ``` | | |
| Evidence | Conclusive | | |
| Peptide list | 39, 113, 217, 423, 474, 509 | | |
| Peptides | 39 | Confidence | Green |
| Runs | 1, 2, 3, 4 |
| Relation | Unique |
| Proteins | NUCL\_HUMAN |
| Sequence | ``` VAVATPAK ``` |
| Position | 72 |
| PTMs | none |
| 113 | Confidence | Green |
| Runs | 1, 2, 3, 4 |
| Relation | Unique |
| Proteins | NUCL\_HUMAN |
| Sequence | ``` ALVATPGK ``` |
| Position | 117 |
| PTMs | none |
| 217 | Confidence | Green |
| Runs | 1, 2, 3, 4 |
| Relation | Unique |
| Proteins | NUCL\_HUMAN |
| Sequence | ``` LELQGPR ``` |
| Position | 555 |
| PTMs | none |
| 423 | Confidence | Green |
| Runs | 1, 2, 3, 4 |
| Relation | Unique |
| Proteins | NUCL\_HUMAN |
| Sequence | ``` NDLAVVDVR ``` |
| Position | 334 |
| PTMs | none |
| 474 | Confidence | Green |
| Runs | 1, 2, 4 |
| Relation | Unique |
| Proteins | NUCL\_HUMAN |
| Sequence | ``` EVFEDAAEIR ``` |
| Position | 411 |
| PTMs | none |
| 509 | Confidence | Green |
| Runs | 1, 2, 3, 4 |
| Relation | Unique |
| Proteins | NUCL\_HUMAN |
| Sequence | ``` ALELTGLK ``` |
| Position | 363 |
| PTMs | none |

  

Protein P02768

| Name | ALBU\_HUMAN | | |
| Description | Serum albumin OS Homo sapiens GN ALB PE 1 SV 2 | | |
| Sequence | ```   1 MKWVTFISLL FLFSSAYSRG VFRRDAHKSE VAHRFKDLGE ENFKALVLIA FAQYLQQCPF EDHVKLVNEV TEFAKTCVAD ESAENCDKSL HTLFGDKLCT 101 VATLRETYGE MADCCAKQEP ERNECFLQHK DDNPNLPRLV RPEVDVMCTA FHDNEETFLK KYLYEIARRH PYFYAPELLF FAKRYKAAFT ECCQAADKAA 201 CLLPKLDELR DEGKASSAKQ RLKCASLQKF GERAFKAWAV ARLSQRFPKA EFAEVSKLVT DLTKVHTECC HGDLLECADD RADLAKYICE NQDSISSKLK 301 ECCEKPLLEK SHCIAEVEND EMPADLPSLA ADFVESKDVC KNYAEAKDVF LGMFLYEYAR RHPDYSVVLL LRLAKTYETT LEKCCAAADP HECYAKVFDE 401 FKPLVEEPQN LIKQNCELFE QLGEYKFQNA LLVRYTKKVP QVSTPTLVEV SRNLGKVGSK CCKHPEAKRM PCAEDYLSVV LNQLCVLHEK TPVSDRVTKC 501 CTESLVNRRP CFSALEVDET YVPKEFNAET FTFHADICTL SEKERQIKKQ TALVELVKHK PKATKEQLKA VMDDFAAFVE KCCKADDKET CFAEEGKKLV 601 AASQAALGL ``` | | |
| Evidence | Conclusive | | |
| Peptide list | 133\*\*, 153\*\*, 205\*\*, 446, 511\*\* | | |
| Peptides | 133\*\* | Confidence | Green |
| Runs | 1, 2, 3, 4 |
| Relation | NonDiscriminating |
| Proteins | ALBU\_HUMAN, ALBU\_HUMAN |
| Sequence | ``` CCTESLVNR ``` |
| Position | 500 |
| PTMs | Carbamidomethyl+C(1) Carbamidomethyl+C(2) |
| 153\*\* | Confidence | Green |
| Runs | 1, 2, 3, 4 |
| Relation | NonDiscriminating |
| Proteins | ALBU\_HUMAN, ALBU\_HUMAN |
| Sequence | ``` YICENQDSISSK ``` |
| Position | 287 |
| PTMs | Carbamidomethyl+C(3) |
| 205\*\* | Confidence | Green |
| Runs | 1, 2, 3, 4 |
| Relation | NonDiscriminating |
| Proteins | ALBU\_HUMAN, ALBU\_HUMAN |
| Sequence | ``` LVTDLTK ``` |
| Position | 258 |
| PTMs | none |
| 446 | Confidence | Green |
| Runs | 1, 2, 3, 4 |
| Relation | Unique |
| Proteins | ALBU\_HUMAN |
| Sequence | ``` YLYEIAR ``` |
| Position | 162 |
| PTMs | none |
| 511\*\* | Confidence | Green |
| Runs | 1, 2, 3, 4 |
| Relation | NonDiscriminating |
| Proteins | ALBU\_HUMAN, ALBU\_HUMAN |
| Sequence | ``` KVPQVSTPTLVEVSR ``` |
| Position | 438 |
| PTMs | none |

  

Protein P17987

| Name | TCPA\_HUMAN | | |
| Description | T complex protein 1 subunit alpha OS Homo sapiens GN TCP1 PE 1 SV 1 | | |
| Sequence | ```   1 MEGPLSVFGD RSTGETIRSQ NVMAAASIAN IVKSSLGPVG LDKMLVDDIG DVTITNDGAT ILKLLEVEHP AAKVLCELAD LQDKEVGDGT TSVVIIAAEL 101 LKNADELVKQ KIHPTSVISG YRLACKEAVR YINENLIVNT DELGRDCLIN AAKTSMSSKI IGINGDFFAN MVVDAVLAIK YTDIRGQPRY PVNSVNILKA 201 HGRSQMESML ISGYALNCVV GSQGMPKRIV NAKIACLDFS LQKTKMKLGV QVVITDPEKL DQIRQRESDI TKERIQKILA TGANVILTTG GIDDMCLKYF 301 VEAGAMAVRR VLKRDLKRIA KASGATILST LANLEGEETF EAAMLGQAEE VVQERICDDE LILIKNTKAR TSASIILRGA NDFMCDEMER SLHDALCVVK 401 RVLESKSVVP GGGAVEAALS IYLENYATSM GSREQLAIAE FARSLLVIPN TLAVNAAQDS TDLVAKLRAF HNEAQVNPER KNLKWIGLDL SNGKPRDNKQ 501 AGVFEPTIVK VKSLKFATEA AITILRIDDL IKLHPESKDD KHGSYEDAVH SGALND ``` | | |
| Evidence | Conclusive | | |
| Peptide list | 95, 323, 682 | | |
| Peptides | 95 | Confidence | Green |
| Runs | 1, 2, 3, 4 |
| Relation | Unique |
| Proteins | TCPA\_HUMAN |
| Sequence | ``` AFHNEAQVNPER ``` |
| Position | 469 |
| PTMs | none |
| 323 | Confidence | Green |
| Runs | 1, 3, 4 |
| Relation | Unique |
| Proteins | TCPA\_HUMAN |
| Sequence | ``` IHPTSVISGYR ``` |
| Position | 112 |
| PTMs | none |
| 682 | Confidence | Green |
| Runs | 1, 2, 3, 4 |
| Relation | Unique |
| Proteins | TCPA\_HUMAN |
| Sequence | ``` EQLAIAEFAR ``` |
| Position | 434 |
| PTMs | Variant #1: none Variant #2: Deamidation+Q(2) |

  

Protein P22314

| Name | UBA1\_HUMAN | | |
| Description | Ubiquitin like modifier activating enzyme 1 OS Homo sapiens GN UBA1 PE 1 SV 3 | | |
| Sequence | ```    1 MSSSPLSKKR RVSGPDPKPG SNCSPAQSVL SEVPSVPTNG MAKNGSEADI DEGLYSRQLY VLGHEAMKRL QTSSVLVSGL RGLGVEIAKN IILGGVKAVT  101 LHDQGTAQWA DLSSQFYLRE EDIGKNRAEV SQPRLAELNS YVPVTAYTGP LVEDFLSGFQ VVVLTNTPLE DQLRVGEFCH NRGIKLVVAD TRGLFGQLFC  201 DFGEEMILTD SNGEQPLSAM VSMVTKDNPG VVTCLDEARH GFESGDFVSF SEVQGMVELN GNQPMEIKVL GPYTFSICDT SNFSDYIRGG IVSQVKVPKK  301 ISFKSLVASL AEPDFVVTDF AKFSRPAQLH IGFQALHQFC AQHGRPPRPR NEEDAAELVA LAQAVNARAL PAVQQNNLDE DLIRKLAYVA AGDLAPINAF  401 IGGLAAQEVM KACSGKFMPI MQWLYFDALE CLPEDKEVLT EDKCLQRQNR YDGQVAVFGS DLQEKLGKQK YFLVGAGAIG CELLKNFAMI GLGCGEGGEI  501 IVTDMDTIEK SNLNRQFLFR PWDVTKLKSD TAAAAVRQMN PHIRVTSHQN RVGPDTERIY DDDFFQNLDG VANALDNVDA RMYMDRRCVY YRKPLLESGT  601 LGTKGNVQVV IPFLTESYSS SQDPPEKSIP ICTLKNFPNA IEHTLQWARD EFEGLFKQPA ENVNQYLTDP KFVERTLRLA GTQPLEVLEA VQRSLVLQRP  701 QTWADCVTWA CHHWHTQYSN NIRQLLHNFP PDQLTSSGAP FWSGPKRCPH PLTFDVNNPL HLDYVMAAAN LFAQTYGLTG SQDRAAVATF LQSVQVPEFT  801 PKSGVKIHVS DQELQSANAS VDDSRLEELK ATLPSPDKLP GFKMYPIDFE KDDDSNFHMD FIVAASNLRA ENYDIPSADR HKSKLIAGKI IPAIATTTAA  901 VVGLVCLELY KVVQGHRQLD SYKNGFLNLA LPFFGFSEPL AAPRHQYYNQ EWTLWDRFEV QGLQPNGEEM TLKQFLDYFK TEHKLEITML SQGVSMLYSF 1001 FMPAAKLKER LDQPMTEIVS RVSKRKLGRH VRALVLELCC NDESGEDVEV PYVRYTIR ``` | | |
| Evidence | Conclusive | | |
| Peptide list | 305, 428, 589, 832, 881, 924, 1009, 1032 | | |
| Peptides | 305 | Confidence | Green |
| Runs | 1, 2, 3 |
| Relation | Unique |
| Proteins | UBA1\_HUMAN |
| Sequence | ``` KPLLESGTLGTK ``` |
| Position | 593 |
| PTMs | none |
| 428 | Confidence | Green |
| Runs | 1, 3, 4 |
| Relation | Unique |
| Proteins | UBA1\_HUMAN |
| Sequence | ``` NIILGGVK ``` |
| Position | 90 |
| PTMs | none |
| 589 | Confidence | Green |
| Runs | 1, 2, 3, 4 |
| Relation | Unique |
| Proteins | UBA1\_HUMAN |
| Sequence | ``` LQTSSVLVSGLR ``` |
| Position | 70 |
| PTMs | none |
| 832 | Confidence | Green |
| Runs | 1, 3, 4 |
| Relation | Unique |
| Proteins | UBA1\_HUMAN |
| Sequence | ``` NFPNAIEHTLQWAR ``` |
| Position | 636 |
| PTMs | none |
| 881 | Confidence | Green |
| Runs | 1, 2, 3, 4 |
| Relation | Unique |
| Proteins | UBA1\_HUMAN |
| Sequence | ``` LAGTQPLEVLEAVQR ``` |
| Position | 679 |
| PTMs | none |
| 924 | Confidence | Green |
| Runs | 1, 3, 4 |
| Relation | Unique |
| Proteins | UBA1\_HUMAN |
| Sequence | ``` NEEDAAELVALAQAVNAR ``` |
| Position | 351 |
| PTMs | none |
| 1009 | Confidence | Green |
| Runs | 1, 2, 3, 4 |
| Relation | Unique |
| Proteins | UBA1\_HUMAN |
| Sequence | ``` AAVATFLQSVQVPEFTPK ``` |
| Position | 785 |
| PTMs | none |
| 1032 | Confidence | Green |
| Runs | 1, 2, 3, 4 |
| Relation | Unique |
| Proteins | UBA1\_HUMAN |
| Sequence | ``` SLVASLAEPDFVVTDFAK ``` |
| Position | 305 |
| PTMs | none |

  

Protein P06744

| Name | G6PI\_HUMAN | | |
| Description | Glucose 6 phosphate isomerase OS Homo sapiens GN GPI PE 1 SV 4 | | |
| Sequence | ```   1 MAALTRDPQF QKLQQWYREH RSELNLRRLF DANKDRFNHF SLTLNTNHGH ILVDYSKNLV TEDVMRMLVD LAKSRGVEAA RERMFNGEKI NYTEGRAVLH 101 VALRNRSNTP ILVDGKDVMP EVNKVLDKMK SFCQRVRSGD WKGYTGKTIT DVINIGIGGS DLGPLMVTEA LKPYSSGGPR VWYVSNIDGT HIAKTLAQLN 201 PESSLFIIAS KTFTTQETIT NAETAKEWFL QAAKDPSAVA KHFVALSTNT TKVKEFGIDP QNMFEFWDWV GGRYSLWSAI GLSIALHVGF DNFEQLLSGA 301 HWMDQHFRTT PLEKNAPVLL ALLGIWYINC FGCETHAMLP YDQYLHRFAA YFQQGDMESN GKYITKSGTR VDHQTGPIVW GEPGTNGQHA FYQLIHQGTK 401 MIPCDFLIPV QTQHPIRKGL HHKILLANFL AQTEALMRGK STEEARKELQ AAGKSPEDLE RLLPHKVFEG NRPTNSIVFT KLTPFMLGAL VAMYEHKIFV 501 QGIIWDINSF DQWGVELGKQ LAKKIEPELD GSAQVTSHDA STNGLINFIK QQREARVQ ``` | | |
| Evidence | Conclusive | | |
| Peptide list | 261, 373, 1006 | | |
| Peptides | 261 | Confidence | Green |
| Runs | 1, 2, 3, 4 |
| Relation | Unique |
| Proteins | G6PI\_HUMAN |
| Sequence | ``` SNTPILVDGK ``` |
| Position | 107 |
| PTMs | none |
| 373 | Confidence | Green |
| Runs | 1, 2, 3 |
| Relation | Unique |
| Proteins | G6PI\_HUMAN |
| Sequence | ``` TFTTQETITNAETAK ``` |
| Position | 212 |
| PTMs | none |
| 1006 | Confidence | Green |
| Runs | 1, 2, 3, 4 |
| Relation | Unique |
| Proteins | G6PI\_HUMAN |
| Sequence | ``` TLAQLNPESSLFIIASK ``` |
| Position | 195 |
| PTMs | none |

  

Protein P02765

| Name | FETUA\_HUMAN | | |
| Description | Alpha 2 HS glycoprotein OS Homo sapiens GN AHSG PE 1 SV 1 | | |
| Sequence | ```   1 MKSLVLLLCL AQLWGCHSAP HGPGLIYRQP NCDDPETEEA ALVAIDYINQ NLPWGYKHTL NQIDEVKVWP QQPSGELFEI EIDTLETTCH VLDPTPVARC 101 SVRQLKEHAV EGDCDFQLLK LDGKFSVVYA KCDSSPDSAE DVRKVCQDCP LLAPLNDTRV VHAAKAALAA FNAQNNGSNF QLEEISRAQL VPLPPSTYVE 201 FTVSGTDCVA KEATEAAKCN LLAEKQYGFC KATLSEKLGG AEVAVTCTVF QTQPVTSQPQ PEGANEAVPT PVVDPDAPPS PPLGAPGLPP AGSPPDSHVL 301 LAAPPGHQLH RAHYDLRHTF MGVVSLGSPS GEVSHPRKTR TVVQPSVGAA AGPVVPPCPG RIRHFKV ``` | | |
| Evidence | Conclusive | | |
| Peptide list | 15, 43, 193 | | |
| Peptides | 15 | Confidence | Green |
| Runs | 1, 2, 3, 4 |
| Relation | Unique |
| Proteins | FETUA\_HUMAN |
| Sequence | ``` CDSSPDSAEDVRK ``` |
| Position | 132 |
| PTMs | Carbamidomethyl+C(1) |
| 43 | Confidence | Green |
| Runs | 1, 2, 3, 4 |
| Relation | Unique |
| Proteins | FETUA\_HUMAN |
| Sequence | ``` CDSSPDSAEDVR ``` |
| Position | 132 |
| PTMs | Carbamidomethyl+C(1) |
| 193 | Confidence | Green |
| Runs | 1, 2, 3, 4 |
| Relation | Unique |
| Proteins | FETUA\_HUMAN |
| Sequence | ``` CNLLAEK ``` |
| Position | 219 |
| PTMs | Carbamidomethyl+C(1) |

  

Protein P05141

| Name | ADT2\_HUMAN | | |
| Description | ADP ATP translocase 2 OS Homo sapiens GN SLC25A5 PE 1 SV 7 | | |
| Sequence | ```   1 MTDAAVSFAK DFLAGGVAAA ISKTAVAPIE RVKLLLQVQH ASKQITADKQ YKGIIDCVVR IPKEQGVLSF WRGNLANVIR YFPTQALNFA FKDKYKQIFL 101 GGVDKRTQFW LYFAGNLASG GAAGATSLCF VYPLDFARTR LAADVGKAGA EREFRGLGDC LVKIYKSDGI KGLYQGFNVS VQGIIIYRAA YFGIYDTAKG 201 MLPDPKNTHI VISWMIAQTV TAVAGLTSYP FDTVRRRMMM QSGRKGTDIM YTGTLDCWRK IARDEGGKAF FKGAWSNVLR GMGGAFVLVL YDEIKKYT ``` | | |
| Evidence | Conclusive | | |
| Peptide list | 118\*\*, 528\*\*, 754, 864\*\*, 980\*\* | | |
| Peptides | 118\*\* | Confidence | Green |
| Runs | 1, 2, 3, 4 |
| Relation | NonDiscriminating |
| Proteins | ADT2\_HUMAN, ADT3\_HUMAN, ADT1\_HUMAN, ADT4\_HUMAN |
| Sequence | ``` TAVAPIER ``` |
| Position | 24 |
| PTMs | none |
| 528\*\* | Confidence | Green |
| Runs | 1, 2, 4 |
| Relation | NonDiscriminating |
| Proteins | ADT2\_HUMAN, ADT3\_HUMAN, ADT1\_HUMAN |
| Sequence | ``` GAWSNVLR ``` |
| Position | 273 |
| PTMs | none |
| 754 | Confidence | Green |
| Runs | 1, 2, 3, 4 |
| Relation | Unique |
| Proteins | ADT2\_HUMAN |
| Sequence | ``` DFLAGGVAAAISK ``` |
| Position | 11 |
| PTMs | none |
| 864\*\* | Confidence | Green |
| Runs | 1, 2, 3, 4 |
| Relation | NonDiscriminating |
| Proteins | ADT2\_HUMAN, ADT3\_HUMAN |
| Sequence | ``` EQGVLSFWR ``` |
| Position | 64 |
| PTMs | none |
| 980\*\* | Confidence | Green |
| Runs | 1, 2, 3, 4 |
| Relation | NonDiscriminating |
| Proteins | ADT2\_HUMAN, ADT3\_HUMAN, ADT1\_HUMAN, ADT4\_HUMAN |
| Sequence | ``` YFPTQALNFAFK ``` |
| Position | 81 |
| PTMs | none |

  

Protein P50990

| Name | TCPQ\_HUMAN | | |
| Description | T complex protein 1 subunit theta OS Homo sapiens GN CCT8 PE 1 SV 4 | | |
| Sequence | ```   1 MALHVPKAPG FAQMLKEGAK HFSGLEEAVY RNIQACKELA QTTRTAYGPN GMNKMVINHL EKLFVTNDAA TILRELEVQH PAAKMIVMAS HMQEQEVGDG 101 TNFVLVFAGA LLELAEELLR IGLSVSEVIE GYEIACRKAH EILPNLVCCS AKNLRDIDEV SSLLRTSIMS KQYGNEVFLA KLIAQACVSI FPDSGHFNVD 201 NIRVCKILGS GISSSSVLHG MVFKKETEGD VTSVKDAKIA VYSCPFDGMI TETKGTVLIK TAEELMNFSK GEENLMDAQV KAIADTGANV VVTGGKVADM 301 ALHYANKYNI MLVRLNSKWD LRRLCKTVGA TALPRLTPPV LEEMGHCDSV YLSEVGDTQV VVFKHEKEDG AISTIVLRGS TDNLMDDIER AVDDGVNTFK 401 VLTRDKRLVP GGGATEIELA KQITSYGETC PGLEQYAIKK FAEAFEAIPR ALAENSGVKA NEVISKLYAV HQEGNKNVGL DIEAEVPAVK DMLEAGILDT 501 YLGKYWAIKL ATNAAVTVLR VDQIIMAKPA GGPKPPSGKK DWDDDQND ``` | | |
| Evidence | Conclusive | | |
| Peptide list | 660, 757 | | |
| Peptides | 660 | Confidence | Green |
| Runs | 1, 2, 3 |
| Relation | Unique |
| Proteins | TCPQ\_HUMAN |
| Sequence | ``` FAEAFEAIPR ``` |
| Position | 441 |
| PTMs | none |
| 757 | Confidence | Green |
| Runs | 1, 2, 3 |
| Relation | Unique |
| Proteins | TCPQ\_HUMAN |
| Sequence | ``` LFVTNDAATILR ``` |
| Position | 63 |
| PTMs | none |

  

Protein P34932

| Name | HSP74\_HUMAN | | |
| Description | Heat shock 70 kDa protein 4 OS Homo sapiens GN HSPA4 PE 1 SV 4 | | |
| Sequence | ```   1 MSVVGIDLGF QSCYVAVARA GGIETIANEY SDRCTPACIS FGPKNRSIGA AAKSQVISNA KNTVQGFKRF HGRAFSDPFV EAEKSNLAYD IVQLPTGLTG 101 IKVTYMEEER NFTTEQVTAM LLSKLKETAE SVLKKPVVDC VVSVPCFYTD AERRSVMDAT QIAGLNCLRL MNETTAVALA YGIYKQDLPA LEEKPRNVVF 201 VDMGHSAYQV SVCAFNRGKL KVLATAFDTT LGGRKFDEVL VNHFCEEFGK KYKLDIKSKI RALLRLSQEC EKLKKLMSAN ASDLPLSIEC FMNDVDVSGT 301 MNRGKFLEMC NDLLARVEPP LRSVLEQTKL KKEDIYAVEI VGGATRIPAV KEKISKFFGK ELSTTLNADE AVTRGCALQC AILSPAFKVR EFSITDVVPY 401 PISLRWNSPA EEGSSDCEVF SKNHAAPFSK VLTFYRKEPF TLEAYYSSPQ DLPYPDPAIA QFSVQKVTPQ SDGSSSKVKV KVRVNVHGIF SVSSASLVEV 501 HKSEENEEPM ETDQNAKEEE KMQVDQEEPH VEEQQQQTPA ENKAESEEME TSQAGSKDKK MDQPPQAKKA KVKTSTVDLP IENQLLWQID REMLNLYIEN 601 EGKMIMQDKL EKERNDAKNA VEEYVYEMRD KLSGEYEKFV SEDDRNSFTL KLEDTENWLY EDGEDQPKQV YVDKLAELKN LGQPIKIRFQ ESEERPKLFE 701 ELGKQIQQYM KIISSFKNKE DQYDHLDAAD MTKVEKSTNE AMEWMNNKLN LQNKQSLTMD PVVKSKEIEA KIKELTSTCS PIISKPKPKV EPPKEEQKNA 801 EQNGPVDGQG DNPGPQAAEQ GTDTAVPSDS DKKLPEMDID ``` | | |
| Evidence | Conclusive | | |
| Peptide list | 1033, 1071 | | |
| Peptides | 1033 | Confidence | Green |
| Runs | 1, 2, 3 |
| Relation | Unique |
| Proteins | HSP74\_HUMAN |
| Sequence | ``` SNLAYDIVQLPTGLTGIK ``` |
| Position | 85 |
| PTMs | Variant #1: none Variant #2: Deamidation+N(2) |
| 1071 | Confidence | Green |
| Runs | 1, 2, 3, 4 |
| Relation | Unique |
| Proteins | HSP74\_HUMAN |
| Sequence | ``` EFSITDVVPYPISLR ``` |
| Position | 391 |
| PTMs | none |

  

Protein P49411

| Name | EFTU\_HUMAN | | |
| Description | Elongation factor Tu mitochondrial OS Homo sapiens GN TUFM PE 1 SV 2 | | |
| Sequence | ```   1 MAAATLLRAT PHFSGLAAGR TFLLQGLLRL LKAPALPLLC RGLAVEAKKT YVRDKPHVNV GTIGHVDHGK TTLTAAITKI LAEGGGAKFK KYEEIDNAPE 101 ERARGITINA AHVEYSTAAR HYAHTDCPGH ADYVKNMITG TAPLDGCILV VAANDGPMPQ TREHLLLARQ IGVEHVVVYV NKADAVQDSE MVELVELEIR 201 ELLTEFGYKG EETPVIVGSA LCALEGRDPE LGLKSVQKLL DAVDTYIPVP ARDLEKPFLL PVEAVYSVPG RGTVVTGTLE RGILKKGDEC ELLGHSKNIR 301 TVVTGIEMFH KSLERAEAGD NLGALVRGLK REDLRRGLVM VKPGSIKPHQ KVEAQVYILS KEEGGRHKPF VSHFMPVMFS LTWDMACRII LPPEKELAMP 401 GEDLKFNLIL RQPMILEKGQ RFTLRDGNRT IGTGLVTNTL AMTEEEKNIK WG ``` | | |
| Evidence | Conclusive | | |
| Peptide list | 820, 1018 | | |
| Peptides | 820 | Confidence | Green |
| Runs | 1, 2, 4 |
| Relation | Unique |
| Proteins | EFTU\_HUMAN |
| Sequence | ``` LLDAVDTYIPVPAR ``` |
| Position | 239 |
| PTMs | none |
| 1018 | Confidence | Green |
| Runs | 1, 2, 3, 4 |
| Relation | Unique |
| Proteins | EFTU\_HUMAN |
| Sequence | ``` DLEKPFLLPVEAVYSVPGR ``` |
| Position | 253 |
| PTMs | none |

  

Protein P13797

| Name | PLST\_HUMAN | | |
| Description | Plastin 3 OS Homo sapiens GN PLS3 PE 1 SV 4 | | |
| Sequence | ```   1 MDEMATTQIS KDELDELKEA FAKVDLNSNG FICDYELHEL FKEANMPLPG YKVREIIQKL MLDGDRNKDG KISFDEFVYI FQEVKSSDIA KTFRKAINRK 101 EGICALGGTS ELSSEGTQHS YSEEEKYAFV NWINKALEND PDCRHVIPMN PNTDDLFKAV GDGIVLCKMI NLSVPDTIDE RAINKKKLTP FIIQENLNLA 201 LNSASAIGCH VVNIGAEDLR AGKPHLVLGL LWQIIKIGLF ADIELSRNEA LAALLRDGET LEELMKLSPE ELLLRWANFH LENSGWQKIN NFSADIKDSK 301 AYFHLLNQIA PKGQKEGEPR IDINMSGFNE TDDLKRAESM LQQADKLGCR QFVTPADVVS GNPKLNLAFV ANLFNKYPAL TKPENQDIDW TLLEGETREE 401 RTFRNWMNSL GVNPHVNHLY ADLQDALVIL QLYERIKVPV DWSKVNKPPY PKLGANMKKL ENCNYAVELG KHPAKFSLVG IGGQDLNDGN QTLTLALVWQ 501 LMRRYTLNVL EDLGDGQKAN DDIIVNWVNR TLSEAGKSTS IQSFKDKTIS SSLAVVDLID AIQPGCINYD LVKSGNLTED DKHNNAKYAV SMARRIGARV 601 YALPEDLVEV KPKMVMTVFA CLMGRGMKRV ``` | | |
| Evidence | Conclusive | | |
| Peptide list | 21\*\*, 717, 1112, 1150, 1543\*\* | | |
| Peptides | 21\*\* | Confidence | Green |
| Runs | 1, 2, 3, 4 |
| Relation | NonDiscriminating |
| Proteins | PLST\_HUMAN, PLSL\_HUMAN |
| Sequence | ``` ALENDPDCR ``` |
| Position | 136 |
| PTMs | Carbamidomethyl+C(8) |
| 717 | Confidence | Green |
| Runs | 1, 2, 3, 4 |
| Relation | Unique |
| Proteins | PLST\_HUMAN |
| Sequence | ``` NEALAALLR ``` |
| Position | 248 |
| PTMs | none |
| 1112 | Confidence | Green |
| Runs | 1, 2, 4 |
| Relation | Unique |
| Proteins | PLST\_HUMAN |
| Sequence | ``` LNLAFVANLFNK ``` |
| Position | 365 |
| PTMs | Variant #1: none Variant #2: Deamidation+N(8) |
| 1150 | Confidence | Green |
| Runs | 1, 2, 3 |
| Relation | Unique |
| Proteins | PLST\_HUMAN |
| Sequence | ``` TISSSLAVVDLIDAIQPGCINYDLVK ``` |
| Position | 548 |
| PTMs | Carbamidomethyl+C(19) |
| 1543\*\* | Confidence | Green |
| Runs | 2, 3, 4 |
| Relation | NonDiscriminating |
| Proteins | PLST\_HUMAN, PLSL\_HUMAN, PLSI\_HUMAN |
| Sequence | ``` LSPEELLLR ``` |
| Position | 267 |
| PTMs | none |

  

Protein Q14974

| Name | IMB1\_HUMAN | | |
| Description | Importin subunit beta 1 OS Homo sapiens GN KPNB1 PE 1 SV 2 | | |
| Sequence | ```   1 MELITILEKT VSPDRLELEA AQKFLERAAV ENLPTFLVEL SRVLANPGNS QVARVAAGLQ IKNSLTSKDP DIKAQYQQRW LAIDANARRE VKNYVLQTLG 101 TETYRPSSAS QCVAGIACAE IPVNQWPELI PQLVANVTNP NSTEHMKEST LEAIGYICQD IDPEQLQDKS NEILTAIIQG MRKEEPSNNV KLAATNALLN 201 SLEFTKANFD KESERHFIMQ VVCEATQCPD TRVRVAALQN LVKIMSLYYQ YMETYMGPAL FAITIEAMKS DIDEVALQGI EFWSNVCDEE MDLAIEASEA 301 AEQGRPPEHT SKFYAKGALQ YLVPILTQTL TKQDENDDDD DWNPCKAAGV CLMLLATCCE DDIVPHVLPF IKEHIKNPDW RYRDAAVMAF GCILEGPEPS 401 QLKPLVIQAM PTLIELMKDP SVVVRDTAAW TVGRICELLP EAAINDVYLA PLLQCLIEGL SAEPRVASNV CWAFSSLAEA AYEAADVADD QEEPATYCLS 501 SSFELIVQKL LETTDRPDGH QNNLRSSAYE SLMEIVKNSA KDCYPAVQKT TLVIMERLQQ VLQMESHIQS TSDRIQFNDL QSLLCATLQN VLRKVQHQDA 601 LQISDVVMAS LLRMFQSTAG SGGVQEDALM AVSTLVEVLG GEFLKYMEAF KPFLGIGLKN YAEYQVCLAA VGLVGDLCRA LQSNIIPFCD EVMQLLLENL 701 GNENVHRSVK PQILSVFGDI ALAIGGEFKK YLEVVLNTLQ QASQAQVDKS DYDMVDYLNE LRESCLEAYT GIVQGLKGDQ ENVHPDVMLV QPRVEFILSF 801 IDHIAGDEDH TDGVVACAAG LIGDLCTAFG KDVLKLVEAR PMIHELLTEG RRSKTNKAKT LATWATKELR KLKNQA ``` | | |
| Evidence | Conclusive | | |
| Peptide list | 1062, 1106, 1149, 1229 | | |
| Peptides | 1062 | Confidence | Green |
| Runs | 1, 2, 3, 4 |
| Relation | Unique |
| Proteins | IMB1\_HUMAN |
| Sequence | ``` SNEILTAIIQGMR ``` |
| Position | 170 |
| PTMs | none |
| 1106 | Confidence | Green |
| Runs | 1, 2, 4 |
| Relation | Unique |
| Proteins | IMB1\_HUMAN |
| Sequence | ``` AAVENLPTFLVELSR ``` |
| Position | 28 |
| PTMs | none |
| 1149 | Confidence | Green |
| Runs | 1, 2, 3, 4 |
| Relation | Unique |
| Proteins | IMB1\_HUMAN |
| Sequence | ``` GALQYLVPILTQTLTK ``` |
| Position | 317 |
| PTMs | none |
| 1229 | Confidence | Green |
| Runs | 2, 3, 4 |
| Relation | Unique |
| Proteins | IMB1\_HUMAN |
| Sequence | ``` VLANPGNSQVAR ``` |
| Position | 43 |
| PTMs | none |

  

Protein P31948

| Name | STIP1\_HUMAN | | |
| Description | Stress induced phosphoprotein 1 OS Homo sapiens GN STIP1 PE 1 SV 1 | | |
| Sequence | ```   1 MEQVNELKEK GNKALSVGNI DDALQCYSEA IKLDPHNHVL YSNRSAAYAK KGDYQKAYED GCKTVDLKPD WGKGYSRKAA ALEFLNRFEE AKRTYEEGLK 101 HEANNPQLKE GLQNMEARLA ERKFMNPFNM PNLYQKLESD PRTRTLLSDP TYRELIEQLR NKPSDLGTKL QDPRIMTTLS VLLGVDLGSM DEEEEIATPP 201 PPPPPKKETK PEPMEEDLPE NKKQALKEKE LGNDAYKKKD FDTALKHYDK AKELDPTNMT YITNQAAVYF EKGDYNKCRE LCEKAIEVGR ENREDYRQIA 301 KAYARIGNSY FKEEKYKDAI HFYNKSLAEH RTPDVLKKCQ QAEKILKEQE RLAYINPDLA LEEKNKGNEC FQKGDYPQAM KHYTEAIKRN PKDAKLYSNR 401 AACYTKLLEF QLALKDCEEC IQLEPTFIKG YTRKAAALEA MKDYTKAMDV YQKALDLDSS CKEAADGYQR CMMAQYNRHD SPEDVKRRAM ADPEVQQIMS 501 DPAMRLILEQ MQKDPQALSE HLKNPVIAQK IQKLMDVGLI AIR ``` | | |
| Evidence | Conclusive | | |
| Peptide list | 138, 215, 645 | | |
| Peptides | 138 | Confidence | Green |
| Runs | 1, 2, 4 |
| Relation | Unique |
| Proteins | STIP1\_HUMAN |
| Sequence | ``` LDPHNHVLYSNR ``` |
| Position | 33 |
| PTMs | none |
| 215 | Confidence | Yellow |
| Runs | 1, 2, 4 |
| Relation | Unique |
| Proteins | STIP1\_HUMAN |
| Sequence | ``` DPQALSEHLK ``` |
| Position | 514 |
| PTMs | none |
| 645 | Confidence | Green |
| Runs | 1, 2, 4 |
| Relation | Unique |
| Proteins | STIP1\_HUMAN |
| Sequence | ``` AAALEFLNR ``` |
| Position | 79 |
| PTMs | none |

  

Protein P09874

| Name | PARP1\_HUMAN | | |
| Description | Poly ADP ribose polymerase 1 OS Homo sapiens GN PARP1 PE 1 SV 4 | | |
| Sequence | ```    1 MAESSDKLYR VEYAKSGRAS CKKCSESIPK DSLRMAIMVQ SPMFDGKVPH WYHFSCFWKV GHSIRHPDVE VDGFSELRWD DQQKVKKTAE AGGVTGKGQD  101 GIGSKAEKTL GDFAAEYAKS NRSTCKGCME KIEKGQVRLS KKMVDPEKPQ LGMIDRWYHP GCFVKNREEL GFRPEYSASQ LKGFSLLATE DKEALKKQLP  201 GVKSEGKRKG DEVDGVDEVA KKKSKKEKDK DSKLEKALKA QNDLIWNIKD ELKKVCSTND LKELLIFNKQ QVPSGESAIL DRVADGMVFG ALLPCEECSG  301 QLVFKSDAYY CTGDVTAWTK CMVKTQTPNR KEWVTPKEFR EISYLKKLKV KKQDRIFPPE TSASVAATPP PSTASAPAAV NSSASADKPL SNMKILTLGK  401 LSRNKDEVKA MIEKLGGKLT GTANKASLCI STKKEVEKMN KKMEEVKEAN IRVVSEDFLQ DVSASTKSLQ ELFLAHILSP WGAEVKAEPV EVVAPRGKSG  501 AALSKKSKGQ VKEEGINKSE KRMKLTLKGG AAVDPDSGLE HSAHVLEKGG KVFSATLGLV DIVKGTNSYY KLQLLEDDKE NRYWIFRSWG RVGTVIGSNK  601 LEQMPSKEDA IEHFMKLYEE KTGNAWHSKN FTKYPKKFYP LEIDYGQDEE AVKKLTVNPG TKSKLPKPVQ DLIKMIFDVE SMKKAMVEYE IDLQKMPLGK  701 LSKRQIQAAY SILSEVQQAV SQGSSDSQIL DLSNRFYTLI PHDFGMKKPP LLNNADSVQA KVEMLDNLLD IEVAYSLLRG GSDDSSKDPI DVNYEKLKTD  801 IKVVDRDSEE AEIIRKYVKN THATTHNAYD LEVIDIFKIE REGECQRYKP FKQLHNRRLL WHGSRTTNFA GILSQGLRIA PPEAPVTGYM FGKGIYFADM  901 VSKSANYCHT SQGDPIGLIL LGEVALGNMY ELKHASHISK LPKGKHSVKG LGKTTPDPSA NISLDGVDVP LGTGISSGVN DTSLLYNEYI VYDIAQVNLK 1001 YLLKLKFNFK TSLW ``` | | |
| Evidence | Conclusive | | |
| Peptide list | 277, 875 | | |
| Peptides | 277 | Confidence | Green |
| Runs | 1, 2, 3, 4 |
| Relation | Unique |
| Proteins | PARP1\_HUMAN |
| Sequence | ``` AEPVEVVAPR ``` |
| Position | 487 |
| PTMs | none |
| 875 | Confidence | Green |
| Runs | 1, 2, 3, 4 |
| Relation | Unique |
| Proteins | PARP1\_HUMAN |
| Sequence | ``` TTNFAGILSQGLR ``` |
| Position | 866 |
| PTMs | none |

  

Protein Q08211

| Name | DHX9\_HUMAN | | |
| Description | ATP dependent RNA helicase A OS Homo sapiens GN DHX9 PE 1 SV 4 | | |
| Sequence | ```    1 MGDVKNFLYA WCGKRKMTPS YEIRAVGNKN RQKFMCEVQV EGYNYTGMGN STNKKDAQSN AARDFVNYLV RINEIKSEEV PAFGVASPPP LTDTPDTTAN  101 AEGDLPTTMG GPLPPHLALK AENNSEVGAS GYGVPGPTWD RGANLKDYYS RKEEQEVQAT LESEEVDLNA GLHGNWTLEN AKARLNQYFQ KEKIQGEYKY  201 TQVGPDHNRS FIAEMTIYIK QLGRRIFARE HGSNKKLAAQ SCALSLVRQL YHLGVVEAYS GLTKKKEGET VEPYKVNLSQ DLEHQLQNII QELNLEILPP  301 PEDPSVPVAL NIGKLAQFEP SQRQNQVGVV PWSPPQSNWN PWTSSNIDEG PLAFATPEQI SMDLKNELMY QLEQDHDLQA ILQERELLPV KKFESEILEA  401 ISQNSVVIIR GATGCGKTTQ VPQFILDDFI QNDRAAECNI VVTQPRRISA VSVAERVAFE RGEEPGKSCG YSVRFESILP RPHASIMFCT VGVLLRKLEA  501 GIRGISHVIV DEIHERDINT DFLLVVLRDV VQAYPEVRIV LMSATIDTSM FCEYFFNCPI IEVYGRTYPV QEYFLEDCIQ MTHFVPPPKD KKKKDKDDDG  601 GEDDDANCNL ICGDEYGPET RLSMSQLNEK ETPFELIEAL LKYIETLNVP GAVLVFLPGW NLIYTMQKHL EMNPHFGSHR YQILPLHSQI PREEQRKVFD  701 PVPVGVTKVI LSTNIAETSI TINDVVYVID SCKQKVKLFT AHNNMTNYAT VWASKTNLEQ RKGRAGRVRP GFCFHLCSRA RFERLETHMT PEMFRTPLHE  801 IALSIKLLRL GGIGQFLAKA IEPPPLDAVI EAEHTLRELD ALDANDELTP LGRILAKLPI EPRFGKMMIM GCIFYVGDAI CTIAAATCFP EPFINEGKRL  901 GYIHRNFAGN RFSDHVALLS VFQAWDDARM GGEEAEIRFC EHKRLNMATL RMTWEAKVQL KEILINSGFP EDCLLTQVFT NTGPDNNLDV VISLLAFGVY 1001 PNVCYHKEKR KILTTEGRNA LIHKSSVNCP FSSQDMKYPS PFFVFGEKIR TRAISAKGMT LVTPLQLLLF ASKKVQSDGQ IVLVDDWIKL QISHEAAACI 1101 TGLRAAMEAL VVEVTKQPAI ISQLDPVNER MLNMIRQISR PSAAGINLMI GSTRYGDGPR PPKMARYDNG SGYRRGGSSY SGGGYGGGYS SGGYGSGGYG 1201 GSANSFRAGY GAGVGGGYRG VSRGGFRGNS GGDYRGPSGG YRGSGGFQRG GGRGAYGTGY FGQGRGGGGY ``` | | |
| Evidence | Conclusive | | |
| Peptide list | 965, 1154\*\* | | |
| Peptides | 965 | Confidence | Green |
| Runs | 1, 2, 3 |
| Relation | Unique |
| Proteins | DHX9\_HUMAN |
| Sequence | ``` AIEPPPLDAVIEAEHTLR ``` |
| Position | 820 |
| PTMs | none |
| 1154\*\* | Confidence | Green |
| Runs | 1, 2, 3 |
| Relation | NonDiscriminating |
| Proteins | DHX9\_HUMAN, DHX9\_HUMAN |
| Sequence | ``` GMTLVTPLQLLLFASK ``` |
| Position | 1058 |
| PTMs | none |

  

Protein P40227

| Name | TCPZ\_HUMAN | | |
| Description | T complex protein 1 subunit zeta OS Homo sapiens GN CCT6A PE 1 SV 3 | | |
| Sequence | ```   1 MAAVKTLNPK AEVARAQAAL AVNISAARGL QDVLRTNLGP KGTMKMLVSG AGDIKLTKDG NVLLHEMQIQ HPTASLIAKV ATAQDDITGD GTTSNVLIIG 101 ELLKQADLYI SEGLHPRIIT EGFEAAKEKA LQFLEEVKVS REMDRETLID VARTSLRTKV HAELADVLTE AVVDSILAIK KQDEPIDLFM IEIMEMKHKS 201 ETDTSLIRGL VLDHGARHPD MKKRVEDAYI LTCNVSLEYE KTEVNSGFFY KSAEEREKLV KAERKFIEDR VKKIIELKRK VCGDSDKGFV VINQKGIDPF 301 SLDALSKEGI VALRRAKRRN MERLTLACGG VALNSFDDLS PDCLGHAGLV YEYTLGEEKF TFIEKCNNPR SVTLLIKGPN KHTLTQIKDA VRDGLRAVKN 401 AIDDGCVVPG AGAVEVAMAE ALIKHKPSVK GRAQLGVQAF ADALLIIPKV LAQNSGFDLQ ETLVKIQAEH SESGQLVGVD LNTGEPMVAA EVGVWDNYCV 501 KKQLLHSCTV IATNILLVDE IMRAGMSSLK G ``` | | |
| Evidence | Conclusive | | |
| Peptide list | 1079 | | |
| Peptides | 1079 | Confidence | Green |
| Runs | 1, 2, 3, 4 |
| Relation | Unique |
| Proteins | TCPZ\_HUMAN |
| Sequence | ``` VATAQDDITGDGTTSNVLIIGELLK ``` |
| Position | 80 |
| PTMs | none |

  

Protein P43243

| Name | MATR3\_HUMAN | | |
| Description | Matrin 3 OS Homo sapiens GN MATR3 PE 1 SV 2 | | |
| Sequence | ```   1 MSKSFQQSSL SRDSQGHGRD LSAAGIGLLA AATQSLSMPA SLGRMNQGTA RLASLMNLGM SSSLNQQGAH SALSSASTSS HNLQSIFNIG SRGPLPLSSQ 101 HRGDADQASN ILASFGLSAR DLDELSRYPE DKITPENLPQ ILLQLKRRRT EEGPTLSYGR DGRSATREPP YRVPRDDWEE KRHFRRDSFD DRGPSLNPVL 201 DYDHGSRSQE SGYYDRMDYE DDRLRDGERC RDDSFFGETS HNYHKFDSEY ERMGRGPGPL QERSLFEKKR GAPPSSNIED FHGLLPKGYP HLCSICDLPV 301 HSNKEWSQHI NGASHSRRCQ LLLEIYPEWN PDNDTGHTMG DPFMLQQSTN PAPGILGPPP PSFHLGGPAV GPRGNLGAGN GNLQGPRHMQ KGRVETSRVV 401 HIMDFQRGKN LRYQLLQLVE PFGVISNHLI LNKINEAFIE MATTEDAQAA VDYYTTTPAL VFGKPVRVHL SQKYKRIKKP EGKPDQKFDQ KQELGRVIHL 501 SNLPHSGYSD SAVLKLAEPY GKIKNYILMR MKSQAFIEME TREDAMAMVD HCLKKALWFQ GRCVKVDLSE KYKKLVLRIP NRGIDLLKKD KSRKRSYSPD 601 GKESPSDKKS KTDGSQKTES STEGKEQEEK SGEDGEKDTK DDQTEQEPNM LLESEDELLV DEEEAAALLE SGSSVGDETD LANLGDVASD GKKEPSDKAV 701 KKDGSASAAA KKKLKKVDKI EELDQENEAA LENGIKNEEN TEPGAESSEN ADDPNKDTSE NADGQSDENK DDYTIPDEYR IGPYQPNVPV GIDYVIPKTG 801 FYCKLCSLFY TNEEVAKNTH CSSLPHYQKL KKFLNKLAEE RRQKKET ``` | | |
| Evidence | Conclusive | | |
| Peptide list | 1107 | | |
| Peptides | 1107 | Confidence | Green |
| Runs | 1, 2, 3, 4 |
| Relation | Unique |
| Proteins | MATR3\_HUMAN |
| Sequence | ``` ITPENLPQILLQLK ``` |
| Position | 133 |
| PTMs | none |

  

Protein P38646

| Name | GRP75\_HUMAN | | |
| Description | Stress 70 protein mitochondrial OS Homo sapiens GN HSPA9 PE 1 SV 2 | | |
| Sequence | ```   1 MISASRAAAA RLVGAAASRG PTAARHQDSW NGLSHEAFRL VSRRDYASEA IKGAVVGIDL GTTNSCVAVM EGKQAKVLEN AEGARTTPSV VAFTADGERL 101 VGMPAKRQAV TNPNNTFYAT KRLIGRRYDD PEVQKDIKNV PFKIVRASNG DAWVEAHGKL YSPSQIGAFV LMKMKETAEN YLGHTAKNAV ITVPAYFNDS 201 QRQATKDAGQ ISGLNVLRVI NEPTAAALAY GLDKSEDKVI AVYDLGGGTF DISILEIQKG VFEVKSTNGD TFLGGEDFDQ ALLRHIVKEF KRETGVDLTK 301 DNMALQRVRE AAEKAKCELS SSVQTDINLP YLTMDSSGPK HLNMKLTRAQ FEGIVTDLIR RTIAPCQKAM QDAEVSKSDI GEVILVGGMT RMPKVQQTVQ 401 DLFGRAPSKA VNPDEAVAIG AAIQGGVLAG DVTDVLLLDV TPLSLGIETL GGVFTKLINR NTTIPTKKSQ VFSTAADGQT QVEIKVCQGE REMAGDNKLL 501 GQFTLIGIPP APRGVPQIEV TFDIDANGIV HVSAKDKGTG REQQIVIQSS GGLSKDDIEN MVKNAEKYAE EDRRKKERVE AVNMAEGIIH DTETKMEEFK 601 DQLPADECNK LKEEISKMRE LLARKDSETG ENIRQAASSL QQASLKLFEM AYKKMASERE GSGSSGTGEQ KEDQKEEKQ ``` | | |
| Evidence | Conclusive | | |
| Peptide list | 27, 653, 1297, 1607 | | |
| Peptides | 27 | Confidence | Green |
| Runs | 1, 2, 3, 4 |
| Relation | Unique |
| Proteins | GRP75\_HUMAN |
| Sequence | ``` VLENAEGAR ``` |
| Position | 77 |
| PTMs | none |
| 653 | Confidence | Green |
| Runs | 1, 2, 3, 4 |
| Relation | Unique |
| Proteins | GRP75\_HUMAN |
| Sequence | ``` DAGQISGLNVLR ``` |
| Position | 207 |
| PTMs | none |
| 1297 | Confidence | Green |
| Runs | 2, 3, 4 |
| Relation | Unique |
| Proteins | GRP75\_HUMAN |
| Sequence | ``` QAVTNPNNTFYATK ``` |
| Position | 108 |
| PTMs | Variant #1: none Variant #2: Deamidation+N(5) |
| 1607 | Confidence | Green |
| Runs | 2, 3, 4 |
| Relation | Unique |
| Proteins | GRP75\_HUMAN |
| Sequence | ``` AQFEGIVTDLIR ``` |
| Position | 349 |
| PTMs | none |

  

Protein P78371

| Name | TCPB\_HUMAN | | |
| Description | T complex protein 1 subunit beta OS Homo sapiens GN CCT2 PE 1 SV 4 | | |
| Sequence | ```   1 MASLSLAPVN IFKAGADEER AETARLTSFI GAIAIGDLVK STLGPKGMDK ILLSSGRDAS LMVTNDGATI LKNIGVDNPA AKVLVDMSRV QDDEVGDGTT 101 SVTVLAAELL REAESLIAKK IHPQTIIAGW REATKAAREA LLSSAVDHGS DEVKFRQDLM NIAGTTLSSK LLTHHKDHFT KLAVEAVLRL KGSGNLEAIH 201 IIKKLGGSLA DSYLDEGFLL DKKIGVNQPK RIENAKILIA NTGMDTDKIK IFGSRVRVDS TAKVAEIEHA EKEKMKEKVE RILKHGINCF INRQLIYNYP 301 EQLFGAAGVM AIEHADFAGV ERLALVTGGE IASTFDHPEL VKLGSCKLIE EVMIGEDKLI HFSGVALGEA CTIVLRGATQ QILDEAERSL HDALCVLAQT 401 VKDSRTVYGG GCSEMLMAHA VTQLANRTPG KEAVAMESYA KALRMLPTII ADNAGYDSAD LVAQLRAAHS EGNTTAGLDM REGTIGDMAI LGITESFQVK 501 RQVLLSAAEA AEVILRVDNI IKAAPRKRVP DHHPC ``` | | |
| Evidence | Conclusive | | |
| Peptide list | 1685 | | |
| Peptides | 1685 | Confidence | Green |
| Runs | 2, 3, 4 |
| Relation | Unique |
| Proteins | TCPB\_HUMAN |
| Sequence | ``` VQDDEVGDGTTSVTVLAAELLR ``` |
| Position | 90 |
| PTMs | none |

  

Protein P13010

| Name | XRCC5\_HUMAN | | |
| Description | X ray repair cross complementing protein 5 OS Homo sapiens GN XRCC5 PE 1 SV 3 | | |
| Sequence | ```   1 MVRSGNKAAV VLCMDVGFTM SNSIPGIESP FEQAKKVITM FVQRQVFAEN KDEIALVLFG TDGTDNPLSG GDQYQNITVH RHLMLPDFDL LEDIESKIQP 101 GSQQADFLDA LIVSMDVIQH ETIGKKFEKR HIEIFTDLSS RFSKSQLDII IHSLKKCDIS LQFFLPFSLG KEDGSGDRGD GPFRLGGHGP SFPLKGITEQ 201 QKEGLEIVKM VMISLEGEDG LDEIYSFSES LRKLCVFKKI ERHSIHWPCR LTIGSNLSIR IAAYKSILQE RVKKTWTVVD AKTLKKEDIQ KETVYCLNDD 301 DETEVLKEDI IQGFRYGSDI VPFSKVDEEQ MKYKSEGKCF SVLGFCKSSQ VQRRFFMGNQ VLKVFAARDD EAAAVALSSL IHALDDLDMV AIVRYAYDKR 401 ANPQVGVAFP HIKHNYECLV YVQLPFMEDL RQYMFSSLKN SKKYAPTEAQ LNAVDALIDS MSLAKKDEKT DTLEDLFPTT KIPNPRFQRL FQCLLHRALH 501 PREPLPPIQQ HIWNMLNPPA EVTTKSQIPL SKIKTLFPLI EAKKKDQVTA QEIFQDNHED GPTAKKLKTE QGGAHFSVSS LAEGSVTSVG SVNPAENFRV 601 LVKQKKASFE EASNQLINHI EQFLDTNETP YFMKSIDCIR AFREEAIKFS EEQRFNNFLK ALQEKVEIKQ LNHFWEIVVQ DGITLITKEE ASGSSVTAEE 701 AKKFLAPKDK PSGDTAAVFE EGGDVDDLLD MI ``` | | |
| Evidence | Conclusive | | |
| Peptide list | 31 | | |
| Peptides | 31 | Confidence | Green |
| Runs | 1, 2, 3 |
| Relation | Unique |
| Proteins | XRCC5\_HUMAN |
| Sequence | ``` EEASGSSVTAEEAK ``` |
| Position | 689 |
| PTMs | none |

  

Protein Q99832

| Name | TCPH\_HUMAN | | |
| Description | T complex protein 1 subunit eta OS Homo sapiens GN CCT7 PE 1 SV 2 | | |
| Sequence | ```   1 MMPTPVILLK EGTDSSQGIP QLVSNISACQ VIAEAVRTTL GPRGMDKLIV DGRGKATISN DGATILKLLD VVHPAAKTLV DIAKSQDAEV GDGTTSVTLL 101 AAEFLKQVKP YVEEGLHPQI IIRAFRTATQ LAVNKIKEIA VTVKKADKVE QRKLLEKCAM TALSSKLISQ QKAFFAKMVV DAVMMLDDLL QLKMIGIKKV 201 QGGALEDSQL VAGVAFKKTF SYAGFEMQPK KYHNPKIALL NVELELKAEK DNAEIRVHTV EDYQAIVDAE WNILYDKLEK IHHSGAKVVL SKLPIGDVAT 301 QYFADRDMFC AGRVPEEDLK RTMMACGGSI QTSVNALSAD VLGRCQVFEE TQIGGERYNF FTGCPKAKTC TFILRGGAEQ FMEETERSLH DAIMIVRRAI 401 KNDSVVAGGG AIEMELSKYL RDYSRTIPGK QQLLIGAYAK ALEIIPRQLC DNAGFDATNI LNKLRARHAQ GGTWYGVDIN NEDIADNFEA FVWEPAMVRI 501 NALTAASEAA CLIVSVDETI KNPRSTVDAP TAAGRGRGRG RPH ``` | | |
| Evidence | Conclusive | | |
| Peptide list | 74 | | |
| Peptides | 74 | Confidence | Yellow |
| Runs | 1, 3, 4 |
| Relation | Unique |
| Proteins | TCPH\_HUMAN |
| Sequence | ``` TATQLAVNK ``` |
| Position | 127 |
| PTMs | none |

  

Protein Q07021

| Name | C1QBP\_HUMAN | | |
| Description | Complement component 1 Q subcomponent binding protein mitochondrial OS Homo sapiens GN C1QBP PE 1 S | | |
| Sequence | ```   1 MLPLLRCVPR VLGSSVAGLR AAAPASPFRQ LLQPAPRLCT RPFGLLSVRA GSERRPGLLR PRGPCACGCG CGSLHTDGDK AFVDFLSDEI KEERKIQKHK 101 TLPKMSGGWE LELNGTEAKL VRKVAGEKIT VTFNINNSIP PTFDGEEEPS QGQKVEEQEP ELTSTPNFVV EVIKNDDGKK ALVLDCHYPE DEVGQEDEAE 201 SDIFSIREVS FQSTGESEWK DTNYTLNTDS LDWALYDHLM DFLADRGVDN TFADELVELS TALEHQEYIT FLEDLKSFVK SQ ``` | | |
| Evidence | Conclusive | | |
| Peptide list | 1602, 1603 | | |
| Peptides | 1602 | Confidence | Green |
| Runs | 2, 3, 4 |
| Relation | Unique |
| Proteins | C1QBP\_HUMAN |
| Sequence | ``` AFVDFLSDEIK ``` |
| Position | 81 |
| PTMs | none |
| 1603 | Confidence | Green |
| Runs | 2, 3, 4 |
| Relation | Unique |
| Proteins | C1QBP\_HUMAN |
| Sequence | ``` VEEQEPELTSTPNFVVEVIK ``` |
| Position | 155 |
| PTMs | none |

  

Protein P0CG48

| Name | UBC\_HUMAN | | |
| Description | Polyubiquitin C OS Homo sapiens GN UBC PE 1 SV 2 | | |
| Sequence | ```   1 MQIFVKTLTG KTITLEVEPS DTIENVKAKI QDKEGIPPDQ QRLIFAGKQL EDGRTLSDYN IQKESTLHLV LRLRGGMQIF VKTLTGKTIT LEVEPSDTIE 101 NVKAKIQDKE GIPPDQQRLI FAGKQLEDGR TLSDYNIQKE STLHLVLRLR GGMQIFVKTL TGKTITLEVE PSDTIENVKA KIQDKEGIPS DQQRLIFAGK 201 QLEDGRTLSD YNIQKESTLH LVLRLRGGMQ IFVKTLTGKT ITLEVEPSDT IENVKAKIQD KEGIPPDQQR LIFAGKQLED GRTLSDYNIQ KESTLHLVLR 301 LRGGMQIFVK TLTGKTITLE VEPSDTIENV KAKIQDKEGI PPDQQRLIFA GKQLEDGRTL SDYNIQKEST LHLVLRLRGG MQIFVKTLTG KTITLEVEPS 401 DTIENVKAKI QDKEGIPPDQ QRLIFAGKQL EDGRTLSDYN IQKESTLHLV LRLRGGMQIF VKTLTGKTIT LEVEPSDTIE NVKAKIQDKE GIPPDQQRLI 501 FAGKQLEDGR TLSDYNIQKE STLHLVLRLR GGMQIFVKTL TGKTITLEVE PSDTIENVKA KIQDKEGIPP DQQRLIFAGK QLEDGRTLSD YNIQKESTLH 601 LVLRLRGGMQ IFVKTLTGKT ITLEVEPSDT IENVKAKIQD KEGIPPDQQR LIFAGKQLED GRTLSDYNIQ KESTLHLVLR LRGGV ``` | | |
| Evidence | Indistinguishable | | |
| Peptide list | 24\*, 238\*, 439\*, 487\*, 565\* | | |
| Peptides | 24\* | Confidence | Green |
| Runs | 1, 2, 3 |
| Relation | Discriminating |
| Proteins | RL40\_HUMAN, UBC\_HUMAN, UBB\_HUMAN, RS27A\_HUMAN |
| Sequence | ``` EGIPPDQQR ``` |
| Position | 34, 110, 262, 338, 414, 490, 566, 642 |
| PTMs | none |
| 238\* | Confidence | Green |
| Runs | 1, 3, 4 |
| Relation | Discriminating |
| Proteins | RL40\_HUMAN, UBC\_HUMAN, UBB\_HUMAN, RS27A\_HUMAN |
| Sequence | ``` TLSDYNIQK ``` |
| Position | 55, 131, 207, 283, 359, 435, 511, 587, 663 |
| PTMs | none |
| 439\* | Confidence | Green |
| Runs | 1, 2, 3 |
| Relation | Discriminating |
| Proteins | RL40\_HUMAN, UBC\_HUMAN, UBB\_HUMAN, RS27A\_HUMAN |
| Sequence | ``` MQIFVK ``` |
| Position | 1, 77, 153, 229, 305, 381, 457, 533, 609 |
| PTMs | none |
| 487\* | Confidence | Green |
| Runs | 1, 2, 3, 4 |
| Relation | Discriminating |
| Proteins | RL40\_HUMAN, UBC\_HUMAN, UBB\_HUMAN, RS27A\_HUMAN |
| Sequence | ``` ESTLHLVLR ``` |
| Position | 64, 140, 216, 292, 368, 444, 520, 596, 672 |
| PTMs | none |
| 565\* | Confidence | Green |
| Runs | 1, 2, 3, 4 |
| Relation | Discriminating |
| Proteins | RL40\_HUMAN, UBC\_HUMAN, UBB\_HUMAN, RS27A\_HUMAN |
| Sequence | ``` TITLEVEPSDTIENVK ``` |
| Position | 12, 88, 164, 240, 316, 392, 468, 544, 620 |
| PTMs | Variant #1: Deamidation+N(14)  Variant #2: none |

  

Protein P62987

| Name | RL40\_HUMAN | | |
| Description | Ubiquitin 60S ribosomal protein L40 OS Homo sapiens GN UBA52 PE 1 SV 2 | | |
| Sequence | ```   1 MQIFVKTLTG KTITLEVEPS DTIENVKAKI QDKEGIPPDQ QRLIFAGKQL EDGRTLSDYN IQKESTLHLV LRLRGGIIEP SLRQLAQKYN CDKMICRKCY 101 ARLHPRAVNC RKKKCGHTNN LRPKKKVK ``` | | |
| Evidence | Indistinguishable | | |
| Peptide list | 24\*, 238\*, 439\*, 487\*, 565\* | | |
| Peptides | 24\* | Confidence | Green |
| Runs | 1, 2, 3 |
| Relation | Discriminating |
| Proteins | RL40\_HUMAN, UBC\_HUMAN, UBB\_HUMAN, RS27A\_HUMAN |
| Sequence | ``` EGIPPDQQR ``` |
| Position | 34 |
| PTMs | none |
| 238\* | Confidence | Green |
| Runs | 1, 3, 4 |
| Relation | Discriminating |
| Proteins | RL40\_HUMAN, UBC\_HUMAN, UBB\_HUMAN, RS27A\_HUMAN |
| Sequence | ``` TLSDYNIQK ``` |
| Position | 55 |
| PTMs | none |
| 439\* | Confidence | Green |
| Runs | 1, 2, 3 |
| Relation | Discriminating |
| Proteins | RL40\_HUMAN, UBC\_HUMAN, UBB\_HUMAN, RS27A\_HUMAN |
| Sequence | ``` MQIFVK ``` |
| Position | 1 |
| PTMs | none |
| 487\* | Confidence | Green |
| Runs | 1, 2, 3, 4 |
| Relation | Discriminating |
| Proteins | RL40\_HUMAN, UBC\_HUMAN, UBB\_HUMAN, RS27A\_HUMAN |
| Sequence | ``` ESTLHLVLR ``` |
| Position | 64 |
| PTMs | none |
| 565\* | Confidence | Green |
| Runs | 1, 2, 3, 4 |
| Relation | Discriminating |
| Proteins | RL40\_HUMAN, UBC\_HUMAN, UBB\_HUMAN, RS27A\_HUMAN |
| Sequence | ``` TITLEVEPSDTIENVK ``` |
| Position | 12 |
| PTMs | Variant #1: Deamidation+N(14)  Variant #2: none |

  

Protein P0CG47

| Name | UBB\_HUMAN | | |
| Description | Polyubiquitin B OS Homo sapiens GN UBB PE 1 SV 1 | | |
| Sequence | ```   1 MQIFVKTLTG KTITLEVEPS DTIENVKAKI QDKEGIPPDQ QRLIFAGKQL EDGRTLSDYN IQKESTLHLV LRLRGGMQIF VKTLTGKTIT LEVEPSDTIE 101 NVKAKIQDKE GIPPDQQRLI FAGKQLEDGR TLSDYNIQKE STLHLVLRLR GGMQIFVKTL TGKTITLEVE PSDTIENVKA KIQDKEGIPP DQQRLIFAGK 201 QLEDGRTLSD YNIQKESTLH LVLRLRGGC ``` | | |
| Evidence | Indistinguishable | | |
| Peptide list | 24\*, 238\*, 439\*, 487\*, 565\* | | |
| Peptides | 24\* | Confidence | Green |
| Runs | 1, 2, 3 |
| Relation | Discriminating |
| Proteins | RL40\_HUMAN, UBC\_HUMAN, UBB\_HUMAN, RS27A\_HUMAN |
| Sequence | ``` EGIPPDQQR ``` |
| Position | 34, 110, 186 |
| PTMs | none |
| 238\* | Confidence | Green |
| Runs | 1, 3, 4 |
| Relation | Discriminating |
| Proteins | RL40\_HUMAN, UBC\_HUMAN, UBB\_HUMAN, RS27A\_HUMAN |
| Sequence | ``` TLSDYNIQK ``` |
| Position | 55, 131, 207 |
| PTMs | none |
| 439\* | Confidence | Green |
| Runs | 1, 2, 3 |
| Relation | Discriminating |
| Proteins | RL40\_HUMAN, UBC\_HUMAN, UBB\_HUMAN, RS27A\_HUMAN |
| Sequence | ``` MQIFVK ``` |
| Position | 1, 77, 153 |
| PTMs | none |
| 487\* | Confidence | Green |
| Runs | 1, 2, 3, 4 |
| Relation | Discriminating |
| Proteins | RL40\_HUMAN, UBC\_HUMAN, UBB\_HUMAN, RS27A\_HUMAN |
| Sequence | ``` ESTLHLVLR ``` |
| Position | 64, 140, 216 |
| PTMs | none |
| 565\* | Confidence | Green |
| Runs | 1, 2, 3, 4 |
| Relation | Discriminating |
| Proteins | RL40\_HUMAN, UBC\_HUMAN, UBB\_HUMAN, RS27A\_HUMAN |
| Sequence | ``` TITLEVEPSDTIENVK ``` |
| Position | 12, 88, 164 |
| PTMs | Variant #1: Deamidation+N(14)  Variant #2: none |

  

Protein P62979

| Name | RS27A\_HUMAN | | |
| Description | Ubiquitin 40S ribosomal protein S27a OS Homo sapiens GN RPS27A PE 1 SV 2 | | |
| Sequence | ```   1 MQIFVKTLTG KTITLEVEPS DTIENVKAKI QDKEGIPPDQ QRLIFAGKQL EDGRTLSDYN IQKESTLHLV LRLRGGAKKR KKKSYTTPKK NKHKRKKVKL 101 AVLKYYKVDE NGKISRLRRE CPSDECGAGV FMASHFDRHY CGKCCLTYCF NKPEDK ``` | | |
| Evidence | Indistinguishable | | |
| Peptide list | 24\*, 238\*, 439\*, 487\*, 565\* | | |
| Peptides | 24\* | Confidence | Green |
| Runs | 1, 2, 3 |
| Relation | Discriminating |
| Proteins | RL40\_HUMAN, UBC\_HUMAN, UBB\_HUMAN, RS27A\_HUMAN |
| Sequence | ``` EGIPPDQQR ``` |
| Position | 34 |
| PTMs | none |
| 238\* | Confidence | Green |
| Runs | 1, 3, 4 |
| Relation | Discriminating |
| Proteins | RL40\_HUMAN, UBC\_HUMAN, UBB\_HUMAN, RS27A\_HUMAN |
| Sequence | ``` TLSDYNIQK ``` |
| Position | 55 |
| PTMs | none |
| 439\* | Confidence | Green |
| Runs | 1, 2, 3 |
| Relation | Discriminating |
| Proteins | RL40\_HUMAN, UBC\_HUMAN, UBB\_HUMAN, RS27A\_HUMAN |
| Sequence | ``` MQIFVK ``` |
| Position | 1 |
| PTMs | none |
| 487\* | Confidence | Green |
| Runs | 1, 2, 3, 4 |
| Relation | Discriminating |
| Proteins | RL40\_HUMAN, UBC\_HUMAN, UBB\_HUMAN, RS27A\_HUMAN |
| Sequence | ``` ESTLHLVLR ``` |
| Position | 64 |
| PTMs | none |
| 565\* | Confidence | Green |
| Runs | 1, 2, 3, 4 |
| Relation | Discriminating |
| Proteins | RL40\_HUMAN, UBC\_HUMAN, UBB\_HUMAN, RS27A\_HUMAN |
| Sequence | ``` TITLEVEPSDTIENVK ``` |
| Position | 12 |
| PTMs | Variant #1: Deamidation+N(14)  Variant #2: none |

  

Protein P62736

| Name | ACTA\_HUMAN | | |
| Description | Actin aortic smooth muscle OS Homo sapiens GN ACTA2 PE 1 SV 1 | | |
| Sequence | ```   1 MCEEEDSTAL VCDNGSGLCK AGFAGDDAPR AVFPSIVGRP RHQGVMVGMG QKDSYVGDEA QSKRGILTLK YPIEHGIITN WDDMEKIWHH SFYNELRVAP 101 EEHPTLLTEA PLNPKANREK MTQIMFETFN VPAMYVAIQA VLSLYASGRT TGIVLDSGDG VTHNVPIYEG YALPHAIMRL DLAGRDLTDY LMKILTERGY 201 SFVTTAEREI VRDIKEKLCY VALDFENEMA TAASSSSLEK SYELPDGQVI TIGNERFRCP ETLFQPSFIG MESAGIHETT YNSIMKCDID IRKDLYANNV 301 LSGGTTMYPG IADRMQKEIT ALAPSTMKIK IIAPPERKYS VWIGGSILAS LSTFQQMWIS KQEYDEAGPS IVHRKCF ``` | | |
| Evidence | Indistinguishable | | |
| Peptide list | 51\*\*, 121\*\*, 134\*\*, 140\*\*, 141\*\*, 431\*\*, 700\*, 766\*\* | | |
| Peptides | 51\*\* | Confidence | Green |
| Runs | 1, 2, 3, 4 |
| Relation | NonDiscriminating |
| Proteins | ACTG\_HUMAN, ACTB\_HUMAN, ACTC\_HUMAN, ACTS\_HUMAN, ACTH\_HUMAN, ACTA\_HUMAN |
| Sequence | ``` DSYVGDEAQSK ``` |
| Position | 53 |
| PTMs | none |
| 121\*\* | Confidence | Green |
| Runs | 1, 2, 3, 4 |
| Relation | NonDiscriminating |
| Proteins | ACTG\_HUMAN, ACTB\_HUMAN, POTEE\_HUMAN, POTEF\_HUMAN, POTEJ\_HUMAN, POTEI\_HUMAN, ACTC\_HUMAN, ACTS\_HUMAN, ACTH\_HUMAN, ACTA\_HUMAN |
| Sequence | ``` AGFAGDDAPR ``` |
| Position | 21 |
| PTMs | none |
| 134\*\* | Confidence | Green |
| Runs | 1, 2, 4 |
| Relation | NonDiscriminating |
| Proteins | ACTG\_HUMAN, ACTB\_HUMAN, ACTC\_HUMAN, ACTS\_HUMAN, ACTH\_HUMAN, ACTA\_HUMAN, ACTBL\_HUMAN |
| Sequence | ``` HQGVMVGMGQK ``` |
| Position | 42 |
| PTMs | none |
| 140\*\* | Confidence | Green |
| Runs | 1, 2, 4 |
| Relation | NonDiscriminating |
| Proteins | ACTG\_HUMAN, ACTB\_HUMAN, POTEE\_HUMAN, POTEF\_HUMAN, POTEJ\_HUMAN, POTEI\_HUMAN, ACTC\_HUMAN, ACTS\_HUMAN, ACTBM\_HUMAN, ACTH\_HUMAN, ACTA\_HUMAN, ACTBL\_HUMAN |
| Sequence | ``` LDLAGR ``` |
| Position | 180 |
| PTMs | none |
| 141\*\* | Confidence | Green |
| Runs | 1, 2, 3, 4 |
| Relation | NonDiscriminating |
| Proteins | ACTG\_HUMAN, ACTB\_HUMAN, ACTC\_HUMAN, ACTS\_HUMAN, ACTH\_HUMAN, ACTA\_HUMAN, ACTBL\_HUMAN |
| Sequence | ``` IIAPPER ``` |
| Position | 331 |
| PTMs | none |
| 431\*\* | Confidence | Green |
| Runs | 1, 2, 3, 4 |
| Relation | NonDiscriminating |
| Proteins | ACTG\_HUMAN, ACTB\_HUMAN, ACTC\_HUMAN, ACTS\_HUMAN, ACTH\_HUMAN, ACTA\_HUMAN |
| Sequence | ``` EITALAPSTMK ``` |
| Position | 318 |
| PTMs | none |
| 700\* | Confidence | Green |
| Runs | 1, 2, 3 |
| Relation | Discriminating |
| Proteins | ACTC\_HUMAN, ACTS\_HUMAN, ACTH\_HUMAN, ACTA\_HUMAN |
| Sequence | ``` YPIEHGIITNWDDMEK ``` |
| Position | 71 |
| PTMs | none |
| 766\*\* | Confidence | Green |
| Runs | 1, 2, 3, 4 |
| Relation | NonDiscriminating |
| Proteins | ACTG\_HUMAN, ACTB\_HUMAN, POTEE\_HUMAN, POTEF\_HUMAN, ACTC\_HUMAN, ACTS\_HUMAN, ACTBM\_HUMAN, ACTH\_HUMAN, ACTA\_HUMAN, ACTBL\_HUMAN |
| Sequence | ``` SYELPDGQVITIGNER ``` |
| Position | 241 |
| PTMs | none |

  

Protein P68032

| Name | ACTC\_HUMAN | | |
| Description | Actin alpha cardiac muscle 1 OS Homo sapiens GN ACTC1 PE 1 SV 1 | | |
| Sequence | ```   1 MCDDEETTAL VCDNGSGLVK AGFAGDDAPR AVFPSIVGRP RHQGVMVGMG QKDSYVGDEA QSKRGILTLK YPIEHGIITN WDDMEKIWHH TFYNELRVAP 101 EEHPTLLTEA PLNPKANREK MTQIMFETFN VPAMYVAIQA VLSLYASGRT TGIVLDSGDG VTHNVPIYEG YALPHAIMRL DLAGRDLTDY LMKILTERGY 201 SFVTTAEREI VRDIKEKLCY VALDFENEMA TAASSSSLEK SYELPDGQVI TIGNERFRCP ETLFQPSFIG MESAGIHETT YNSIMKCDID IRKDLYANNV 301 LSGGTTMYPG IADRMQKEIT ALAPSTMKIK IIAPPERKYS VWIGGSILAS LSTFQQMWIS KQEYDEAGPS IVHRKCF ``` | | |
| Evidence | Indistinguishable | | |
| Peptide list | 51\*\*, 121\*\*, 134\*\*, 140\*\*, 141\*\*, 431\*\*, 700\*, 766\*\* | | |
| Peptides | 51\*\* | Confidence | Green |
| Runs | 1, 2, 3, 4 |
| Relation | NonDiscriminating |
| Proteins | ACTG\_HUMAN, ACTB\_HUMAN, ACTC\_HUMAN, ACTS\_HUMAN, ACTH\_HUMAN, ACTA\_HUMAN |
| Sequence | ``` DSYVGDEAQSK ``` |
| Position | 53 |
| PTMs | none |
| 121\*\* | Confidence | Green |
| Runs | 1, 2, 3, 4 |
| Relation | NonDiscriminating |
| Proteins | ACTG\_HUMAN, ACTB\_HUMAN, POTEE\_HUMAN, POTEF\_HUMAN, POTEJ\_HUMAN, POTEI\_HUMAN, ACTC\_HUMAN, ACTS\_HUMAN, ACTH\_HUMAN, ACTA\_HUMAN |
| Sequence | ``` AGFAGDDAPR ``` |
| Position | 21 |
| PTMs | none |
| 134\*\* | Confidence | Green |
| Runs | 1, 2, 4 |
| Relation | NonDiscriminating |
| Proteins | ACTG\_HUMAN, ACTB\_HUMAN, ACTC\_HUMAN, ACTS\_HUMAN, ACTH\_HUMAN, ACTA\_HUMAN, ACTBL\_HUMAN |
| Sequence | ``` HQGVMVGMGQK ``` |
| Position | 42 |
| PTMs | none |
| 140\*\* | Confidence | Green |
| Runs | 1, 2, 4 |
| Relation | NonDiscriminating |
| Proteins | ACTG\_HUMAN, ACTB\_HUMAN, POTEE\_HUMAN, POTEF\_HUMAN, POTEJ\_HUMAN, POTEI\_HUMAN, ACTC\_HUMAN, ACTS\_HUMAN, ACTBM\_HUMAN, ACTH\_HUMAN, ACTA\_HUMAN, ACTBL\_HUMAN |
| Sequence | ``` LDLAGR ``` |
| Position | 180 |
| PTMs | none |
| 141\*\* | Confidence | Green |
| Runs | 1, 2, 3, 4 |
| Relation | NonDiscriminating |
| Proteins | ACTG\_HUMAN, ACTB\_HUMAN, ACTC\_HUMAN, ACTS\_HUMAN, ACTH\_HUMAN, ACTA\_HUMAN, ACTBL\_HUMAN |
| Sequence | ``` IIAPPER ``` |
| Position | 331 |
| PTMs | none |
| 431\*\* | Confidence | Green |
| Runs | 1, 2, 3, 4 |
| Relation | NonDiscriminating |
| Proteins | ACTG\_HUMAN, ACTB\_HUMAN, ACTC\_HUMAN, ACTS\_HUMAN, ACTH\_HUMAN, ACTA\_HUMAN |
| Sequence | ``` EITALAPSTMK ``` |
| Position | 318 |
| PTMs | none |
| 700\* | Confidence | Green |
| Runs | 1, 2, 3 |
| Relation | Discriminating |
| Proteins | ACTC\_HUMAN, ACTS\_HUMAN, ACTH\_HUMAN, ACTA\_HUMAN |
| Sequence | ``` YPIEHGIITNWDDMEK ``` |
| Position | 71 |
| PTMs | none |
| 766\*\* | Confidence | Green |
| Runs | 1, 2, 3, 4 |
| Relation | NonDiscriminating |
| Proteins | ACTG\_HUMAN, ACTB\_HUMAN, POTEE\_HUMAN, POTEF\_HUMAN, ACTC\_HUMAN, ACTS\_HUMAN, ACTBM\_HUMAN, ACTH\_HUMAN, ACTA\_HUMAN, ACTBL\_HUMAN |
| Sequence | ``` SYELPDGQVITIGNER ``` |
| Position | 241 |
| PTMs | none |

  

Protein P68133

| Name | ACTS\_HUMAN | | |
| Description | Actin alpha skeletal muscle OS Homo sapiens GN ACTA1 PE 1 SV 1 | | |
| Sequence | ```   1 MCDEDETTAL VCDNGSGLVK AGFAGDDAPR AVFPSIVGRP RHQGVMVGMG QKDSYVGDEA QSKRGILTLK YPIEHGIITN WDDMEKIWHH TFYNELRVAP 101 EEHPTLLTEA PLNPKANREK MTQIMFETFN VPAMYVAIQA VLSLYASGRT TGIVLDSGDG VTHNVPIYEG YALPHAIMRL DLAGRDLTDY LMKILTERGY 201 SFVTTAEREI VRDIKEKLCY VALDFENEMA TAASSSSLEK SYELPDGQVI TIGNERFRCP ETLFQPSFIG MESAGIHETT YNSIMKCDID IRKDLYANNV 301 MSGGTTMYPG IADRMQKEIT ALAPSTMKIK IIAPPERKYS VWIGGSILAS LSTFQQMWIT KQEYDEAGPS IVHRKCF ``` | | |
| Evidence | Indistinguishable | | |
| Peptide list | 51\*\*, 121\*\*, 134\*\*, 140\*\*, 141\*\*, 431\*\*, 700\*, 766\*\* | | |
| Peptides | 51\*\* | Confidence | Green |
| Runs | 1, 2, 3, 4 |
| Relation | NonDiscriminating |
| Proteins | ACTG\_HUMAN, ACTB\_HUMAN, ACTC\_HUMAN, ACTS\_HUMAN, ACTH\_HUMAN, ACTA\_HUMAN |
| Sequence | ``` DSYVGDEAQSK ``` |
| Position | 53 |
| PTMs | none |
| 121\*\* | Confidence | Green |
| Runs | 1, 2, 3, 4 |
| Relation | NonDiscriminating |
| Proteins | ACTG\_HUMAN, ACTB\_HUMAN, POTEE\_HUMAN, POTEF\_HUMAN, POTEJ\_HUMAN, POTEI\_HUMAN, ACTC\_HUMAN, ACTS\_HUMAN, ACTH\_HUMAN, ACTA\_HUMAN |
| Sequence | ``` AGFAGDDAPR ``` |
| Position | 21 |
| PTMs | none |
| 134\*\* | Confidence | Green |
| Runs | 1, 2, 4 |
| Relation | NonDiscriminating |
| Proteins | ACTG\_HUMAN, ACTB\_HUMAN, ACTC\_HUMAN, ACTS\_HUMAN, ACTH\_HUMAN, ACTA\_HUMAN, ACTBL\_HUMAN |
| Sequence | ``` HQGVMVGMGQK ``` |
| Position | 42 |
| PTMs | none |
| 140\*\* | Confidence | Green |
| Runs | 1, 2, 4 |
| Relation | NonDiscriminating |
| Proteins | ACTG\_HUMAN, ACTB\_HUMAN, POTEE\_HUMAN, POTEF\_HUMAN, POTEJ\_HUMAN, POTEI\_HUMAN, ACTC\_HUMAN, ACTS\_HUMAN, ACTBM\_HUMAN, ACTH\_HUMAN, ACTA\_HUMAN, ACTBL\_HUMAN |
| Sequence | ``` LDLAGR ``` |
| Position | 180 |
| PTMs | none |
| 141\*\* | Confidence | Green |
| Runs | 1, 2, 3, 4 |
| Relation | NonDiscriminating |
| Proteins | ACTG\_HUMAN, ACTB\_HUMAN, ACTC\_HUMAN, ACTS\_HUMAN, ACTH\_HUMAN, ACTA\_HUMAN, ACTBL\_HUMAN |
| Sequence | ``` IIAPPER ``` |
| Position | 331 |
| PTMs | none |
| 431\*\* | Confidence | Green |
| Runs | 1, 2, 3, 4 |
| Relation | NonDiscriminating |
| Proteins | ACTG\_HUMAN, ACTB\_HUMAN, ACTC\_HUMAN, ACTS\_HUMAN, ACTH\_HUMAN, ACTA\_HUMAN |
| Sequence | ``` EITALAPSTMK ``` |
| Position | 318 |
| PTMs | none |
| 700\* | Confidence | Green |
| Runs | 1, 2, 3 |
| Relation | Discriminating |
| Proteins | ACTC\_HUMAN, ACTS\_HUMAN, ACTH\_HUMAN, ACTA\_HUMAN |
| Sequence | ``` YPIEHGIITNWDDMEK ``` |
| Position | 71 |
| PTMs | none |
| 766\*\* | Confidence | Green |
| Runs | 1, 2, 3, 4 |
| Relation | NonDiscriminating |
| Proteins | ACTG\_HUMAN, ACTB\_HUMAN, POTEE\_HUMAN, POTEF\_HUMAN, ACTC\_HUMAN, ACTS\_HUMAN, ACTBM\_HUMAN, ACTH\_HUMAN, ACTA\_HUMAN, ACTBL\_HUMAN |
| Sequence | ``` SYELPDGQVITIGNER ``` |
| Position | 241 |
| PTMs | none |

  

Protein P63267

| Name | ACTH\_HUMAN | | |
| Description | Actin gamma enteric smooth muscle OS Homo sapiens GN ACTG2 PE 1 SV 1 | | |
| Sequence | ```   1 MCEEETTALV CDNGSGLCKA GFAGDDAPRA VFPSIVGRPR HQGVMVGMGQ KDSYVGDEAQ SKRGILTLKY PIEHGIITNW DDMEKIWHHS FYNELRVAPE 101 EHPTLLTEAP LNPKANREKM TQIMFETFNV PAMYVAIQAV LSLYASGRTT GIVLDSGDGV THNVPIYEGY ALPHAIMRLD LAGRDLTDYL MKILTERGYS 201 FVTTAEREIV RDIKEKLCYV ALDFENEMAT AASSSSLEKS YELPDGQVIT IGNERFRCPE TLFQPSFIGM ESAGIHETTY NSIMKCDIDI RKDLYANNVL 301 SGGTTMYPGI ADRMQKEITA LAPSTMKIKI IAPPERKYSV WIGGSILASL STFQQMWISK PEYDEAGPSI VHRKCF ``` | | |
| Evidence | Indistinguishable | | |
| Peptide list | 51\*\*, 121\*\*, 134\*\*, 140\*\*, 141\*\*, 431\*\*, 700\*, 766\*\* | | |
| Peptides | 51\*\* | Confidence | Green |
| Runs | 1, 2, 3, 4 |
| Relation | NonDiscriminating |
| Proteins | ACTG\_HUMAN, ACTB\_HUMAN, ACTC\_HUMAN, ACTS\_HUMAN, ACTH\_HUMAN, ACTA\_HUMAN |
| Sequence | ``` DSYVGDEAQSK ``` |
| Position | 52 |
| PTMs | none |
| 121\*\* | Confidence | Green |
| Runs | 1, 2, 3, 4 |
| Relation | NonDiscriminating |
| Proteins | ACTG\_HUMAN, ACTB\_HUMAN, POTEE\_HUMAN, POTEF\_HUMAN, POTEJ\_HUMAN, POTEI\_HUMAN, ACTC\_HUMAN, ACTS\_HUMAN, ACTH\_HUMAN, ACTA\_HUMAN |
| Sequence | ``` AGFAGDDAPR ``` |
| Position | 20 |
| PTMs | none |
| 134\*\* | Confidence | Green |
| Runs | 1, 2, 4 |
| Relation | NonDiscriminating |
| Proteins | ACTG\_HUMAN, ACTB\_HUMAN, ACTC\_HUMAN, ACTS\_HUMAN, ACTH\_HUMAN, ACTA\_HUMAN, ACTBL\_HUMAN |
| Sequence | ``` HQGVMVGMGQK ``` |
| Position | 41 |
| PTMs | none |
| 140\*\* | Confidence | Green |
| Runs | 1, 2, 4 |
| Relation | NonDiscriminating |
| Proteins | ACTG\_HUMAN, ACTB\_HUMAN, POTEE\_HUMAN, POTEF\_HUMAN, POTEJ\_HUMAN, POTEI\_HUMAN, ACTC\_HUMAN, ACTS\_HUMAN, ACTBM\_HUMAN, ACTH\_HUMAN, ACTA\_HUMAN, ACTBL\_HUMAN |
| Sequence | ``` LDLAGR ``` |
| Position | 179 |
| PTMs | none |
| 141\*\* | Confidence | Green |
| Runs | 1, 2, 3, 4 |
| Relation | NonDiscriminating |
| Proteins | ACTG\_HUMAN, ACTB\_HUMAN, ACTC\_HUMAN, ACTS\_HUMAN, ACTH\_HUMAN, ACTA\_HUMAN, ACTBL\_HUMAN |
| Sequence | ``` IIAPPER ``` |
| Position | 330 |
| PTMs | none |
| 431\*\* | Confidence | Green |
| Runs | 1, 2, 3, 4 |
| Relation | NonDiscriminating |
| Proteins | ACTG\_HUMAN, ACTB\_HUMAN, ACTC\_HUMAN, ACTS\_HUMAN, ACTH\_HUMAN, ACTA\_HUMAN |
| Sequence | ``` EITALAPSTMK ``` |
| Position | 317 |
| PTMs | none |
| 700\* | Confidence | Green |
| Runs | 1, 2, 3 |
| Relation | Discriminating |
| Proteins | ACTC\_HUMAN, ACTS\_HUMAN, ACTH\_HUMAN, ACTA\_HUMAN |
| Sequence | ``` YPIEHGIITNWDDMEK ``` |
| Position | 70 |
| PTMs | none |
| 766\*\* | Confidence | Green |
| Runs | 1, 2, 3, 4 |
| Relation | NonDiscriminating |
| Proteins | ACTG\_HUMAN, ACTB\_HUMAN, POTEE\_HUMAN, POTEF\_HUMAN, ACTC\_HUMAN, ACTS\_HUMAN, ACTBM\_HUMAN, ACTH\_HUMAN, ACTA\_HUMAN, ACTBL\_HUMAN |
| Sequence | ``` SYELPDGQVITIGNER ``` |
| Position | 240 |
| PTMs | none |

  

Protein A5A3E0

| Name | POTEF\_HUMAN | | |
| Description | POTE ankyrin domain family member F OS Homo sapiens GN POTEF PE 1 SV 2 | | |
| Sequence | ```    1 MVVEVDSMPA ASSVKKPFGL RSKMGKWCCR CFPCCRESGK SNVGTSGDHD DSAMKTLRSK MGKWCRHCFP CCRGSGKSNV GASGDHDDSA MKTLRNKMGK  101 WCCHCFPCCR GSSKSKVGAW GDYDDSAFME PRYHVRGEDL DKLHRAAWWG KVPRKDLIVM LRDTDVNKQD KQKRTALHLA SANGNSEVVK LLLDRRCQLN  201 VLDNKKRTAL IKAVQCQEDE CALMLLEHGT DPNIPDEYGN TTLHYAIYNE DKLMAKALLL YGADIESKNK HGLTPLLLGV HEQKQQVVKF LIKKKANLNA  301 LDRYGRTALI LAVCCGSASI VSLLLEQNID VSSQDLSGQT AREYAVSSHH HVICQLLSDY KEKQMLKISS ENSNPEQDLK LTSEEESQRF KGSENSQPEK  401 MSQEPEINKD GDREVEEEMK KHESNNVGLL ENLTNGVTAG NGDNGLIPQR KSRTPENQQF PDNESEEYHR ICELLSDYKE KQMPKYSSEN SNPEQDLKLT  501 SEEESQRLKG SENGQPEKRS QEPEINKDGD RELENFMAIE EMKKHRSTHV GFPENLTNGA TAGNGDDGLI PPRKSRTPES QQFPDTENEE YHSDEQNDTQ  601 KQFCEEQNTG ILHDEILIHE EKQIEVVEKM NSELSLSCKK EKDILHENST LREEIAMLRL ELDTMKHQSQ LREKKYLEDI ESVKKRNDNL LKALQLNELT  701 MDDDTAVLVI DNGSGMCKAG FAGDDAPRAV FPSIVGRPRQ QGMMGGMHQK ESYVGKEAQS KRGILTLKYP MEHGIITNWD DMEKIWHHTF YNELRVAPEE  801 HPVLLTEATL NPKANREKMT QIMFETFNTP AMYVAIQAVL SLYTSGRTTG IVMDSGDGVT HTVPIYEGNA LPHATLRLDL AGRELPDYLM KILTEHGYRF  901 TTMAEREIVR DIKEKLCYVA LDFEQEMATV ASSSSLEKSY ELPDGQVITI GNERFRCPEA LFQPCFLGME SCGIHETTFN SIMKSDVDIR KDLYTNTVLS 1001 GGTTMYPGMA HRMQKEIAAL APSMMKIRII APPKRKYSVW VGGSILASLS TFQQMWISKQ EYDESGPSIV HRKCL ``` | | |
| Evidence | Indistinguishable | | |
| Peptide list | 121\*\*, 140\*\*, 216\*\*, 539\*, 766\*\* | | |
| Peptides | 121\*\* | Confidence | Green |
| Runs | 1, 2, 3, 4 |
| Relation | NonDiscriminating |
| Proteins | ACTG\_HUMAN, ACTB\_HUMAN, POTEE\_HUMAN, POTEF\_HUMAN, POTEJ\_HUMAN, POTEI\_HUMAN, ACTC\_HUMAN, ACTS\_HUMAN, ACTH\_HUMAN, ACTA\_HUMAN |
| Sequence | ``` AGFAGDDAPR ``` |
| Position | 719 |
| PTMs | none |
| 140\*\* | Confidence | Green |
| Runs | 1, 2, 4 |
| Relation | NonDiscriminating |
| Proteins | ACTG\_HUMAN, ACTB\_HUMAN, POTEE\_HUMAN, POTEF\_HUMAN, POTEJ\_HUMAN, POTEI\_HUMAN, ACTC\_HUMAN, ACTS\_HUMAN, ACTBM\_HUMAN, ACTH\_HUMAN, ACTA\_HUMAN, ACTBL\_HUMAN |
| Sequence | ``` LDLAGR ``` |
| Position | 878 |
| PTMs | none |
| 216\*\* | Confidence | Green |
| Runs | 1, 2, 3 |
| Relation | NonDiscriminating |
| Proteins | ACTG\_HUMAN, ACTB\_HUMAN, POTEE\_HUMAN, POTEF\_HUMAN, POTEJ\_HUMAN, POTEI\_HUMAN, ACTBM\_HUMAN |
| Sequence | ``` QEYDESGPSIVHR ``` |
| Position | 1060 |
| PTMs | none |
| 539\* | Confidence | Yellow |
| Runs | 1, 3, 4 |
| Relation | Discriminating |
| Proteins | POTEE\_HUMAN, POTEF\_HUMAN, POTEJ\_HUMAN, POTEI\_HUMAN |
| Sequence | ``` DLIVMLRDTDVNK ``` |
| Position | 156 |
| PTMs | none |
| 766\*\* | Confidence | Green |
| Runs | 1, 2, 3, 4 |
| Relation | NonDiscriminating |
| Proteins | ACTG\_HUMAN, ACTB\_HUMAN, POTEE\_HUMAN, POTEF\_HUMAN, ACTC\_HUMAN, ACTS\_HUMAN, ACTBM\_HUMAN, ACTH\_HUMAN, ACTA\_HUMAN, ACTBL\_HUMAN |
| Sequence | ``` SYELPDGQVITIGNER ``` |
| Position | 939 |
| PTMs | none |

  

Protein Q6S8J3

| Name | POTEE\_HUMAN | | |
| Description | POTE ankyrin domain family member E OS Homo sapiens GN POTEE PE 1 SV 3 | | |
| Sequence | ```    1 MVVEVDSMPA ASSVKKPFGL RSKMGKWCCR CFPCYRESGK SNVGTSGDHD DSAMKTLRSK MGKWCHHCFP CCRGSGKSNV GASGDHDDSA MKTLRNKMGK  101 WCCHCFPCCR GSGKSKVGAW GDYDDSAFME PRYHVRGEDL DKLHRAAWWG KVPRKDLIVM LRDTDVNKKD KQKRTALHLA SANGNSEVVK LLLDRRCQLN  201 VLDNKKRTAL IKAVQCQEDE CALMLLEHGT DPNIPDEYGN TTLHYAIYNE DKLMAKALLL YGADIESKNK HGLTPLLLGV HEQKQQVVKF LIKKKANLNA  301 LDRYGRTALI LAVCCGSASI VSLLLEQNID VSSQDLSGQT AREYAVSSHH HVICQLLSDY KEKQMLKISS ENSNPEQELK LTSEEESQRF KGSENSQPEK  401 MSQELEINKD GDREVEEEMK KHESNNVGLL ENLTNGVTAG NGDNGLIPQR KSRTPENQQF PDNESEEYHR ICELLSDYKE KQMPKYSSEN SNPEQDLKLT  501 SEEESQRLKG SENGQPEKRS QEPEINKDGD RELENFMAIE EMKKHGSTHV GFPENLTNGA TAGNGDDGLI PPRKSRTPES QQFPDTENEE YHSDEQNDTQ  601 KQFCEEQNTG ILHDEILIHE EKQIEVVEKM NSELSLSCKK EKDVLHENST LREEIAMLRL ELDTMKHQSQ LREKKYLEDI ESVKKKNDNL LKALQLNELT  701 MDDDTAVLVI DNGSGMCKAG FAGDDAPRAV FPSIVGRPRQ QGMMGGMHQK ESYVGKEAQS KRGILTLKYP MEHGIITNWD DMEKIWHHTF YNELRVAPEE  801 HPILLTEAPL NPKANREKMT QIMFETFNTP AMYVAIQAVP SLYTSGRTTG IVMDSGDGVT HTVPIYEGNA LPHATLRLDL AGRELPDYLM KILTERGYRF  901 TTMAEREIVR DIKEKLCYVA LDFEQEMATA ASSSSLEKSY ELPDGQVITI GNERFRCPEA LFQPCFLGME SCGIHETTFN SIMKSDVDIR KDLYTNTVLS 1001 GGTTMYPGMA HRMQKEIAAL APSMMKIRII APPKRKYSVW VGGSILASLS TFQQMWISKQ EYDESGPSIV HRKCF ``` | | |
| Evidence | Indistinguishable | | |
| Peptide list | 121\*\*, 140\*\*, 216\*\*, 539\*, 766\*\*, 1050\*\* | | |
| Peptides | 121\*\* | Confidence | Green |
| Runs | 1, 2, 3, 4 |
| Relation | NonDiscriminating |
| Proteins | ACTG\_HUMAN, ACTB\_HUMAN, POTEE\_HUMAN, POTEF\_HUMAN, POTEJ\_HUMAN, POTEI\_HUMAN, ACTC\_HUMAN, ACTS\_HUMAN, ACTH\_HUMAN, ACTA\_HUMAN |
| Sequence | ``` AGFAGDDAPR ``` |
| Position | 719 |
| PTMs | none |
| 140\*\* | Confidence | Green |
| Runs | 1, 2, 4 |
| Relation | NonDiscriminating |
| Proteins | ACTG\_HUMAN, ACTB\_HUMAN, POTEE\_HUMAN, POTEF\_HUMAN, POTEJ\_HUMAN, POTEI\_HUMAN, ACTC\_HUMAN, ACTS\_HUMAN, ACTBM\_HUMAN, ACTH\_HUMAN, ACTA\_HUMAN, ACTBL\_HUMAN |
| Sequence | ``` LDLAGR ``` |
| Position | 878 |
| PTMs | none |
| 216\*\* | Confidence | Green |
| Runs | 1, 2, 3 |
| Relation | NonDiscriminating |
| Proteins | ACTG\_HUMAN, ACTB\_HUMAN, POTEE\_HUMAN, POTEF\_HUMAN, POTEJ\_HUMAN, POTEI\_HUMAN, ACTBM\_HUMAN |
| Sequence | ``` QEYDESGPSIVHR ``` |
| Position | 1060 |
| PTMs | none |
| 539\* | Confidence | Yellow |
| Runs | 1, 3, 4 |
| Relation | Discriminating |
| Proteins | POTEE\_HUMAN, POTEF\_HUMAN, POTEJ\_HUMAN, POTEI\_HUMAN |
| Sequence | ``` DLIVMLRDTDVNK ``` |
| Position | 156 |
| PTMs | none |
| 766\*\* | Confidence | Green |
| Runs | 1, 2, 3, 4 |
| Relation | NonDiscriminating |
| Proteins | ACTG\_HUMAN, ACTB\_HUMAN, POTEE\_HUMAN, POTEF\_HUMAN, ACTC\_HUMAN, ACTS\_HUMAN, ACTBM\_HUMAN, ACTH\_HUMAN, ACTA\_HUMAN, ACTBL\_HUMAN |
| Sequence | ``` SYELPDGQVITIGNER ``` |
| Position | 939 |
| PTMs | none |
| 1050\*\* | Confidence | Green |
| Runs | 1, 2, 4 |
| Relation | NonDiscriminating |
| Proteins | ACTG\_HUMAN, ACTB\_HUMAN, POTEE\_HUMAN |
| Sequence | ``` LCYVALDFEQEMATAASSSSLEK ``` |
| Position | 916 |
| PTMs | Carbamidomethyl+C(2) |

  

Protein P0CG39

| Name | POTEJ\_HUMAN | | |
| Description | POTE ankyrin domain family member J OS Homo sapiens GN POTEJ PE 3 SV 1 | | |
| Sequence | ```    1 MVAEVDSMPA ASSVKKPFVL RSKMGKWCRH CFPCCRGSGK SNVGTSGDQD DSTMKTLRSK MGKWCCHCFP CCRGSGKSNV GAWGDYDDSA FVEPRYHVRR  101 EDLDKLHRAA WWGKVARKDL IVMLRDTDVN KQDKQKRTAL HLASANGNSG VVKLLLDRRC QLNVLDNKKR TALTKAVQCQ EDECALMLLE HGTDPNIPDE  201 YGNTTLHYAI YNEDKLMAKA LLLYGADIES KNKHGLTPLL LGVHEQKQQV VKFLIKKKAN LNALDRYGRT ALILAVCCGS ASIVSLLLEQ NIDVSSQDLS  301 GQTAREYAVS SHHHVICQLL SDYKEKQMLK ISSENSNPEQ DLKLTSEEES QRFKGSENSQ PEKMSQEPEI NKDGDREVEE EMKKHESNNV GLLENLSNGV  401 TAGNGDDGLI PQRKSRTPEN QQFPDNESEE YHRICELVSD YKEKQMPKYS SENSNPEQDL KLTSEEESQR LKGSENGQPE KRSQEPEINK DGDRELENFM  501 AIEEMKKHGS THVGFPENLT NGATAGNGDD GLIPPRKSRT PESQQFPDTE NEEYHSDEQN DTQKQFCEEQ NTGILHDEIL IHEEKQIEVV EKMNSELSLS  601 CKKERDFLHE NSMLREEIAM LRLELDTMKH QSQLRKKKYL EDIESVKKKN DNLLKALQLN ELTMDDDTAV LVIDNGSGMC KAGFAGDDAP RAVFPSIVGC  701 PRQQGMMGGM HQKESYVGKE AQSKRGILTL KYPMEHGIIT NWDDMEKIWH HTFYNELRVA PEEHPILLTE APLNPKANRE KMTQIMFETF NTPAMYVAIQ  801 AMLSLYTSGR TTGIVMDSGD GVTHTVPIYD GNALPHATLR LDLAGRELTD YLMKILTERG YRFTTMAERE IVRDIKEKLC YVALDFEQEM AMVASSSSLE  901 KSYELPDGQV ITISNEWFRC PEALFQPCFL GMESCGIHET TFNSIMKSDV DIRKDLYTNT VLSGGTTMYP GMAHRMQKEI AALAPSMMKI RIIAPPKRKY 1001 SVWVGGSILA SLSTFQQMWI SKQEYDESGP SIVHRKCF ``` | | |
| Evidence | Indistinguishable | | |
| Peptide list | 121\*\*, 140\*\*, 216\*\*, 539\* | | |
| Peptides | 121\*\* | Confidence | Green |
| Runs | 1, 2, 3, 4 |
| Relation | NonDiscriminating |
| Proteins | ACTG\_HUMAN, ACTB\_HUMAN, POTEE\_HUMAN, POTEF\_HUMAN, POTEJ\_HUMAN, POTEI\_HUMAN, ACTC\_HUMAN, ACTS\_HUMAN, ACTH\_HUMAN, ACTA\_HUMAN |
| Sequence | ``` AGFAGDDAPR ``` |
| Position | 682 |
| PTMs | none |
| 140\*\* | Confidence | Green |
| Runs | 1, 2, 4 |
| Relation | NonDiscriminating |
| Proteins | ACTG\_HUMAN, ACTB\_HUMAN, POTEE\_HUMAN, POTEF\_HUMAN, POTEJ\_HUMAN, POTEI\_HUMAN, ACTC\_HUMAN, ACTS\_HUMAN, ACTBM\_HUMAN, ACTH\_HUMAN, ACTA\_HUMAN, ACTBL\_HUMAN |
| Sequence | ``` LDLAGR ``` |
| Position | 841 |
| PTMs | none |
| 216\*\* | Confidence | Green |
| Runs | 1, 2, 3 |
| Relation | NonDiscriminating |
| Proteins | ACTG\_HUMAN, ACTB\_HUMAN, POTEE\_HUMAN, POTEF\_HUMAN, POTEJ\_HUMAN, POTEI\_HUMAN, ACTBM\_HUMAN |
| Sequence | ``` QEYDESGPSIVHR ``` |
| Position | 1023 |
| PTMs | none |
| 539\* | Confidence | Yellow |
| Runs | 1, 3, 4 |
| Relation | Discriminating |
| Proteins | POTEE\_HUMAN, POTEF\_HUMAN, POTEJ\_HUMAN, POTEI\_HUMAN |
| Sequence | ``` DLIVMLRDTDVNK ``` |
| Position | 119 |
| PTMs | none |

  

Protein P0CG38

| Name | POTEI\_HUMAN | | |
| Description | POTE ankyrin domain family member I OS Homo sapiens GN POTEI PE 3 SV 1 | | |
| Sequence | ```    1 MVAEVDSMPA ASSVKKPFVL RSKMGKWCRH CFPCCRGSGK SNVGTSGDQD DSTMKTLRSK MGKWCCHCFP CCRGSGKSNV GTSGDHDDSA MKTLRSKMGK  101 WCCHCFPCCR GSGKSNVGAW GDYDDSAFVE PRYHVRREDL DKLHRAAWWG KVARKDLIVM LRDTDVNKQD KQKRTALHLA SANGNSGVVK LLLDRRCQLN  201 VLDNKKRTAL TKAVQCQEDE CALMLLEHGT DPNIPDEYGN TTLHYAIYNE DKLMAKALLL YGADIESKNK HGLTPLLLGV HEQKQQVVKF LIKKKANLNA  301 LDRYGRTALI LAVCCGSASI VSLLLEQNID VSSQDLSGQT AREYAVSSHH HVICQLLSDY KEKQMLKISS ENSNPEQDLK LTSEEESQRF KGSENSQPEK  401 MSQEPEINKD GDREVEEEMK KHESNNVGLL ENLSNGVTAG NGDDGLIPQR KSRTPENQQF PDNESEEYHR ICELVSDYKE KQMPKYSSEN SNPEQDLKLT  501 SEEESQRLKG SENGQPEKRS QEPEINKDGD RELENFMAIE EMKKHGSTHV GFPENLTNGA TAGNGDDGLI PPRKSRTPES QQFPDTENEE YHSDEQNDTQ  601 KQFCEEQNTG ILHDEILIHE EKQIEVVEKM NSELSLSCKK EKDFLHENST LREEIAMLRL ELDTMKHQSQ LRKKKYLEDI ESVKKKNDNL LKALQLNELT  701 MDDDTAVLVI DNGSGMCKAG FAGDDAPRAV FPSIVGRPRQ QGMMGGMHQK ESYVGKEAQS KRGILTLKYP MEHGIITNWD DMEKIWHHTF YNELRVAPEE  801 HPILLTEAPL NPKANREKMT QIMFETFNTP AMYVAIQAML SLYTSGRTTG IVMDSGDGVT HTVPIYDGNA LPHATLRLDL AGRELTDYLM KILTERGYRF  901 TTMAEREIVR DIKEKLCYVA LDFEQEMAMA ASSSSLEKSY ELPDGQVITI GNEWFRCPEA LFQPCFLGME SCGIHETTFN SIMKSDVDIR KDLYTNTVLS 1001 GGTTMYPGMA HRMQKEIAAL APSMLKIRII APPKRKYSVW VGGSILASLS TFQQMWISKQ EYDESGPSIV HRKCF ``` | | |
| Evidence | Indistinguishable | | |
| Peptide list | 121\*\*, 140\*\*, 216\*\*, 539\* | | |
| Peptides | 121\*\* | Confidence | Green |
| Runs | 1, 2, 3, 4 |
| Relation | NonDiscriminating |
| Proteins | ACTG\_HUMAN, ACTB\_HUMAN, POTEE\_HUMAN, POTEF\_HUMAN, POTEJ\_HUMAN, POTEI\_HUMAN, ACTC\_HUMAN, ACTS\_HUMAN, ACTH\_HUMAN, ACTA\_HUMAN |
| Sequence | ``` AGFAGDDAPR ``` |
| Position | 719 |
| PTMs | none |
| 140\*\* | Confidence | Green |
| Runs | 1, 2, 4 |
| Relation | NonDiscriminating |
| Proteins | ACTG\_HUMAN, ACTB\_HUMAN, POTEE\_HUMAN, POTEF\_HUMAN, POTEJ\_HUMAN, POTEI\_HUMAN, ACTC\_HUMAN, ACTS\_HUMAN, ACTBM\_HUMAN, ACTH\_HUMAN, ACTA\_HUMAN, ACTBL\_HUMAN |
| Sequence | ``` LDLAGR ``` |
| Position | 878 |
| PTMs | none |
| 216\*\* | Confidence | Green |
| Runs | 1, 2, 3 |
| Relation | NonDiscriminating |
| Proteins | ACTG\_HUMAN, ACTB\_HUMAN, POTEE\_HUMAN, POTEF\_HUMAN, POTEJ\_HUMAN, POTEI\_HUMAN, ACTBM\_HUMAN |
| Sequence | ``` QEYDESGPSIVHR ``` |
| Position | 1060 |
| PTMs | none |
| 539\* | Confidence | Yellow |
| Runs | 1, 3, 4 |
| Relation | Discriminating |
| Proteins | POTEE\_HUMAN, POTEF\_HUMAN, POTEJ\_HUMAN, POTEI\_HUMAN |
| Sequence | ``` DLIVMLRDTDVNK ``` |
| Position | 156 |
| PTMs | none |

  

Protein P57053

| Name | H2BFS\_HUMAN | | |
| Description | Histone H2B type F S OS Homo sapiens GN H2BFS PE 1 SV 2 | | |
| Sequence | ```   1 MPEPAKSAPA PKKGSKKAVT KAQKKDGRKR KRSRKESYSV YVYKVLKQVH PDTGISSKAM GIMNSFVNDI FERIAGEASR LPHYNKRSTI TSREIQTAVR 101 LLLPGELAKH AVSEGTKAVT KYTSAK ``` | | |
| Evidence | Indistinguishable | | |
| Peptide list | 25\*\*, 46\*\*, 309\*, 652\*\*, 1119\*\* | | |
| Peptides | 25\*\* | Confidence | Green |
| Runs | 1, 2, 3, 4 |
| Relation | NonDiscriminating |
| Proteins | H2B1N\_HUMAN, H2B1M\_HUMAN, H2B2F\_HUMAN, H2B1K\_HUMAN, H2BFS\_HUMAN, H2B1L\_HUMAN, H2B1D\_HUMAN, H2B1C\_HUMAN, H2B1H\_HUMAN, H2B2E\_HUMAN, H2B1B\_HUMAN, H2B1J\_HUMAN, H2B1O\_HUMAN, H2B3B\_HUMAN, H2B1A\_HUMAN |
| Sequence | ``` QVHPDTGISSK ``` |
| Position | 48 |
| PTMs | none |
| 46\*\* | Confidence | Green |
| Runs | 1, 2, 3, 4 |
| Relation | NonDiscriminating |
| Proteins | H2B1N\_HUMAN, H2B1M\_HUMAN, H2B2F\_HUMAN, H2B1K\_HUMAN, H2BFS\_HUMAN, H2B1L\_HUMAN, H2B1D\_HUMAN, H2B1C\_HUMAN, H2B1H\_HUMAN, H2B2E\_HUMAN, H2B1B\_HUMAN, H2B1J\_HUMAN, H2B1O\_HUMAN, H2B1A\_HUMAN |
| Sequence | ``` EIQTAVR ``` |
| Position | 94 |
| PTMs | Variant #1: none Variant #2: Deamidation+Q(3) |
| 309\* | Confidence | Green |
| Runs | 1, 2, 3, 4 |
| Relation | Discriminating |
| Proteins | H2B1N\_HUMAN, H2B1M\_HUMAN, H2B2F\_HUMAN, H2B1K\_HUMAN, H2BFS\_HUMAN, H2B1L\_HUMAN, H2B1D\_HUMAN, H2B1C\_HUMAN, H2B1H\_HUMAN |
| Sequence | ``` KESYSVYVYK ``` |
| Position | 35 |
| PTMs | none |
| 652\*\* | Confidence | Green |
| Runs | 1, 2, 3, 4 |
| Relation | NonDiscriminating |
| Proteins | H2B1N\_HUMAN, H2B1M\_HUMAN, H2B2F\_HUMAN, H2B1K\_HUMAN, H2BFS\_HUMAN, H2B1L\_HUMAN, H2B1D\_HUMAN, H2B1C\_HUMAN, H2B1H\_HUMAN, H2B2E\_HUMAN, H2B1B\_HUMAN, H2B1J\_HUMAN, H2B1O\_HUMAN, H2B3B\_HUMAN, H2B1A\_HUMAN |
| Sequence | ``` LLLPGELAK ``` |
| Position | 101 |
| PTMs | none |
| 1119\*\* | Confidence | Green |
| Runs | 1, 2, 3, 4 |
| Relation | NonDiscriminating |
| Proteins | H2B1N\_HUMAN, H2B1M\_HUMAN, H2B2F\_HUMAN, H2B1K\_HUMAN, H2BFS\_HUMAN, H2B1L\_HUMAN, H2B1D\_HUMAN, H2B1C\_HUMAN, H2B1H\_HUMAN, H2B2E\_HUMAN, H2B1B\_HUMAN, H2B1J\_HUMAN, H2B1O\_HUMAN, H2B3B\_HUMAN |
| Sequence | ``` AMGIMNSFVNDIFER ``` |
| Position | 59 |
| PTMs | none |

  

Protein Q99877

| Name | H2B1N\_HUMAN | | |
| Description | Histone H2B type 1 N OS Homo sapiens GN HIST1H2BN PE 1 SV 3 | | |
| Sequence | ```   1 MPEPSKSAPA PKKGSKKAVT KAQKKDGKKR KRSRKESYSV YVYKVLKQVH PDTGISSKAM GIMNSFVNDI FERIAGEASR LAHYNKRSTI TSREIQTAVR 101 LLLPGELAKH AVSEGTKAVT KYTSSK ``` | | |
| Evidence | Indistinguishable | | |
| Peptide list | 25\*\*, 46\*\*, 309\*, 652\*\*, 1119\*\* | | |
| Peptides | 25\*\* | Confidence | Green |
| Runs | 1, 2, 3, 4 |
| Relation | NonDiscriminating |
| Proteins | H2B1N\_HUMAN, H2B1M\_HUMAN, H2B2F\_HUMAN, H2B1K\_HUMAN, H2BFS\_HUMAN, H2B1L\_HUMAN, H2B1D\_HUMAN, H2B1C\_HUMAN, H2B1H\_HUMAN, H2B2E\_HUMAN, H2B1B\_HUMAN, H2B1J\_HUMAN, H2B1O\_HUMAN, H2B3B\_HUMAN, H2B1A\_HUMAN |
| Sequence | ``` QVHPDTGISSK ``` |
| Position | 48 |
| PTMs | none |
| 46\*\* | Confidence | Green |
| Runs | 1, 2, 3, 4 |
| Relation | NonDiscriminating |
| Proteins | H2B1N\_HUMAN, H2B1M\_HUMAN, H2B2F\_HUMAN, H2B1K\_HUMAN, H2BFS\_HUMAN, H2B1L\_HUMAN, H2B1D\_HUMAN, H2B1C\_HUMAN, H2B1H\_HUMAN, H2B2E\_HUMAN, H2B1B\_HUMAN, H2B1J\_HUMAN, H2B1O\_HUMAN, H2B1A\_HUMAN |
| Sequence | ``` EIQTAVR ``` |
| Position | 94 |
| PTMs | Variant #1: none Variant #2: Deamidation+Q(3) |
| 309\* | Confidence | Green |
| Runs | 1, 2, 3, 4 |
| Relation | Discriminating |
| Proteins | H2B1N\_HUMAN, H2B1M\_HUMAN, H2B2F\_HUMAN, H2B1K\_HUMAN, H2BFS\_HUMAN, H2B1L\_HUMAN, H2B1D\_HUMAN, H2B1C\_HUMAN, H2B1H\_HUMAN |
| Sequence | ``` KESYSVYVYK ``` |
| Position | 35 |
| PTMs | none |
| 652\*\* | Confidence | Green |
| Runs | 1, 2, 3, 4 |
| Relation | NonDiscriminating |
| Proteins | H2B1N\_HUMAN, H2B1M\_HUMAN, H2B2F\_HUMAN, H2B1K\_HUMAN, H2BFS\_HUMAN, H2B1L\_HUMAN, H2B1D\_HUMAN, H2B1C\_HUMAN, H2B1H\_HUMAN, H2B2E\_HUMAN, H2B1B\_HUMAN, H2B1J\_HUMAN, H2B1O\_HUMAN, H2B3B\_HUMAN, H2B1A\_HUMAN |
| Sequence | ``` LLLPGELAK ``` |
| Position | 101 |
| PTMs | none |
| 1119\*\* | Confidence | Green |
| Runs | 1, 2, 3, 4 |
| Relation | NonDiscriminating |
| Proteins | H2B1N\_HUMAN, H2B1M\_HUMAN, H2B2F\_HUMAN, H2B1K\_HUMAN, H2BFS\_HUMAN, H2B1L\_HUMAN, H2B1D\_HUMAN, H2B1C\_HUMAN, H2B1H\_HUMAN, H2B2E\_HUMAN, H2B1B\_HUMAN, H2B1J\_HUMAN, H2B1O\_HUMAN, H2B3B\_HUMAN |
| Sequence | ``` AMGIMNSFVNDIFER ``` |
| Position | 59 |
| PTMs | none |

  

Protein Q99879

| Name | H2B1M\_HUMAN | | |
| Description | Histone H2B type 1 M OS Homo sapiens GN HIST1H2BM PE 1 SV 3 | | |
| Sequence | ```   1 MPEPVKSAPV PKKGSKKAIN KAQKKDGKKR KRSRKESYSV YVYKVLKQVH PDTGISSKAM GIMNSFVNDI FERIAGEASR LAHYNKRSTI TSREIQTAVR 101 LLLPGELAKH AVSEGTKAVT KYTSSK ``` | | |
| Evidence | Indistinguishable | | |
| Peptide list | 25\*\*, 46\*\*, 309\*, 652\*\*, 1119\*\* | | |
| Peptides | 25\*\* | Confidence | Green |
| Runs | 1, 2, 3, 4 |
| Relation | NonDiscriminating |
| Proteins | H2B1N\_HUMAN, H2B1M\_HUMAN, H2B2F\_HUMAN, H2B1K\_HUMAN, H2BFS\_HUMAN, H2B1L\_HUMAN, H2B1D\_HUMAN, H2B1C\_HUMAN, H2B1H\_HUMAN, H2B2E\_HUMAN, H2B1B\_HUMAN, H2B1J\_HUMAN, H2B1O\_HUMAN, H2B3B\_HUMAN, H2B1A\_HUMAN |
| Sequence | ``` QVHPDTGISSK ``` |
| Position | 48 |
| PTMs | none |
| 46\*\* | Confidence | Green |
| Runs | 1, 2, 3, 4 |
| Relation | NonDiscriminating |
| Proteins | H2B1N\_HUMAN, H2B1M\_HUMAN, H2B2F\_HUMAN, H2B1K\_HUMAN, H2BFS\_HUMAN, H2B1L\_HUMAN, H2B1D\_HUMAN, H2B1C\_HUMAN, H2B1H\_HUMAN, H2B2E\_HUMAN, H2B1B\_HUMAN, H2B1J\_HUMAN, H2B1O\_HUMAN, H2B1A\_HUMAN |
| Sequence | ``` EIQTAVR ``` |
| Position | 94 |
| PTMs | Variant #1: none Variant #2: Deamidation+Q(3) |
| 309\* | Confidence | Green |
| Runs | 1, 2, 3, 4 |
| Relation | Discriminating |
| Proteins | H2B1N\_HUMAN, H2B1M\_HUMAN, H2B2F\_HUMAN, H2B1K\_HUMAN, H2BFS\_HUMAN, H2B1L\_HUMAN, H2B1D\_HUMAN, H2B1C\_HUMAN, H2B1H\_HUMAN |
| Sequence | ``` KESYSVYVYK ``` |
| Position | 35 |
| PTMs | none |
| 652\*\* | Confidence | Green |
| Runs | 1, 2, 3, 4 |
| Relation | NonDiscriminating |
| Proteins | H2B1N\_HUMAN, H2B1M\_HUMAN, H2B2F\_HUMAN, H2B1K\_HUMAN, H2BFS\_HUMAN, H2B1L\_HUMAN, H2B1D\_HUMAN, H2B1C\_HUMAN, H2B1H\_HUMAN, H2B2E\_HUMAN, H2B1B\_HUMAN, H2B1J\_HUMAN, H2B1O\_HUMAN, H2B3B\_HUMAN, H2B1A\_HUMAN |
| Sequence | ``` LLLPGELAK ``` |
| Position | 101 |
| PTMs | none |
| 1119\*\* | Confidence | Green |
| Runs | 1, 2, 3, 4 |
| Relation | NonDiscriminating |
| Proteins | H2B1N\_HUMAN, H2B1M\_HUMAN, H2B2F\_HUMAN, H2B1K\_HUMAN, H2BFS\_HUMAN, H2B1L\_HUMAN, H2B1D\_HUMAN, H2B1C\_HUMAN, H2B1H\_HUMAN, H2B2E\_HUMAN, H2B1B\_HUMAN, H2B1J\_HUMAN, H2B1O\_HUMAN, H2B3B\_HUMAN |
| Sequence | ``` AMGIMNSFVNDIFER ``` |
| Position | 59 |
| PTMs | none |

  

Protein Q5QNW6

| Name | H2B2F\_HUMAN | | |
| Description | Histone H2B type 2 F OS Homo sapiens GN HIST2H2BF PE 1 SV 3 | | |
| Sequence | ```   1 MPDPAKSAPA PKKGSKKAVT KVQKKDGKKR KRSRKESYSV YVYKVLKQVH PDTGISSKAM GIMNSFVNDI FERIAGEASR LAHYNKRSTI TSREIQTAVR 101 LLLPGELAKH AVSEGTKAVT KYTSSK ``` | | |
| Evidence | Indistinguishable | | |
| Peptide list | 25\*\*, 46\*\*, 309\*, 652\*\*, 1119\*\* | | |
| Peptides | 25\*\* | Confidence | Green |
| Runs | 1, 2, 3, 4 |
| Relation | NonDiscriminating |
| Proteins | H2B1N\_HUMAN, H2B1M\_HUMAN, H2B2F\_HUMAN, H2B1K\_HUMAN, H2BFS\_HUMAN, H2B1L\_HUMAN, H2B1D\_HUMAN, H2B1C\_HUMAN, H2B1H\_HUMAN, H2B2E\_HUMAN, H2B1B\_HUMAN, H2B1J\_HUMAN, H2B1O\_HUMAN, H2B3B\_HUMAN, H2B1A\_HUMAN |
| Sequence | ``` QVHPDTGISSK ``` |
| Position | 48 |
| PTMs | none |
| 46\*\* | Confidence | Green |
| Runs | 1, 2, 3, 4 |
| Relation | NonDiscriminating |
| Proteins | H2B1N\_HUMAN, H2B1M\_HUMAN, H2B2F\_HUMAN, H2B1K\_HUMAN, H2BFS\_HUMAN, H2B1L\_HUMAN, H2B1D\_HUMAN, H2B1C\_HUMAN, H2B1H\_HUMAN, H2B2E\_HUMAN, H2B1B\_HUMAN, H2B1J\_HUMAN, H2B1O\_HUMAN, H2B1A\_HUMAN |
| Sequence | ``` EIQTAVR ``` |
| Position | 94 |
| PTMs | Variant #1: none Variant #2: Deamidation+Q(3) |
| 309\* | Confidence | Green |
| Runs | 1, 2, 3, 4 |
| Relation | Discriminating |
| Proteins | H2B1N\_HUMAN, H2B1M\_HUMAN, H2B2F\_HUMAN, H2B1K\_HUMAN, H2BFS\_HUMAN, H2B1L\_HUMAN, H2B1D\_HUMAN, H2B1C\_HUMAN, H2B1H\_HUMAN |
| Sequence | ``` KESYSVYVYK ``` |
| Position | 35 |
| PTMs | none |
| 652\*\* | Confidence | Green |
| Runs | 1, 2, 3, 4 |
| Relation | NonDiscriminating |
| Proteins | H2B1N\_HUMAN, H2B1M\_HUMAN, H2B2F\_HUMAN, H2B1K\_HUMAN, H2BFS\_HUMAN, H2B1L\_HUMAN, H2B1D\_HUMAN, H2B1C\_HUMAN, H2B1H\_HUMAN, H2B2E\_HUMAN, H2B1B\_HUMAN, H2B1J\_HUMAN, H2B1O\_HUMAN, H2B3B\_HUMAN, H2B1A\_HUMAN |
| Sequence | ``` LLLPGELAK ``` |
| Position | 101 |
| PTMs | none |
| 1119\*\* | Confidence | Green |
| Runs | 1, 2, 3, 4 |
| Relation | NonDiscriminating |
| Proteins | H2B1N\_HUMAN, H2B1M\_HUMAN, H2B2F\_HUMAN, H2B1K\_HUMAN, H2BFS\_HUMAN, H2B1L\_HUMAN, H2B1D\_HUMAN, H2B1C\_HUMAN, H2B1H\_HUMAN, H2B2E\_HUMAN, H2B1B\_HUMAN, H2B1J\_HUMAN, H2B1O\_HUMAN, H2B3B\_HUMAN |
| Sequence | ``` AMGIMNSFVNDIFER ``` |
| Position | 59 |
| PTMs | none |

  

Protein O60814

| Name | H2B1K\_HUMAN | | |
| Description | Histone H2B type 1 K OS Homo sapiens GN HIST1H2BK PE 1 SV 3 | | |
| Sequence | ```   1 MPEPAKSAPA PKKGSKKAVT KAQKKDGKKR KRSRKESYSV YVYKVLKQVH PDTGISSKAM GIMNSFVNDI FERIAGEASR LAHYNKRSTI TSREIQTAVR 101 LLLPGELAKH AVSEGTKAVT KYTSAK ``` | | |
| Evidence | Indistinguishable | | |
| Peptide list | 25\*\*, 46\*\*, 309\*, 652\*\*, 1119\*\* | | |
| Peptides | 25\*\* | Confidence | Green |
| Runs | 1, 2, 3, 4 |
| Relation | NonDiscriminating |
| Proteins | H2B1N\_HUMAN, H2B1M\_HUMAN, H2B2F\_HUMAN, H2B1K\_HUMAN, H2BFS\_HUMAN, H2B1L\_HUMAN, H2B1D\_HUMAN, H2B1C\_HUMAN, H2B1H\_HUMAN, H2B2E\_HUMAN, H2B1B\_HUMAN, H2B1J\_HUMAN, H2B1O\_HUMAN, H2B3B\_HUMAN, H2B1A\_HUMAN |
| Sequence | ``` QVHPDTGISSK ``` |
| Position | 48 |
| PTMs | none |
| 46\*\* | Confidence | Green |
| Runs | 1, 2, 3, 4 |
| Relation | NonDiscriminating |
| Proteins | H2B1N\_HUMAN, H2B1M\_HUMAN, H2B2F\_HUMAN, H2B1K\_HUMAN, H2BFS\_HUMAN, H2B1L\_HUMAN, H2B1D\_HUMAN, H2B1C\_HUMAN, H2B1H\_HUMAN, H2B2E\_HUMAN, H2B1B\_HUMAN, H2B1J\_HUMAN, H2B1O\_HUMAN, H2B1A\_HUMAN |
| Sequence | ``` EIQTAVR ``` |
| Position | 94 |
| PTMs | Variant #1: none Variant #2: Deamidation+Q(3) |
| 309\* | Confidence | Green |
| Runs | 1, 2, 3, 4 |
| Relation | Discriminating |
| Proteins | H2B1N\_HUMAN, H2B1M\_HUMAN, H2B2F\_HUMAN, H2B1K\_HUMAN, H2BFS\_HUMAN, H2B1L\_HUMAN, H2B1D\_HUMAN, H2B1C\_HUMAN, H2B1H\_HUMAN |
| Sequence | ``` KESYSVYVYK ``` |
| Position | 35 |
| PTMs | none |
| 652\*\* | Confidence | Green |
| Runs | 1, 2, 3, 4 |
| Relation | NonDiscriminating |
| Proteins | H2B1N\_HUMAN, H2B1M\_HUMAN, H2B2F\_HUMAN, H2B1K\_HUMAN, H2BFS\_HUMAN, H2B1L\_HUMAN, H2B1D\_HUMAN, H2B1C\_HUMAN, H2B1H\_HUMAN, H2B2E\_HUMAN, H2B1B\_HUMAN, H2B1J\_HUMAN, H2B1O\_HUMAN, H2B3B\_HUMAN, H2B1A\_HUMAN |
| Sequence | ``` LLLPGELAK ``` |
| Position | 101 |
| PTMs | none |
| 1119\*\* | Confidence | Green |
| Runs | 1, 2, 3, 4 |
| Relation | NonDiscriminating |
| Proteins | H2B1N\_HUMAN, H2B1M\_HUMAN, H2B2F\_HUMAN, H2B1K\_HUMAN, H2BFS\_HUMAN, H2B1L\_HUMAN, H2B1D\_HUMAN, H2B1C\_HUMAN, H2B1H\_HUMAN, H2B2E\_HUMAN, H2B1B\_HUMAN, H2B1J\_HUMAN, H2B1O\_HUMAN, H2B3B\_HUMAN |
| Sequence | ``` AMGIMNSFVNDIFER ``` |
| Position | 59 |
| PTMs | none |

  

Protein Q99880

| Name | H2B1L\_HUMAN | | |
| Description | Histone H2B type 1 L OS Homo sapiens GN HIST1H2BL PE 1 SV 3 | | |
| Sequence | ```   1 MPELAKSAPA PKKGSKKAVT KAQKKDGKKR KRSRKESYSV YVYKVLKQVH PDTGISSKAM GIMNSFVNDI FERIASEASR LAHYNKRSTI TSREIQTAVR 101 LLLPGELAKH AVSEGTKAVT KYTSSK ``` | | |
| Evidence | Indistinguishable | | |
| Peptide list | 25\*\*, 46\*\*, 309\*, 652\*\*, 1119\*\* | | |
| Peptides | 25\*\* | Confidence | Green |
| Runs | 1, 2, 3, 4 |
| Relation | NonDiscriminating |
| Proteins | H2B1N\_HUMAN, H2B1M\_HUMAN, H2B2F\_HUMAN, H2B1K\_HUMAN, H2BFS\_HUMAN, H2B1L\_HUMAN, H2B1D\_HUMAN, H2B1C\_HUMAN, H2B1H\_HUMAN, H2B2E\_HUMAN, H2B1B\_HUMAN, H2B1J\_HUMAN, H2B1O\_HUMAN, H2B3B\_HUMAN, H2B1A\_HUMAN |
| Sequence | ``` QVHPDTGISSK ``` |
| Position | 48 |
| PTMs | none |
| 46\*\* | Confidence | Green |
| Runs | 1, 2, 3, 4 |
| Relation | NonDiscriminating |
| Proteins | H2B1N\_HUMAN, H2B1M\_HUMAN, H2B2F\_HUMAN, H2B1K\_HUMAN, H2BFS\_HUMAN, H2B1L\_HUMAN, H2B1D\_HUMAN, H2B1C\_HUMAN, H2B1H\_HUMAN, H2B2E\_HUMAN, H2B1B\_HUMAN, H2B1J\_HUMAN, H2B1O\_HUMAN, H2B1A\_HUMAN |
| Sequence | ``` EIQTAVR ``` |
| Position | 94 |
| PTMs | Variant #1: none Variant #2: Deamidation+Q(3) |
| 309\* | Confidence | Green |
| Runs | 1, 2, 3, 4 |
| Relation | Discriminating |
| Proteins | H2B1N\_HUMAN, H2B1M\_HUMAN, H2B2F\_HUMAN, H2B1K\_HUMAN, H2BFS\_HUMAN, H2B1L\_HUMAN, H2B1D\_HUMAN, H2B1C\_HUMAN, H2B1H\_HUMAN |
| Sequence | ``` KESYSVYVYK ``` |
| Position | 35 |
| PTMs | none |
| 652\*\* | Confidence | Green |
| Runs | 1, 2, 3, 4 |
| Relation | NonDiscriminating |
| Proteins | H2B1N\_HUMAN, H2B1M\_HUMAN, H2B2F\_HUMAN, H2B1K\_HUMAN, H2BFS\_HUMAN, H2B1L\_HUMAN, H2B1D\_HUMAN, H2B1C\_HUMAN, H2B1H\_HUMAN, H2B2E\_HUMAN, H2B1B\_HUMAN, H2B1J\_HUMAN, H2B1O\_HUMAN, H2B3B\_HUMAN, H2B1A\_HUMAN |
| Sequence | ``` LLLPGELAK ``` |
| Position | 101 |
| PTMs | none |
| 1119\*\* | Confidence | Green |
| Runs | 1, 2, 3, 4 |
| Relation | NonDiscriminating |
| Proteins | H2B1N\_HUMAN, H2B1M\_HUMAN, H2B2F\_HUMAN, H2B1K\_HUMAN, H2BFS\_HUMAN, H2B1L\_HUMAN, H2B1D\_HUMAN, H2B1C\_HUMAN, H2B1H\_HUMAN, H2B2E\_HUMAN, H2B1B\_HUMAN, H2B1J\_HUMAN, H2B1O\_HUMAN, H2B3B\_HUMAN |
| Sequence | ``` AMGIMNSFVNDIFER ``` |
| Position | 59 |
| PTMs | none |

  

Protein P58876

| Name | H2B1D\_HUMAN | | |
| Description | Histone H2B type 1 D OS Homo sapiens GN HIST1H2BD PE 1 SV 2 | | |
| Sequence | ```   1 MPEPTKSAPA PKKGSKKAVT KAQKKDGKKR KRSRKESYSV YVYKVLKQVH PDTGISSKAM GIMNSFVNDI FERIAGEASR LAHYNKRSTI TSREIQTAVR 101 LLLPGELAKH AVSEGTKAVT KYTSSK ``` | | |
| Evidence | Indistinguishable | | |
| Peptide list | 25\*\*, 46\*\*, 309\*, 652\*\*, 1119\*\* | | |
| Peptides | 25\*\* | Confidence | Green |
| Runs | 1, 2, 3, 4 |
| Relation | NonDiscriminating |
| Proteins | H2B1N\_HUMAN, H2B1M\_HUMAN, H2B2F\_HUMAN, H2B1K\_HUMAN, H2BFS\_HUMAN, H2B1L\_HUMAN, H2B1D\_HUMAN, H2B1C\_HUMAN, H2B1H\_HUMAN, H2B2E\_HUMAN, H2B1B\_HUMAN, H2B1J\_HUMAN, H2B1O\_HUMAN, H2B3B\_HUMAN, H2B1A\_HUMAN |
| Sequence | ``` QVHPDTGISSK ``` |
| Position | 48 |
| PTMs | none |
| 46\*\* | Confidence | Green |
| Runs | 1, 2, 3, 4 |
| Relation | NonDiscriminating |
| Proteins | H2B1N\_HUMAN, H2B1M\_HUMAN, H2B2F\_HUMAN, H2B1K\_HUMAN, H2BFS\_HUMAN, H2B1L\_HUMAN, H2B1D\_HUMAN, H2B1C\_HUMAN, H2B1H\_HUMAN, H2B2E\_HUMAN, H2B1B\_HUMAN, H2B1J\_HUMAN, H2B1O\_HUMAN, H2B1A\_HUMAN |
| Sequence | ``` EIQTAVR ``` |
| Position | 94 |
| PTMs | Variant #1: none Variant #2: Deamidation+Q(3) |
| 309\* | Confidence | Green |
| Runs | 1, 2, 3, 4 |
| Relation | Discriminating |
| Proteins | H2B1N\_HUMAN, H2B1M\_HUMAN, H2B2F\_HUMAN, H2B1K\_HUMAN, H2BFS\_HUMAN, H2B1L\_HUMAN, H2B1D\_HUMAN, H2B1C\_HUMAN, H2B1H\_HUMAN |
| Sequence | ``` KESYSVYVYK ``` |
| Position | 35 |
| PTMs | none |
| 652\*\* | Confidence | Green |
| Runs | 1, 2, 3, 4 |
| Relation | NonDiscriminating |
| Proteins | H2B1N\_HUMAN, H2B1M\_HUMAN, H2B2F\_HUMAN, H2B1K\_HUMAN, H2BFS\_HUMAN, H2B1L\_HUMAN, H2B1D\_HUMAN, H2B1C\_HUMAN, H2B1H\_HUMAN, H2B2E\_HUMAN, H2B1B\_HUMAN, H2B1J\_HUMAN, H2B1O\_HUMAN, H2B3B\_HUMAN, H2B1A\_HUMAN |
| Sequence | ``` LLLPGELAK ``` |
| Position | 101 |
| PTMs | none |
| 1119\*\* | Confidence | Green |
| Runs | 1, 2, 3, 4 |
| Relation | NonDiscriminating |
| Proteins | H2B1N\_HUMAN, H2B1M\_HUMAN, H2B2F\_HUMAN, H2B1K\_HUMAN, H2BFS\_HUMAN, H2B1L\_HUMAN, H2B1D\_HUMAN, H2B1C\_HUMAN, H2B1H\_HUMAN, H2B2E\_HUMAN, H2B1B\_HUMAN, H2B1J\_HUMAN, H2B1O\_HUMAN, H2B3B\_HUMAN |
| Sequence | ``` AMGIMNSFVNDIFER ``` |
| Position | 59 |
| PTMs | none |

  

Protein P62807

| Name | H2B1C\_HUMAN | | |
| Description | Histone H2B type 1 C E F G I OS Homo sapiens GN HIST1H2BC PE 1 SV 4 | | |
| Sequence | ```   1 MPEPAKSAPA PKKGSKKAVT KAQKKDGKKR KRSRKESYSV YVYKVLKQVH PDTGISSKAM GIMNSFVNDI FERIAGEASR LAHYNKRSTI TSREIQTAVR 101 LLLPGELAKH AVSEGTKAVT KYTSSK ``` | | |
| Evidence | Indistinguishable | | |
| Peptide list | 25\*\*, 46\*\*, 309\*, 652\*\*, 1119\*\* | | |
| Peptides | 25\*\* | Confidence | Green |
| Runs | 1, 2, 3, 4 |
| Relation | NonDiscriminating |
| Proteins | H2B1N\_HUMAN, H2B1M\_HUMAN, H2B2F\_HUMAN, H2B1K\_HUMAN, H2BFS\_HUMAN, H2B1L\_HUMAN, H2B1D\_HUMAN, H2B1C\_HUMAN, H2B1H\_HUMAN, H2B2E\_HUMAN, H2B1B\_HUMAN, H2B1J\_HUMAN, H2B1O\_HUMAN, H2B3B\_HUMAN, H2B1A\_HUMAN |
| Sequence | ``` QVHPDTGISSK ``` |
| Position | 48 |
| PTMs | none |
| 46\*\* | Confidence | Green |
| Runs | 1, 2, 3, 4 |
| Relation | NonDiscriminating |
| Proteins | H2B1N\_HUMAN, H2B1M\_HUMAN, H2B2F\_HUMAN, H2B1K\_HUMAN, H2BFS\_HUMAN, H2B1L\_HUMAN, H2B1D\_HUMAN, H2B1C\_HUMAN, H2B1H\_HUMAN, H2B2E\_HUMAN, H2B1B\_HUMAN, H2B1J\_HUMAN, H2B1O\_HUMAN, H2B1A\_HUMAN |
| Sequence | ``` EIQTAVR ``` |
| Position | 94 |
| PTMs | Variant #1: none Variant #2: Deamidation+Q(3) |
| 309\* | Confidence | Green |
| Runs | 1, 2, 3, 4 |
| Relation | Discriminating |
| Proteins | H2B1N\_HUMAN, H2B1M\_HUMAN, H2B2F\_HUMAN, H2B1K\_HUMAN, H2BFS\_HUMAN, H2B1L\_HUMAN, H2B1D\_HUMAN, H2B1C\_HUMAN, H2B1H\_HUMAN |
| Sequence | ``` KESYSVYVYK ``` |
| Position | 35 |
| PTMs | none |
| 652\*\* | Confidence | Green |
| Runs | 1, 2, 3, 4 |
| Relation | NonDiscriminating |
| Proteins | H2B1N\_HUMAN, H2B1M\_HUMAN, H2B2F\_HUMAN, H2B1K\_HUMAN, H2BFS\_HUMAN, H2B1L\_HUMAN, H2B1D\_HUMAN, H2B1C\_HUMAN, H2B1H\_HUMAN, H2B2E\_HUMAN, H2B1B\_HUMAN, H2B1J\_HUMAN, H2B1O\_HUMAN, H2B3B\_HUMAN, H2B1A\_HUMAN |
| Sequence | ``` LLLPGELAK ``` |
| Position | 101 |
| PTMs | none |
| 1119\*\* | Confidence | Green |
| Runs | 1, 2, 3, 4 |
| Relation | NonDiscriminating |
| Proteins | H2B1N\_HUMAN, H2B1M\_HUMAN, H2B2F\_HUMAN, H2B1K\_HUMAN, H2BFS\_HUMAN, H2B1L\_HUMAN, H2B1D\_HUMAN, H2B1C\_HUMAN, H2B1H\_HUMAN, H2B2E\_HUMAN, H2B1B\_HUMAN, H2B1J\_HUMAN, H2B1O\_HUMAN, H2B3B\_HUMAN |
| Sequence | ``` AMGIMNSFVNDIFER ``` |
| Position | 59 |
| PTMs | none |

  

Protein Q93079

| Name | H2B1H\_HUMAN | | |
| Description | Histone H2B type 1 H OS Homo sapiens GN HIST1H2BH PE 1 SV 3 | | |
| Sequence | ```   1 MPDPAKSAPA PKKGSKKAVT KAQKKDGKKR KRSRKESYSV YVYKVLKQVH PDTGISSKAM GIMNSFVNDI FERIAGEASR LAHYNKRSTI TSREIQTAVR 101 LLLPGELAKH AVSEGTKAVT KYTSSK ``` | | |
| Evidence | Indistinguishable | | |
| Peptide list | 25\*\*, 46\*\*, 309\*, 652\*\*, 1119\*\* | | |
| Peptides | 25\*\* | Confidence | Green |
| Runs | 1, 2, 3, 4 |
| Relation | NonDiscriminating |
| Proteins | H2B1N\_HUMAN, H2B1M\_HUMAN, H2B2F\_HUMAN, H2B1K\_HUMAN, H2BFS\_HUMAN, H2B1L\_HUMAN, H2B1D\_HUMAN, H2B1C\_HUMAN, H2B1H\_HUMAN, H2B2E\_HUMAN, H2B1B\_HUMAN, H2B1J\_HUMAN, H2B1O\_HUMAN, H2B3B\_HUMAN, H2B1A\_HUMAN |
| Sequence | ``` QVHPDTGISSK ``` |
| Position | 48 |
| PTMs | none |
| 46\*\* | Confidence | Green |
| Runs | 1, 2, 3, 4 |
| Relation | NonDiscriminating |
| Proteins | H2B1N\_HUMAN, H2B1M\_HUMAN, H2B2F\_HUMAN, H2B1K\_HUMAN, H2BFS\_HUMAN, H2B1L\_HUMAN, H2B1D\_HUMAN, H2B1C\_HUMAN, H2B1H\_HUMAN, H2B2E\_HUMAN, H2B1B\_HUMAN, H2B1J\_HUMAN, H2B1O\_HUMAN, H2B1A\_HUMAN |
| Sequence | ``` EIQTAVR ``` |
| Position | 94 |
| PTMs | Variant #1: none Variant #2: Deamidation+Q(3) |
| 309\* | Confidence | Green |
| Runs | 1, 2, 3, 4 |
| Relation | Discriminating |
| Proteins | H2B1N\_HUMAN, H2B1M\_HUMAN, H2B2F\_HUMAN, H2B1K\_HUMAN, H2BFS\_HUMAN, H2B1L\_HUMAN, H2B1D\_HUMAN, H2B1C\_HUMAN, H2B1H\_HUMAN |
| Sequence | ``` KESYSVYVYK ``` |
| Position | 35 |
| PTMs | none |
| 652\*\* | Confidence | Green |
| Runs | 1, 2, 3, 4 |
| Relation | NonDiscriminating |
| Proteins | H2B1N\_HUMAN, H2B1M\_HUMAN, H2B2F\_HUMAN, H2B1K\_HUMAN, H2BFS\_HUMAN, H2B1L\_HUMAN, H2B1D\_HUMAN, H2B1C\_HUMAN, H2B1H\_HUMAN, H2B2E\_HUMAN, H2B1B\_HUMAN, H2B1J\_HUMAN, H2B1O\_HUMAN, H2B3B\_HUMAN, H2B1A\_HUMAN |
| Sequence | ``` LLLPGELAK ``` |
| Position | 101 |
| PTMs | none |
| 1119\*\* | Confidence | Green |
| Runs | 1, 2, 3, 4 |
| Relation | NonDiscriminating |
| Proteins | H2B1N\_HUMAN, H2B1M\_HUMAN, H2B2F\_HUMAN, H2B1K\_HUMAN, H2BFS\_HUMAN, H2B1L\_HUMAN, H2B1D\_HUMAN, H2B1C\_HUMAN, H2B1H\_HUMAN, H2B2E\_HUMAN, H2B1B\_HUMAN, H2B1J\_HUMAN, H2B1O\_HUMAN, H2B3B\_HUMAN |
| Sequence | ``` AMGIMNSFVNDIFER ``` |
| Position | 59 |
| PTMs | none |

  

Protein P33778

| Name | H2B1B\_HUMAN | | |
| Description | Histone H2B type 1 B OS Homo sapiens GN HIST1H2BB PE 1 SV 2 | | |
| Sequence | ```   1 MPEPSKSAPA PKKGSKKAIT KAQKKDGKKR KRSRKESYSI YVYKVLKQVH PDTGISSKAM GIMNSFVNDI FERIAGEASR LAHYNKRSTI TSREIQTAVR 101 LLLPGELAKH AVSEGTKAVT KYTSSK ``` | | |
| Evidence | Indistinguishable | | |
| Peptide list | 25\*\*, 46\*\*, 507\*, 652\*\*, 1119\*\* | | |
| Peptides | 25\*\* | Confidence | Green |
| Runs | 1, 2, 3, 4 |
| Relation | NonDiscriminating |
| Proteins | H2B1N\_HUMAN, H2B1M\_HUMAN, H2B2F\_HUMAN, H2B1K\_HUMAN, H2BFS\_HUMAN, H2B1L\_HUMAN, H2B1D\_HUMAN, H2B1C\_HUMAN, H2B1H\_HUMAN, H2B2E\_HUMAN, H2B1B\_HUMAN, H2B1J\_HUMAN, H2B1O\_HUMAN, H2B3B\_HUMAN, H2B1A\_HUMAN |
| Sequence | ``` QVHPDTGISSK ``` |
| Position | 48 |
| PTMs | none |
| 46\*\* | Confidence | Green |
| Runs | 1, 2, 3, 4 |
| Relation | NonDiscriminating |
| Proteins | H2B1N\_HUMAN, H2B1M\_HUMAN, H2B2F\_HUMAN, H2B1K\_HUMAN, H2BFS\_HUMAN, H2B1L\_HUMAN, H2B1D\_HUMAN, H2B1C\_HUMAN, H2B1H\_HUMAN, H2B2E\_HUMAN, H2B1B\_HUMAN, H2B1J\_HUMAN, H2B1O\_HUMAN, H2B1A\_HUMAN |
| Sequence | ``` EIQTAVR ``` |
| Position | 94 |
| PTMs | Variant #1: none Variant #2: Deamidation+Q(3) |
| 507\* | Confidence | Green |
| Runs | 1, 2, 3, 4 |
| Relation | Discriminating |
| Proteins | H2B2E\_HUMAN, H2B1B\_HUMAN, H2B1J\_HUMAN, H2B1O\_HUMAN, H2B3B\_HUMAN, H2B2C\_HUMAN, H2B2D\_HUMAN |
| Sequence | ``` ESYSIYVYK ``` |
| Position | 36 |
| PTMs | none |
| 652\*\* | Confidence | Green |
| Runs | 1, 2, 3, 4 |
| Relation | NonDiscriminating |
| Proteins | H2B1N\_HUMAN, H2B1M\_HUMAN, H2B2F\_HUMAN, H2B1K\_HUMAN, H2BFS\_HUMAN, H2B1L\_HUMAN, H2B1D\_HUMAN, H2B1C\_HUMAN, H2B1H\_HUMAN, H2B2E\_HUMAN, H2B1B\_HUMAN, H2B1J\_HUMAN, H2B1O\_HUMAN, H2B3B\_HUMAN, H2B1A\_HUMAN |
| Sequence | ``` LLLPGELAK ``` |
| Position | 101 |
| PTMs | none |
| 1119\*\* | Confidence | Green |
| Runs | 1, 2, 3, 4 |
| Relation | NonDiscriminating |
| Proteins | H2B1N\_HUMAN, H2B1M\_HUMAN, H2B2F\_HUMAN, H2B1K\_HUMAN, H2BFS\_HUMAN, H2B1L\_HUMAN, H2B1D\_HUMAN, H2B1C\_HUMAN, H2B1H\_HUMAN, H2B2E\_HUMAN, H2B1B\_HUMAN, H2B1J\_HUMAN, H2B1O\_HUMAN, H2B3B\_HUMAN |
| Sequence | ``` AMGIMNSFVNDIFER ``` |
| Position | 59 |
| PTMs | none |

  

Protein Q16778

| Name | H2B2E\_HUMAN | | |
| Description | Histone H2B type 2 E OS Homo sapiens GN HIST2H2BE PE 1 SV 3 | | |
| Sequence | ```   1 MPEPAKSAPA PKKGSKKAVT KAQKKDGKKR KRSRKESYSI YVYKVLKQVH PDTGISSKAM GIMNSFVNDI FERIAGEASR LAHYNKRSTI TSREIQTAVR 101 LLLPGELAKH AVSEGTKAVT KYTSSK ``` | | |
| Evidence | Indistinguishable | | |
| Peptide list | 25\*\*, 46\*\*, 507\*, 652\*\*, 1119\*\* | | |
| Peptides | 25\*\* | Confidence | Green |
| Runs | 1, 2, 3, 4 |
| Relation | NonDiscriminating |
| Proteins | H2B1N\_HUMAN, H2B1M\_HUMAN, H2B2F\_HUMAN, H2B1K\_HUMAN, H2BFS\_HUMAN, H2B1L\_HUMAN, H2B1D\_HUMAN, H2B1C\_HUMAN, H2B1H\_HUMAN, H2B2E\_HUMAN, H2B1B\_HUMAN, H2B1J\_HUMAN, H2B1O\_HUMAN, H2B3B\_HUMAN, H2B1A\_HUMAN |
| Sequence | ``` QVHPDTGISSK ``` |
| Position | 48 |
| PTMs | none |
| 46\*\* | Confidence | Green |
| Runs | 1, 2, 3, 4 |
| Relation | NonDiscriminating |
| Proteins | H2B1N\_HUMAN, H2B1M\_HUMAN, H2B2F\_HUMAN, H2B1K\_HUMAN, H2BFS\_HUMAN, H2B1L\_HUMAN, H2B1D\_HUMAN, H2B1C\_HUMAN, H2B1H\_HUMAN, H2B2E\_HUMAN, H2B1B\_HUMAN, H2B1J\_HUMAN, H2B1O\_HUMAN, H2B1A\_HUMAN |
| Sequence | ``` EIQTAVR ``` |
| Position | 94 |
| PTMs | Variant #1: none Variant #2: Deamidation+Q(3) |
| 507\* | Confidence | Green |
| Runs | 1, 2, 3, 4 |
| Relation | Discriminating |
| Proteins | H2B2E\_HUMAN, H2B1B\_HUMAN, H2B1J\_HUMAN, H2B1O\_HUMAN, H2B3B\_HUMAN, H2B2C\_HUMAN, H2B2D\_HUMAN |
| Sequence | ``` ESYSIYVYK ``` |
| Position | 36 |
| PTMs | none |
| 652\*\* | Confidence | Green |
| Runs | 1, 2, 3, 4 |
| Relation | NonDiscriminating |
| Proteins | H2B1N\_HUMAN, H2B1M\_HUMAN, H2B2F\_HUMAN, H2B1K\_HUMAN, H2BFS\_HUMAN, H2B1L\_HUMAN, H2B1D\_HUMAN, H2B1C\_HUMAN, H2B1H\_HUMAN, H2B2E\_HUMAN, H2B1B\_HUMAN, H2B1J\_HUMAN, H2B1O\_HUMAN, H2B3B\_HUMAN, H2B1A\_HUMAN |
| Sequence | ``` LLLPGELAK ``` |
| Position | 101 |
| PTMs | none |
| 1119\*\* | Confidence | Green |
| Runs | 1, 2, 3, 4 |
| Relation | NonDiscriminating |
| Proteins | H2B1N\_HUMAN, H2B1M\_HUMAN, H2B2F\_HUMAN, H2B1K\_HUMAN, H2BFS\_HUMAN, H2B1L\_HUMAN, H2B1D\_HUMAN, H2B1C\_HUMAN, H2B1H\_HUMAN, H2B2E\_HUMAN, H2B1B\_HUMAN, H2B1J\_HUMAN, H2B1O\_HUMAN, H2B3B\_HUMAN |
| Sequence | ``` AMGIMNSFVNDIFER ``` |
| Position | 59 |
| PTMs | none |

  

Protein P06899

| Name | H2B1J\_HUMAN | | |
| Description | Histone H2B type 1 J OS Homo sapiens GN HIST1H2BJ PE 1 SV 3 | | |
| Sequence | ```   1 MPEPAKSAPA PKKGSKKAVT KAQKKDGKKR KRSRKESYSI YVYKVLKQVH PDTGISSKAM GIMNSFVNDI FERIAGEASR LAHYNKRSTI TSREIQTAVR 101 LLLPGELAKH AVSEGTKAVT KYTSAK ``` | | |
| Evidence | Indistinguishable | | |
| Peptide list | 25\*\*, 46\*\*, 507\*, 652\*\*, 1119\*\* | | |
| Peptides | 25\*\* | Confidence | Green |
| Runs | 1, 2, 3, 4 |
| Relation | NonDiscriminating |
| Proteins | H2B1N\_HUMAN, H2B1M\_HUMAN, H2B2F\_HUMAN, H2B1K\_HUMAN, H2BFS\_HUMAN, H2B1L\_HUMAN, H2B1D\_HUMAN, H2B1C\_HUMAN, H2B1H\_HUMAN, H2B2E\_HUMAN, H2B1B\_HUMAN, H2B1J\_HUMAN, H2B1O\_HUMAN, H2B3B\_HUMAN, H2B1A\_HUMAN |
| Sequence | ``` QVHPDTGISSK ``` |
| Position | 48 |
| PTMs | none |
| 46\*\* | Confidence | Green |
| Runs | 1, 2, 3, 4 |
| Relation | NonDiscriminating |
| Proteins | H2B1N\_HUMAN, H2B1M\_HUMAN, H2B2F\_HUMAN, H2B1K\_HUMAN, H2BFS\_HUMAN, H2B1L\_HUMAN, H2B1D\_HUMAN, H2B1C\_HUMAN, H2B1H\_HUMAN, H2B2E\_HUMAN, H2B1B\_HUMAN, H2B1J\_HUMAN, H2B1O\_HUMAN, H2B1A\_HUMAN |
| Sequence | ``` EIQTAVR ``` |
| Position | 94 |
| PTMs | Variant #1: none Variant #2: Deamidation+Q(3) |
| 507\* | Confidence | Green |
| Runs | 1, 2, 3, 4 |
| Relation | Discriminating |
| Proteins | H2B2E\_HUMAN, H2B1B\_HUMAN, H2B1J\_HUMAN, H2B1O\_HUMAN, H2B3B\_HUMAN, H2B2C\_HUMAN, H2B2D\_HUMAN |
| Sequence | ``` ESYSIYVYK ``` |
| Position | 36 |
| PTMs | none |
| 652\*\* | Confidence | Green |
| Runs | 1, 2, 3, 4 |
| Relation | NonDiscriminating |
| Proteins | H2B1N\_HUMAN, H2B1M\_HUMAN, H2B2F\_HUMAN, H2B1K\_HUMAN, H2BFS\_HUMAN, H2B1L\_HUMAN, H2B1D\_HUMAN, H2B1C\_HUMAN, H2B1H\_HUMAN, H2B2E\_HUMAN, H2B1B\_HUMAN, H2B1J\_HUMAN, H2B1O\_HUMAN, H2B3B\_HUMAN, H2B1A\_HUMAN |
| Sequence | ``` LLLPGELAK ``` |
| Position | 101 |
| PTMs | none |
| 1119\*\* | Confidence | Green |
| Runs | 1, 2, 3, 4 |
| Relation | NonDiscriminating |
| Proteins | H2B1N\_HUMAN, H2B1M\_HUMAN, H2B2F\_HUMAN, H2B1K\_HUMAN, H2BFS\_HUMAN, H2B1L\_HUMAN, H2B1D\_HUMAN, H2B1C\_HUMAN, H2B1H\_HUMAN, H2B2E\_HUMAN, H2B1B\_HUMAN, H2B1J\_HUMAN, H2B1O\_HUMAN, H2B3B\_HUMAN |
| Sequence | ``` AMGIMNSFVNDIFER ``` |
| Position | 59 |
| PTMs | none |

  

Protein P23527

| Name | H2B1O\_HUMAN | | |
| Description | Histone H2B type 1 O OS Homo sapiens GN HIST1H2BO PE 1 SV 3 | | |
| Sequence | ```   1 MPDPAKSAPA PKKGSKKAVT KAQKKDGKKR KRSRKESYSI YVYKVLKQVH PDTGISSKAM GIMNSFVNDI FERIAGEASR LAHYNKRSTI TSREIQTAVR 101 LLLPGELAKH AVSEGTKAVT KYTSSK ``` | | |
| Evidence | Indistinguishable | | |
| Peptide list | 25\*\*, 46\*\*, 507\*, 652\*\*, 1119\*\* | | |
| Peptides | 25\*\* | Confidence | Green |
| Runs | 1, 2, 3, 4 |
| Relation | NonDiscriminating |
| Proteins | H2B1N\_HUMAN, H2B1M\_HUMAN, H2B2F\_HUMAN, H2B1K\_HUMAN, H2BFS\_HUMAN, H2B1L\_HUMAN, H2B1D\_HUMAN, H2B1C\_HUMAN, H2B1H\_HUMAN, H2B2E\_HUMAN, H2B1B\_HUMAN, H2B1J\_HUMAN, H2B1O\_HUMAN, H2B3B\_HUMAN, H2B1A\_HUMAN |
| Sequence | ``` QVHPDTGISSK ``` |
| Position | 48 |
| PTMs | none |
| 46\*\* | Confidence | Green |
| Runs | 1, 2, 3, 4 |
| Relation | NonDiscriminating |
| Proteins | H2B1N\_HUMAN, H2B1M\_HUMAN, H2B2F\_HUMAN, H2B1K\_HUMAN, H2BFS\_HUMAN, H2B1L\_HUMAN, H2B1D\_HUMAN, H2B1C\_HUMAN, H2B1H\_HUMAN, H2B2E\_HUMAN, H2B1B\_HUMAN, H2B1J\_HUMAN, H2B1O\_HUMAN, H2B1A\_HUMAN |
| Sequence | ``` EIQTAVR ``` |
| Position | 94 |
| PTMs | Variant #1: none Variant #2: Deamidation+Q(3) |
| 507\* | Confidence | Green |
| Runs | 1, 2, 3, 4 |
| Relation | Discriminating |
| Proteins | H2B2E\_HUMAN, H2B1B\_HUMAN, H2B1J\_HUMAN, H2B1O\_HUMAN, H2B3B\_HUMAN, H2B2C\_HUMAN, H2B2D\_HUMAN |
| Sequence | ``` ESYSIYVYK ``` |
| Position | 36 |
| PTMs | none |
| 652\*\* | Confidence | Green |
| Runs | 1, 2, 3, 4 |
| Relation | NonDiscriminating |
| Proteins | H2B1N\_HUMAN, H2B1M\_HUMAN, H2B2F\_HUMAN, H2B1K\_HUMAN, H2BFS\_HUMAN, H2B1L\_HUMAN, H2B1D\_HUMAN, H2B1C\_HUMAN, H2B1H\_HUMAN, H2B2E\_HUMAN, H2B1B\_HUMAN, H2B1J\_HUMAN, H2B1O\_HUMAN, H2B3B\_HUMAN, H2B1A\_HUMAN |
| Sequence | ``` LLLPGELAK ``` |
| Position | 101 |
| PTMs | none |
| 1119\*\* | Confidence | Green |
| Runs | 1, 2, 3, 4 |
| Relation | NonDiscriminating |
| Proteins | H2B1N\_HUMAN, H2B1M\_HUMAN, H2B2F\_HUMAN, H2B1K\_HUMAN, H2BFS\_HUMAN, H2B1L\_HUMAN, H2B1D\_HUMAN, H2B1C\_HUMAN, H2B1H\_HUMAN, H2B2E\_HUMAN, H2B1B\_HUMAN, H2B1J\_HUMAN, H2B1O\_HUMAN, H2B3B\_HUMAN |
| Sequence | ``` AMGIMNSFVNDIFER ``` |
| Position | 59 |
| PTMs | none |

  

Protein Q8N257

| Name | H2B3B\_HUMAN | | |
| Description | Histone H2B type 3 B OS Homo sapiens GN HIST3H2BB PE 1 SV 3 | | |
| Sequence | ```   1 MPDPSKSAPA PKKGSKKAVT KAQKKDGKKR KRGRKESYSI YVYKVLKQVH PDTGISSKAM GIMNSFVNDI FERIASEASR LAHYNKRSTI TSREVQTAVR 101 LLLPGELAKH AVSEGTKAVT KYTSSK ``` | | |
| Evidence | Indistinguishable | | |
| Peptide list | 25\*\*, 507\*, 652\*\*, 1119\*\* | | |
| Peptides | 25\*\* | Confidence | Green |
| Runs | 1, 2, 3, 4 |
| Relation | NonDiscriminating |
| Proteins | H2B1N\_HUMAN, H2B1M\_HUMAN, H2B2F\_HUMAN, H2B1K\_HUMAN, H2BFS\_HUMAN, H2B1L\_HUMAN, H2B1D\_HUMAN, H2B1C\_HUMAN, H2B1H\_HUMAN, H2B2E\_HUMAN, H2B1B\_HUMAN, H2B1J\_HUMAN, H2B1O\_HUMAN, H2B3B\_HUMAN, H2B1A\_HUMAN |
| Sequence | ``` QVHPDTGISSK ``` |
| Position | 48 |
| PTMs | none |
| 507\* | Confidence | Green |
| Runs | 1, 2, 3, 4 |
| Relation | Discriminating |
| Proteins | H2B2E\_HUMAN, H2B1B\_HUMAN, H2B1J\_HUMAN, H2B1O\_HUMAN, H2B3B\_HUMAN, H2B2C\_HUMAN, H2B2D\_HUMAN |
| Sequence | ``` ESYSIYVYK ``` |
| Position | 36 |
| PTMs | none |
| 652\*\* | Confidence | Green |
| Runs | 1, 2, 3, 4 |
| Relation | NonDiscriminating |
| Proteins | H2B1N\_HUMAN, H2B1M\_HUMAN, H2B2F\_HUMAN, H2B1K\_HUMAN, H2BFS\_HUMAN, H2B1L\_HUMAN, H2B1D\_HUMAN, H2B1C\_HUMAN, H2B1H\_HUMAN, H2B2E\_HUMAN, H2B1B\_HUMAN, H2B1J\_HUMAN, H2B1O\_HUMAN, H2B3B\_HUMAN, H2B1A\_HUMAN |
| Sequence | ``` LLLPGELAK ``` |
| Position | 101 |
| PTMs | none |
| 1119\*\* | Confidence | Green |
| Runs | 1, 2, 3, 4 |
| Relation | NonDiscriminating |
| Proteins | H2B1N\_HUMAN, H2B1M\_HUMAN, H2B2F\_HUMAN, H2B1K\_HUMAN, H2BFS\_HUMAN, H2B1L\_HUMAN, H2B1D\_HUMAN, H2B1C\_HUMAN, H2B1H\_HUMAN, H2B2E\_HUMAN, H2B1B\_HUMAN, H2B1J\_HUMAN, H2B1O\_HUMAN, H2B3B\_HUMAN |
| Sequence | ``` AMGIMNSFVNDIFER ``` |
| Position | 59 |
| PTMs | none |

  

Protein Q6DN03

| Name | H2B2C\_HUMAN | | |
| Description | Putative histone H2B type 2 C OS Homo sapiens GN HIST2H2BC PE 5 SV 3 | | |
| Sequence | ```   1 MPEPAKFAPA PKKGSKKAVT KAQKKDGKKR KRSRKESYSI YVYKVLKRVH PDTGIWCKAM GIMNSFLNDI FERIAGEASR LAHYNKRSTI TSRRSRRPCA 101 CCCPASWPST PCPRAPRRSP STPAPSESLP GPGARSLPPS LPPRVAGCFV SKGSFQGHLT TSVKESFLCC QSQLMFLASR LVNFRRAHNT KHR ``` | | |
| Evidence | Indistinguishable | | |
| Peptide list | 507\* | | |
| Peptides | 507\* | Confidence | Green |
| Runs | 1, 2, 3, 4 |
| Relation | Discriminating |
| Proteins | H2B2E\_HUMAN, H2B1B\_HUMAN, H2B1J\_HUMAN, H2B1O\_HUMAN, H2B3B\_HUMAN, H2B2C\_HUMAN, H2B2D\_HUMAN |
| Sequence | ``` ESYSIYVYK ``` |
| Position | 36 |
| PTMs | none |

  

Protein Q6DRA6

| Name | H2B2D\_HUMAN | | |
| Description | Putative histone H2B type 2 D OS Homo sapiens GN HIST2H2BD PE 5 SV 3 | | |
| Sequence | ```   1 MPEPAKFAPA PKKGSKKAVT KAQKKDGKKR KRSRKESYSI YVYKVLKRVH PDTGIWCKAM GIMNSFLNDI FERIAGEASR LAHYNKRSTI TSRRSRRPCA 101 CCCPASWPST PCPRAPRRSP STPAPSESLP GPGARSLPPS LPPRVAGCFV SKGSFQGHLT PLVK ``` | | |
| Evidence | Indistinguishable | | |
| Peptide list | 507\* | | |
| Peptides | 507\* | Confidence | Green |
| Runs | 1, 2, 3, 4 |
| Relation | Discriminating |
| Proteins | H2B2E\_HUMAN, H2B1B\_HUMAN, H2B1J\_HUMAN, H2B1O\_HUMAN, H2B3B\_HUMAN, H2B2C\_HUMAN, H2B2D\_HUMAN |
| Sequence | ``` ESYSIYVYK ``` |
| Position | 36 |
| PTMs | none |

  

Protein P07900

| Name | HS90A\_HUMAN | | |
| Description | Heat shock protein HSP 90 alpha OS Homo sapiens GN HSP90AA1 PE 1 SV 5 | | |
| Sequence | ```   1 MPEETQTQDQ PMEEEEVETF AFQAEIAQLM SLIINTFYSN KEIFLRELIS NSSDALDKIR YESLTDPSKL DSGKELHINL IPNKQDRTLT IVDTGIGMTK 101 ADLINNLGTI AKSGTKAFME ALQAGADISM IGQFGVGFYS AYLVAEKVTV ITKHNDDEQY AWESSAGGSF TVRTDTGEPM GRGTKVILHL KEDQTEYLEE 201 RRIKEIVKKH SQFIGYPITL FVEKERDKEV SDDEAEEKED KEEEKEKEEK ESEDKPEIED VGSDEEEEKK DGDKKKKKKI KEKYIDQEEL NKTKPIWTRN 301 PDDITNEEYG EFYKSLTNDW EDHLAVKHFS VEGQLEFRAL LFVPRRAPFD LFENRKKKNN IKLYVRRVFI MDNCEELIPE YLNFIRGVVD SEDLPLNISR 401 EMLQQSKILK VIRKNLVKKC LELFTELAED KENYKKFYEQ FSKNIKLGIH EDSQNRKKLS ELLRYYTSAS GDEMVSLKDY CTRMKENQKH IYYITGETKD 501 QVANSAFVER LRKHGLEVIY MIEPIDEYCV QQLKEFEGKT LVSVTKEGLE LPEDEEEKKK QEEKKTKFEN LCKIMKDILE KKVEKVVVSN RLVTSPCCIV 601 TSTYGWTANM ERIMKAQALR DNSTMGYMAA KKHLEINPDH SIIETLRQKA EADKNDKSVK DLVILLYETA LLSSGFSLED PQTHANRIYR MIKLGLGIDE 701 DDPTADDTSA AVTEEMPPLE GDDDTSRMEE VD ``` | | |
| Evidence | Indistinguishable | | |
| Peptide list | 38\*, 146\*, 147\*\*, 183\*\*, 236\*\*, 243\*\*, 255\*\*, 264\*, 598\*\*, 619\*\*, 638\*\*, 651\*, 679\*, 689\*\*, 784\*, 986\*\*, 1302\*\*, 1557\*, 1666\* | | |
| Peptides | 38\* | Confidence | Green |
| Runs | 1, 2, 3, 4 |
| Relation | Discriminating |
| Proteins | HS90A\_HUMAN, HS90A\_HUMAN |
| Sequence | ``` LGIHEDSQNR ``` |
| Position | 447 |
| PTMs | none |
| 146\* | Confidence | Green |
| Runs | 1, 2, 3, 4 |
| Relation | Discriminating |
| Proteins | HS90A\_HUMAN, HS90A\_HUMAN |
| Sequence | ``` TLVSVTK ``` |
| Position | 540 |
| PTMs | none |
| 147\*\* | Confidence | Green |
| Runs | 1, 2, 3, 4 |
| Relation | NonDiscriminating |
| Proteins | HS90B\_HUMAN, HS90A\_HUMAN, HS90A\_HUMAN, H90B3\_HUMAN, H90B2\_HUMAN, H90B4\_HUMAN, HS902\_HUMAN |
| Sequence | ``` YESLTDPSK ``` |
| Position | 61 |
| PTMs | none |
| 183\*\* | Confidence | Green |
| Runs | 1, 2, 4 |
| Relation | NonDiscriminating |
| Proteins | HS90B\_HUMAN, HS90A\_HUMAN, HS90A\_HUMAN, H90B2\_HUMAN, HS902\_HUMAN |
| Sequence | ``` YIDQEELNK ``` |
| Position | 284 |
| PTMs | none |
| 236\*\* | Confidence | Green |
| Runs | 1, 2, 3, 4 |
| Relation | NonDiscriminating |
| Proteins | HS90B\_HUMAN, HS90A\_HUMAN, HS90A\_HUMAN, H90B3\_HUMAN, H90B4\_HUMAN, HS902\_HUMAN |
| Sequence | ``` VILHLK ``` |
| Position | 186 |
| PTMs | none |
| 243\*\* | Confidence | Green |
| Runs | 1, 2, 3, 4 |
| Relation | NonDiscriminating |
| Proteins | HS90B\_HUMAN, HS90A\_HUMAN, HS90A\_HUMAN, H90B3\_HUMAN, H90B4\_HUMAN |
| Sequence | ``` EDQTEYLEER ``` |
| Position | 192 |
| PTMs | none |
| 255\*\* | Confidence | Green |
| Runs | 1, 2, 3, 4 |
| Relation | NonDiscriminating |
| Proteins | HS90A\_HUMAN, HS90A\_HUMAN, HS905\_HUMAN |
| Sequence | ``` HIYYITGETK ``` |
| Position | 490 |
| PTMs | none |
| 264\* | Confidence | Green |
| Runs | 1, 3, 4 |
| Relation | Discriminating |
| Proteins | HS90A\_HUMAN, HS90A\_HUMAN |
| Sequence | ``` DQVANSAFVER ``` |
| Position | 500 |
| PTMs | none |
| 598\*\* | Confidence | Green |
| Runs | 1, 2, 3, 4 |
| Relation | NonDiscriminating |
| Proteins | HS90A\_HUMAN, HS90A\_HUMAN, HS902\_HUMAN |
| Sequence | ``` ELHINLIPNK ``` |
| Position | 75 |
| PTMs | none |
| 619\*\* | Confidence | Green |
| Runs | 1, 3, 4 |
| Relation | NonDiscriminating |
| Proteins | HS90B\_HUMAN, HS90A\_HUMAN, HS90A\_HUMAN |
| Sequence | ``` SLTNDWEDHLAVK ``` |
| Position | 315 |
| PTMs | Variant #1: none Variant #2: Deamidation+N(4) |
| 638\*\* | Confidence | Green |
| Runs | 1, 2, 3, 4 |
| Relation | NonDiscriminating |
| Proteins | HS90B\_HUMAN, HS90A\_HUMAN, HS90A\_HUMAN, H90B2\_HUMAN, HS902\_HUMAN |
| Sequence | ``` ADLINNLGTIAK ``` |
| Position | 101 |
| PTMs | Variant #1: Deamidation+N(5)  Variant #2: none |
| 651\* | Confidence | Green |
| Runs | 1, 2, 3, 4 |
| Relation | Discriminating |
| Proteins | HS90A\_HUMAN, HS90A\_HUMAN |
| Sequence | ``` HLEINPDHSIIETLR ``` |
| Position | 633 |
| PTMs | none |
| 679\* | Confidence | Green |
| Runs | 1, 2, 3, 4 |
| Relation | Discriminating |
| Proteins | HS90A\_HUMAN, HS90A\_HUMAN |
| Sequence | ``` ALLFVPR ``` |
| Position | 339 |
| PTMs | none |
| 689\*\* | Confidence | Green |
| Runs | 1, 2, 3, 4 |
| Relation | NonDiscriminating |
| Proteins | HS90B\_HUMAN, HS90A\_HUMAN, HS90A\_HUMAN, H90B3\_HUMAN |
| Sequence | ``` GVVDSEDLPLNISR ``` |
| Position | 387 |
| PTMs | none |
| 784\* | Confidence | Green |
| Runs | 1, 2, 4 |
| Relation | Discriminating |
| Proteins | HS90A\_HUMAN, HS90A\_HUMAN |
| Sequence | ``` APFDLFENR ``` |
| Position | 347 |
| PTMs | none |
| 986\*\* | Confidence | Green |
| Runs | 1, 2, 3, 4 |
| Relation | NonDiscriminating |
| Proteins | HS90A\_HUMAN, HS90A\_HUMAN, HS905\_HUMAN |
| Sequence | ``` HSQFIGYPITLFVEK ``` |
| Position | 210 |
| PTMs | none |
| 1302\*\* | Confidence | Green |
| Runs | 2, 3, 4 |
| Relation | NonDiscriminating |
| Proteins | HS90B\_HUMAN, HS90A\_HUMAN, HS90A\_HUMAN, GBF1\_HUMAN |
| Sequence | ``` LSELLR ``` |
| Position | 459 |
| PTMs | none |
| 1557\* | Confidence | Green |
| Runs | 2, 3, 4 |
| Relation | Discriminating |
| Proteins | HS90A\_HUMAN, HS90A\_HUMAN |
| Sequence | ``` CLELFTELAEDKENYK ``` |
| Position | 420 |
| PTMs | Carbamidomethyl+C(1) |
| 1666\* | Confidence | Green |
| Runs | 2, 3, 4 |
| Relation | Discriminating |
| Proteins | HS90A\_HUMAN, HS90A\_HUMAN |
| Sequence | ``` VFIMDNCEELIPEYLNFIR ``` |
| Position | 368 |
| PTMs | Variant #1: Carbamidomethyl+C(7) Oxidation+M(4)  Variant #2: Carbamidomethyl+C(7) |

  

Protein P07900-2

| Name | HS90A\_HUMAN | | |
| Description | Isoform 2 of Heat shock protein HSP 90 alpha OS Homo sapiens GN HSP90AA1 | | |
| Sequence | ```   1 MPPCSGGDGS TPPGPSLRDR DCPAQSAEYP RDRLDPRPGS PSEASSPPFL RSRAPVNWYQ EKAQVFLWHL MVSGSTTLLC LWKQPFHVSA FPVTASLAFR 101 QSQGAGQHLY KDLQPFILLR LLMPEETQTQ DQPMEEEEVE TFAFQAEIAQ LMSLIINTFY SNKEIFLREL ISNSSDALDK IRYESLTDPS KLDSGKELHI 201 NLIPNKQDRT LTIVDTGIGM TKADLINNLG TIAKSGTKAF MEALQAGADI SMIGQFGVGF YSAYLVAEKV TVITKHNDDE QYAWESSAGG SFTVRTDTGE 301 PMGRGTKVIL HLKEDQTEYL EERRIKEIVK KHSQFIGYPI TLFVEKERDK EVSDDEAEEK EDKEEEKEKE EKESEDKPEI EDVGSDEEEE KKDGDKKKKK 401 KIKEKYIDQE ELNKTKPIWT RNPDDITNEE YGEFYKSLTN DWEDHLAVKH FSVEGQLEFR ALLFVPRRAP FDLFENRKKK NNIKLYVRRV FIMDNCEELI 501 PEYLNFIRGV VDSEDLPLNI SREMLQQSKI LKVIRKNLVK KCLELFTELA EDKENYKKFY EQFSKNIKLG IHEDSQNRKK LSELLRYYTS ASGDEMVSLK 601 DYCTRMKENQ KHIYYITGET KDQVANSAFV ERLRKHGLEV IYMIEPIDEY CVQQLKEFEG KTLVSVTKEG LELPEDEEEK KKQEEKKTKF ENLCKIMKDI 701 LEKKVEKVVV SNRLVTSPCC IVTSTYGWTA NMERIMKAQA LRDNSTMGYM AAKKHLEINP DHSIIETLRQ KAEADKNDKS VKDLVILLYE TALLSSGFSL 801 EDPQTHANRI YRMIKLGLGI DEDDPTADDT SAAVTEEMPP LEGDDDTSRM EEVD ``` | | |
| Evidence | Indistinguishable | | |
| Peptide list | 38\*, 146\*, 147\*\*, 183\*\*, 236\*\*, 243\*\*, 255\*\*, 264\*, 598\*\*, 619\*\*, 638\*\*, 651\*, 679\*, 689\*\*, 784\*, 986\*\*, 1302\*\*, 1557\*, 1666\* | | |
| Peptides | 38\* | Confidence | Green |
| Runs | 1, 2, 3, 4 |
| Relation | Discriminating |
| Proteins | HS90A\_HUMAN, HS90A\_HUMAN |
| Sequence | ``` LGIHEDSQNR ``` |
| Position | 569 |
| PTMs | none |
| 146\* | Confidence | Green |
| Runs | 1, 2, 3, 4 |
| Relation | Discriminating |
| Proteins | HS90A\_HUMAN, HS90A\_HUMAN |
| Sequence | ``` TLVSVTK ``` |
| Position | 662 |
| PTMs | none |
| 147\*\* | Confidence | Green |
| Runs | 1, 2, 3, 4 |
| Relation | NonDiscriminating |
| Proteins | HS90B\_HUMAN, HS90A\_HUMAN, HS90A\_HUMAN, H90B3\_HUMAN, H90B2\_HUMAN, H90B4\_HUMAN, HS902\_HUMAN |
| Sequence | ``` YESLTDPSK ``` |
| Position | 183 |
| PTMs | none |
| 183\*\* | Confidence | Green |
| Runs | 1, 2, 4 |
| Relation | NonDiscriminating |
| Proteins | HS90B\_HUMAN, HS90A\_HUMAN, HS90A\_HUMAN, H90B2\_HUMAN, HS902\_HUMAN |
| Sequence | ``` YIDQEELNK ``` |
| Position | 406 |
| PTMs | none |
| 236\*\* | Confidence | Green |
| Runs | 1, 2, 3, 4 |
| Relation | NonDiscriminating |
| Proteins | HS90B\_HUMAN, HS90A\_HUMAN, HS90A\_HUMAN, H90B3\_HUMAN, H90B4\_HUMAN, HS902\_HUMAN |
| Sequence | ``` VILHLK ``` |
| Position | 308 |
| PTMs | none |
| 243\*\* | Confidence | Green |
| Runs | 1, 2, 3, 4 |
| Relation | NonDiscriminating |
| Proteins | HS90B\_HUMAN, HS90A\_HUMAN, HS90A\_HUMAN, H90B3\_HUMAN, H90B4\_HUMAN |
| Sequence | ``` EDQTEYLEER ``` |
| Position | 314 |
| PTMs | none |
| 255\*\* | Confidence | Green |
| Runs | 1, 2, 3, 4 |
| Relation | NonDiscriminating |
| Proteins | HS90A\_HUMAN, HS90A\_HUMAN, HS905\_HUMAN |
| Sequence | ``` HIYYITGETK ``` |
| Position | 612 |
| PTMs | none |
| 264\* | Confidence | Green |
| Runs | 1, 3, 4 |
| Relation | Discriminating |
| Proteins | HS90A\_HUMAN, HS90A\_HUMAN |
| Sequence | ``` DQVANSAFVER ``` |
| Position | 622 |
| PTMs | none |
| 598\*\* | Confidence | Green |
| Runs | 1, 2, 3, 4 |
| Relation | NonDiscriminating |
| Proteins | HS90A\_HUMAN, HS90A\_HUMAN, HS902\_HUMAN |
| Sequence | ``` ELHINLIPNK ``` |
| Position | 197 |
| PTMs | none |
| 619\*\* | Confidence | Green |
| Runs | 1, 3, 4 |
| Relation | NonDiscriminating |
| Proteins | HS90B\_HUMAN, HS90A\_HUMAN, HS90A\_HUMAN |
| Sequence | ``` SLTNDWEDHLAVK ``` |
| Position | 437 |
| PTMs | Variant #1: none Variant #2: Deamidation+N(4) |
| 638\*\* | Confidence | Green |
| Runs | 1, 2, 3, 4 |
| Relation | NonDiscriminating |
| Proteins | HS90B\_HUMAN, HS90A\_HUMAN, HS90A\_HUMAN, H90B2\_HUMAN, HS902\_HUMAN |
| Sequence | ``` ADLINNLGTIAK ``` |
| Position | 223 |
| PTMs | Variant #1: Deamidation+N(5)  Variant #2: none |
| 651\* | Confidence | Green |
| Runs | 1, 2, 3, 4 |
| Relation | Discriminating |
| Proteins | HS90A\_HUMAN, HS90A\_HUMAN |
| Sequence | ``` HLEINPDHSIIETLR ``` |
| Position | 755 |
| PTMs | none |
| 679\* | Confidence | Green |
| Runs | 1, 2, 3, 4 |
| Relation | Discriminating |
| Proteins | HS90A\_HUMAN, HS90A\_HUMAN |
| Sequence | ``` ALLFVPR ``` |
| Position | 461 |
| PTMs | none |
| 689\*\* | Confidence | Green |
| Runs | 1, 2, 3, 4 |
| Relation | NonDiscriminating |
| Proteins | HS90B\_HUMAN, HS90A\_HUMAN, HS90A\_HUMAN, H90B3\_HUMAN |
| Sequence | ``` GVVDSEDLPLNISR ``` |
| Position | 509 |
| PTMs | none |
| 784\* | Confidence | Green |
| Runs | 1, 2, 4 |
| Relation | Discriminating |
| Proteins | HS90A\_HUMAN, HS90A\_HUMAN |
| Sequence | ``` APFDLFENR ``` |
| Position | 469 |
| PTMs | none |
| 986\*\* | Confidence | Green |
| Runs | 1, 2, 3, 4 |
| Relation | NonDiscriminating |
| Proteins | HS90A\_HUMAN, HS90A\_HUMAN, HS905\_HUMAN |
| Sequence | ``` HSQFIGYPITLFVEK ``` |
| Position | 332 |
| PTMs | none |
| 1302\*\* | Confidence | Green |
| Runs | 2, 3, 4 |
| Relation | NonDiscriminating |
| Proteins | HS90B\_HUMAN, HS90A\_HUMAN, HS90A\_HUMAN, GBF1\_HUMAN |
| Sequence | ``` LSELLR ``` |
| Position | 581 |
| PTMs | none |
| 1557\* | Confidence | Green |
| Runs | 2, 3, 4 |
| Relation | Discriminating |
| Proteins | HS90A\_HUMAN, HS90A\_HUMAN |
| Sequence | ``` CLELFTELAEDKENYK ``` |
| Position | 542 |
| PTMs | Carbamidomethyl+C(1) |
| 1666\* | Confidence | Green |
| Runs | 2, 3, 4 |
| Relation | Discriminating |
| Proteins | HS90A\_HUMAN, HS90A\_HUMAN |
| Sequence | ``` VFIMDNCEELIPEYLNFIR ``` |
| Position | 490 |
| PTMs | Variant #1: Carbamidomethyl+C(7) Oxidation+M(4)  Variant #2: Carbamidomethyl+C(7) |

  

Protein Q3ZCM7

| Name | TBB8\_HUMAN | | |
| Description | Tubulin beta 8 chain OS Homo sapiens GN TUBB8 PE 1 SV 2 | | |
| Sequence | ```   1 MREIVLTQIG QCGNQIGAKF WEVISDEHAI DSAGTYHGDS HLQLERINVY YNEASGGRYV PRAVLVDLEP GTMDSVRSGP FGQVFRPDNF IFGQCGAGNN 101 WAKGHYTEGA ELMESVMDVV RKEAESCDCL QGFQLTHSLG GGTGSGMGTL LLSKIREEYP DRIINTFSIL PSPKVSDTVV EPYNATLSVH QLIENADETF 201 CIDNEALYDI CSKTLKLPTP TYGDLNHLVS ATMSGVTTCL RFPGQLNADL RKLAVNMVPF PRLHFFMPGF APLTSRGSQQ YRALTVAELT QQMFDAKNMM 301 AACDPRHGRY LTAAAIFRGR MPMREVDEQM FNIQDKNSSY FADWLPNNVK TAVCDIPPRG LKMSATFIGN NTAIQELFKR VSEQFTAMFR RKAFLHWYTG 401 EGMDEMEFTE AESNMNDLVS EYQQYQDATA EEEEDEEYAE EEVA ``` | | |
| Evidence | Indistinguishable | | |
| Peptide list | 42\*\*, 877\*, 995\*\* | | |
| Peptides | 42\*\* | Confidence | Green |
| Runs | 1, 2, 3, 4 |
| Relation | NonDiscriminating |
| Proteins | TBB5\_HUMAN, TBB4B\_HUMAN, TBB2A\_HUMAN, TBB2B\_HUMAN, TBB8\_HUMAN, YI016\_HUMAN, TBB8B\_HUMAN, TBB1\_HUMAN |
| Sequence | ``` IREEYPDR ``` |
| Position | 155 |
| PTMs | none |
| 877\* | Confidence | Green |
| Runs | 1, 2, 3 |
| Relation | Discriminating |
| Proteins | TBB8\_HUMAN, YI016\_HUMAN, TBB8B\_HUMAN |
| Sequence | ``` ALTVAELTQQMFDAK ``` |
| Position | 283 |
| PTMs | Variant #1: none Variant #2: Deamidation+Q(9) |
| 995\*\* | Confidence | Green |
| Runs | 1, 2, 3, 4 |
| Relation | NonDiscriminating |
| Proteins | TBB5\_HUMAN, TBB4B\_HUMAN, TBB2A\_HUMAN, TBB2B\_HUMAN, TBB4A\_HUMAN, TBB8\_HUMAN, YI016\_HUMAN, TBB8B\_HUMAN, TBB6\_HUMAN |
| Sequence | ``` LHFFMPGFAPLTSR ``` |
| Position | 263 |
| PTMs | none |

  

Protein A6NKZ8

| Name | YI016\_HUMAN | | |
| Description | Putative tubulin beta chain like protein ENSP00000290377 OS Homo sapiens PE 5 SV 2 | | |
| Sequence | ```   1 MDSVRSGPFG QVLRPDNFIF GQCGAGNNWA KGRYTEGAEL TESVMDVVRK EAESCDCLQG FQLTHSLGGG TGSGMVTLLI SKIREEYPDR IINTFSILPS 101 PKVSDTVVEP YNATLSVHQL IENADETFCI DNEALYDICS KTLKLPTPTY GDLNHLVSAT MSGVTTCLRF PGQLNADLRK LAVNMVPFPR LHFFMPGFAP 201 LTSRGSQQYR ALTVAELTQQ MFDAKNMRAA RDPRHGRYLT AAAIFQGRMP MREVDEQMFN IQDKNSSYFA DWFPDNVKTA ICDIPPRGLK MSASFIGNNA 301 AIQELFTCVS EQFTAMFRRK AFLHWYTGEG MDEMEFTEAE SNMNDLVSEY QQYQDATAKE EEDEEYAEEE VA ``` | | |
| Evidence | Indistinguishable | | |
| Peptide list | 42\*\*, 877\*, 995\*\* | | |
| Peptides | 42\*\* | Confidence | Green |
| Runs | 1, 2, 3, 4 |
| Relation | NonDiscriminating |
| Proteins | TBB5\_HUMAN, TBB4B\_HUMAN, TBB2A\_HUMAN, TBB2B\_HUMAN, TBB8\_HUMAN, YI016\_HUMAN, TBB8B\_HUMAN, TBB1\_HUMAN |
| Sequence | ``` IREEYPDR ``` |
| Position | 83 |
| PTMs | none |
| 877\* | Confidence | Green |
| Runs | 1, 2, 3 |
| Relation | Discriminating |
| Proteins | TBB8\_HUMAN, YI016\_HUMAN, TBB8B\_HUMAN |
| Sequence | ``` ALTVAELTQQMFDAK ``` |
| Position | 211 |
| PTMs | Variant #1: none Variant #2: Deamidation+Q(9) |
| 995\*\* | Confidence | Green |
| Runs | 1, 2, 3, 4 |
| Relation | NonDiscriminating |
| Proteins | TBB5\_HUMAN, TBB4B\_HUMAN, TBB2A\_HUMAN, TBB2B\_HUMAN, TBB4A\_HUMAN, TBB8\_HUMAN, YI016\_HUMAN, TBB8B\_HUMAN, TBB6\_HUMAN |
| Sequence | ``` LHFFMPGFAPLTSR ``` |
| Position | 191 |
| PTMs | none |

  

Protein A6NNZ2

| Name | TBB8B\_HUMAN | | |
| Description | Tubulin beta 8 chain B OS Homo sapiens PE 1 SV 1 | | |
| Sequence | ```   1 MREIVLTQTG QCGNQIGAKF WEVISDEHAI DSAGTYHGDS HLQLERINVH HHEASGGRYV PRAVLVDLEP GTMDSVHSGP FGQVFRPDNF ISGQCGAGNN 101 WAKGRYTEGA ELTESVMDVV RKEAESCDCL QGFQLTHSLG GGTGSGMGTL LISKIREEYP DRIINTFSIL PSPKVSDTVV EPYNATLSVH QLIENADETF 201 CIDNEALYDI CSRTLKLPTP TYGDLNHLVS ATMSGVTTCL RFPGQLNADL RKLAVNMVPF PRLHFFMPGF APLTSRGSQQ YRALTVAELT QQMFDAKNMM 301 AACDPRHGCY LTVAAIFRGR MPMREVDEQM FNIQDKNSSY FADWFPDNVK TAVCDIPPRG LKMSATFIGN NAAIQELFTC VSEQFTAMFR RKAFLHWYTG 401 EGMDEMEFTE AESNMNDLVS EYQQYQDATA EEEEDEEYAE EEVA ``` | | |
| Evidence | Indistinguishable | | |
| Peptide list | 42\*\*, 877\*, 995\*\* | | |
| Peptides | 42\*\* | Confidence | Green |
| Runs | 1, 2, 3, 4 |
| Relation | NonDiscriminating |
| Proteins | TBB5\_HUMAN, TBB4B\_HUMAN, TBB2A\_HUMAN, TBB2B\_HUMAN, TBB8\_HUMAN, YI016\_HUMAN, TBB8B\_HUMAN, TBB1\_HUMAN |
| Sequence | ``` IREEYPDR ``` |
| Position | 155 |
| PTMs | none |
| 877\* | Confidence | Green |
| Runs | 1, 2, 3 |
| Relation | Discriminating |
| Proteins | TBB8\_HUMAN, YI016\_HUMAN, TBB8B\_HUMAN |
| Sequence | ``` ALTVAELTQQMFDAK ``` |
| Position | 283 |
| PTMs | Variant #1: none Variant #2: Deamidation+Q(9) |
| 995\*\* | Confidence | Green |
| Runs | 1, 2, 3, 4 |
| Relation | NonDiscriminating |
| Proteins | TBB5\_HUMAN, TBB4B\_HUMAN, TBB2A\_HUMAN, TBB2B\_HUMAN, TBB4A\_HUMAN, TBB8\_HUMAN, YI016\_HUMAN, TBB8B\_HUMAN, TBB6\_HUMAN |
| Sequence | ``` LHFFMPGFAPLTSR ``` |
| Position | 263 |
| PTMs | none |

  

Protein P60174

| Name | TPIS\_HUMAN | | |
| Description | Triosephosphate isomerase OS Homo sapiens GN TPI1 PE 1 SV 3 | | |
| Sequence | ```   1 MAEDGEEAEF HFAALYISGQ WPRLRADTDL QRLGSSAMAP SRKFFVGGNW KMNGRKQSLG ELIGTLNAAK VPADTEVVCA PPTAYIDFAR QKLDPKIAVA 101 AQNCYKVTNG AFTGEISPGM IKDCGATWVV LGHSERRHVF GESDELIGQK VAHALAEGLG VIACIGEKLD EREAGITEKV VFEQTKVIAD NVKDWSKVVL 201 AYEPVWAIGT GKTATPQQAQ EVHEKLRGWL KSNVSDAVAQ STRIIYGGSV TGATCKELAS QPDVDGFLVG GASLKPEFVD IINAKQ ``` | | |
| Evidence | Indistinguishable | | |
| Peptide list | 17\*, 119\*, 143\*, 150\*, 274\*, 421\*, 858\*, 882\*, 927\*, 1081\* | | |
| Peptides | 17\* | Confidence | Green |
| Runs | 1, 2, 3, 4 |
| Relation | Discriminating |
| Proteins | TPIS\_HUMAN, TPIS\_HUMAN |
| Sequence | ``` TATPQQAQEVHEK ``` |
| Position | 213 |
| PTMs | Variant #1: none Variant #2: Deamidation+Q(8) |
| 119\* | Confidence | Green |
| Runs | 1, 2, 3 |
| Relation | Discriminating |
| Proteins | TPIS\_HUMAN, TPIS\_HUMAN |
| Sequence | ``` VVFEQTK ``` |
| Position | 180 |
| PTMs | none |
| 143\* | Confidence | Green |
| Runs | 1, 2, 3, 4 |
| Relation | Discriminating |
| Proteins | TPIS\_HUMAN, TPIS\_HUMAN |
| Sequence | ``` SNVSDAVAQSTR ``` |
| Position | 232 |
| PTMs | none |
| 150\* | Confidence | Green |
| Runs | 1, 3, 4 |
| Relation | Discriminating |
| Proteins | TPIS\_HUMAN, TPIS\_HUMAN |
| Sequence | ``` IAVAAQNCYK ``` |
| Position | 97 |
| PTMs | Carbamidomethyl+C(8) |
| 274\* | Confidence | Green |
| Runs | 1, 2, 3, 4 |
| Relation | Discriminating |
| Proteins | TPIS\_HUMAN, TPIS\_HUMAN |
| Sequence | ``` IIYGGSVTGATCK ``` |
| Position | 244 |
| PTMs | Carbamidomethyl+C(12) |
| 421\* | Confidence | Green |
| Runs | 1, 2, 3, 4 |
| Relation | Discriminating |
| Proteins | TPIS\_HUMAN, TPIS\_HUMAN |
| Sequence | ``` HVFGESDELIGQK ``` |
| Position | 138 |
| PTMs | none |
| 858\* | Confidence | Green |
| Runs | 1, 2, 3, 4 |
| Relation | Discriminating |
| Proteins | TPIS\_HUMAN, TPIS\_HUMAN |
| Sequence | ``` VPADTEVVCAPPTAYIDFAR ``` |
| Position | 71 |
| PTMs | Carbamidomethyl+C(9) |
| 882\* | Confidence | Green |
| Runs | 1, 2, 3, 4 |
| Relation | Discriminating |
| Proteins | TPIS\_HUMAN, TPIS\_HUMAN |
| Sequence | ``` QSLGELIGTLNAAK ``` |
| Position | 57 |
| PTMs | none |
| 927\* | Confidence | Green |
| Runs | 1, 2, 3, 4 |
| Relation | Discriminating |
| Proteins | TPIS\_HUMAN, TPIS\_HUMAN |
| Sequence | ``` VVLAYEPVWAIGTGK ``` |
| Position | 198 |
| PTMs | none |
| 1081\* | Confidence | Green |
| Runs | 1, 2, 3, 4 |
| Relation | Discriminating |
| Proteins | TPIS\_HUMAN, TPIS\_HUMAN |
| Sequence | ``` ELASQPDVDGFLVGGASLKPEFVDIINAK ``` |
| Position | 257 |
| PTMs | none |

  

Protein P60174-1

| Name | TPIS\_HUMAN | | |
| Description | Isoform 2 of Triosephosphate isomerase OS Homo sapiens GN TPI1 | | |
| Sequence | ```   1 MAPSRKFFVG GNWKMNGRKQ SLGELIGTLN AAKVPADTEV VCAPPTAYID FARQKLDPKI AVAAQNCYKV TNGAFTGEIS PGMIKDCGAT WVVLGHSERR 101 HVFGESDELI GQKVAHALAE GLGVIACIGE KLDEREAGIT EKVVFEQTKV IADNVKDWSK VVLAYEPVWA IGTGKTATPQ QAQEVHEKLR GWLKSNVSDA 201 VAQSTRIIYG GSVTGATCKE LASQPDVDGF LVGGASLKPE FVDIINAKQ ``` | | |
| Evidence | Indistinguishable | | |
| Peptide list | 17\*, 119\*, 143\*, 150\*, 274\*, 421\*, 858\*, 882\*, 927\*, 1081\* | | |
| Peptides | 17\* | Confidence | Green |
| Runs | 1, 2, 3, 4 |
| Relation | Discriminating |
| Proteins | TPIS\_HUMAN, TPIS\_HUMAN |
| Sequence | ``` TATPQQAQEVHEK ``` |
| Position | 176 |
| PTMs | Variant #1: none Variant #2: Deamidation+Q(8) |
| 119\* | Confidence | Green |
| Runs | 1, 2, 3 |
| Relation | Discriminating |
| Proteins | TPIS\_HUMAN, TPIS\_HUMAN |
| Sequence | ``` VVFEQTK ``` |
| Position | 143 |
| PTMs | none |
| 143\* | Confidence | Green |
| Runs | 1, 2, 3, 4 |
| Relation | Discriminating |
| Proteins | TPIS\_HUMAN, TPIS\_HUMAN |
| Sequence | ``` SNVSDAVAQSTR ``` |
| Position | 195 |
| PTMs | none |
| 150\* | Confidence | Green |
| Runs | 1, 3, 4 |
| Relation | Discriminating |
| Proteins | TPIS\_HUMAN, TPIS\_HUMAN |
| Sequence | ``` IAVAAQNCYK ``` |
| Position | 60 |
| PTMs | Carbamidomethyl+C(8) |
| 274\* | Confidence | Green |
| Runs | 1, 2, 3, 4 |
| Relation | Discriminating |
| Proteins | TPIS\_HUMAN, TPIS\_HUMAN |
| Sequence | ``` IIYGGSVTGATCK ``` |
| Position | 207 |
| PTMs | Carbamidomethyl+C(12) |
| 421\* | Confidence | Green |
| Runs | 1, 2, 3, 4 |
| Relation | Discriminating |
| Proteins | TPIS\_HUMAN, TPIS\_HUMAN |
| Sequence | ``` HVFGESDELIGQK ``` |
| Position | 101 |
| PTMs | none |
| 858\* | Confidence | Green |
| Runs | 1, 2, 3, 4 |
| Relation | Discriminating |
| Proteins | TPIS\_HUMAN, TPIS\_HUMAN |
| Sequence | ``` VPADTEVVCAPPTAYIDFAR ``` |
| Position | 34 |
| PTMs | Carbamidomethyl+C(9) |
| 882\* | Confidence | Green |
| Runs | 1, 2, 3, 4 |
| Relation | Discriminating |
| Proteins | TPIS\_HUMAN, TPIS\_HUMAN |
| Sequence | ``` QSLGELIGTLNAAK ``` |
| Position | 20 |
| PTMs | none |
| 927\* | Confidence | Green |
| Runs | 1, 2, 3, 4 |
| Relation | Discriminating |
| Proteins | TPIS\_HUMAN, TPIS\_HUMAN |
| Sequence | ``` VVLAYEPVWAIGTGK ``` |
| Position | 161 |
| PTMs | none |
| 1081\* | Confidence | Green |
| Runs | 1, 2, 3, 4 |
| Relation | Discriminating |
| Proteins | TPIS\_HUMAN, TPIS\_HUMAN |
| Sequence | ``` ELASQPDVDGFLVGGASLKPEFVDIINAK ``` |
| Position | 220 |
| PTMs | none |

  

Protein Q16695

| Name | H31T\_HUMAN | | |
| Description | Histone H3 1t OS Homo sapiens GN HIST3H3 PE 1 SV 3 | | |
| Sequence | ```   1 MARTKQTARK STGGKAPRKQ LATKVARKSA PATGGVKKPH RYRPGTVALR EIRRYQKSTE LLIRKLPFQR LMREIAQDFK TDLRFQSSAV MALQEACESY 101 LVGLFEDTNL CVIHAKRVTI MPKDIQLARR IRGERA ``` | | |
| Evidence | Indistinguishable | | |
| Peptide list | 120\*\*, 171\*, 172\*\*, 388\*\* | | |
| Peptides | 120\*\* | Confidence | Green |
| Runs | 1, 3, 4 |
| Relation | NonDiscriminating |
| Proteins | H32\_HUMAN, H31T\_HUMAN, H33\_HUMAN, H31\_HUMAN, H3C\_HUMAN |
| Sequence | ``` RVTIMPK ``` |
| Position | 117 |
| PTMs | none |
| 171\* | Confidence | Green |
| Runs | 1, 2, 3, 4 |
| Relation | Discriminating |
| Proteins | H32\_HUMAN, H31T\_HUMAN, H33\_HUMAN, H31\_HUMAN |
| Sequence | ``` EIAQDFK ``` |
| Position | 74 |
| PTMs | none |
| 172\*\* | Confidence | Green |
| Runs | 1, 2, 3, 4 |
| Relation | NonDiscriminating |
| Proteins | H32\_HUMAN, H31T\_HUMAN, H33\_HUMAN, H31\_HUMAN, H3C\_HUMAN |
| Sequence | ``` YRPGTVALR ``` |
| Position | 42 |
| PTMs | none |
| 388\*\* | Confidence | Green |
| Runs | 1, 2, 3, 4 |
| Relation | NonDiscriminating |
| Proteins | H32\_HUMAN, H31T\_HUMAN, H33\_HUMAN, H31\_HUMAN, H3C\_HUMAN |
| Sequence | ``` STELLIR ``` |
| Position | 58 |
| PTMs | none |

  

Protein Q71DI3

| Name | H32\_HUMAN | | |
| Description | Histone H3 2 OS Homo sapiens GN HIST2H3A PE 1 SV 3 | | |
| Sequence | ```   1 MARTKQTARK STGGKAPRKQ LATKAARKSA PATGGVKKPH RYRPGTVALR EIRRYQKSTE LLIRKLPFQR LVREIAQDFK TDLRFQSSAV MALQEASEAY 101 LVGLFEDTNL CAIHAKRVTI MPKDIQLARR IRGERA ``` | | |
| Evidence | Indistinguishable | | |
| Peptide list | 120\*\*, 171\*, 172\*\*, 388\*\* | | |
| Peptides | 120\*\* | Confidence | Green |
| Runs | 1, 3, 4 |
| Relation | NonDiscriminating |
| Proteins | H32\_HUMAN, H31T\_HUMAN, H33\_HUMAN, H31\_HUMAN, H3C\_HUMAN |
| Sequence | ``` RVTIMPK ``` |
| Position | 117 |
| PTMs | none |
| 171\* | Confidence | Green |
| Runs | 1, 2, 3, 4 |
| Relation | Discriminating |
| Proteins | H32\_HUMAN, H31T\_HUMAN, H33\_HUMAN, H31\_HUMAN |
| Sequence | ``` EIAQDFK ``` |
| Position | 74 |
| PTMs | none |
| 172\*\* | Confidence | Green |
| Runs | 1, 2, 3, 4 |
| Relation | NonDiscriminating |
| Proteins | H32\_HUMAN, H31T\_HUMAN, H33\_HUMAN, H31\_HUMAN, H3C\_HUMAN |
| Sequence | ``` YRPGTVALR ``` |
| Position | 42 |
| PTMs | none |
| 388\*\* | Confidence | Green |
| Runs | 1, 2, 3, 4 |
| Relation | NonDiscriminating |
| Proteins | H32\_HUMAN, H31T\_HUMAN, H33\_HUMAN, H31\_HUMAN, H3C\_HUMAN |
| Sequence | ``` STELLIR ``` |
| Position | 58 |
| PTMs | none |

  

Protein P84243

| Name | H33\_HUMAN | | |
| Description | Histone H3 3 OS Homo sapiens GN H3F3A PE 1 SV 2 | | |
| Sequence | ```   1 MARTKQTARK STGGKAPRKQ LATKAARKSA PSTGGVKKPH RYRPGTVALR EIRRYQKSTE LLIRKLPFQR LVREIAQDFK TDLRFQSAAI GALQEASEAY 101 LVGLFEDTNL CAIHAKRVTI MPKDIQLARR IRGERA ``` | | |
| Evidence | Indistinguishable | | |
| Peptide list | 120\*\*, 171\*, 172\*\*, 388\*\* | | |
| Peptides | 120\*\* | Confidence | Green |
| Runs | 1, 3, 4 |
| Relation | NonDiscriminating |
| Proteins | H32\_HUMAN, H31T\_HUMAN, H33\_HUMAN, H31\_HUMAN, H3C\_HUMAN |
| Sequence | ``` RVTIMPK ``` |
| Position | 117 |
| PTMs | none |
| 171\* | Confidence | Green |
| Runs | 1, 2, 3, 4 |
| Relation | Discriminating |
| Proteins | H32\_HUMAN, H31T\_HUMAN, H33\_HUMAN, H31\_HUMAN |
| Sequence | ``` EIAQDFK ``` |
| Position | 74 |
| PTMs | none |
| 172\*\* | Confidence | Green |
| Runs | 1, 2, 3, 4 |
| Relation | NonDiscriminating |
| Proteins | H32\_HUMAN, H31T\_HUMAN, H33\_HUMAN, H31\_HUMAN, H3C\_HUMAN |
| Sequence | ``` YRPGTVALR ``` |
| Position | 42 |
| PTMs | none |
| 388\*\* | Confidence | Green |
| Runs | 1, 2, 3, 4 |
| Relation | NonDiscriminating |
| Proteins | H32\_HUMAN, H31T\_HUMAN, H33\_HUMAN, H31\_HUMAN, H3C\_HUMAN |
| Sequence | ``` STELLIR ``` |
| Position | 58 |
| PTMs | none |

  

Protein P68431

| Name | H31\_HUMAN | | |
| Description | Histone H3 1 OS Homo sapiens GN HIST1H3A PE 1 SV 2 | | |
| Sequence | ```   1 MARTKQTARK STGGKAPRKQ LATKAARKSA PATGGVKKPH RYRPGTVALR EIRRYQKSTE LLIRKLPFQR LVREIAQDFK TDLRFQSSAV MALQEACEAY 101 LVGLFEDTNL CAIHAKRVTI MPKDIQLARR IRGERA ``` | | |
| Evidence | Indistinguishable | | |
| Peptide list | 120\*\*, 171\*, 172\*\*, 388\*\* | | |
| Peptides | 120\*\* | Confidence | Green |
| Runs | 1, 3, 4 |
| Relation | NonDiscriminating |
| Proteins | H32\_HUMAN, H31T\_HUMAN, H33\_HUMAN, H31\_HUMAN, H3C\_HUMAN |
| Sequence | ``` RVTIMPK ``` |
| Position | 117 |
| PTMs | none |
| 171\* | Confidence | Green |
| Runs | 1, 2, 3, 4 |
| Relation | Discriminating |
| Proteins | H32\_HUMAN, H31T\_HUMAN, H33\_HUMAN, H31\_HUMAN |
| Sequence | ``` EIAQDFK ``` |
| Position | 74 |
| PTMs | none |
| 172\*\* | Confidence | Green |
| Runs | 1, 2, 3, 4 |
| Relation | NonDiscriminating |
| Proteins | H32\_HUMAN, H31T\_HUMAN, H33\_HUMAN, H31\_HUMAN, H3C\_HUMAN |
| Sequence | ``` YRPGTVALR ``` |
| Position | 42 |
| PTMs | none |
| 388\*\* | Confidence | Green |
| Runs | 1, 2, 3, 4 |
| Relation | NonDiscriminating |
| Proteins | H32\_HUMAN, H31T\_HUMAN, H33\_HUMAN, H31\_HUMAN, H3C\_HUMAN |
| Sequence | ``` STELLIR ``` |
| Position | 58 |
| PTMs | none |

  

Protein P00338

| Name | LDHA\_HUMAN | | |
| Description | L lactate dehydrogenase A chain OS Homo sapiens GN LDHA PE 1 SV 2 | | |
| Sequence | ```   1 MATLKDQLIY NLLKEEQTPQ NKITVVGVGA VGMACAISIL MKDLADELAL VDVIEDKLKG EMMDLQHGSL FLRTPKIVSG KDYNVTANSK LVIITAGARQ 101 QEGESRLNLV QRNVNIFKFI IPNVVKYSPN CKLLIVSNPV DILTYVAWKI SGFPKNRVIG SGCNLDSARF RYLMGERLGV HPLSCHGWVL GEHGDSSVPV 201 WSGMNVAGVS LKTLHPDLGT DKDKEQWKEV HKQVVESAYE VIKLKGYTSW AIGLSVADLA ESIMKNLRRV HPVSTMIKGL YGIKDDVFLS VPCILGQNGI 301 SDLVKVTLTS EEEARLKKSA DTLWGIQKEL QF ``` | | |
| Evidence | Indistinguishable | | |
| Peptide list | 135\*\*, 151\*\*, 180\*\*, 213\*\*, 425\*\*, 501\*, 576\*\*, 713\*\*, 890\*\* | | |
| Peptides | 135\*\* | Confidence | Green |
| Runs | 1, 2, 3, 4 |
| Relation | NonDiscriminating |
| Proteins | LDHA\_HUMAN, LDHA\_HUMAN, LDHA\_HUMAN |
| Sequence | ``` TLHPDLGTDK ``` |
| Position | 213 |
| PTMs | none |
| 151\*\* | Confidence | Green |
| Runs | 1, 2, 3, 4 |
| Relation | NonDiscriminating |
| Proteins | LDHA\_HUMAN, LDHA\_HUMAN, LDHA\_HUMAN |
| Sequence | ``` VTLTSEEEAR ``` |
| Position | 306 |
| PTMs | none |
| 180\*\* | Confidence | Green |
| Runs | 1, 2, 3, 4 |
| Relation | NonDiscriminating |
| Proteins | LDHB\_HUMAN, LDHA\_HUMAN, LDHA\_HUMAN, LDHA\_HUMAN, LDH6A\_HUMAN, LDHC\_HUMAN |
| Sequence | ``` VIGSGCNLDSAR ``` |
| Position | 158 |
| PTMs | Carbamidomethyl+C(6) |
| 213\*\* | Confidence | Green |
| Runs | 1, 2, 3 |
| Relation | NonDiscriminating |
| Proteins | LDHB\_HUMAN, LDHA\_HUMAN, LDHA\_HUMAN, LDHA\_HUMAN |
| Sequence | ``` LNLVQR ``` |
| Position | 107 |
| PTMs | none |
| 425\*\* | Confidence | Green |
| Runs | 1, 2, 3 |
| Relation | NonDiscriminating |
| Proteins | LDHA\_HUMAN, LDHA\_HUMAN, LDHA\_HUMAN, LDH6A\_HUMAN |
| Sequence | ``` LVIITAGAR ``` |
| Position | 91 |
| PTMs | none |
| 501\* | Confidence | Green |
| Runs | 1, 2, 3, 4 |
| Relation | Discriminating |
| Proteins | LDHA\_HUMAN, LDHA\_HUMAN |
| Sequence | ``` QVVESAYEVIK ``` |
| Position | 233 |
| PTMs | none |
| 576\*\* | Confidence | Green |
| Runs | 1, 2, 3, 4 |
| Relation | NonDiscriminating |
| Proteins | LDHA\_HUMAN, LDHA\_HUMAN, LDHA\_HUMAN |
| Sequence | ``` SADTLWGIQK ``` |
| Position | 319 |
| PTMs | none |
| 713\*\* | Confidence | Green |
| Runs | 1, 2, 3, 4 |
| Relation | NonDiscriminating |
| Proteins | LDHA\_HUMAN, LDHA\_HUMAN, LDHA\_HUMAN |
| Sequence | ``` FIIPNVVK ``` |
| Position | 119 |
| PTMs | none |
| 890\*\* | Confidence | Green |
| Runs | 1, 2, 3, 4 |
| Relation | NonDiscriminating |
| Proteins | LDHA\_HUMAN, LDHA\_HUMAN, LDHA\_HUMAN |
| Sequence | ``` DQLIYNLLK ``` |
| Position | 6 |
| PTMs | none |

  

Protein P00338-3

| Name | LDHA\_HUMAN | | |
| Description | Isoform 3 of L lactate dehydrogenase A chain OS Homo sapiens GN LDHA | | |
| Sequence | ```   1 MGEPSGGYTY TQTSIFLFHA KIPFGSKSNM ATLKDQLIYN LLKEEQTPQN KITVVGVGAV GMACAISILM KDLADELALV DVIEDKLKGE MMDLQHGSLF 101 LRTPKIVSGK DYNVTANSKL VIITAGARQQ EGESRLNLVQ RNVNIFKFII PNVVKYSPNC KLLIVSNPVD ILTYVAWKIS GFPKNRVIGS GCNLDSARFR 201 YLMGERLGVH PLSCHGWVLG EHGDSSVPVW SGMNVAGVSL KTLHPDLGTD KDKEQWKEVH KQVVESAYEV IKLKGYTSWA IGLSVADLAE SIMKNLRRVH 301 PVSTMIKGLY GIKDDVFLSV PCILGQNGIS DLVKVTLTSE EEARLKKSAD TLWGIQKELQ F ``` | | |
| Evidence | Indistinguishable | | |
| Peptide list | 135\*\*, 151\*\*, 180\*\*, 213\*\*, 425\*\*, 501\*, 576\*\*, 713\*\*, 890\*\* | | |
| Peptides | 135\*\* | Confidence | Green |
| Runs | 1, 2, 3, 4 |
| Relation | NonDiscriminating |
| Proteins | LDHA\_HUMAN, LDHA\_HUMAN, LDHA\_HUMAN |
| Sequence | ``` TLHPDLGTDK ``` |
| Position | 242 |
| PTMs | none |
| 151\*\* | Confidence | Green |
| Runs | 1, 2, 3, 4 |
| Relation | NonDiscriminating |
| Proteins | LDHA\_HUMAN, LDHA\_HUMAN, LDHA\_HUMAN |
| Sequence | ``` VTLTSEEEAR ``` |
| Position | 335 |
| PTMs | none |
| 180\*\* | Confidence | Green |
| Runs | 1, 2, 3, 4 |
| Relation | NonDiscriminating |
| Proteins | LDHB\_HUMAN, LDHA\_HUMAN, LDHA\_HUMAN, LDHA\_HUMAN, LDH6A\_HUMAN, LDHC\_HUMAN |
| Sequence | ``` VIGSGCNLDSAR ``` |
| Position | 187 |
| PTMs | Carbamidomethyl+C(6) |
| 213\*\* | Confidence | Green |
| Runs | 1, 2, 3 |
| Relation | NonDiscriminating |
| Proteins | LDHB\_HUMAN, LDHA\_HUMAN, LDHA\_HUMAN, LDHA\_HUMAN |
| Sequence | ``` LNLVQR ``` |
| Position | 136 |
| PTMs | none |
| 425\*\* | Confidence | Green |
| Runs | 1, 2, 3 |
| Relation | NonDiscriminating |
| Proteins | LDHA\_HUMAN, LDHA\_HUMAN, LDHA\_HUMAN, LDH6A\_HUMAN |
| Sequence | ``` LVIITAGAR ``` |
| Position | 120 |
| PTMs | none |
| 501\* | Confidence | Green |
| Runs | 1, 2, 3, 4 |
| Relation | Discriminating |
| Proteins | LDHA\_HUMAN, LDHA\_HUMAN |
| Sequence | ``` QVVESAYEVIK ``` |
| Position | 262 |
| PTMs | none |
| 576\*\* | Confidence | Green |
| Runs | 1, 2, 3, 4 |
| Relation | NonDiscriminating |
| Proteins | LDHA\_HUMAN, LDHA\_HUMAN, LDHA\_HUMAN |
| Sequence | ``` SADTLWGIQK ``` |
| Position | 348 |
| PTMs | none |
| 713\*\* | Confidence | Green |
| Runs | 1, 2, 3, 4 |
| Relation | NonDiscriminating |
| Proteins | LDHA\_HUMAN, LDHA\_HUMAN, LDHA\_HUMAN |
| Sequence | ``` FIIPNVVK ``` |
| Position | 148 |
| PTMs | none |
| 890\*\* | Confidence | Green |
| Runs | 1, 2, 3, 4 |
| Relation | NonDiscriminating |
| Proteins | LDHA\_HUMAN, LDHA\_HUMAN, LDHA\_HUMAN |
| Sequence | ``` DQLIYNLLK ``` |
| Position | 35 |
| PTMs | none |

  

Protein P22392-2

| Name | NDKB\_HUMAN | | |
| Description | Isoform 3 of Nucleoside diphosphate kinase B OS Homo sapiens GN NME2 | | |
| Sequence | ```   1 MANCERTFIA IKPDGVQRGL VGEIIKRFEQ KGFRLVGLKF MQASEDLLKE HYVDLKDRPF FAGLVKYMHS GPVVAMVWEG LNVVKTGRVM LGETNPADSK 101 PGTIRGDFCI QVGRTMANLE RTFIAIKPDG VQRGLVGEII KRFEQKGFRL VAMKFLRASE EHLKQHYIDL KDRPFFPGLV KYMNSGPVVA MVWEGLNVVK 201 TGRVMLGETN PADSKPGTIR GDFCIQVGRN IIHGSDSVKS AEKEISLWFK PEELVDYKSC AHDWVYE ``` | | |
| Evidence | Indistinguishable | | |
| Peptide list | 63\*\*, 347\*\*, 480\*\*, 577\*\*, 579\*\*, 688\*\*, 1010\* | | |
| Peptides | 63\*\* | Confidence | Green |
| Runs | 1, 2, 3, 4 |
| Relation | NonDiscriminating |
| Proteins | NDKB\_HUMAN, NDKB\_HUMAN, NDK8\_HUMAN |
| Sequence | ``` NIIHGSDSVK ``` |
| Position | 230 |
| PTMs | none |
| 347\*\* | Confidence | Green |
| Runs | 1, 2, 3, 4 |
| Relation | NonDiscriminating |
| Proteins | NDKB\_HUMAN, NDKB\_HUMAN, NDKA\_HUMAN, NDKA\_HUMAN, NDK8\_HUMAN |
| Sequence | ``` VMLGETNPADSKPGTIR ``` |
| Position | 89, 204 |
| PTMs | none |
| 480\*\* | Confidence | Green |
| Runs | 1, 2, 3, 4 |
| Relation | NonDiscriminating |
| Proteins | NDKB\_HUMAN, NDKB\_HUMAN, NDKA\_HUMAN, NDKA\_HUMAN, NDK8\_HUMAN |
| Sequence | ``` GDFCIQVGR ``` |
| Position | 106, 221 |
| PTMs | Carbamidomethyl+C(4) |
| 577\*\* | Confidence | Green |
| Runs | 1, 2, 3, 4 |
| Relation | NonDiscriminating |
| Proteins | NDKB\_HUMAN, NDKB\_HUMAN, NDKA\_HUMAN, NDKA\_HUMAN |
| Sequence | ``` GLVGEIIK ``` |
| Position | 19, 134 |
| PTMs | none |
| 579\*\* | Confidence | Green |
| Runs | 1, 2, 3, 4 |
| Relation | NonDiscriminating |
| Proteins | NDKB\_HUMAN, NDKA\_HUMAN, NDKA\_HUMAN |
| Sequence | ``` FMQASEDLLK ``` |
| Position | 40 |
| PTMs | none |
| 688\*\* | Confidence | Green |
| Runs | 1, 2, 3, 4 |
| Relation | NonDiscriminating |
| Proteins | NDKB\_HUMAN, NDKB\_HUMAN, NDK8\_HUMAN |
| Sequence | ``` DRPFFPGLVK ``` |
| Position | 172 |
| PTMs | none |
| 1010\* | Confidence | Green |
| Runs | 1, 2, 3, 4 |
| Relation | Discriminating |
| Proteins | NDKB\_HUMAN, NDKB\_HUMAN |
| Sequence | ``` EISLWFKPEELVDYK ``` |
| Position | 244 |
| PTMs | none |

  

Protein P22392

| Name | NDKB\_HUMAN | | |
| Description | Nucleoside diphosphate kinase B OS Homo sapiens GN NME2 PE 1 SV 1 | | |
| Sequence | ```   1 MANLERTFIA IKPDGVQRGL VGEIIKRFEQ KGFRLVAMKF LRASEEHLKQ HYIDLKDRPF FPGLVKYMNS GPVVAMVWEG LNVVKTGRVM LGETNPADSK 101 PGTIRGDFCI QVGRNIIHGS DSVKSAEKEI SLWFKPEELV DYKSCAHDWV YE ``` | | |
| Evidence | Indistinguishable | | |
| Peptide list | 63\*\*, 347\*\*, 480\*\*, 577\*\*, 688\*\*, 1010\* | | |
| Peptides | 63\*\* | Confidence | Green |
| Runs | 1, 2, 3, 4 |
| Relation | NonDiscriminating |
| Proteins | NDKB\_HUMAN, NDKB\_HUMAN, NDK8\_HUMAN |
| Sequence | ``` NIIHGSDSVK ``` |
| Position | 115 |
| PTMs | none |
| 347\*\* | Confidence | Green |
| Runs | 1, 2, 3, 4 |
| Relation | NonDiscriminating |
| Proteins | NDKB\_HUMAN, NDKB\_HUMAN, NDKA\_HUMAN, NDKA\_HUMAN, NDK8\_HUMAN |
| Sequence | ``` VMLGETNPADSKPGTIR ``` |
| Position | 89 |
| PTMs | none |
| 480\*\* | Confidence | Green |
| Runs | 1, 2, 3, 4 |
| Relation | NonDiscriminating |
| Proteins | NDKB\_HUMAN, NDKB\_HUMAN, NDKA\_HUMAN, NDKA\_HUMAN, NDK8\_HUMAN |
| Sequence | ``` GDFCIQVGR ``` |
| Position | 106 |
| PTMs | Carbamidomethyl+C(4) |
| 577\*\* | Confidence | Green |
| Runs | 1, 2, 3, 4 |
| Relation | NonDiscriminating |
| Proteins | NDKB\_HUMAN, NDKB\_HUMAN, NDKA\_HUMAN, NDKA\_HUMAN |
| Sequence | ``` GLVGEIIK ``` |
| Position | 19 |
| PTMs | none |
| 688\*\* | Confidence | Green |
| Runs | 1, 2, 3, 4 |
| Relation | NonDiscriminating |
| Proteins | NDKB\_HUMAN, NDKB\_HUMAN, NDK8\_HUMAN |
| Sequence | ``` DRPFFPGLVK ``` |
| Position | 57 |
| PTMs | none |
| 1010\* | Confidence | Green |
| Runs | 1, 2, 3, 4 |
| Relation | Discriminating |
| Proteins | NDKB\_HUMAN, NDKB\_HUMAN |
| Sequence | ``` EISLWFKPEELVDYK ``` |
| Position | 129 |
| PTMs | none |

  

Protein P15531

| Name | NDKA\_HUMAN | | |
| Description | Nucleoside diphosphate kinase A OS Homo sapiens GN NME1 PE 1 SV 1 | | |
| Sequence | ```   1 MANCERTFIA IKPDGVQRGL VGEIIKRFEQ KGFRLVGLKF MQASEDLLKE HYVDLKDRPF FAGLVKYMHS GPVVAMVWEG LNVVKTGRVM LGETNPADSK 101 PGTIRGDFCI QVGRNIIHGS DSVESAEKEI GLWFHPEELV DYTSCAQNWI YE ``` | | |
| Evidence | Indistinguishable | | |
| Peptide list | 149\*, 347\*\*, 480\*\*, 577\*\*, 579\*\* | | |
| Peptides | 149\* | Confidence | Green |
| Runs | 1, 2, 3, 4 |
| Relation | Discriminating |
| Proteins | NDKA\_HUMAN, NDKA\_HUMAN |
| Sequence | ``` NIIHGSDSVESAEK ``` |
| Position | 115 |
| PTMs | none |
| 347\*\* | Confidence | Green |
| Runs | 1, 2, 3, 4 |
| Relation | NonDiscriminating |
| Proteins | NDKB\_HUMAN, NDKB\_HUMAN, NDKA\_HUMAN, NDKA\_HUMAN, NDK8\_HUMAN |
| Sequence | ``` VMLGETNPADSKPGTIR ``` |
| Position | 89 |
| PTMs | none |
| 480\*\* | Confidence | Green |
| Runs | 1, 2, 3, 4 |
| Relation | NonDiscriminating |
| Proteins | NDKB\_HUMAN, NDKB\_HUMAN, NDKA\_HUMAN, NDKA\_HUMAN, NDK8\_HUMAN |
| Sequence | ``` GDFCIQVGR ``` |
| Position | 106 |
| PTMs | Carbamidomethyl+C(4) |
| 577\*\* | Confidence | Green |
| Runs | 1, 2, 3, 4 |
| Relation | NonDiscriminating |
| Proteins | NDKB\_HUMAN, NDKB\_HUMAN, NDKA\_HUMAN, NDKA\_HUMAN |
| Sequence | ``` GLVGEIIK ``` |
| Position | 19 |
| PTMs | none |
| 579\*\* | Confidence | Green |
| Runs | 1, 2, 3, 4 |
| Relation | NonDiscriminating |
| Proteins | NDKB\_HUMAN, NDKA\_HUMAN, NDKA\_HUMAN |
| Sequence | ``` FMQASEDLLK ``` |
| Position | 40 |
| PTMs | none |

  

Protein P15531-2

| Name | NDKA\_HUMAN | | |
| Description | Isoform 2 of Nucleoside diphosphate kinase A OS Homo sapiens GN NME1 | | |
| Sequence | ```   1 MVLLSTLGIV FQGEGPPISS CDTGTMANCE RTFIAIKPDG VQRGLVGEII KRFEQKGFRL VGLKFMQASE DLLKEHYVDL KDRPFFAGLV KYMHSGPVVA 101 MVWEGLNVVK TGRVMLGETN PADSKPGTIR GDFCIQVGRN IIHGSDSVES AEKEIGLWFH PEELVDYTSC AQNWIYE ``` | | |
| Evidence | Indistinguishable | | |
| Peptide list | 149\*, 347\*\*, 480\*\*, 577\*\*, 579\*\* | | |
| Peptides | 149\* | Confidence | Green |
| Runs | 1, 2, 3, 4 |
| Relation | Discriminating |
| Proteins | NDKA\_HUMAN, NDKA\_HUMAN |
| Sequence | ``` NIIHGSDSVESAEK ``` |
| Position | 140 |
| PTMs | none |
| 347\*\* | Confidence | Green |
| Runs | 1, 2, 3, 4 |
| Relation | NonDiscriminating |
| Proteins | NDKB\_HUMAN, NDKB\_HUMAN, NDKA\_HUMAN, NDKA\_HUMAN, NDK8\_HUMAN |
| Sequence | ``` VMLGETNPADSKPGTIR ``` |
| Position | 114 |
| PTMs | none |
| 480\*\* | Confidence | Green |
| Runs | 1, 2, 3, 4 |
| Relation | NonDiscriminating |
| Proteins | NDKB\_HUMAN, NDKB\_HUMAN, NDKA\_HUMAN, NDKA\_HUMAN, NDK8\_HUMAN |
| Sequence | ``` GDFCIQVGR ``` |
| Position | 131 |
| PTMs | Carbamidomethyl+C(4) |
| 577\*\* | Confidence | Green |
| Runs | 1, 2, 3, 4 |
| Relation | NonDiscriminating |
| Proteins | NDKB\_HUMAN, NDKB\_HUMAN, NDKA\_HUMAN, NDKA\_HUMAN |
| Sequence | ``` GLVGEIIK ``` |
| Position | 44 |
| PTMs | none |
| 579\*\* | Confidence | Green |
| Runs | 1, 2, 3, 4 |
| Relation | NonDiscriminating |
| Proteins | NDKB\_HUMAN, NDKA\_HUMAN, NDKA\_HUMAN |
| Sequence | ``` FMQASEDLLK ``` |
| Position | 65 |
| PTMs | none |

  

Protein P62258

| Name | 1433E\_HUMAN | | |
| Description | 14 3 3 protein epsilon OS Homo sapiens GN YWHAE PE 1 SV 1 | | |
| Sequence | ```   1 MDDREDLVYQ AKLAEQAERY DEMVESMKKV AGMDVELTVE ERNLLSVAYK NVIGARRASW RIISSIEQKE ENKGGEDKLK MIREYRQMVE TELKLICCDI 101 LDVLDKHLIP AANTGESKVF YYKMKGDYHR YLAEFATGND RKEAAENSLV AYKAASDIAM TELPPTHPIR LGLALNFSVF YYEILNSPDR ACRLAKAAFD 201 DAIAELDTLS EESYKDSTLI MQLLRDNLTL WTSDMQGDGE EQNKEALQDV EDENQ ``` | | |
| Evidence | Indistinguishable | | |
| Peptide list | 112\*, 297\*, 434\*, 538\*\*, 1005\*, 1030\*\*, 1074\* | | |
| Peptides | 112\* | Confidence | Green |
| Runs | 1, 2, 3, 4 |
| Relation | Discriminating |
| Proteins | 1433E\_HUMAN, 1433E\_HUMAN |
| Sequence | ``` HLIPAANTGESK ``` |
| Position | 107 |
| PTMs | none |
| 297\* | Confidence | Green |
| Runs | 1, 3, 4 |
| Relation | Discriminating |
| Proteins | 1433E\_HUMAN, 1433E\_HUMAN |
| Sequence | ``` EAAENSLVAYK ``` |
| Position | 143 |
| PTMs | none |
| 434\* | Confidence | Green |
| Runs | 1, 2, 3, 4 |
| Relation | Discriminating |
| Proteins | 1433E\_HUMAN, 1433E\_HUMAN |
| Sequence | ``` YLAEFATGNDR ``` |
| Position | 131 |
| PTMs | none |
| 538\*\* | Confidence | Green |
| Runs | 1, 2, 3, 4 |
| Relation | NonDiscriminating |
| Proteins | 1433E\_HUMAN, 1433E\_HUMAN, 1433T\_HUMAN, 1433Z\_HUMAN, 1433B\_HUMAN, 1433B\_HUMAN, 1433F\_HUMAN, 1433G\_HUMAN, 1433S\_HUMAN, 1433S\_HUMAN |
| Sequence | ``` NLLSVAYK ``` |
| Position | 43 |
| PTMs | none |
| 1005\* | Confidence | Green |
| Runs | 1, 2, 3, 4 |
| Relation | Discriminating |
| Proteins | 1433E\_HUMAN, 1433E\_HUMAN |
| Sequence | ``` LICCDILDVLDK ``` |
| Position | 95 |
| PTMs | Carbamidomethyl+C(3) Carbamidomethyl+C(4) |
| 1030\*\* | Confidence | Green |
| Runs | 1, 2, 3, 4 |
| Relation | NonDiscriminating |
| Proteins | 1433E\_HUMAN, 1433E\_HUMAN, 1433T\_HUMAN, 1433Z\_HUMAN, 1433B\_HUMAN, 1433B\_HUMAN, 1433F\_HUMAN, 1433G\_HUMAN, 1433S\_HUMAN, 1433S\_HUMAN |
| Sequence | ``` DSTLIMQLLR ``` |
| Position | 216 |
| PTMs | none |
| 1074\* | Confidence | Green |
| Runs | 1, 2, 3, 4 |
| Relation | Discriminating |
| Proteins | 1433E\_HUMAN, 1433E\_HUMAN |
| Sequence | ``` AAFDDAIAELDTLSEESYK ``` |
| Position | 197 |
| PTMs | none |

  

Protein P62258-2

| Name | 1433E\_HUMAN | | |
| Description | Isoform SV of 14 3 3 protein epsilon OS Homo sapiens GN YWHAE | | |
| Sequence | ```   1 MVESMKKVAG MDVELTVEER NLLSVAYKNV IGARRASWRI ISSIEQKEEN KGGEDKLKMI REYRQMVETE LKLICCDILD VLDKHLIPAA NTGESKVFYY 101 KMKGDYHRYL AEFATGNDRK EAAENSLVAY KAASDIAMTE LPPTHPIRLG LALNFSVFYY EILNSPDRAC RLAKAAFDDA IAELDTLSEE SYKDSTLIMQ 201 LLRDNLTLWT SDMQGDGEEQ NKEALQDVED ENQ ``` | | |
| Evidence | Indistinguishable | | |
| Peptide list | 112\*, 297\*, 434\*, 538\*\*, 1005\*, 1030\*\*, 1074\* | | |
| Peptides | 112\* | Confidence | Green |
| Runs | 1, 2, 3, 4 |
| Relation | Discriminating |
| Proteins | 1433E\_HUMAN, 1433E\_HUMAN |
| Sequence | ``` HLIPAANTGESK ``` |
| Position | 85 |
| PTMs | none |
| 297\* | Confidence | Green |
| Runs | 1, 3, 4 |
| Relation | Discriminating |
| Proteins | 1433E\_HUMAN, 1433E\_HUMAN |
| Sequence | ``` EAAENSLVAYK ``` |
| Position | 121 |
| PTMs | none |
| 434\* | Confidence | Green |
| Runs | 1, 2, 3, 4 |
| Relation | Discriminating |
| Proteins | 1433E\_HUMAN, 1433E\_HUMAN |
| Sequence | ``` YLAEFATGNDR ``` |
| Position | 109 |
| PTMs | none |
| 538\*\* | Confidence | Green |
| Runs | 1, 2, 3, 4 |
| Relation | NonDiscriminating |
| Proteins | 1433E\_HUMAN, 1433E\_HUMAN, 1433T\_HUMAN, 1433Z\_HUMAN, 1433B\_HUMAN, 1433B\_HUMAN, 1433F\_HUMAN, 1433G\_HUMAN, 1433S\_HUMAN, 1433S\_HUMAN |
| Sequence | ``` NLLSVAYK ``` |
| Position | 21 |
| PTMs | none |
| 1005\* | Confidence | Green |
| Runs | 1, 2, 3, 4 |
| Relation | Discriminating |
| Proteins | 1433E\_HUMAN, 1433E\_HUMAN |
| Sequence | ``` LICCDILDVLDK ``` |
| Position | 73 |
| PTMs | Carbamidomethyl+C(3) Carbamidomethyl+C(4) |
| 1030\*\* | Confidence | Green |
| Runs | 1, 2, 3, 4 |
| Relation | NonDiscriminating |
| Proteins | 1433E\_HUMAN, 1433E\_HUMAN, 1433T\_HUMAN, 1433Z\_HUMAN, 1433B\_HUMAN, 1433B\_HUMAN, 1433F\_HUMAN, 1433G\_HUMAN, 1433S\_HUMAN, 1433S\_HUMAN |
| Sequence | ``` DSTLIMQLLR ``` |
| Position | 194 |
| PTMs | none |
| 1074\* | Confidence | Green |
| Runs | 1, 2, 3, 4 |
| Relation | Discriminating |
| Proteins | 1433E\_HUMAN, 1433E\_HUMAN |
| Sequence | ``` AAFDDAIAELDTLSEESYK ``` |
| Position | 175 |
| PTMs | none |

  

Protein P31946

| Name | 1433B\_HUMAN | | |
| Description | 14 3 3 protein beta alpha OS Homo sapiens GN YWHAB PE 1 SV 3 | | |
| Sequence | ```   1 MTMDKSELVQ KAKLAEQAER YDDMAAAMKA VTEQGHELSN EERNLLSVAY KNVVGARRSS WRVISSIEQK TERNEKKQQM GKEYREKIEA ELQDICNDVL 101 ELLDKYLIPN ATQPESKVFY LKMKGDYFRY LSEVASGDNK QTTVSNSQQA YQEAFEISKK EMQPTHPIRL GLALNFSVFY YEILNSPEKA CSLAKTAFDE 201 AIAELDTLNE ESYKDSTLIM QLLRDNLTLW TSENQGDEGD AGEGEN ``` | | |
| Evidence | Indistinguishable | | |
| Peptide list | 57\*\*, 66\*, 88\*\*, 538\*\*, 1030\*\*, 1070\* | | |
| Peptides | 57\*\* | Confidence | Green |
| Runs | 1, 2, 4 |
| Relation | NonDiscriminating |
| Proteins | 1433T\_HUMAN, 1433Z\_HUMAN, 1433B\_HUMAN, 1433B\_HUMAN |
| Sequence | ``` EMQPTHPIR ``` |
| Position | 161 |
| PTMs | none |
| 66\* | Confidence | Green |
| Runs | 1, 2, 3, 4 |
| Relation | Discriminating |
| Proteins | 1433B\_HUMAN, 1433B\_HUMAN |
| Sequence | ``` AVTEQGHELSNEER ``` |
| Position | 30 |
| PTMs | none |
| 88\*\* | Confidence | Green |
| Runs | 1, 2, 3, 4 |
| Relation | NonDiscriminating |
| Proteins | 1433T\_HUMAN, 1433B\_HUMAN, 1433B\_HUMAN, 1433F\_HUMAN, 1433G\_HUMAN |
| Sequence | ``` VISSIEQK ``` |
| Position | 63 |
| PTMs | none |
| 538\*\* | Confidence | Green |
| Runs | 1, 2, 3, 4 |
| Relation | NonDiscriminating |
| Proteins | 1433E\_HUMAN, 1433E\_HUMAN, 1433T\_HUMAN, 1433Z\_HUMAN, 1433B\_HUMAN, 1433B\_HUMAN, 1433F\_HUMAN, 1433G\_HUMAN, 1433S\_HUMAN, 1433S\_HUMAN |
| Sequence | ``` NLLSVAYK ``` |
| Position | 44 |
| PTMs | none |
| 1030\*\* | Confidence | Green |
| Runs | 1, 2, 3, 4 |
| Relation | NonDiscriminating |
| Proteins | 1433E\_HUMAN, 1433E\_HUMAN, 1433T\_HUMAN, 1433Z\_HUMAN, 1433B\_HUMAN, 1433B\_HUMAN, 1433F\_HUMAN, 1433G\_HUMAN, 1433S\_HUMAN, 1433S\_HUMAN |
| Sequence | ``` DSTLIMQLLR ``` |
| Position | 215 |
| PTMs | none |
| 1070\* | Confidence | Green |
| Runs | 1, 3, 4 |
| Relation | Discriminating |
| Proteins | 1433B\_HUMAN, 1433B\_HUMAN |
| Sequence | ``` TAFDEAIAELDTLNEESYK ``` |
| Position | 196 |
| PTMs | none |

  

Protein P31946-2

| Name | 1433B\_HUMAN | | |
| Description | Isoform Short of 14 3 3 protein beta alpha OS Homo sapiens GN YWHAB | | |
| Sequence | ```   1 MDKSELVQKA KLAEQAERYD DMAAAMKAVT EQGHELSNEE RNLLSVAYKN VVGARRSSWR VISSIEQKTE RNEKKQQMGK EYREKIEAEL QDICNDVLEL 101 LDKYLIPNAT QPESKVFYLK MKGDYFRYLS EVASGDNKQT TVSNSQQAYQ EAFEISKKEM QPTHPIRLGL ALNFSVFYYE ILNSPEKACS LAKTAFDEAI 201 AELDTLNEES YKDSTLIMQL LRDNLTLWTS ENQGDEGDAG EGEN ``` | | |
| Evidence | Indistinguishable | | |
| Peptide list | 57\*\*, 66\*, 88\*\*, 538\*\*, 1030\*\*, 1070\* | | |
| Peptides | 57\*\* | Confidence | Green |
| Runs | 1, 2, 4 |
| Relation | NonDiscriminating |
| Proteins | 1433T\_HUMAN, 1433Z\_HUMAN, 1433B\_HUMAN, 1433B\_HUMAN |
| Sequence | ``` EMQPTHPIR ``` |
| Position | 159 |
| PTMs | none |
| 66\* | Confidence | Green |
| Runs | 1, 2, 3, 4 |
| Relation | Discriminating |
| Proteins | 1433B\_HUMAN, 1433B\_HUMAN |
| Sequence | ``` AVTEQGHELSNEER ``` |
| Position | 28 |
| PTMs | none |
| 88\*\* | Confidence | Green |
| Runs | 1, 2, 3, 4 |
| Relation | NonDiscriminating |
| Proteins | 1433T\_HUMAN, 1433B\_HUMAN, 1433B\_HUMAN, 1433F\_HUMAN, 1433G\_HUMAN |
| Sequence | ``` VISSIEQK ``` |
| Position | 61 |
| PTMs | none |
| 538\*\* | Confidence | Green |
| Runs | 1, 2, 3, 4 |
| Relation | NonDiscriminating |
| Proteins | 1433E\_HUMAN, 1433E\_HUMAN, 1433T\_HUMAN, 1433Z\_HUMAN, 1433B\_HUMAN, 1433B\_HUMAN, 1433F\_HUMAN, 1433G\_HUMAN, 1433S\_HUMAN, 1433S\_HUMAN |
| Sequence | ``` NLLSVAYK ``` |
| Position | 42 |
| PTMs | none |
| 1030\*\* | Confidence | Green |
| Runs | 1, 2, 3, 4 |
| Relation | NonDiscriminating |
| Proteins | 1433E\_HUMAN, 1433E\_HUMAN, 1433T\_HUMAN, 1433Z\_HUMAN, 1433B\_HUMAN, 1433B\_HUMAN, 1433F\_HUMAN, 1433G\_HUMAN, 1433S\_HUMAN, 1433S\_HUMAN |
| Sequence | ``` DSTLIMQLLR ``` |
| Position | 213 |
| PTMs | none |
| 1070\* | Confidence | Green |
| Runs | 1, 3, 4 |
| Relation | Discriminating |
| Proteins | 1433B\_HUMAN, 1433B\_HUMAN |
| Sequence | ``` TAFDEAIAELDTLNEESYK ``` |
| Position | 194 |
| PTMs | none |

  

Protein P31947

| Name | 1433S\_HUMAN | | |
| Description | 14 3 3 protein sigma OS Homo sapiens GN SFN PE 1 SV 1 | | |
| Sequence | ```   1 MERASLIQKA KLAEQAERYE DMAAFMKGAV EKGEELSCEE RNLLSVAYKN VVGGQRAAWR VLSSIEQKSN EEGSEEKGPE VREYREKVET ELQGVCDTVL 101 GLLDSHLIKE AGDAESRVFY LKMKGDYYRY LAEVATGDDK KRIIDSARSA YQEAMDISKK EMPPTNPIRL GLALNFSVFH YEIANSPEEA ISLAKTTFDE 201 AMADLHTLSE DSYKDSTLIM QLLRDNLTLW TADNAGEEGG EAPQEPQS ``` | | |
| Evidence | Indistinguishable | | |
| Peptide list | 538\*\*, 807\*, 1030\*\*, 1253\* | | |
| Peptides | 538\*\* | Confidence | Green |
| Runs | 1, 2, 3, 4 |
| Relation | NonDiscriminating |
| Proteins | 1433E\_HUMAN, 1433E\_HUMAN, 1433T\_HUMAN, 1433Z\_HUMAN, 1433B\_HUMAN, 1433B\_HUMAN, 1433F\_HUMAN, 1433G\_HUMAN, 1433S\_HUMAN, 1433S\_HUMAN |
| Sequence | ``` NLLSVAYK ``` |
| Position | 42 |
| PTMs | none |
| 807\* | Confidence | Green |
| Runs | 1, 3, 4 |
| Relation | Discriminating |
| Proteins | 1433S\_HUMAN, 1433S\_HUMAN |
| Sequence | ``` DNLTLWTADNAGEEGGEAPQEPQS ``` |
| Position | 225 |
| PTMs | Variant #1: Deamidation+Q(20) Deamidation+Q(23)  Variant #2: none |
| 1030\*\* | Confidence | Green |
| Runs | 1, 2, 3, 4 |
| Relation | NonDiscriminating |
| Proteins | 1433E\_HUMAN, 1433E\_HUMAN, 1433T\_HUMAN, 1433Z\_HUMAN, 1433B\_HUMAN, 1433B\_HUMAN, 1433F\_HUMAN, 1433G\_HUMAN, 1433S\_HUMAN, 1433S\_HUMAN |
| Sequence | ``` DSTLIMQLLR ``` |
| Position | 215 |
| PTMs | none |
| 1253\* | Confidence | Yellow |
| Runs | 2, 3, 4 |
| Relation | Discriminating |
| Proteins | 1433S\_HUMAN, 1433S\_HUMAN |
| Sequence | ``` SAYQEAMDISK ``` |
| Position | 149 |
| PTMs | none |

  

Protein P31947-2

| Name | 1433S\_HUMAN | | |
| Description | Isoform 2 of 14 3 3 protein sigma OS Homo sapiens GN SFN | | |
| Sequence | ```   1 MERASLIQKA KLAEQAERYE DMAAFMKGAV EKGEELSCEE RNLLSVAYKN VVGGQRAAWR VLSSIEQKSN EEGSEEKGPE VREYRVFYLK MKGDYYRYLA 101 EVATGDDKKR IIDSARSAYQ EAMDISKKEM PPTNPIRLGL ALNFSVFHYE IANSPEEAIS LAKTTFDEAM ADLHTLSEDS YKDSTLIMQL LRDNLTLWTA 201 DNAGEEGGEA PQEPQS ``` | | |
| Evidence | Indistinguishable | | |
| Peptide list | 538\*\*, 807\*, 1030\*\*, 1253\* | | |
| Peptides | 538\*\* | Confidence | Green |
| Runs | 1, 2, 3, 4 |
| Relation | NonDiscriminating |
| Proteins | 1433E\_HUMAN, 1433E\_HUMAN, 1433T\_HUMAN, 1433Z\_HUMAN, 1433B\_HUMAN, 1433B\_HUMAN, 1433F\_HUMAN, 1433G\_HUMAN, 1433S\_HUMAN, 1433S\_HUMAN |
| Sequence | ``` NLLSVAYK ``` |
| Position | 42 |
| PTMs | none |
| 807\* | Confidence | Green |
| Runs | 1, 3, 4 |
| Relation | Discriminating |
| Proteins | 1433S\_HUMAN, 1433S\_HUMAN |
| Sequence | ``` DNLTLWTADNAGEEGGEAPQEPQS ``` |
| Position | 193 |
| PTMs | Variant #1: Deamidation+Q(20) Deamidation+Q(23)  Variant #2: none |
| 1030\*\* | Confidence | Green |
| Runs | 1, 2, 3, 4 |
| Relation | NonDiscriminating |
| Proteins | 1433E\_HUMAN, 1433E\_HUMAN, 1433T\_HUMAN, 1433Z\_HUMAN, 1433B\_HUMAN, 1433B\_HUMAN, 1433F\_HUMAN, 1433G\_HUMAN, 1433S\_HUMAN, 1433S\_HUMAN |
| Sequence | ``` DSTLIMQLLR ``` |
| Position | 183 |
| PTMs | none |
| 1253\* | Confidence | Yellow |
| Runs | 2, 3, 4 |
| Relation | Discriminating |
| Proteins | 1433S\_HUMAN, 1433S\_HUMAN |
| Sequence | ``` SAYQEAMDISK ``` |
| Position | 117 |
| PTMs | none |

  

Protein P09651

| Name | ROA1\_HUMAN | | |
| Description | Heterogeneous nuclear ribonucleoprotein A1 OS Homo sapiens GN HNRNPA1 PE 1 SV 5 | | |
| Sequence | ```   1 MSKSESPKEP EQLRKLFIGG LSFETTDESL RSHFEQWGTL TDCVVMRDPN TKRSRGFGFV TYATVEEVDA AMNARPHKVD GRVVEPKRAV SREDSQRPGA 101 HLTVKKIFVG GIKEDTEEHH LRDYFEQYGK IEVIEIMTDR GSGKKRGFAF VTFDDHDSVD KIVIQKYHTV NGHNCEVRKA LSKQEMASAS SSQRGRSGSG 201 NFGGGRGGGF GGNDNFGRGG NFSGRGGFGG SRGGGGYGGS GDGYNGFGND GGYGGGGPGY SGGSRGYGSG GQGYGNQGSG YGGSGSYDSY NNGGGGGFGG 301 GSGSNFGGGG SYNDFGNYNN QSSNFGPMKG GNFGGRSSGP YGGGGQYFAK PRNQGGYGGS SSSSSYGSGR RF ``` | | |
| Evidence | Indistinguishable | | |
| Peptide list | 8\*, 13\*\*, 37\*\*, 61\*\*, 269\*\*, 741\*\*, 782\*\* | | |
| Peptides | 8\* | Confidence | Green |
| Runs | 1, 3, 4 |
| Relation | Discriminating |
| Proteins | ROA1\_HUMAN, ROA1\_HUMAN |
| Sequence | ``` GGGFGGNDNFGR ``` |
| Position | 207 |
| PTMs | Variant #1: Deamidation+N(7) Deamidation+N(9)  Variant #2: none |
| 13\*\* | Confidence | Green |
| Runs | 1, 2, 3, 4 |
| Relation | NonDiscriminating |
| Proteins | ROA1\_HUMAN, ROA1\_HUMAN, ROA1\_HUMAN |
| Sequence | ``` YHTVNGHNCEVR ``` |
| Position | 167 |
| PTMs | Variant #1: Carbamidomethyl+C(9)  Variant #2: Carbamidomethyl+C(9) Deamidation+N(5) |
| 37\*\* | Confidence | Green |
| Runs | 1, 2, 3, 4 |
| Relation | NonDiscriminating |
| Proteins | ROA1\_HUMAN, ROA1\_HUMAN, ROA1\_HUMAN |
| Sequence | ``` NQGGYGGSSSSSSYGSGR ``` |
| Position | 353 |
| PTMs | none |
| 61\*\* | Confidence | Green |
| Runs | 1, 2, 3, 4 |
| Relation | NonDiscriminating |
| Proteins | ROA1\_HUMAN, ROA1\_HUMAN, ROA1\_HUMAN, RA1L2\_HUMAN |
| Sequence | ``` EDSQRPGAHLTVK ``` |
| Position | 93 |
| PTMs | none |
| 269\*\* | Confidence | Green |
| Runs | 1, 3, 4 |
| Relation | NonDiscriminating |
| Proteins | ROA2\_HUMAN, ROA2\_HUMAN, ROA1\_HUMAN, ROA1\_HUMAN, ROA1\_HUMAN, RA1L2\_HUMAN |
| Sequence | ``` LTDCVVMR ``` |
| Position | 40 |
| PTMs | Carbamidomethyl+C(4) |
| 741\*\* | Confidence | Green |
| Runs | 1, 2, 3, 4 |
| Relation | NonDiscriminating |
| Proteins | ROA1\_HUMAN, ROA1\_HUMAN, ROA1\_HUMAN, RA1L2\_HUMAN |
| Sequence | ``` GFAFVTFDDHDSVDK ``` |
| Position | 147 |
| PTMs | none |
| 782\*\* | Confidence | Green |
| Runs | 1, 2, 3, 4 |
| Relation | NonDiscriminating |
| Proteins | ROA1\_HUMAN, ROA1\_HUMAN, ROA1\_HUMAN, RA1L2\_HUMAN |
| Sequence | ``` IEVIEIMTDR ``` |
| Position | 131 |
| PTMs | none |

  

Protein P09651-2

| Name | ROA1\_HUMAN | | |
| Description | Isoform A1 A of Heterogeneous nuclear ribonucleoprotein A1 OS Homo sapiens GN HNRNPA1 | | |
| Sequence | ```   1 MSKSESPKEP EQLRKLFIGG LSFETTDESL RSHFEQWGTL TDCVVMRDPN TKRSRGFGFV TYATVEEVDA AMNARPHKVD GRVVEPKRAV SREDSQRPGA 101 HLTVKKIFVG GIKEDTEEHH LRDYFEQYGK IEVIEIMTDR GSGKKRGFAF VTFDDHDSVD KIVIQKYHTV NGHNCEVRKA LSKQEMASAS SSQRGRSGSG 201 NFGGGRGGGF GGNDNFGRGG NFSGRGGFGG SRGGGGYGGS GDGYNGFGND GSNFGGGGSY NDFGNYNNQS SNFGPMKGGN FGGRSSGPYG GGGQYFAKPR 301 NQGGYGGSSS SSSYGSGRRF ``` | | |
| Evidence | Indistinguishable | | |
| Peptide list | 8\*, 13\*\*, 37\*\*, 61\*\*, 269\*\*, 741\*\*, 782\*\* | | |
| Peptides | 8\* | Confidence | Green |
| Runs | 1, 3, 4 |
| Relation | Discriminating |
| Proteins | ROA1\_HUMAN, ROA1\_HUMAN |
| Sequence | ``` GGGFGGNDNFGR ``` |
| Position | 207 |
| PTMs | Variant #1: Deamidation+N(7) Deamidation+N(9)  Variant #2: none |
| 13\*\* | Confidence | Green |
| Runs | 1, 2, 3, 4 |
| Relation | NonDiscriminating |
| Proteins | ROA1\_HUMAN, ROA1\_HUMAN, ROA1\_HUMAN |
| Sequence | ``` YHTVNGHNCEVR ``` |
| Position | 167 |
| PTMs | Variant #1: Carbamidomethyl+C(9)  Variant #2: Carbamidomethyl+C(9) Deamidation+N(5) |
| 37\*\* | Confidence | Green |
| Runs | 1, 2, 3, 4 |
| Relation | NonDiscriminating |
| Proteins | ROA1\_HUMAN, ROA1\_HUMAN, ROA1\_HUMAN |
| Sequence | ``` NQGGYGGSSSSSSYGSGR ``` |
| Position | 301 |
| PTMs | none |
| 61\*\* | Confidence | Green |
| Runs | 1, 2, 3, 4 |
| Relation | NonDiscriminating |
| Proteins | ROA1\_HUMAN, ROA1\_HUMAN, ROA1\_HUMAN, RA1L2\_HUMAN |
| Sequence | ``` EDSQRPGAHLTVK ``` |
| Position | 93 |
| PTMs | none |
| 269\*\* | Confidence | Green |
| Runs | 1, 3, 4 |
| Relation | NonDiscriminating |
| Proteins | ROA2\_HUMAN, ROA2\_HUMAN, ROA1\_HUMAN, ROA1\_HUMAN, ROA1\_HUMAN, RA1L2\_HUMAN |
| Sequence | ``` LTDCVVMR ``` |
| Position | 40 |
| PTMs | Carbamidomethyl+C(4) |
| 741\*\* | Confidence | Green |
| Runs | 1, 2, 3, 4 |
| Relation | NonDiscriminating |
| Proteins | ROA1\_HUMAN, ROA1\_HUMAN, ROA1\_HUMAN, RA1L2\_HUMAN |
| Sequence | ``` GFAFVTFDDHDSVDK ``` |
| Position | 147 |
| PTMs | none |
| 782\*\* | Confidence | Green |
| Runs | 1, 2, 3, 4 |
| Relation | NonDiscriminating |
| Proteins | ROA1\_HUMAN, ROA1\_HUMAN, ROA1\_HUMAN, RA1L2\_HUMAN |
| Sequence | ``` IEVIEIMTDR ``` |
| Position | 131 |
| PTMs | none |

  

Protein P61978

| Name | HNRPK\_HUMAN | | |
| Description | Heterogeneous nuclear ribonucleoprotein K OS Homo sapiens GN HNRNPK PE 1 SV 1 | | |
| Sequence | ```   1 METEQPEETF PNTETNGEFG KRPAEDMEEE QAFKRSRNTD EMVELRILLQ SKNAGAVIGK GGKNIKALRT DYNASVSVPD SSGPERILSI SADIETIGEI 101 LKKIIPTLEE GLQLPSPTAT SQLPLESDAV ECLNYQHYKG SDFDCELRLL IHQSLAGGII GVKGAKIKEL RENTQTTIKL FQECCPHSTD RVVLIGGKPD 201 RVVECIKIIL DLISESPIKG RAQPYDPNFY DETYDYGGFT MMFDDRRGRP VGFPMRGRGG FDRMPPGRGG RPMPPSRRDY DDMSPRRGPP PPPPGRGGRG 301 GSRARNLPLP PPPPPRGGDL MAYDRRGRPG DRYDGMVGFS ADETWDSAID TWSPSEWQMA YEPQGGSGYD YSYAGGRGSY GDLGGPIITT QVTIPKDLAG 401 SIIGKGGQRI KQIRHESGAS IKIDEPLEGS EDRIITITGT QDQIQNAQYL LQNSVKQYSG KFF ``` | | |
| Evidence | Indistinguishable | | |
| Peptide list | 144\*\*, 226\*\*, 240\*\*, 314\*\*, 329\*\*, 798\*\*, 967\*\*, 971\*\*, 1038\* | | |
| Peptides | 144\*\* | Confidence | Green |
| Runs | 1, 2, 3 |
| Relation | NonDiscriminating |
| Proteins | HNRPK\_HUMAN, HNRPK\_HUMAN, HNRPK\_HUMAN |
| Sequence | ``` ILLQSK ``` |
| Position | 47 |
| PTMs | none |
| 226\*\* | Confidence | Green |
| Runs | 1, 2, 4 |
| Relation | NonDiscriminating |
| Proteins | HNRPK\_HUMAN, HNRPK\_HUMAN, HNRPK\_HUMAN |
| Sequence | ``` IDEPLEGSEDR ``` |
| Position | 423 |
| PTMs | none |
| 240\*\* | Confidence | Green |
| Runs | 1, 2, 4 |
| Relation | NonDiscriminating |
| Proteins | HNRPK\_HUMAN, HNRPK\_HUMAN, HNRPK\_HUMAN |
| Sequence | ``` RPAEDMEEEQAFK ``` |
| Position | 22 |
| PTMs | none |
| 314\*\* | Confidence | Green |
| Runs | 1, 2, 4 |
| Relation | NonDiscriminating |
| Proteins | HNRPK\_HUMAN, HNRPK\_HUMAN, HNRPK\_HUMAN |
| Sequence | ``` GSDFDCELR ``` |
| Position | 140 |
| PTMs | Carbamidomethyl+C(6) |
| 329\*\* | Confidence | Green |
| Runs | 1, 2, 3, 4 |
| Relation | NonDiscriminating |
| Proteins | HNRPK\_HUMAN, HNRPK\_HUMAN, HNRPK\_HUMAN |
| Sequence | ``` TDYNASVSVPDSSGPER ``` |
| Position | 70 |
| PTMs | none |
| 798\*\* | Confidence | Green |
| Runs | 1, 2, 3, 4 |
| Relation | NonDiscriminating |
| Proteins | HNRPK\_HUMAN, HNRPK\_HUMAN, HNRPK\_HUMAN |
| Sequence | ``` GSYGDLGGPIITTQVTIPK ``` |
| Position | 378 |
| PTMs | none |
| 967\*\* | Confidence | Green |
| Runs | 1, 2, 3, 4 |
| Relation | NonDiscriminating |
| Proteins | HNRPK\_HUMAN, HNRPK\_HUMAN, HNRPK\_HUMAN |
| Sequence | ``` IILDLISESPIK ``` |
| Position | 208 |
| PTMs | none |
| 971\*\* | Confidence | Green |
| Runs | 1, 2, 3, 4 |
| Relation | NonDiscriminating |
| Proteins | HNRPK\_HUMAN, HNRPK\_HUMAN, HNRPK\_HUMAN |
| Sequence | ``` IITITGTQDQIQNAQYLLQNSVK ``` |
| Position | 434 |
| PTMs | Variant #1: none Variant #2: Deamidation+N(13) |
| 1038\* | Confidence | Green |
| Runs | 1, 2, 4 |
| Relation | Discriminating |
| Proteins | HNRPK\_HUMAN, HNRPK\_HUMAN |
| Sequence | ``` IIPTLEEGLQLPSPTATSQLPLESDAVECLNYQHYK ``` |
| Position | 104 |
| PTMs | Carbamidomethyl+C(29) |

  

Protein P61978-2

| Name | HNRPK\_HUMAN | | |
| Description | Isoform 2 of Heterogeneous nuclear ribonucleoprotein K OS Homo sapiens GN HNRNPK | | |
| Sequence | ```   1 METEQPEETF PNTETNGEFG KRPAEDMEEE QAFKRSRNTD EMVELRILLQ SKNAGAVIGK GGKNIKALRT DYNASVSVPD SSGPERILSI SADIETIGEI 101 LKKIIPTLEE GLQLPSPTAT SQLPLESDAV ECLNYQHYKG SDFDCELRLL IHQSLAGGII GVKGAKIKEL RENTQTTIKL FQECCPHSTD RVVLIGGKPD 201 RVVECIKIIL DLISESPIKG RAQPYDPNFY DETYDYGGFT MMFDDRRGRP VGFPMRGRGG FDRMPPGRGG RPMPPSRRDY DDMSPRRGPP PPPPGRGGRG 301 GSRARNLPLP PPPPPRGGDL MAYDRRGRPG DRYDGMVGFS ADETWDSAID TWSPSEWQMA YEPQGGSGYD YSYAGGRGSY GDLGGPIITT QVTIPKDLAG 401 SIIGKGGQRI KQIRHESGAS IKIDEPLEGS EDRIITITGT QDQIQNAQYL LQNSVKQYAD VEGF ``` | | |
| Evidence | Indistinguishable | | |
| Peptide list | 144\*\*, 226\*\*, 240\*\*, 314\*\*, 329\*\*, 798\*\*, 967\*\*, 971\*\*, 1038\* | | |
| Peptides | 144\*\* | Confidence | Green |
| Runs | 1, 2, 3 |
| Relation | NonDiscriminating |
| Proteins | HNRPK\_HUMAN, HNRPK\_HUMAN, HNRPK\_HUMAN |
| Sequence | ``` ILLQSK ``` |
| Position | 47 |
| PTMs | none |
| 226\*\* | Confidence | Green |
| Runs | 1, 2, 4 |
| Relation | NonDiscriminating |
| Proteins | HNRPK\_HUMAN, HNRPK\_HUMAN, HNRPK\_HUMAN |
| Sequence | ``` IDEPLEGSEDR ``` |
| Position | 423 |
| PTMs | none |
| 240\*\* | Confidence | Green |
| Runs | 1, 2, 4 |
| Relation | NonDiscriminating |
| Proteins | HNRPK\_HUMAN, HNRPK\_HUMAN, HNRPK\_HUMAN |
| Sequence | ``` RPAEDMEEEQAFK ``` |
| Position | 22 |
| PTMs | none |
| 314\*\* | Confidence | Green |
| Runs | 1, 2, 4 |
| Relation | NonDiscriminating |
| Proteins | HNRPK\_HUMAN, HNRPK\_HUMAN, HNRPK\_HUMAN |
| Sequence | ``` GSDFDCELR ``` |
| Position | 140 |
| PTMs | Carbamidomethyl+C(6) |
| 329\*\* | Confidence | Green |
| Runs | 1, 2, 3, 4 |
| Relation | NonDiscriminating |
| Proteins | HNRPK\_HUMAN, HNRPK\_HUMAN, HNRPK\_HUMAN |
| Sequence | ``` TDYNASVSVPDSSGPER ``` |
| Position | 70 |
| PTMs | none |
| 798\*\* | Confidence | Green |
| Runs | 1, 2, 3, 4 |
| Relation | NonDiscriminating |
| Proteins | HNRPK\_HUMAN, HNRPK\_HUMAN, HNRPK\_HUMAN |
| Sequence | ``` GSYGDLGGPIITTQVTIPK ``` |
| Position | 378 |
| PTMs | none |
| 967\*\* | Confidence | Green |
| Runs | 1, 2, 3, 4 |
| Relation | NonDiscriminating |
| Proteins | HNRPK\_HUMAN, HNRPK\_HUMAN, HNRPK\_HUMAN |
| Sequence | ``` IILDLISESPIK ``` |
| Position | 208 |
| PTMs | none |
| 971\*\* | Confidence | Green |
| Runs | 1, 2, 3, 4 |
| Relation | NonDiscriminating |
| Proteins | HNRPK\_HUMAN, HNRPK\_HUMAN, HNRPK\_HUMAN |
| Sequence | ``` IITITGTQDQIQNAQYLLQNSVK ``` |
| Position | 434 |
| PTMs | Variant #1: none Variant #2: Deamidation+N(13) |
| 1038\* | Confidence | Green |
| Runs | 1, 2, 4 |
| Relation | Discriminating |
| Proteins | HNRPK\_HUMAN, HNRPK\_HUMAN |
| Sequence | ``` IIPTLEEGLQLPSPTATSQLPLESDAVECLNYQHYK ``` |
| Position | 104 |
| PTMs | Carbamidomethyl+C(29) |

  

Protein Q96QV6

| Name | H2A1A\_HUMAN | | |
| Description | Histone H2A type 1 A OS Homo sapiens GN HIST1H2AA PE 1 SV 3 | | |
| Sequence | ```   1 MSGRGKQGGK ARAKSKSRSS RAGLQFPVGR IHRLLRKGNY AERIGAGAPV YLAAVLEYLT AEILELAGNA SRDNKKTRII PRHLQLAIRN DEELNKLLGG 101 VTIAQGGVLP NIQAVLLPKK TESHHHKAQS K ``` | | |
| Evidence | Indistinguishable | | |
| Peptide list | 298\*\*, 551\*\*, 1055\* | | |
| Peptides | 298\*\* | Confidence | Green |
| Runs | 1, 2, 3, 4 |
| Relation | NonDiscriminating |
| Proteins | H2A1A\_HUMAN, H2AX\_HUMAN, H2A1J\_HUMAN, H2A2A\_HUMAN, H2A3\_HUMAN, H2A1B\_HUMAN, H2A2C\_HUMAN, H2A1C\_HUMAN, H2AJ\_HUMAN, H2A1\_HUMAN, H2A1D\_HUMAN, H2A1H\_HUMAN, H2AJ\_HUMAN, H2AZ\_HUMAN, H2AV\_HUMAN |
| Sequence | ``` HLQLAIR ``` |
| Position | 83 |
| PTMs | none |
| 551\*\* | Confidence | Green |
| Runs | 1, 2, 3, 4 |
| Relation | NonDiscriminating |
| Proteins | H2A1A\_HUMAN, H2AX\_HUMAN, H2A1J\_HUMAN, H2A2A\_HUMAN, H2A3\_HUMAN, H2A1B\_HUMAN, H2A2C\_HUMAN, H2A1C\_HUMAN, H2AJ\_HUMAN, H2A1\_HUMAN, H2A1D\_HUMAN, H2A1H\_HUMAN, H2A2B\_HUMAN, H2AJ\_HUMAN, H2AZ\_HUMAN, H2AV\_HUMAN |
| Sequence | ``` AGLQFPVGR ``` |
| Position | 22 |
| PTMs | none |
| 1055\* | Confidence | Green |
| Runs | 1, 2, 3, 4 |
| Relation | Discriminating |
| Proteins | H2A1A\_HUMAN, H2AX\_HUMAN, H2A1J\_HUMAN, H2A2A\_HUMAN, H2A3\_HUMAN, H2A1B\_HUMAN, H2A2C\_HUMAN, H2A1C\_HUMAN, H2AJ\_HUMAN, H2A1\_HUMAN, H2A1D\_HUMAN, H2A1H\_HUMAN, H2A2B\_HUMAN |
| Sequence | ``` VTIAQGGVLPNIQAVLLPK ``` |
| Position | 101 |
| PTMs | Variant #1: none Variant #2: Deamidation+Q(5) |

  

Protein P16104

| Name | H2AX\_HUMAN | | |
| Description | Histone H2A x OS Homo sapiens GN H2AFX PE 1 SV 2 | | |
| Sequence | ```   1 MSGRGKTGGK ARAKAKSRSS RAGLQFPVGR VHRLLRKGHY AERVGAGAPV YLAAVLEYLT AEILELAGNA ARDNKKTRII PRHLQLAIRN DEELNKLLGG 101 VTIAQGGVLP NIQAVLLPKK TSATVGPKAP SGGKKATQAS QEY ``` | | |
| Evidence | Indistinguishable | | |
| Peptide list | 298\*\*, 551\*\*, 1055\* | | |
| Peptides | 298\*\* | Confidence | Green |
| Runs | 1, 2, 3, 4 |
| Relation | NonDiscriminating |
| Proteins | H2A1A\_HUMAN, H2AX\_HUMAN, H2A1J\_HUMAN, H2A2A\_HUMAN, H2A3\_HUMAN, H2A1B\_HUMAN, H2A2C\_HUMAN, H2A1C\_HUMAN, H2AJ\_HUMAN, H2A1\_HUMAN, H2A1D\_HUMAN, H2A1H\_HUMAN, H2AJ\_HUMAN, H2AZ\_HUMAN, H2AV\_HUMAN |
| Sequence | ``` HLQLAIR ``` |
| Position | 83 |
| PTMs | none |
| 551\*\* | Confidence | Green |
| Runs | 1, 2, 3, 4 |
| Relation | NonDiscriminating |
| Proteins | H2A1A\_HUMAN, H2AX\_HUMAN, H2A1J\_HUMAN, H2A2A\_HUMAN, H2A3\_HUMAN, H2A1B\_HUMAN, H2A2C\_HUMAN, H2A1C\_HUMAN, H2AJ\_HUMAN, H2A1\_HUMAN, H2A1D\_HUMAN, H2A1H\_HUMAN, H2A2B\_HUMAN, H2AJ\_HUMAN, H2AZ\_HUMAN, H2AV\_HUMAN |
| Sequence | ``` AGLQFPVGR ``` |
| Position | 22 |
| PTMs | none |
| 1055\* | Confidence | Green |
| Runs | 1, 2, 3, 4 |
| Relation | Discriminating |
| Proteins | H2A1A\_HUMAN, H2AX\_HUMAN, H2A1J\_HUMAN, H2A2A\_HUMAN, H2A3\_HUMAN, H2A1B\_HUMAN, H2A2C\_HUMAN, H2A1C\_HUMAN, H2AJ\_HUMAN, H2A1\_HUMAN, H2A1D\_HUMAN, H2A1H\_HUMAN, H2A2B\_HUMAN |
| Sequence | ``` VTIAQGGVLPNIQAVLLPK ``` |
| Position | 101 |
| PTMs | Variant #1: none Variant #2: Deamidation+Q(5) |

  

Protein Q99878

| Name | H2A1J\_HUMAN | | |
| Description | Histone H2A type 1 J OS Homo sapiens GN HIST1H2AJ PE 1 SV 3 | | |
| Sequence | ```   1 MSGRGKQGGK ARAKAKTRSS RAGLQFPVGR VHRLLRKGNY AERVGAGAPV YLAAVLEYLT AEILELAGNA ARDNKKTRII PRHLQLAIRN DEELNKLLGK 101 VTIAQGGVLP NIQAVLLPKK TESHHKTK ``` | | |
| Evidence | Indistinguishable | | |
| Peptide list | 298\*\*, 551\*\*, 1055\* | | |
| Peptides | 298\*\* | Confidence | Green |
| Runs | 1, 2, 3, 4 |
| Relation | NonDiscriminating |
| Proteins | H2A1A\_HUMAN, H2AX\_HUMAN, H2A1J\_HUMAN, H2A2A\_HUMAN, H2A3\_HUMAN, H2A1B\_HUMAN, H2A2C\_HUMAN, H2A1C\_HUMAN, H2AJ\_HUMAN, H2A1\_HUMAN, H2A1D\_HUMAN, H2A1H\_HUMAN, H2AJ\_HUMAN, H2AZ\_HUMAN, H2AV\_HUMAN |
| Sequence | ``` HLQLAIR ``` |
| Position | 83 |
| PTMs | none |
| 551\*\* | Confidence | Green |
| Runs | 1, 2, 3, 4 |
| Relation | NonDiscriminating |
| Proteins | H2A1A\_HUMAN, H2AX\_HUMAN, H2A1J\_HUMAN, H2A2A\_HUMAN, H2A3\_HUMAN, H2A1B\_HUMAN, H2A2C\_HUMAN, H2A1C\_HUMAN, H2AJ\_HUMAN, H2A1\_HUMAN, H2A1D\_HUMAN, H2A1H\_HUMAN, H2A2B\_HUMAN, H2AJ\_HUMAN, H2AZ\_HUMAN, H2AV\_HUMAN |
| Sequence | ``` AGLQFPVGR ``` |
| Position | 22 |
| PTMs | none |
| 1055\* | Confidence | Green |
| Runs | 1, 2, 3, 4 |
| Relation | Discriminating |
| Proteins | H2A1A\_HUMAN, H2AX\_HUMAN, H2A1J\_HUMAN, H2A2A\_HUMAN, H2A3\_HUMAN, H2A1B\_HUMAN, H2A2C\_HUMAN, H2A1C\_HUMAN, H2AJ\_HUMAN, H2A1\_HUMAN, H2A1D\_HUMAN, H2A1H\_HUMAN, H2A2B\_HUMAN |
| Sequence | ``` VTIAQGGVLPNIQAVLLPK ``` |
| Position | 101 |
| PTMs | Variant #1: none Variant #2: Deamidation+Q(5) |

  

Protein Q6FI13

| Name | H2A2A\_HUMAN | | |
| Description | Histone H2A type 2 A OS Homo sapiens GN HIST2H2AA3 PE 1 SV 3 | | |
| Sequence | ```   1 MSGRGKQGGK ARAKAKSRSS RAGLQFPVGR VHRLLRKGNY AERVGAGAPV YMAAVLEYLT AEILELAGNA ARDNKKTRII PRHLQLAIRN DEELNKLLGK 101 VTIAQGGVLP NIQAVLLPKK TESHHKAKGK ``` | | |
| Evidence | Indistinguishable | | |
| Peptide list | 298\*\*, 551\*\*, 1055\* | | |
| Peptides | 298\*\* | Confidence | Green |
| Runs | 1, 2, 3, 4 |
| Relation | NonDiscriminating |
| Proteins | H2A1A\_HUMAN, H2AX\_HUMAN, H2A1J\_HUMAN, H2A2A\_HUMAN, H2A3\_HUMAN, H2A1B\_HUMAN, H2A2C\_HUMAN, H2A1C\_HUMAN, H2AJ\_HUMAN, H2A1\_HUMAN, H2A1D\_HUMAN, H2A1H\_HUMAN, H2AJ\_HUMAN, H2AZ\_HUMAN, H2AV\_HUMAN |
| Sequence | ``` HLQLAIR ``` |
| Position | 83 |
| PTMs | none |
| 551\*\* | Confidence | Green |
| Runs | 1, 2, 3, 4 |
| Relation | NonDiscriminating |
| Proteins | H2A1A\_HUMAN, H2AX\_HUMAN, H2A1J\_HUMAN, H2A2A\_HUMAN, H2A3\_HUMAN, H2A1B\_HUMAN, H2A2C\_HUMAN, H2A1C\_HUMAN, H2AJ\_HUMAN, H2A1\_HUMAN, H2A1D\_HUMAN, H2A1H\_HUMAN, H2A2B\_HUMAN, H2AJ\_HUMAN, H2AZ\_HUMAN, H2AV\_HUMAN |
| Sequence | ``` AGLQFPVGR ``` |
| Position | 22 |
| PTMs | none |
| 1055\* | Confidence | Green |
| Runs | 1, 2, 3, 4 |
| Relation | Discriminating |
| Proteins | H2A1A\_HUMAN, H2AX\_HUMAN, H2A1J\_HUMAN, H2A2A\_HUMAN, H2A3\_HUMAN, H2A1B\_HUMAN, H2A2C\_HUMAN, H2A1C\_HUMAN, H2AJ\_HUMAN, H2A1\_HUMAN, H2A1D\_HUMAN, H2A1H\_HUMAN, H2A2B\_HUMAN |
| Sequence | ``` VTIAQGGVLPNIQAVLLPK ``` |
| Position | 101 |
| PTMs | Variant #1: none Variant #2: Deamidation+Q(5) |

  

Protein Q7L7L0

| Name | H2A3\_HUMAN | | |
| Description | Histone H2A type 3 OS Homo sapiens GN HIST3H2A PE 1 SV 3 | | |
| Sequence | ```   1 MSGRGKQGGK ARAKAKSRSS RAGLQFPVGR VHRLLRKGNY SERVGAGAPV YLAAVLEYLT AEILELAGNA ARDNKKTRII PRHLQLAIRN DEELNKLLGR 101 VTIAQGGVLP NIQAVLLPKK TESHHKAKGK ``` | | |
| Evidence | Indistinguishable | | |
| Peptide list | 298\*\*, 551\*\*, 1055\* | | |
| Peptides | 298\*\* | Confidence | Green |
| Runs | 1, 2, 3, 4 |
| Relation | NonDiscriminating |
| Proteins | H2A1A\_HUMAN, H2AX\_HUMAN, H2A1J\_HUMAN, H2A2A\_HUMAN, H2A3\_HUMAN, H2A1B\_HUMAN, H2A2C\_HUMAN, H2A1C\_HUMAN, H2AJ\_HUMAN, H2A1\_HUMAN, H2A1D\_HUMAN, H2A1H\_HUMAN, H2AJ\_HUMAN, H2AZ\_HUMAN, H2AV\_HUMAN |
| Sequence | ``` HLQLAIR ``` |
| Position | 83 |
| PTMs | none |
| 551\*\* | Confidence | Green |
| Runs | 1, 2, 3, 4 |
| Relation | NonDiscriminating |
| Proteins | H2A1A\_HUMAN, H2AX\_HUMAN, H2A1J\_HUMAN, H2A2A\_HUMAN, H2A3\_HUMAN, H2A1B\_HUMAN, H2A2C\_HUMAN, H2A1C\_HUMAN, H2AJ\_HUMAN, H2A1\_HUMAN, H2A1D\_HUMAN, H2A1H\_HUMAN, H2A2B\_HUMAN, H2AJ\_HUMAN, H2AZ\_HUMAN, H2AV\_HUMAN |
| Sequence | ``` AGLQFPVGR ``` |
| Position | 22 |
| PTMs | none |
| 1055\* | Confidence | Green |
| Runs | 1, 2, 3, 4 |
| Relation | Discriminating |
| Proteins | H2A1A\_HUMAN, H2AX\_HUMAN, H2A1J\_HUMAN, H2A2A\_HUMAN, H2A3\_HUMAN, H2A1B\_HUMAN, H2A2C\_HUMAN, H2A1C\_HUMAN, H2AJ\_HUMAN, H2A1\_HUMAN, H2A1D\_HUMAN, H2A1H\_HUMAN, H2A2B\_HUMAN |
| Sequence | ``` VTIAQGGVLPNIQAVLLPK ``` |
| Position | 101 |
| PTMs | Variant #1: none Variant #2: Deamidation+Q(5) |

  

Protein P04908

| Name | H2A1B\_HUMAN | | |
| Description | Histone H2A type 1 B E OS Homo sapiens GN HIST1H2AB PE 1 SV 2 | | |
| Sequence | ```   1 MSGRGKQGGK ARAKAKTRSS RAGLQFPVGR VHRLLRKGNY SERVGAGAPV YLAAVLEYLT AEILELAGNA ARDNKKTRII PRHLQLAIRN DEELNKLLGR 101 VTIAQGGVLP NIQAVLLPKK TESHHKAKGK ``` | | |
| Evidence | Indistinguishable | | |
| Peptide list | 298\*\*, 551\*\*, 1055\* | | |
| Peptides | 298\*\* | Confidence | Green |
| Runs | 1, 2, 3, 4 |
| Relation | NonDiscriminating |
| Proteins | H2A1A\_HUMAN, H2AX\_HUMAN, H2A1J\_HUMAN, H2A2A\_HUMAN, H2A3\_HUMAN, H2A1B\_HUMAN, H2A2C\_HUMAN, H2A1C\_HUMAN, H2AJ\_HUMAN, H2A1\_HUMAN, H2A1D\_HUMAN, H2A1H\_HUMAN, H2AJ\_HUMAN, H2AZ\_HUMAN, H2AV\_HUMAN |
| Sequence | ``` HLQLAIR ``` |
| Position | 83 |
| PTMs | none |
| 551\*\* | Confidence | Green |
| Runs | 1, 2, 3, 4 |
| Relation | NonDiscriminating |
| Proteins | H2A1A\_HUMAN, H2AX\_HUMAN, H2A1J\_HUMAN, H2A2A\_HUMAN, H2A3\_HUMAN, H2A1B\_HUMAN, H2A2C\_HUMAN, H2A1C\_HUMAN, H2AJ\_HUMAN, H2A1\_HUMAN, H2A1D\_HUMAN, H2A1H\_HUMAN, H2A2B\_HUMAN, H2AJ\_HUMAN, H2AZ\_HUMAN, H2AV\_HUMAN |
| Sequence | ``` AGLQFPVGR ``` |
| Position | 22 |
| PTMs | none |
| 1055\* | Confidence | Green |
| Runs | 1, 2, 3, 4 |
| Relation | Discriminating |
| Proteins | H2A1A\_HUMAN, H2AX\_HUMAN, H2A1J\_HUMAN, H2A2A\_HUMAN, H2A3\_HUMAN, H2A1B\_HUMAN, H2A2C\_HUMAN, H2A1C\_HUMAN, H2AJ\_HUMAN, H2A1\_HUMAN, H2A1D\_HUMAN, H2A1H\_HUMAN, H2A2B\_HUMAN |
| Sequence | ``` VTIAQGGVLPNIQAVLLPK ``` |
| Position | 101 |
| PTMs | Variant #1: none Variant #2: Deamidation+Q(5) |

  

Protein Q16777

| Name | H2A2C\_HUMAN | | |
| Description | Histone H2A type 2 C OS Homo sapiens GN HIST2H2AC PE 1 SV 4 | | |
| Sequence | ```   1 MSGRGKQGGK ARAKAKSRSS RAGLQFPVGR VHRLLRKGNY AERVGAGAPV YMAAVLEYLT AEILELAGNA ARDNKKTRII PRHLQLAIRN DEELNKLLGK 101 VTIAQGGVLP NIQAVLLPKK TESHKAKSK ``` | | |
| Evidence | Indistinguishable | | |
| Peptide list | 298\*\*, 551\*\*, 1055\* | | |
| Peptides | 298\*\* | Confidence | Green |
| Runs | 1, 2, 3, 4 |
| Relation | NonDiscriminating |
| Proteins | H2A1A\_HUMAN, H2AX\_HUMAN, H2A1J\_HUMAN, H2A2A\_HUMAN, H2A3\_HUMAN, H2A1B\_HUMAN, H2A2C\_HUMAN, H2A1C\_HUMAN, H2AJ\_HUMAN, H2A1\_HUMAN, H2A1D\_HUMAN, H2A1H\_HUMAN, H2AJ\_HUMAN, H2AZ\_HUMAN, H2AV\_HUMAN |
| Sequence | ``` HLQLAIR ``` |
| Position | 83 |
| PTMs | none |
| 551\*\* | Confidence | Green |
| Runs | 1, 2, 3, 4 |
| Relation | NonDiscriminating |
| Proteins | H2A1A\_HUMAN, H2AX\_HUMAN, H2A1J\_HUMAN, H2A2A\_HUMAN, H2A3\_HUMAN, H2A1B\_HUMAN, H2A2C\_HUMAN, H2A1C\_HUMAN, H2AJ\_HUMAN, H2A1\_HUMAN, H2A1D\_HUMAN, H2A1H\_HUMAN, H2A2B\_HUMAN, H2AJ\_HUMAN, H2AZ\_HUMAN, H2AV\_HUMAN |
| Sequence | ``` AGLQFPVGR ``` |
| Position | 22 |
| PTMs | none |
| 1055\* | Confidence | Green |
| Runs | 1, 2, 3, 4 |
| Relation | Discriminating |
| Proteins | H2A1A\_HUMAN, H2AX\_HUMAN, H2A1J\_HUMAN, H2A2A\_HUMAN, H2A3\_HUMAN, H2A1B\_HUMAN, H2A2C\_HUMAN, H2A1C\_HUMAN, H2AJ\_HUMAN, H2A1\_HUMAN, H2A1D\_HUMAN, H2A1H\_HUMAN, H2A2B\_HUMAN |
| Sequence | ``` VTIAQGGVLPNIQAVLLPK ``` |
| Position | 101 |
| PTMs | Variant #1: none Variant #2: Deamidation+Q(5) |

  

Protein Q93077

| Name | H2A1C\_HUMAN | | |
| Description | Histone H2A type 1 C OS Homo sapiens GN HIST1H2AC PE 1 SV 3 | | |
| Sequence | ```   1 MSGRGKQGGK ARAKAKSRSS RAGLQFPVGR VHRLLRKGNY AERVGAGAPV YLAAVLEYLT AEILELAGNA ARDNKKTRII PRHLQLAIRN DEELNKLLGR 101 VTIAQGGVLP NIQAVLLPKK TESHHKAKGK ``` | | |
| Evidence | Indistinguishable | | |
| Peptide list | 298\*\*, 551\*\*, 1055\* | | |
| Peptides | 298\*\* | Confidence | Green |
| Runs | 1, 2, 3, 4 |
| Relation | NonDiscriminating |
| Proteins | H2A1A\_HUMAN, H2AX\_HUMAN, H2A1J\_HUMAN, H2A2A\_HUMAN, H2A3\_HUMAN, H2A1B\_HUMAN, H2A2C\_HUMAN, H2A1C\_HUMAN, H2AJ\_HUMAN, H2A1\_HUMAN, H2A1D\_HUMAN, H2A1H\_HUMAN, H2AJ\_HUMAN, H2AZ\_HUMAN, H2AV\_HUMAN |
| Sequence | ``` HLQLAIR ``` |
| Position | 83 |
| PTMs | none |
| 551\*\* | Confidence | Green |
| Runs | 1, 2, 3, 4 |
| Relation | NonDiscriminating |
| Proteins | H2A1A\_HUMAN, H2AX\_HUMAN, H2A1J\_HUMAN, H2A2A\_HUMAN, H2A3\_HUMAN, H2A1B\_HUMAN, H2A2C\_HUMAN, H2A1C\_HUMAN, H2AJ\_HUMAN, H2A1\_HUMAN, H2A1D\_HUMAN, H2A1H\_HUMAN, H2A2B\_HUMAN, H2AJ\_HUMAN, H2AZ\_HUMAN, H2AV\_HUMAN |
| Sequence | ``` AGLQFPVGR ``` |
| Position | 22 |
| PTMs | none |
| 1055\* | Confidence | Green |
| Runs | 1, 2, 3, 4 |
| Relation | Discriminating |
| Proteins | H2A1A\_HUMAN, H2AX\_HUMAN, H2A1J\_HUMAN, H2A2A\_HUMAN, H2A3\_HUMAN, H2A1B\_HUMAN, H2A2C\_HUMAN, H2A1C\_HUMAN, H2AJ\_HUMAN, H2A1\_HUMAN, H2A1D\_HUMAN, H2A1H\_HUMAN, H2A2B\_HUMAN |
| Sequence | ``` VTIAQGGVLPNIQAVLLPK ``` |
| Position | 101 |
| PTMs | Variant #1: none Variant #2: Deamidation+Q(5) |

  

Protein Q9BTM1

| Name | H2AJ\_HUMAN | | |
| Description | Histone H2A J OS Homo sapiens GN H2AFJ PE 1 SV 1 | | |
| Sequence | ```   1 MSGRGKQGGK VRAKAKSRSS RAGLQFPVGR VHRLLRKGNY AERVGAGAPV YLAAVLEYLT AEILELAGNA ARDNKKTRII PRHLQLAIRN DEELNKLLGK 101 VTIAQGGVLP NIQAVLLPKK TESQKTKSK ``` | | |
| Evidence | Indistinguishable | | |
| Peptide list | 298\*\*, 551\*\*, 1055\* | | |
| Peptides | 298\*\* | Confidence | Green |
| Runs | 1, 2, 3, 4 |
| Relation | NonDiscriminating |
| Proteins | H2A1A\_HUMAN, H2AX\_HUMAN, H2A1J\_HUMAN, H2A2A\_HUMAN, H2A3\_HUMAN, H2A1B\_HUMAN, H2A2C\_HUMAN, H2A1C\_HUMAN, H2AJ\_HUMAN, H2A1\_HUMAN, H2A1D\_HUMAN, H2A1H\_HUMAN, H2AJ\_HUMAN, H2AZ\_HUMAN, H2AV\_HUMAN |
| Sequence | ``` HLQLAIR ``` |
| Position | 83 |
| PTMs | none |
| 551\*\* | Confidence | Green |
| Runs | 1, 2, 3, 4 |
| Relation | NonDiscriminating |
| Proteins | H2A1A\_HUMAN, H2AX\_HUMAN, H2A1J\_HUMAN, H2A2A\_HUMAN, H2A3\_HUMAN, H2A1B\_HUMAN, H2A2C\_HUMAN, H2A1C\_HUMAN, H2AJ\_HUMAN, H2A1\_HUMAN, H2A1D\_HUMAN, H2A1H\_HUMAN, H2A2B\_HUMAN, H2AJ\_HUMAN, H2AZ\_HUMAN, H2AV\_HUMAN |
| Sequence | ``` AGLQFPVGR ``` |
| Position | 22 |
| PTMs | none |
| 1055\* | Confidence | Green |
| Runs | 1, 2, 3, 4 |
| Relation | Discriminating |
| Proteins | H2A1A\_HUMAN, H2AX\_HUMAN, H2A1J\_HUMAN, H2A2A\_HUMAN, H2A3\_HUMAN, H2A1B\_HUMAN, H2A2C\_HUMAN, H2A1C\_HUMAN, H2AJ\_HUMAN, H2A1\_HUMAN, H2A1D\_HUMAN, H2A1H\_HUMAN, H2A2B\_HUMAN |
| Sequence | ``` VTIAQGGVLPNIQAVLLPK ``` |
| Position | 101 |
| PTMs | Variant #1: none Variant #2: Deamidation+Q(5) |

  

Protein P0C0S8

| Name | H2A1\_HUMAN | | |
| Description | Histone H2A type 1 OS Homo sapiens GN HIST1H2AG PE 1 SV 2 | | |
| Sequence | ```   1 MSGRGKQGGK ARAKAKTRSS RAGLQFPVGR VHRLLRKGNY AERVGAGAPV YLAAVLEYLT AEILELAGNA ARDNKKTRII PRHLQLAIRN DEELNKLLGK 101 VTIAQGGVLP NIQAVLLPKK TESHHKAKGK ``` | | |
| Evidence | Indistinguishable | | |
| Peptide list | 298\*\*, 551\*\*, 1055\* | | |
| Peptides | 298\*\* | Confidence | Green |
| Runs | 1, 2, 3, 4 |
| Relation | NonDiscriminating |
| Proteins | H2A1A\_HUMAN, H2AX\_HUMAN, H2A1J\_HUMAN, H2A2A\_HUMAN, H2A3\_HUMAN, H2A1B\_HUMAN, H2A2C\_HUMAN, H2A1C\_HUMAN, H2AJ\_HUMAN, H2A1\_HUMAN, H2A1D\_HUMAN, H2A1H\_HUMAN, H2AJ\_HUMAN, H2AZ\_HUMAN, H2AV\_HUMAN |
| Sequence | ``` HLQLAIR ``` |
| Position | 83 |
| PTMs | none |
| 551\*\* | Confidence | Green |
| Runs | 1, 2, 3, 4 |
| Relation | NonDiscriminating |
| Proteins | H2A1A\_HUMAN, H2AX\_HUMAN, H2A1J\_HUMAN, H2A2A\_HUMAN, H2A3\_HUMAN, H2A1B\_HUMAN, H2A2C\_HUMAN, H2A1C\_HUMAN, H2AJ\_HUMAN, H2A1\_HUMAN, H2A1D\_HUMAN, H2A1H\_HUMAN, H2A2B\_HUMAN, H2AJ\_HUMAN, H2AZ\_HUMAN, H2AV\_HUMAN |
| Sequence | ``` AGLQFPVGR ``` |
| Position | 22 |
| PTMs | none |
| 1055\* | Confidence | Green |
| Runs | 1, 2, 3, 4 |
| Relation | Discriminating |
| Proteins | H2A1A\_HUMAN, H2AX\_HUMAN, H2A1J\_HUMAN, H2A2A\_HUMAN, H2A3\_HUMAN, H2A1B\_HUMAN, H2A2C\_HUMAN, H2A1C\_HUMAN, H2AJ\_HUMAN, H2A1\_HUMAN, H2A1D\_HUMAN, H2A1H\_HUMAN, H2A2B\_HUMAN |
| Sequence | ``` VTIAQGGVLPNIQAVLLPK ``` |
| Position | 101 |
| PTMs | Variant #1: none Variant #2: Deamidation+Q(5) |

  

Protein P20671

| Name | H2A1D\_HUMAN | | |
| Description | Histone H2A type 1 D OS Homo sapiens GN HIST1H2AD PE 1 SV 2 | | |
| Sequence | ```   1 MSGRGKQGGK ARAKAKTRSS RAGLQFPVGR VHRLLRKGNY SERVGAGAPV YLAAVLEYLT AEILELAGNA ARDNKKTRII PRHLQLAIRN DEELNKLLGK 101 VTIAQGGVLP NIQAVLLPKK TESHHKAKGK ``` | | |
| Evidence | Indistinguishable | | |
| Peptide list | 298\*\*, 551\*\*, 1055\* | | |
| Peptides | 298\*\* | Confidence | Green |
| Runs | 1, 2, 3, 4 |
| Relation | NonDiscriminating |
| Proteins | H2A1A\_HUMAN, H2AX\_HUMAN, H2A1J\_HUMAN, H2A2A\_HUMAN, H2A3\_HUMAN, H2A1B\_HUMAN, H2A2C\_HUMAN, H2A1C\_HUMAN, H2AJ\_HUMAN, H2A1\_HUMAN, H2A1D\_HUMAN, H2A1H\_HUMAN, H2AJ\_HUMAN, H2AZ\_HUMAN, H2AV\_HUMAN |
| Sequence | ``` HLQLAIR ``` |
| Position | 83 |
| PTMs | none |
| 551\*\* | Confidence | Green |
| Runs | 1, 2, 3, 4 |
| Relation | NonDiscriminating |
| Proteins | H2A1A\_HUMAN, H2AX\_HUMAN, H2A1J\_HUMAN, H2A2A\_HUMAN, H2A3\_HUMAN, H2A1B\_HUMAN, H2A2C\_HUMAN, H2A1C\_HUMAN, H2AJ\_HUMAN, H2A1\_HUMAN, H2A1D\_HUMAN, H2A1H\_HUMAN, H2A2B\_HUMAN, H2AJ\_HUMAN, H2AZ\_HUMAN, H2AV\_HUMAN |
| Sequence | ``` AGLQFPVGR ``` |
| Position | 22 |
| PTMs | none |
| 1055\* | Confidence | Green |
| Runs | 1, 2, 3, 4 |
| Relation | Discriminating |
| Proteins | H2A1A\_HUMAN, H2AX\_HUMAN, H2A1J\_HUMAN, H2A2A\_HUMAN, H2A3\_HUMAN, H2A1B\_HUMAN, H2A2C\_HUMAN, H2A1C\_HUMAN, H2AJ\_HUMAN, H2A1\_HUMAN, H2A1D\_HUMAN, H2A1H\_HUMAN, H2A2B\_HUMAN |
| Sequence | ``` VTIAQGGVLPNIQAVLLPK ``` |
| Position | 101 |
| PTMs | Variant #1: none Variant #2: Deamidation+Q(5) |

  

Protein Q96KK5

| Name | H2A1H\_HUMAN | | |
| Description | Histone H2A type 1 H OS Homo sapiens GN HIST1H2AH PE 1 SV 3 | | |
| Sequence | ```   1 MSGRGKQGGK ARAKAKTRSS RAGLQFPVGR VHRLLRKGNY AERVGAGAPV YLAAVLEYLT AEILELAGNA ARDNKKTRII PRHLQLAIRN DEELNKLLGK 101 VTIAQGGVLP NIQAVLLPKK TESHHKAK ``` | | |
| Evidence | Indistinguishable | | |
| Peptide list | 298\*\*, 551\*\*, 1055\* | | |
| Peptides | 298\*\* | Confidence | Green |
| Runs | 1, 2, 3, 4 |
| Relation | NonDiscriminating |
| Proteins | H2A1A\_HUMAN, H2AX\_HUMAN, H2A1J\_HUMAN, H2A2A\_HUMAN, H2A3\_HUMAN, H2A1B\_HUMAN, H2A2C\_HUMAN, H2A1C\_HUMAN, H2AJ\_HUMAN, H2A1\_HUMAN, H2A1D\_HUMAN, H2A1H\_HUMAN, H2AJ\_HUMAN, H2AZ\_HUMAN, H2AV\_HUMAN |
| Sequence | ``` HLQLAIR ``` |
| Position | 83 |
| PTMs | none |
| 551\*\* | Confidence | Green |
| Runs | 1, 2, 3, 4 |
| Relation | NonDiscriminating |
| Proteins | H2A1A\_HUMAN, H2AX\_HUMAN, H2A1J\_HUMAN, H2A2A\_HUMAN, H2A3\_HUMAN, H2A1B\_HUMAN, H2A2C\_HUMAN, H2A1C\_HUMAN, H2AJ\_HUMAN, H2A1\_HUMAN, H2A1D\_HUMAN, H2A1H\_HUMAN, H2A2B\_HUMAN, H2AJ\_HUMAN, H2AZ\_HUMAN, H2AV\_HUMAN |
| Sequence | ``` AGLQFPVGR ``` |
| Position | 22 |
| PTMs | none |
| 1055\* | Confidence | Green |
| Runs | 1, 2, 3, 4 |
| Relation | Discriminating |
| Proteins | H2A1A\_HUMAN, H2AX\_HUMAN, H2A1J\_HUMAN, H2A2A\_HUMAN, H2A3\_HUMAN, H2A1B\_HUMAN, H2A2C\_HUMAN, H2A1C\_HUMAN, H2AJ\_HUMAN, H2A1\_HUMAN, H2A1D\_HUMAN, H2A1H\_HUMAN, H2A2B\_HUMAN |
| Sequence | ``` VTIAQGGVLPNIQAVLLPK ``` |
| Position | 101 |
| PTMs | Variant #1: none Variant #2: Deamidation+Q(5) |

  

Protein Q8IUE6

| Name | H2A2B\_HUMAN | | |
| Description | Histone H2A type 2 B OS Homo sapiens GN HIST2H2AB PE 1 SV 3 | | |
| Sequence | ```   1 MSGRGKQGGK ARAKAKSRSS RAGLQFPVGR VHRLLRKGNY AERVGAGAPV YLAAVLEYLT AEILELAGNA ARDNKKTRII PRHLQLAVRN DEELNKLLGG 101 VTIAQGGVLP NIQAVLLPKK TESHKPGKNK ``` | | |
| Evidence | Indistinguishable | | |
| Peptide list | 551\*\*, 1055\* | | |
| Peptides | 551\*\* | Confidence | Green |
| Runs | 1, 2, 3, 4 |
| Relation | NonDiscriminating |
| Proteins | H2A1A\_HUMAN, H2AX\_HUMAN, H2A1J\_HUMAN, H2A2A\_HUMAN, H2A3\_HUMAN, H2A1B\_HUMAN, H2A2C\_HUMAN, H2A1C\_HUMAN, H2AJ\_HUMAN, H2A1\_HUMAN, H2A1D\_HUMAN, H2A1H\_HUMAN, H2A2B\_HUMAN, H2AJ\_HUMAN, H2AZ\_HUMAN, H2AV\_HUMAN |
| Sequence | ``` AGLQFPVGR ``` |
| Position | 22 |
| PTMs | none |
| 1055\* | Confidence | Green |
| Runs | 1, 2, 3, 4 |
| Relation | Discriminating |
| Proteins | H2A1A\_HUMAN, H2AX\_HUMAN, H2A1J\_HUMAN, H2A2A\_HUMAN, H2A3\_HUMAN, H2A1B\_HUMAN, H2A2C\_HUMAN, H2A1C\_HUMAN, H2AJ\_HUMAN, H2A1\_HUMAN, H2A1D\_HUMAN, H2A1H\_HUMAN, H2A2B\_HUMAN |
| Sequence | ``` VTIAQGGVLPNIQAVLLPK ``` |
| Position | 101 |
| PTMs | Variant #1: none Variant #2: Deamidation+Q(5) |

  

Protein P06748

| Name | NPM\_HUMAN | | |
| Description | Nucleophosmin OS Homo sapiens GN NPM1 PE 1 SV 2 | | |
| Sequence | ```   1 MEDSMDMDMS PLRPQNYLFG CELKADKDYH FKVDNDENEH QLSLRTVSLG AGAKDELHIV EAEAMNYEGS PIKVTLATLK MSVQPTVSLG GFEITPPVVL 101 RLKCGSGPVH ISGQHLVAVE EDAESEDEEE EDVKLLSISG KRSAPGGGSK VPQKKVKLAA DEDDDDDDEE DDDEDDDDDD FDDEEAEEKA PVKKSIRDTP 201 AKNAQKSNQN GKDSKPSSTP RSKGQESFKK QEKTPKTPKG PSSVEDIKAK MQASIEKGGS LPKVEAKFIN YVKNCFRMTD QEAIQDLWQW RKSL ``` | | |
| Evidence | Indistinguishable | | |
| Peptide list | 71\*, 124\*, 195\*, 336\*, 856\*, 1073\* | | |
| Peptides | 71\* | Confidence | Green |
| Runs | 1, 2, 3, 4 |
| Relation | Discriminating |
| Proteins | NPM\_HUMAN, NPM\_HUMAN |
| Sequence | ``` TVSLGAGAK ``` |
| Position | 46 |
| PTMs | none |
| 124\* | Confidence | Green |
| Runs | 1, 2, 3, 4 |
| Relation | Discriminating |
| Proteins | NPM\_HUMAN, NPM\_HUMAN |
| Sequence | ``` GPSSVEDIK ``` |
| Position | 240 |
| PTMs | none |
| 195\* | Confidence | Green |
| Runs | 1, 2, 3, 4 |
| Relation | Discriminating |
| Proteins | NPM\_HUMAN, NPM\_HUMAN |
| Sequence | ``` VDNDENEHQLSLR ``` |
| Position | 33 |
| PTMs | Variant #1: none Variant #2: Deamidation+N(3) |
| 336\* | Confidence | Green |
| Runs | 1, 2, 3, 4 |
| Relation | Discriminating |
| Proteins | NPM\_HUMAN, NPM\_HUMAN |
| Sequence | ``` VTLATLK ``` |
| Position | 74 |
| PTMs | none |
| 856\* | Confidence | Green |
| Runs | 1, 2, 4 |
| Relation | Discriminating |
| Proteins | NPM\_HUMAN, NPM\_HUMAN |
| Sequence | ``` DELHIVEAEAMNYEGSPIK ``` |
| Position | 55 |
| PTMs | none |
| 1073\* | Confidence | Green |
| Runs | 1, 2, 3, 4 |
| Relation | Discriminating |
| Proteins | NPM\_HUMAN, NPM\_HUMAN |
| Sequence | ``` MSVQPTVSLGGFEITPPVVLR ``` |
| Position | 81 |
| PTMs | Variant #1: none Variant #2: Oxidation+M(1) |

  

Protein P06748-2

| Name | NPM\_HUMAN | | |
| Description | Isoform 2 of Nucleophosmin OS Homo sapiens GN NPM1 | | |
| Sequence | ```   1 MEDSMDMDMS PLRPQNYLFG CELKADKDYH FKVDNDENEH QLSLRTVSLG AGAKDELHIV EAEAMNYEGS PIKVTLATLK MSVQPTVSLG GFEITPPVVL 101 RLKCGSGPVH ISGQHLVAVE EDAESEDEEE EDVKLLSISG KRSAPGGGSK VPQKKVKLAA DEDDDDDDEE DDDEDDDDDD FDDEEAEEKA PVKKGQESFK 201 KQEKTPKTPK GPSSVEDIKA KMQASIEKGG SLPKVEAKFI NYVKNCFRMT DQEAIQDLWQ WRKSL ``` | | |
| Evidence | Indistinguishable | | |
| Peptide list | 71\*, 124\*, 195\*, 336\*, 856\*, 1073\* | | |
| Peptides | 71\* | Confidence | Green |
| Runs | 1, 2, 3, 4 |
| Relation | Discriminating |
| Proteins | NPM\_HUMAN, NPM\_HUMAN |
| Sequence | ``` TVSLGAGAK ``` |
| Position | 46 |
| PTMs | none |
| 124\* | Confidence | Green |
| Runs | 1, 2, 3, 4 |
| Relation | Discriminating |
| Proteins | NPM\_HUMAN, NPM\_HUMAN |
| Sequence | ``` GPSSVEDIK ``` |
| Position | 211 |
| PTMs | none |
| 195\* | Confidence | Green |
| Runs | 1, 2, 3, 4 |
| Relation | Discriminating |
| Proteins | NPM\_HUMAN, NPM\_HUMAN |
| Sequence | ``` VDNDENEHQLSLR ``` |
| Position | 33 |
| PTMs | Variant #1: none Variant #2: Deamidation+N(3) |
| 336\* | Confidence | Green |
| Runs | 1, 2, 3, 4 |
| Relation | Discriminating |
| Proteins | NPM\_HUMAN, NPM\_HUMAN |
| Sequence | ``` VTLATLK ``` |
| Position | 74 |
| PTMs | none |
| 856\* | Confidence | Green |
| Runs | 1, 2, 4 |
| Relation | Discriminating |
| Proteins | NPM\_HUMAN, NPM\_HUMAN |
| Sequence | ``` DELHIVEAEAMNYEGSPIK ``` |
| Position | 55 |
| PTMs | none |
| 1073\* | Confidence | Green |
| Runs | 1, 2, 3, 4 |
| Relation | Discriminating |
| Proteins | NPM\_HUMAN, NPM\_HUMAN |
| Sequence | ``` MSVQPTVSLGGFEITPPVVLR ``` |
| Position | 81 |
| PTMs | Variant #1: none Variant #2: Oxidation+M(1) |

  

Protein Q00839

| Name | HNRPU\_HUMAN | | |
| Description | Heterogeneous nuclear ribonucleoprotein U OS Homo sapiens GN HNRNPU PE 1 SV 6 | | |
| Sequence | ```   1 MSSSPVNVKK LKVSELKEEL KKRRLSDKGL KAELMERLQA ALDDEEAGGR PAMEPGNGSL DLGGDSAGRS GAGLEQEAAA GGDEEEEEEE EEEEGISALD 101 GDQMELGEEN GAAGAADSGP MEEEEAASED ENGDDQGFQE GEDELGDEEE GAGDENGHGE QQPQPPATQQ QQPQQQRGAA KEAAGKSSGP TSLFAVTVAP 201 PGARQGQQQA GGKKKAEGGG GGGRPGAPAA GDGKTEQKGG DKKRGVKRPR EDHGRGYFEY IEENKYSRAK SPQPPVEEED EHFDDTVVCL DTYNCDLHFK 301 ISRDRLSASS LTMESFAFLW AGGRASYGVS KGKVCFEMKV TEKIPVRHLY TKDIDIHEVR IGWSLTTSGM LLGEEEFSYG YSLKGIKTCN CETEDYGEKF 401 DENDVITCFA NFESDEVELS YAKNGQDLGV AFKISKEVLA GRPLFPHVLC HNCAVEFNFG QKEKPYFPIP EEYTFIQNVP LEDRVRGPKG PEEKKDCEVV 501 MMIGLPGAGK TTWVTKHAAE NPGKYNILGT NTIMDKMMVA GFKKQMADTG KLNTLLQRAP QCLGKFIEIA ARKKRNFILD QTNVSAAAQR RKMCLFAGFQ 601 RKAVVVCPKD EDYKQRTQKK AEVEGKDLPE HAVLKMKGNF TLPEVAECFD EITYVELQKE EAQKLLEQYK EESKKALPPE KKQNTGSKKS NKNKSGKNQF 701 NRGGGHRGRG GFNMRGGNFR GGAPGNRGGY NRRGNMPQRG GGGGGSGGIG YPYPRAPVFP GRGSYSNRGN YNRGGMPNRG NYNQNFRGRG NNRGYKNQSQ 801 GYNQWQQGQF WGQKPWSQHY HQGYY ``` | | |
| Evidence | Indistinguishable | | |
| Peptide list | 248\*, 392\*, 610\*, 814\*, 1008\*, 1277\* | | |
| Peptides | 248\* | Confidence | Green |
| Runs | 1, 2, 3, 4 |
| Relation | Discriminating |
| Proteins | HNRPU\_HUMAN, HNRPU\_HUMAN |
| Sequence | ``` DLPEHAVLK ``` |
| Position | 627 |
| PTMs | none |
| 392\* | Confidence | Green |
| Runs | 1, 2, 3, 4 |
| Relation | Discriminating |
| Proteins | HNRPU\_HUMAN, HNRPU\_HUMAN |
| Sequence | ``` FIEIAAR ``` |
| Position | 566 |
| PTMs | none |
| 610\* | Confidence | Green |
| Runs | 1, 2, 4 |
| Relation | Discriminating |
| Proteins | HNRPU\_HUMAN, HNRPU\_HUMAN |
| Sequence | ``` NFILDQTNVSAAAQR ``` |
| Position | 576 |
| PTMs | Variant #1: none Variant #2: Deamidation+N(8) Deamidation+Q(6) |
| 814\* | Confidence | Green |
| Runs | 1, 2, 3, 4 |
| Relation | Discriminating |
| Proteins | HNRPU\_HUMAN, HNRPU\_HUMAN |
| Sequence | ``` SSGPTSLFAVTVAPPGAR ``` |
| Position | 187 |
| PTMs | none |
| 1008\* | Confidence | Green |
| Runs | 1, 2, 3, 4 |
| Relation | Discriminating |
| Proteins | HNRPU\_HUMAN, HNRPU\_HUMAN |
| Sequence | ``` EKPYFPIPEEYTFIQNVPLEDR ``` |
| Position | 463 |
| PTMs | none |
| 1277\* | Confidence | Green |
| Runs | 2, 3, 4 |
| Relation | Discriminating |
| Proteins | HNRPU\_HUMAN, HNRPU\_HUMAN |
| Sequence | ``` DIDIHEVR ``` |
| Position | 353 |
| PTMs | none |

  

Protein Q00839-2

| Name | HNRPU\_HUMAN | | |
| Description | Isoform Short of Heterogeneous nuclear ribonucleoprotein U OS Homo sapiens GN HNRNPU | | |
| Sequence | ```   1 MSSSPVNVKK LKVSELKEEL KKRRLSDKGL KAELMERLQA ALDDEEAGGR PAMEPGNGSL DLGGDSAGRS GAGLEQEAAA GGDEEEEEEE EEEEGISALD 101 GDQMELGEEN GAAGAADSGP MEEEEAASED ENGDDQGFQE GEDELGDEEE GAGDENGHGE QQPQPPATQQ QQPQQQRGAA KEAAGKSSGP TSLFAVTVAP 201 PGARQGQQQA GGDGKTEQKG GDKKRGVKRP REDHGRGYFE YIEENKYSRA KSPQPPVEEE DEHFDDTVVC LDTYNCDLHF KISRDRLSAS SLTMESFAFL 301 WAGGRASYGV SKGKVCFEMK VTEKIPVRHL YTKDIDIHEV RIGWSLTTSG MLLGEEEFSY GYSLKGIKTC NCETEDYGEK FDENDVITCF ANFESDEVEL 401 SYAKNGQDLG VAFKISKEVL AGRPLFPHVL CHNCAVEFNF GQKEKPYFPI PEEYTFIQNV PLEDRVRGPK GPEEKKDCEV VMMIGLPGAG KTTWVTKHAA 501 ENPGKYNILG TNTIMDKMMV AGFKKQMADT GKLNTLLQRA PQCLGKFIEI AARKKRNFIL DQTNVSAAAQ RRKMCLFAGF QRKAVVVCPK DEDYKQRTQK 601 KAEVEGKDLP EHAVLKMKGN FTLPEVAECF DEITYVELQK EEAQKLLEQY KEESKKALPP EKKQNTGSKK SNKNKSGKNQ FNRGGGHRGR GGFNMRGGNF 701 RGGAPGNRGG YNRRGNMPQR GGGGGGSGGI GYPYPRAPVF PGRGSYSNRG NYNRGGMPNR GNYNQNFRGR GNNRGYKNQS QGYNQWQQGQ FWGQKPWSQH 801 YHQGYY ``` | | |
| Evidence | Indistinguishable | | |
| Peptide list | 248\*, 392\*, 610\*, 814\*, 1008\*, 1277\* | | |
| Peptides | 248\* | Confidence | Green |
| Runs | 1, 2, 3, 4 |
| Relation | Discriminating |
| Proteins | HNRPU\_HUMAN, HNRPU\_HUMAN |
| Sequence | ``` DLPEHAVLK ``` |
| Position | 608 |
| PTMs | none |
| 392\* | Confidence | Green |
| Runs | 1, 2, 3, 4 |
| Relation | Discriminating |
| Proteins | HNRPU\_HUMAN, HNRPU\_HUMAN |
| Sequence | ``` FIEIAAR ``` |
| Position | 547 |
| PTMs | none |
| 610\* | Confidence | Green |
| Runs | 1, 2, 4 |
| Relation | Discriminating |
| Proteins | HNRPU\_HUMAN, HNRPU\_HUMAN |
| Sequence | ``` NFILDQTNVSAAAQR ``` |
| Position | 557 |
| PTMs | Variant #1: none Variant #2: Deamidation+N(8) Deamidation+Q(6) |
| 814\* | Confidence | Green |
| Runs | 1, 2, 3, 4 |
| Relation | Discriminating |
| Proteins | HNRPU\_HUMAN, HNRPU\_HUMAN |
| Sequence | ``` SSGPTSLFAVTVAPPGAR ``` |
| Position | 187 |
| PTMs | none |
| 1008\* | Confidence | Green |
| Runs | 1, 2, 3, 4 |
| Relation | Discriminating |
| Proteins | HNRPU\_HUMAN, HNRPU\_HUMAN |
| Sequence | ``` EKPYFPIPEEYTFIQNVPLEDR ``` |
| Position | 444 |
| PTMs | none |
| 1277\* | Confidence | Green |
| Runs | 2, 3, 4 |
| Relation | Discriminating |
| Proteins | HNRPU\_HUMAN, HNRPU\_HUMAN |
| Sequence | ``` DIDIHEVR ``` |
| Position | 334 |
| PTMs | none |

  

Protein P07910

| Name | HNRPC\_HUMAN | | |
| Description | Heterogeneous nuclear ribonucleoproteins C1 C2 OS Homo sapiens GN HNRNPC PE 1 SV 4 | | |
| Sequence | ```   1 MASNVTNKTD PRSMNSRVFI GNLNTLVVKK SDVEAIFSKY GKIVGCSVHK GFAFVQYVNE RNARAAVAGE DGRMIAGQVL DINLAAEPKV NRGKAGVKRS 101 AAEMYGSVTE HPSPSPLLSS SFDLDYDFQR DYYDRMYSYP ARVPPPPPIA RAVVPSKRQR VSGNTSRRGK SGFNSKSGQR GSSKSGKLKG DDLQAIKKEL 201 TQIKQKVDSL LENLEKIEKE QSKQAVEMKN DKSEEEQSSS SVKKDETNVK MESEGGADDS AEEGDLLDDD DNEDRGDDQL ELIKDDEKEA EEGEDDRDSA 301 NGEDDS ``` | | |
| Evidence | Indistinguishable | | |
| Peptide list | 415\*\*, 692\*\*, 752\*, 835\*\* | | |
| Peptides | 415\*\* | Confidence | Green |
| Runs | 1, 2, 3 |
| Relation | NonDiscriminating |
| Proteins | HNRPC\_HUMAN, HNRPC\_HUMAN, HNRPC\_HUMAN, HNRCL\_HUMAN |
| Sequence | ``` KSDVEAIFSK ``` |
| Position | 30 |
| PTMs | none |
| 692\*\* | Confidence | Green |
| Runs | 1, 2, 4 |
| Relation | NonDiscriminating |
| Proteins | HNRPC\_HUMAN, HNRPC\_HUMAN, HNRPC\_HUMAN, HNRCL\_HUMAN, HNRPC\_HUMAN |
| Sequence | ``` VDSLLENLEK ``` |
| Position | 207 |
| PTMs | none |
| 752\* | Confidence | Green |
| Runs | 1, 2, 3 |
| Relation | Discriminating |
| Proteins | HNRPC\_HUMAN, HNRPC\_HUMAN, HNRPC\_HUMAN |
| Sequence | ``` GFAFVQYVNER ``` |
| Position | 51 |
| PTMs | none |
| 835\*\* | Confidence | Green |
| Runs | 1, 2, 3, 4 |
| Relation | NonDiscriminating |
| Proteins | HNRPC\_HUMAN, HNRPC\_HUMAN, HNRPC\_HUMAN, HNRCL\_HUMAN, HNRPC\_HUMAN |
| Sequence | ``` VFIGNLNTLVVK ``` |
| Position | 18 |
| PTMs | none |

  

Protein P07910-4

| Name | HNRPC\_HUMAN | | |
| Description | Isoform 4 of Heterogeneous nuclear ribonucleoproteins C1 C2 OS Homo sapiens GN HNRNPC | | |
| Sequence | ```   1 MASNVTNKTD PRSMNSRVFI GNLNTLVVKK SDVEAIFSKY GKIVGCSVHK GFAFVQYVNE RNARAAVAGE DGRMIAGQVL DINLAAEPKV NRGKAGVKRS 101 AAEMYGSSFD LDYDFQRDYY DRMYSYPARV PPPPPIARAI KKELTQIKQK VDSLLENLEK IEKEQSKQAV EMKNDKSEEE QSSSSVKKDE TNVKMESEGG 201 ADDSAEEGDL LDDDDNEDRG DDQLELIKDD EKEAEEGEDD RDSANGEDDS ``` | | |
| Evidence | Indistinguishable | | |
| Peptide list | 415\*\*, 692\*\*, 752\*, 835\*\* | | |
| Peptides | 415\*\* | Confidence | Green |
| Runs | 1, 2, 3 |
| Relation | NonDiscriminating |
| Proteins | HNRPC\_HUMAN, HNRPC\_HUMAN, HNRPC\_HUMAN, HNRCL\_HUMAN |
| Sequence | ``` KSDVEAIFSK ``` |
| Position | 30 |
| PTMs | none |
| 692\*\* | Confidence | Green |
| Runs | 1, 2, 4 |
| Relation | NonDiscriminating |
| Proteins | HNRPC\_HUMAN, HNRPC\_HUMAN, HNRPC\_HUMAN, HNRCL\_HUMAN, HNRPC\_HUMAN |
| Sequence | ``` VDSLLENLEK ``` |
| Position | 151 |
| PTMs | none |
| 752\* | Confidence | Green |
| Runs | 1, 2, 3 |
| Relation | Discriminating |
| Proteins | HNRPC\_HUMAN, HNRPC\_HUMAN, HNRPC\_HUMAN |
| Sequence | ``` GFAFVQYVNER ``` |
| Position | 51 |
| PTMs | none |
| 835\*\* | Confidence | Green |
| Runs | 1, 2, 3, 4 |
| Relation | NonDiscriminating |
| Proteins | HNRPC\_HUMAN, HNRPC\_HUMAN, HNRPC\_HUMAN, HNRCL\_HUMAN, HNRPC\_HUMAN |
| Sequence | ``` VFIGNLNTLVVK ``` |
| Position | 18 |
| PTMs | none |

  

Protein P07910-2

| Name | HNRPC\_HUMAN | | |
| Description | Isoform C1 of Heterogeneous nuclear ribonucleoproteins C1 C2 OS Homo sapiens GN HNRNPC | | |
| Sequence | ```   1 MASNVTNKTD PRSMNSRVFI GNLNTLVVKK SDVEAIFSKY GKIVGCSVHK GFAFVQYVNE RNARAAVAGE DGRMIAGQVL DINLAAEPKV NRGKAGVKRS 101 AAEMYGSSFD LDYDFQRDYY DRMYSYPARV PPPPPIARAV VPSKRQRVSG NTSRRGKSGF NSKSGQRGSS KSGKLKGDDL QAIKKELTQI KQKVDSLLEN 201 LEKIEKEQSK QAVEMKNDKS EEEQSSSSVK KDETNVKMES EGGADDSAEE GDLLDDDDNE DRGDDQLELI KDDEKEAEEG EDDRDSANGE DDS ``` | | |
| Evidence | Indistinguishable | | |
| Peptide list | 415\*\*, 692\*\*, 752\*, 835\*\* | | |
| Peptides | 415\*\* | Confidence | Green |
| Runs | 1, 2, 3 |
| Relation | NonDiscriminating |
| Proteins | HNRPC\_HUMAN, HNRPC\_HUMAN, HNRPC\_HUMAN, HNRCL\_HUMAN |
| Sequence | ``` KSDVEAIFSK ``` |
| Position | 30 |
| PTMs | none |
| 692\*\* | Confidence | Green |
| Runs | 1, 2, 4 |
| Relation | NonDiscriminating |
| Proteins | HNRPC\_HUMAN, HNRPC\_HUMAN, HNRPC\_HUMAN, HNRCL\_HUMAN, HNRPC\_HUMAN |
| Sequence | ``` VDSLLENLEK ``` |
| Position | 194 |
| PTMs | none |
| 752\* | Confidence | Green |
| Runs | 1, 2, 3 |
| Relation | Discriminating |
| Proteins | HNRPC\_HUMAN, HNRPC\_HUMAN, HNRPC\_HUMAN |
| Sequence | ``` GFAFVQYVNER ``` |
| Position | 51 |
| PTMs | none |
| 835\*\* | Confidence | Green |
| Runs | 1, 2, 3, 4 |
| Relation | NonDiscriminating |
| Proteins | HNRPC\_HUMAN, HNRPC\_HUMAN, HNRPC\_HUMAN, HNRCL\_HUMAN, HNRPC\_HUMAN |
| Sequence | ``` VFIGNLNTLVVK ``` |
| Position | 18 |
| PTMs | none |

  

Protein Q14103

| Name | HNRPD\_HUMAN | | |
| Description | Heterogeneous nuclear ribonucleoprotein D0 OS Homo sapiens GN HNRNPD PE 1 SV 1 | | |
| Sequence | ```   1 MSEEQFGGDG AAAAATAAVG GSAGEQEGAM VAATQGAAAA AGSGAGTGGG TASGGTEGGS AESEGAKIDA SKNEEDEGHS NSSPRHSEAA TAQREEWKMF 101 IGGLSWDTTK KDLKDYFSKF GEVVDCTLKL DPITGRSRGF GFVLFKESES VDKVMDQKEH KLNGKVIDPK RAKAMKTKEP VKKIFVGGLS PDTPEEKIRE 201 YFGGFGEVES IELPMDNKTN KRRGFCFITF KEEEPVKKIM EKKYHNVGLS KCEIKVAMSK EQYQQQQQWG SRGGFAGRAR GRGGGPSQNW NQGYSNYWNQ 301 GYGNYGYNSQ GYGGYGGYDY TGYNNYYGYG DYSNQQSGYG KVSRRGGHQN SYKPY ``` | | |
| Evidence | Indistinguishable | | |
| Peptide list | 563\*, 887\*, 1629\*\* | | |
| Peptides | 563\* | Confidence | Green |
| Runs | 1, 2, 4 |
| Relation | Discriminating |
| Proteins | HNRPD\_HUMAN, HNRPD\_HUMAN, HNRPD\_HUMAN, HNRPD\_HUMAN |
| Sequence | ``` IFVGGLSPDTPEEK ``` |
| Position | 184 |
| PTMs | none |
| 887\* | Confidence | Green |
| Runs | 1, 2, 3, 4 |
| Relation | Discriminating |
| Proteins | HNRPD\_HUMAN, HNRPD\_HUMAN, HNRPD\_HUMAN, HNRPD\_HUMAN |
| Sequence | ``` GFCFITFK ``` |
| Position | 224 |
| PTMs | Carbamidomethyl+C(3) |
| 1629\*\* | Confidence | Green |
| Runs | 2, 3, 4 |
| Relation | NonDiscriminating |
| Proteins | HNRPD\_HUMAN, HNRPD\_HUMAN, HNRPD\_HUMAN, HNRPD\_HUMAN, HNRDL\_HUMAN, HNRDL\_HUMAN, HNRDL\_HUMAN |
| Sequence | ``` GFGFVLFK ``` |
| Position | 139 |
| PTMs | none |

  

Protein Q14103-4

| Name | HNRPD\_HUMAN | | |
| Description | Isoform 4 of Heterogeneous nuclear ribonucleoprotein D0 OS Homo sapiens GN HNRNPD | | |
| Sequence | ```   1 MSEEQFGGDG AAAAATAAVG GSAGEQEGAM VAATQGAAAA AGSGAGTGGG TASGGTEGGS AESEGAKIDA SKNEEDEGKM FIGGLSWDTT KKDLKDYFSK 101 FGEVVDCTLK LDPITGRSRG FGFVLFKESE SVDKVMDQKE HKLNGKVIDP KRAKAMKTKE PVKKIFVGGL SPDTPEEKIR EYFGGFGEVE SIELPMDNKT 201 NKRRGFCFIT FKEEEPVKKI MEKKYHNVGL SKCEIKVAMS KEQYQQQQQW GSRGGFAGRA RGRGGDQQSG YGKVSRRGGH QNSYKPY ``` | | |
| Evidence | Indistinguishable | | |
| Peptide list | 563\*, 887\*, 1629\*\* | | |
| Peptides | 563\* | Confidence | Green |
| Runs | 1, 2, 4 |
| Relation | Discriminating |
| Proteins | HNRPD\_HUMAN, HNRPD\_HUMAN, HNRPD\_HUMAN, HNRPD\_HUMAN |
| Sequence | ``` IFVGGLSPDTPEEK ``` |
| Position | 165 |
| PTMs | none |
| 887\* | Confidence | Green |
| Runs | 1, 2, 3, 4 |
| Relation | Discriminating |
| Proteins | HNRPD\_HUMAN, HNRPD\_HUMAN, HNRPD\_HUMAN, HNRPD\_HUMAN |
| Sequence | ``` GFCFITFK ``` |
| Position | 205 |
| PTMs | Carbamidomethyl+C(3) |
| 1629\*\* | Confidence | Green |
| Runs | 2, 3, 4 |
| Relation | NonDiscriminating |
| Proteins | HNRPD\_HUMAN, HNRPD\_HUMAN, HNRPD\_HUMAN, HNRPD\_HUMAN, HNRDL\_HUMAN, HNRDL\_HUMAN, HNRDL\_HUMAN |
| Sequence | ``` GFGFVLFK ``` |
| Position | 120 |
| PTMs | none |

  

Protein Q14103-3

| Name | HNRPD\_HUMAN | | |
| Description | Isoform 3 of Heterogeneous nuclear ribonucleoprotein D0 OS Homo sapiens GN HNRNPD | | |
| Sequence | ```   1 MSEEQFGGDG AAAAATAAVG GSAGEQEGAM VAATQGAAAA AGSGAGTGGG TASGGTEGGS AESEGAKIDA SKNEEDEGHS NSSPRHSEAA TAQREEWKMF 101 IGGLSWDTTK KDLKDYFSKF GEVVDCTLKL DPITGRSRGF GFVLFKESES VDKVMDQKEH KLNGKVIDPK RAKAMKTKEP VKKIFVGGLS PDTPEEKIRE 201 YFGGFGEVES IELPMDNKTN KRRGFCFITF KEEEPVKKIM EKKYHNVGLS KCEIKVAMSK EQYQQQQQWG SRGGFAGRAR GRGGDQQSGY GKVSRRGGHQ 301 NSYKPY ``` | | |
| Evidence | Indistinguishable | | |
| Peptide list | 563\*, 887\*, 1629\*\* | | |
| Peptides | 563\* | Confidence | Green |
| Runs | 1, 2, 4 |
| Relation | Discriminating |
| Proteins | HNRPD\_HUMAN, HNRPD\_HUMAN, HNRPD\_HUMAN, HNRPD\_HUMAN |
| Sequence | ``` IFVGGLSPDTPEEK ``` |
| Position | 184 |
| PTMs | none |
| 887\* | Confidence | Green |
| Runs | 1, 2, 3, 4 |
| Relation | Discriminating |
| Proteins | HNRPD\_HUMAN, HNRPD\_HUMAN, HNRPD\_HUMAN, HNRPD\_HUMAN |
| Sequence | ``` GFCFITFK ``` |
| Position | 224 |
| PTMs | Carbamidomethyl+C(3) |
| 1629\*\* | Confidence | Green |
| Runs | 2, 3, 4 |
| Relation | NonDiscriminating |
| Proteins | HNRPD\_HUMAN, HNRPD\_HUMAN, HNRPD\_HUMAN, HNRPD\_HUMAN, HNRDL\_HUMAN, HNRDL\_HUMAN, HNRDL\_HUMAN |
| Sequence | ``` GFGFVLFK ``` |
| Position | 139 |
| PTMs | none |

  

Protein Q14103-2

| Name | HNRPD\_HUMAN | | |
| Description | Isoform 2 of Heterogeneous nuclear ribonucleoprotein D0 OS Homo sapiens GN HNRNPD | | |
| Sequence | ```   1 MSEEQFGGDG AAAAATAAVG GSAGEQEGAM VAATQGAAAA AGSGAGTGGG TASGGTEGGS AESEGAKIDA SKNEEDEGKM FIGGLSWDTT KKDLKDYFSK 101 FGEVVDCTLK LDPITGRSRG FGFVLFKESE SVDKVMDQKE HKLNGKVIDP KRAKAMKTKE PVKKIFVGGL SPDTPEEKIR EYFGGFGEVE SIELPMDNKT 201 NKRRGFCFIT FKEEEPVKKI MEKKYHNVGL SKCEIKVAMS KEQYQQQQQW GSRGGFAGRA RGRGGGPSQN WNQGYSNYWN QGYGNYGYNS QGYGGYGGYD 301 YTGYNNYYGY GDYSNQQSGY GKVSRRGGHQ NSYKPY ``` | | |
| Evidence | Indistinguishable | | |
| Peptide list | 563\*, 887\*, 1629\*\* | | |
| Peptides | 563\* | Confidence | Green |
| Runs | 1, 2, 4 |
| Relation | Discriminating |
| Proteins | HNRPD\_HUMAN, HNRPD\_HUMAN, HNRPD\_HUMAN, HNRPD\_HUMAN |
| Sequence | ``` IFVGGLSPDTPEEK ``` |
| Position | 165 |
| PTMs | none |
| 887\* | Confidence | Green |
| Runs | 1, 2, 3, 4 |
| Relation | Discriminating |
| Proteins | HNRPD\_HUMAN, HNRPD\_HUMAN, HNRPD\_HUMAN, HNRPD\_HUMAN |
| Sequence | ``` GFCFITFK ``` |
| Position | 205 |
| PTMs | Carbamidomethyl+C(3) |
| 1629\*\* | Confidence | Green |
| Runs | 2, 3, 4 |
| Relation | NonDiscriminating |
| Proteins | HNRPD\_HUMAN, HNRPD\_HUMAN, HNRPD\_HUMAN, HNRPD\_HUMAN, HNRDL\_HUMAN, HNRDL\_HUMAN, HNRDL\_HUMAN |
| Sequence | ``` GFGFVLFK ``` |
| Position | 120 |
| PTMs | none |

  

Protein P49368

| Name | TCPG\_HUMAN | | |
| Description | T complex protein 1 subunit gamma OS Homo sapiens GN CCT3 PE 1 SV 4 | | |
| Sequence | ```   1 MMGHRPVLVL SQNTKRESGR KVQSGNINAA KTIADIIRTC LGPKSMMKML LDPMGGIVMT NDGNAILREI QVQHPAAKSM IEISRTQDEE VGDGTTSVII 101 LAGEMLSVAE HFLEQQMHPT VVISAYRKAL DDMISTLKKI SIPVDISDSD MMLNIINSSI TTKAISRWSS LACNIALDAV KMVQFEENGR KEIDIKKYAR 201 VEKIPGGIIE DSCVLRGVMI NKDVTHPRMR RYIKNPRIVL LDSSLEYKKG ESQTDIEITR EEDFTRILQM EEEYIQQLCE DIIQLKPDVV ITEKGISDLA 301 QHYLMRANIT AIRRVRKTDN NRIARACGAR IVSRPEELRE DDVGTGAGLL EIKKIGDEYF TFITDCKDPK ACTILLRGAS KEILSEVERN LQDAMQVCRN 401 VLLDPQLVPG GGASEMAVAH ALTEKSKAMT GVEQWPYRAV AQALEVIPRT LIQNCGASTI RLLTSLRAKH TQENCETWGV NGETGTLVDM KELGIWEPLA 501 VKLQTYKTAV ETAVLLLRID DIVSGHKKKG DDQSRQGGAP DAGQE ``` | | |
| Evidence | Indistinguishable | | |
| Peptide list | 631\*, 854\*, 969\* | | |
| Peptides | 631\* | Confidence | Green |
| Runs | 1, 2, 3, 4 |
| Relation | Discriminating |
| Proteins | TCPG\_HUMAN, TCPG\_HUMAN |
| Sequence | ``` AVAQALEVIPR ``` |
| Position | 439 |
| PTMs | none |
| 854\* | Confidence | Green |
| Runs | 1, 2, 3, 4 |
| Relation | Discriminating |
| Proteins | TCPG\_HUMAN, TCPG\_HUMAN |
| Sequence | ``` TAVETAVLLLR ``` |
| Position | 508 |
| PTMs | none |
| 969\* | Confidence | Green |
| Runs | 1, 2, 3, 4 |
| Relation | Discriminating |
| Proteins | TCPG\_HUMAN, TCPG\_HUMAN |
| Sequence | ``` NVLLDPQLVPGGGASEMAVAHALTEK ``` |
| Position | 400 |
| PTMs | none |

  

Protein P49368-2

| Name | TCPG\_HUMAN | | |
| Description | Isoform 2 of T complex protein 1 subunit gamma OS Homo sapiens GN CCT3 | | |
| Sequence | ```   1 MMGHRPVLVL SQNTKRESGR KVQSGNINAA KIQVQHPAAK SMIEISRTQD EEVGDGTTSV IILAGEMLSV AEHFLEQQMH PTVVISAYRK ALDDMISTLK 101 KISIPVDISD SDMMLNIINS SITTKAISRW SSLACNIALD AVKMVQFEEN GRKEIDIKKY ARVEKIPGGI IEDSCVLRGV MINKDVTHPR MRRYIKNPRI 201 VLLDSSLEYK KGESQTDIEI TREEDFTRIL QMEEEYIQQL CEDIIQLKPD VVITEKGISD LAQHYLMRAN ITAIRRVRKT DNNRIARACG ARIVSRPEEL 301 REDDVGTGAG LLEIKKIGDE YFTFITDCKD PKACTILLRG ASKEILSEVE RNLQDAMQVC RNVLLDPQLV PGGGASEMAV AHALTEKSKA MTGVEQWPYR 401 AVAQALEVIP RTLIQNCGAS TIRLLTSLRA KHTQENCETW GVNGETGTLV DMKELGIWEP LAVKLQTYKT AVETAVLLLR IDDIVSGHKK KGDDQSRQGG 501 APDAGQE ``` | | |
| Evidence | Indistinguishable | | |
| Peptide list | 631\*, 854\*, 969\* | | |
| Peptides | 631\* | Confidence | Green |
| Runs | 1, 2, 3, 4 |
| Relation | Discriminating |
| Proteins | TCPG\_HUMAN, TCPG\_HUMAN |
| Sequence | ``` AVAQALEVIPR ``` |
| Position | 401 |
| PTMs | none |
| 854\* | Confidence | Green |
| Runs | 1, 2, 3, 4 |
| Relation | Discriminating |
| Proteins | TCPG\_HUMAN, TCPG\_HUMAN |
| Sequence | ``` TAVETAVLLLR ``` |
| Position | 470 |
| PTMs | none |
| 969\* | Confidence | Green |
| Runs | 1, 2, 3, 4 |
| Relation | Discriminating |
| Proteins | TCPG\_HUMAN, TCPG\_HUMAN |
| Sequence | ``` NVLLDPQLVPGGGASEMAVAHALTEK ``` |
| Position | 362 |
| PTMs | none |

  

Protein P52272

| Name | HNRPM\_HUMAN | | |
| Description | Heterogeneous nuclear ribonucleoprotein M OS Homo sapiens GN HNRNPM PE 1 SV 3 | | |
| Sequence | ```   1 MAAGVEAAAE VAATEIKMEE ESGAPGVPSG NGAPGPKGEG ERPAQNEKRK EKNIKRGGNR FEPYANPTKR YRAFITNIPF DVKWQSLKDL VKEKVGEVTY 101 VELLMDAEGK SRGCAVVEFK MEESMKKAAE VLNKHSLSGR PLKVKEDPDG EHARRAMQKV MATTGGMGMG PGGPGMITIP PSILNNPNIP NEIIHALQAG 201 RLGSTVFVAN LDYKVGWKKL KEVFSMAGVV VRADILEDKD GKSRGIGTVT FEQSIEAVQA ISMFNGQLLF DRPMHVKMDE RALPKGDFFP PERPQQLPHG 301 LGGIGMGLGP GGQPIDANHL NKGIGMGNIG PAGMGMEGIG FGINKMGGME GPFGGGMENM GRFGSGMNMG RINEILSNAL KRGEIIAKQG GGGGGGSVPG 401 IERMGPGIDR LGGAGMERMG AGLGHGMDRV GSEIERMGLV MDRMGSVERM GSGIERMGPL GLDHMASSIE RMGQTMERIG SGVERMGAGM GFGLERMAAP 501 IDRVGQTIER MGSGVERMGP AIERMGLSME RMVPAGMGAG LERMGPVMDR MATGLERMGA NNLERMGLER MGANSLERMG LERMGANSLE RMGPAMGPAL 601 GAGIERMGLA MGGGGGASFD RAIEMERGNF GGSFAGSFGG AGGHAPGVAR KACQIFVRNL PFDFTWKMLK DKFNECGHVL YADIKMENGK SKGCGVVKFE 701 SPEVAERACR MMNGMKLSGR EIDVRIDRNA ``` | | |
| Evidence | Indistinguishable | | |
| Peptide list | 220\*, 886\*, 999\* | | |
| Peptides | 220\* | Confidence | Green |
| Runs | 1, 2, 3 |
| Relation | Discriminating |
| Proteins | HNRPM\_HUMAN, HNRPM\_HUMAN |
| Sequence | ``` FEPYANPTK ``` |
| Position | 61 |
| PTMs | none |
| 886\* | Confidence | Green |
| Runs | 1, 2, 3 |
| Relation | Discriminating |
| Proteins | HNRPM\_HUMAN, HNRPM\_HUMAN |
| Sequence | ``` AFITNIPFDVK ``` |
| Position | 73 |
| PTMs | none |
| 999\* | Confidence | Green |
| Runs | 1, 2, 3 |
| Relation | Discriminating |
| Proteins | HNRPM\_HUMAN, HNRPM\_HUMAN |
| Sequence | ``` AAGVEAAAEVAATEIK ``` |
| Position | 2 |
| PTMs | Acetyl+N-TERM(1) |

  

Protein P52272-2

| Name | HNRPM\_HUMAN | | |
| Description | Isoform 2 of Heterogeneous nuclear ribonucleoprotein M OS Homo sapiens GN HNRNPM | | |
| Sequence | ```   1 MAAGVEAAAE VAATEIKMEE ESGAPGVPSG NGAPGPKGEG ERPAQNEKRK EKNIKRGGNR FEPYANPTKR YRAFITNIPF DVKWQSLKDL VKEKVGEVTY 101 VELLMDAEGK SRGCAVVEFK MEESMKKAAE VLNKHSLSGR PLKVKEDPDG EHARRAMQKA GRLGSTVFVA NLDYKVGWKK LKEVFSMAGV VVRADILEDK 201 DGKSRGIGTV TFEQSIEAVQ AISMFNGQLL FDRPMHVKMD ERALPKGDFF PPERPQQLPH GLGGIGMGLG PGGQPIDANH LNKGIGMGNI GPAGMGMEGI 301 GFGINKMGGM EGPFGGGMEN MGRFGSGMNM GRINEILSNA LKRGEIIAKQ GGGGGGGSVP GIERMGPGID RLGGAGMERM GAGLGHGMDR VGSEIERMGL 401 VMDRMGSVER MGSGIERMGP LGLDHMASSI ERMGQTMERI GSGVERMGAG MGFGLERMAA PIDRVGQTIE RMGSGVERMG PAIERMGLSM ERMVPAGMGA 501 GLERMGPVMD RMATGLERMG ANNLERMGLE RMGANSLERM GLERMGANSL ERMGPAMGPA LGAGIERMGL AMGGGGGASF DRAIEMERGN FGGSFAGSFG 601 GAGGHAPGVA RKACQIFVRN LPFDFTWKML KDKFNECGHV LYADIKMENG KSKGCGVVKF ESPEVAERAC RMMNGMKLSG REIDVRIDRN A ``` | | |
| Evidence | Indistinguishable | | |
| Peptide list | 220\*, 886\*, 999\* | | |
| Peptides | 220\* | Confidence | Green |
| Runs | 1, 2, 3 |
| Relation | Discriminating |
| Proteins | HNRPM\_HUMAN, HNRPM\_HUMAN |
| Sequence | ``` FEPYANPTK ``` |
| Position | 61 |
| PTMs | none |
| 886\* | Confidence | Green |
| Runs | 1, 2, 3 |
| Relation | Discriminating |
| Proteins | HNRPM\_HUMAN, HNRPM\_HUMAN |
| Sequence | ``` AFITNIPFDVK ``` |
| Position | 73 |
| PTMs | none |
| 999\* | Confidence | Green |
| Runs | 1, 2, 3 |
| Relation | Discriminating |
| Proteins | HNRPM\_HUMAN, HNRPM\_HUMAN |
| Sequence | ``` AAGVEAAAEVAATEIK ``` |
| Position | 2 |
| PTMs | Acetyl+N-TERM(1) |

  

Protein O00148

| Name | DX39A\_HUMAN | | |
| Description | ATP dependent RNA helicase DDX39A OS Homo sapiens GN DDX39A PE 1 SV 2 | | |
| Sequence | ```   1 MAEQDVENDL LDYDEEEEPQ APQESTPAPP KKDIKGSYVS IHSSGFRDFL LKPELLRAIV DCGFEHPSEV QHECIPQAIL GMDVLCQAKS GMGKTAVFVL 101 ATLQQIEPVN GQVTVLVMCH TRELAFQISK EYERFSKYMP SVKVSVFFGG LSIKKDEEVL KKNCPHVVVG TPGRILALVR NRSFSLKNVK HFVLDECDKM 201 LEQLDMRRDV QEIFRLTPHE KQCMMFSATL SKDIRPVCRK FMQDPMEVFV DDETKLTLHG LQQYYVKLKD SEKNRKLFDL LDVLEFNQVI IFVKSVQRCM 301 ALAQLLVEQN FPAIAIHRGM AQEERLSRYQ QFKDFQRRIL VATNLFGRGM DIERVNIVFN YDMPEDSDTY LHRVARAGRF GTKGLAITFV SDENDAKILN 401 DVQDRFEVNV AELPEEIDIS TYIEQSR ``` | | |
| Evidence | Indistinguishable | | |
| Peptide list | 333\*, 827\* | | |
| Peptides | 333\* | Confidence | Green |
| Runs | 1, 2, 4 |
| Relation | Discriminating |
| Proteins | DX39B\_HUMAN, DX39B\_HUMAN, DX39A\_HUMAN |
| Sequence | ``` GSYVSIHSSGFR ``` |
| Position | 36 |
| PTMs | none |
| 827\* | Confidence | Green |
| Runs | 1, 2, 3, 4 |
| Relation | Discriminating |
| Proteins | DX39B\_HUMAN, DX39B\_HUMAN, DX39A\_HUMAN |
| Sequence | ``` DFLLKPELLR ``` |
| Position | 48 |
| PTMs | none |

  

Protein Q13838

| Name | DX39B\_HUMAN | | |
| Description | Spliceosome RNA helicase DDX39B OS Homo sapiens GN DDX39B PE 1 SV 1 | | |
| Sequence | ```   1 MAENDVDNEL LDYEDDEVET AAGGDGAEAP AKKDVKGSYV SIHSSGFRDF LLKPELLRAI VDCGFEHPSE VQHECIPQAI LGMDVLCQAK SGMGKTAVFV 101 LATLQQLEPV TGQVSVLVMC HTRELAFQIS KEYERFSKYM PNVKVAVFFG GLSIKKDEEV LKKNCPHIVV GTPGRILALA RNKSLNLKHI KHFILDECDK 201 MLEQLDMRRD VQEIFRMTPH EKQVMMFSAT LSKEIRPVCR KFMQDPMEIF VDDETKLTLH GLQQYYVKLK DNEKNRKLFD LLDVLEFNQV VIFVKSVQRC 301 IALAQLLVEQ NFPAIAIHRG MPQEERLSRY QQFKDFQRRI LVATNLFGRG MDIERVNIAF NYDMPEDSDT YLHRVARAGR FGTKGLAITF VSDENDAKIL 401 NDVQDRFEVN ISELPDEIDI SSYIEQTR ``` | | |
| Evidence | Indistinguishable | | |
| Peptide list | 333\*, 827\* | | |
| Peptides | 333\* | Confidence | Green |
| Runs | 1, 2, 4 |
| Relation | Discriminating |
| Proteins | DX39B\_HUMAN, DX39B\_HUMAN, DX39A\_HUMAN |
| Sequence | ``` GSYVSIHSSGFR ``` |
| Position | 37 |
| PTMs | none |
| 827\* | Confidence | Green |
| Runs | 1, 2, 3, 4 |
| Relation | Discriminating |
| Proteins | DX39B\_HUMAN, DX39B\_HUMAN, DX39A\_HUMAN |
| Sequence | ``` DFLLKPELLR ``` |
| Position | 49 |
| PTMs | none |

  

Protein Q13838-2

| Name | DX39B\_HUMAN | | |
| Description | Isoform 2 of Spliceosome RNA helicase DDX39B OS Homo sapiens GN DDX39B | | |
| Sequence | ```   1 MAENDVDNEL LDYEDDEVET AAGGDGAEAP AKKDVKGSYV SIHSSGFRDF LLKPELLRAI VDCGFEHPSE VQHECIPQAI LGMDVLCQAK SGMGKTAVFV 101 LATLQQLEPV TGQVYLGRVL GRGFWLGLVS VLVMCHTREL AFQISKEYER FSKYMPNVKV AVFFGGLSIK KDEEVLKKNC PHIVVGTPGR ILALARNKSL 201 NLKHIKHFIL DECDKMLEQL DMRRDVQEIF RMTPHEKQVM MFSATLSKEI RPVCRKFMQD PMEIFVDDET KLTLHGLQQY YVKLKDNEKN RKLFDLLDVL 301 EFNQVVIFVK SVQRCIALAQ LLVEQNFPAI AIHRGMPQEE RLSRYQQFKD FQRRILVATN LFGRGMDIER VNIAFNYDMP EDSDTYLHRV ARAGRFGTKG 401 LAITFVSDEN DAKILNDVQD RFEVNISELP DEIDISSYIE QTR ``` | | |
| Evidence | Indistinguishable | | |
| Peptide list | 333\*, 827\* | | |
| Peptides | 333\* | Confidence | Green |
| Runs | 1, 2, 4 |
| Relation | Discriminating |
| Proteins | DX39B\_HUMAN, DX39B\_HUMAN, DX39A\_HUMAN |
| Sequence | ``` GSYVSIHSSGFR ``` |
| Position | 37 |
| PTMs | none |
| 827\* | Confidence | Green |
| Runs | 1, 2, 3, 4 |
| Relation | Discriminating |
| Proteins | DX39B\_HUMAN, DX39B\_HUMAN, DX39A\_HUMAN |
| Sequence | ``` DFLLKPELLR ``` |
| Position | 49 |
| PTMs | none |

  

Protein Q15084

| Name | PDIA6\_HUMAN | | |
| Description | Protein disulfide isomerase A6 OS Homo sapiens GN PDIA6 PE 1 SV 1 | | |
| Sequence | ```   1 MALLVLGLVS CTFFLAVNGL YSSSDDVIEL TPSNFNREVI QSDSLWLVEF YAPWCGHCQR LTPEWKKAAT ALKDVVKVGA VDADKHHSLG GQYGVQGFPT 101 IKIFGSNKNR PEDYQGGRTG EAIVDAALSA LRQLVKDRLG GRSGGYSSGK QGRSDSSSKK DVIELTDDSF DKNVLDSEDV WMVEFYAPWC GHCKNLEPEW 201 AAAASEVKEQ TKGKVKLAAV DATVNQVLAS RYGIRGFPTI KIFQKGESPV DYDGGRTRSD IVSRALDLFS DNAPPPELLE IINEDIAKRT CEEHQLCVVA 301 VLPHILDTGA AGRNSYLEVL LKLADKYKKK MWGWLWTEAG AQSELETALG IGGFGYPAMA AINARKMKFA LLKGSFSEQG INEFLRELSF GRGSTAPVGG 401 GAFPTIVERE PWDGRDGELP VEDDIDLSDV ELDDLGKDEL ``` | | |
| Evidence | Indistinguishable | | |
| Peptide list | 1021\* | | |
| Peptides | 1021\* | Confidence | Green |
| Runs | 1, 2, 3, 4 |
| Relation | Discriminating |
| Proteins | PDIA6\_HUMAN, PDIA6\_HUMAN |
| Sequence | ``` TGEAIVDAALSALR ``` |
| Position | 119 |
| PTMs | none |

  

Protein Q15084-2

| Name | PDIA6\_HUMAN | | |
| Description | Isoform 2 of Protein disulfide isomerase A6 OS Homo sapiens GN PDIA6 | | |
| Sequence | ```   1 MRRDLREKLV WVCRPLAPVE VPANISSDFQ PCSPTSPAHS LSRKSPIMYP STTMANAPGL VSCTFFLAVN GLYSSSDDVI ELTPSNFNRE VIQSDSLWLV 101 EFYAPWCGHC QRLTPEWKKA ATALKDVVKV GAVDADKHHS LGGQYGVQGF PTIKIFGSNK NRPEDYQGGR TGEAIVDAAL SALRQLVKDR LGGRSGGYSS 201 GKQGRSDSSS KKDVIELTDD SFDKNVLDSE DVWMVEFYAP WCGHCKNLEP EWAAAASEVK EQTKGKVKLA AVDATVNQVL ASRYGIRGFP TIKIFQKGES 301 PVDYDGGRTR SDIVSRALDL FSDNAPPPEL LEIINEDIAK RTCEEHQLCV VAVLPHILDT GAAGRNSYLE VLLKLADKYK KKMWGWLWTE AGAQSELETA 401 LGIGGFGYPA MAAINARKMK FALLKGSFSE QGINEFLREL SFGRGSTAPV GGGAFPTIVE REPWDGRDGE LPVEDDIDLS DVELDDLGKD EL ``` | | |
| Evidence | Indistinguishable | | |
| Peptide list | 1021\* | | |
| Peptides | 1021\* | Confidence | Green |
| Runs | 1, 2, 3, 4 |
| Relation | Discriminating |
| Proteins | PDIA6\_HUMAN, PDIA6\_HUMAN |
| Sequence | ``` TGEAIVDAALSALR ``` |
| Position | 171 |
| PTMs | none |

  

Protein Q92841

| Name | DDX17\_HUMAN | | |
| Description | Probable ATP dependent RNA helicase DDX17 OS Homo sapiens GN DDX17 PE 1 SV 1 | | |
| Sequence | ```   1 MRGGGFGDRD RDRDRGGFGA RGGGGLPPKK FGNPGERLRK KKWDLSELPK FEKNFYVEHP EVARLTPYEV DELRRKKEIT VRGGDVCPKP VFAFHHANFP 101 QYVMDVLMDQ HFTEPTPIQC QGFPLALSGR DMVGIAQTGS GKTLAYLLPA IVHINHQPYL ERGDGPICLV LAPTRELAQQ VQQVADDYGK CSRLKSTCIY 201 GGAPKGPQIR DLERGVEICI ATPGRLIDFL ESGKTNLRRC TYLVLDEADR MLDMGFEPQI RKIVDQIRPD RQTLMWSATW PKEVRQLAED FLRDYTQINV 301 GNLELSANHN ILQIVDVCME SEKDHKLIQL MEEIMAEKEN KTIIFVETKR RCDDLTRRMR RDGWPAMCIH GDKSQPERDW VLNEFRSGKA PILIATDVAS 401 RGLDVEDVKF VINYDYPNSS EDYVHRIGRT ARSTNKGTAY TFFTPGNLKQ ARELIKVLEE ANQAINPKLM QLVDHRGGGG GGGGRSRYRT TSSANNPNLM 501 YQDECDRRLR GVKDGGRRDS ASYRDRSETD RAGYANGSGY GSPNSAFGAQ AGQYTYGQGT YGAAAYGTSS YTAQEYGAGT YGASSTTSTG RSSQSSSQQF 601 SGIGRSGQQP QPLMSQQFAQ PPGATNMIGY MGQTAYQYPP PPPPPPPSRK ``` | | |
| Evidence | Indistinguishable | | |
| Peptide list | 267\*, 575\*\* | | |
| Peptides | 267\* | Confidence | Yellow |
| Runs | 1, 2, 4 |
| Relation | Discriminating |
| Proteins | DDX17\_HUMAN, DDX17\_HUMAN, DDX17\_HUMAN |
| Sequence | ``` LMQLVDHRGGGGGGGGR ``` |
| Position | 469 |
| PTMs | Deamidation+Q(3) |
| 575\*\* | Confidence | Green |
| Runs | 1, 2, 3, 4 |
| Relation | NonDiscriminating |
| Proteins | DDX17\_HUMAN, DDX17\_HUMAN, DDX17\_HUMAN, DDX17\_HUMAN, DDX5\_HUMAN |
| Sequence | ``` APILIATDVASR ``` |
| Position | 390 |
| PTMs | none |

  

Protein Q92841-4

| Name | DDX17\_HUMAN | | |
| Description | Isoform 4 of Probable ATP dependent RNA helicase DDX17 OS Homo sapiens GN DDX17 | | |
| Sequence | ```   1 MPTGFVAPIL CVLLPSPTRE AATVASATGD SASERESAAP AAAPTAEAPP PSVVTRPEPQ ALPSPAIRAP LPDLYPFGTM RGGGFGDRDR DRDRGGFGAR 101 GGGGLPPKKF GNPGERLRKK KWDLSELPKF EKNFYVEHPE VARLTPYEVD ELRRKKEITV RGGDVCPKPV FAFHHANFPQ YVMDVLMDQH FTEPTPIQCQ 201 GFPLALSGRD MVGIAQTGSG KTLAYLLPAI VHINHQPYLE RGDGPICLVL APTRELAQQV QQVADDYGKC SRLKSTCIYG GAPKGPQIRD LERGVEICIA 301 TPGRLIDFLE SGKTNLRRCT YLVLDEADRM LDMGFEPQIR KIVDQIRPDR QTLMWSATWP KEVRQLAEDF LRDYTQINVG NLELSANHNI LQIVDVCMES 401 EKDHKLIQLM EEIMAEKENK TIIFVETKRR CDDLTRRMRR DGWPAMCIHG DKSQPERDWV LNEFRSGKAP ILIATDVASR GLDVEDVKFV INYDYPNSSE 501 DYVHRIGRTA RSTNKGTAYT FFTPGNLKQA RELIKVLEEA NQAINPKLMQ LVDHRGGGGG GGGRSRYRTT SSANNPNLMY QDECDRRLRG VKDGGRRDSA 601 SYRDRSETDR AGYANGSGYG SPNSAFGAQA GQYTYGQGTY GAAAYGTSSY TAQEYGAGTY GASSTTSTGR SSQSSSQQFS GIGRSGQQPQ PLMSQQFAQP 701 PGATNMIGYM GQTAYQYPPP PPPPPPSRK ``` | | |
| Evidence | Indistinguishable | | |
| Peptide list | 267\*, 575\*\* | | |
| Peptides | 267\* | Confidence | Yellow |
| Runs | 1, 2, 4 |
| Relation | Discriminating |
| Proteins | DDX17\_HUMAN, DDX17\_HUMAN, DDX17\_HUMAN |
| Sequence | ``` LMQLVDHRGGGGGGGGR ``` |
| Position | 548 |
| PTMs | Deamidation+Q(3) |
| 575\*\* | Confidence | Green |
| Runs | 1, 2, 3, 4 |
| Relation | NonDiscriminating |
| Proteins | DDX17\_HUMAN, DDX17\_HUMAN, DDX17\_HUMAN, DDX17\_HUMAN, DDX5\_HUMAN |
| Sequence | ``` APILIATDVASR ``` |
| Position | 469 |
| PTMs | none |

  

Protein Q92841-2

| Name | DDX17\_HUMAN | | |
| Description | Isoform 2 of Probable ATP dependent RNA helicase DDX17 OS Homo sapiens GN DDX17 | | |
| Sequence | ```   1 MRGGGFGDRD RDRDRGGFGA RGGGGLPPKK FGNPGERLRK KKWDLSELPK FEKNFYVEHP EVARLTPYEV DELRRKKEIT VRGGDVCPKP VFAFHHANFP 101 QYVMDVLMDQ HFTEPTPIQC QGFPLALSGR DMVGIAQTGS GKTLAYLLPA IVHINHQPYL ERGDGPICLV LAPTRELAQQ VQQVADDYGK CSRLKSTCIY 201 GGAPKGPQIR DLERGVEICI ATPGRLIDFL ESGKTNLRRC TYLVLDEADR MLDMGFEPQI RKIVDQIRPD RQTLMWSATW PKEVRQLAED FLRDYTQINV 301 GNLELSANHN ILQIVDVCME SEKDHKLIQL MEEIMAEKEN KTIIFVETKR RCDDLTRRMR RDGWPAMCIH GDKSQPERDW VLNEFRSGKA PILIATDVAS 401 RGLGLDVEDV KFVINYDYPN SSEDYVHRIG RTARSTNKGT AYTFFTPGNL KQARELIKVL EEANQAINPK LMQLVDHRGG GGGGGGRSRY RTTSSANNPN 501 LMYQDECDRR LRGVKDGGRR DSASYRDRSE TDRAGYANGS GYGSPNSAFG AQAGQYTYGQ GTYGAAAYGT SSYTAQEYGA GTYGASSTTS TGRSSQSSSQ 601 QFSGIGRSGQ QPQPLMSQQF AQPPGATNMI GYMGQTAYQY PPPPPPPPPS RK ``` | | |
| Evidence | Indistinguishable | | |
| Peptide list | 267\*, 575\*\* | | |
| Peptides | 267\* | Confidence | Yellow |
| Runs | 1, 2, 4 |
| Relation | Discriminating |
| Proteins | DDX17\_HUMAN, DDX17\_HUMAN, DDX17\_HUMAN |
| Sequence | ``` LMQLVDHRGGGGGGGGR ``` |
| Position | 471 |
| PTMs | Deamidation+Q(3) |
| 575\*\* | Confidence | Green |
| Runs | 1, 2, 3, 4 |
| Relation | NonDiscriminating |
| Proteins | DDX17\_HUMAN, DDX17\_HUMAN, DDX17\_HUMAN, DDX17\_HUMAN, DDX5\_HUMAN |
| Sequence | ``` APILIATDVASR ``` |
| Position | 390 |
| PTMs | none |

  

Protein P39687

| Name | AN32A\_HUMAN | | |
| Description | Acidic leucine rich nuclear phosphoprotein 32 family member A OS Homo sapiens GN ANP32A PE 1 SV 1 | | |
| Sequence | ```   1 MEMGRRIHLE LRNRTPSDVK ELVLDNSRSN EGKLEGLTDE FEELEFLSTI NVGLTSIANL PKLNKLKKLE LSDNRVSGGL EVLAEKCPNL THLNLSGNKI 101 KDLSTIEPLK KLENLKSLDL FNCEVTNLND YRENVFKLLP QLTYLDGYDR DDKEAPDSDA EGYVEGLDDE EEDEDEEEYD EDAQVVEDEE DEDEEEEGEE 201 EDVSGEEEED EEGYNDGEVD DEEDEEELGE EERGQKRKRE PEDEGEDDD ``` | | |
| Evidence | Indistinguishable | | |
| Peptide list | 884\*, 919\* | | |
| Peptides | 884\* | Confidence | Green |
| Runs | 1, 2, 4 |
| Relation | Discriminating |
| Proteins | AN32A\_HUMAN, AN32B\_HUMAN, AN32B\_HUMAN |
| Sequence | ``` LLPQLTYLDGYDR ``` |
| Position | 138 |
| PTMs | none |
| 919\* | Confidence | Green |
| Runs | 1, 2, 4 |
| Relation | Discriminating |
| Proteins | AN32A\_HUMAN, AN32B\_HUMAN, AN32B\_HUMAN |
| Sequence | ``` SLDLFNCEVTNLNDYR ``` |
| Position | 117 |
| PTMs | Carbamidomethyl+C(7) |

  

Protein Q92688-2

| Name | AN32B\_HUMAN | | |
| Description | Isoform 2 of Acidic leucine rich nuclear phosphoprotein 32 family member B OS Homo sapiens GN ANP32B | | |
| Sequence | ```   1 MDMKRRIHLE LRNRTPAAVR ELVLDNCKSN DGKIEGLTAE FVNLEFLSLI NVGLISVSNL PKLPKLKKLE LSENRIFGGL DMLAEKLPNL THLNLSGNKL 101 KDISTLEPLK KLECLKSLDL FNCEVTNLND YRESVFKLLP QLTYLDGYDR EDQEAPDSDA EVDGVDEEEE DEEGEDEEDE DDEDGEEEEF DEEDD ``` | | |
| Evidence | Indistinguishable | | |
| Peptide list | 884\*, 919\* | | |
| Peptides | 884\* | Confidence | Green |
| Runs | 1, 2, 4 |
| Relation | Discriminating |
| Proteins | AN32A\_HUMAN, AN32B\_HUMAN, AN32B\_HUMAN |
| Sequence | ``` LLPQLTYLDGYDR ``` |
| Position | 138 |
| PTMs | none |
| 919\* | Confidence | Green |
| Runs | 1, 2, 4 |
| Relation | Discriminating |
| Proteins | AN32A\_HUMAN, AN32B\_HUMAN, AN32B\_HUMAN |
| Sequence | ``` SLDLFNCEVTNLNDYR ``` |
| Position | 117 |
| PTMs | Carbamidomethyl+C(7) |

  

Protein Q92688

| Name | AN32B\_HUMAN | | |
| Description | Acidic leucine rich nuclear phosphoprotein 32 family member B OS Homo sapiens GN ANP32B PE 1 SV 1 | | |
| Sequence | ```   1 MDMKRRIHLE LRNRTPAAVR ELVLDNCKSN DGKIEGLTAE FVNLEFLSLI NVGLISVSNL PKLPKLKKLE LSENRIFGGL DMLAEKLPNL THLNLSGNKL 101 KDISTLEPLK KLECLKSLDL FNCEVTNLND YRESVFKLLP QLTYLDGYDR EDQEAPDSDA EVDGVDEEEE DEEGEDEEDE DDEDGEEEEF DEEDDEDEDV 201 EGDEDDDEVS EEEEEFGLDE EDEDEDEDEE EEEGGKGEKR KRETDDEGED D ``` | | |
| Evidence | Indistinguishable | | |
| Peptide list | 884\*, 919\* | | |
| Peptides | 884\* | Confidence | Green |
| Runs | 1, 2, 4 |
| Relation | Discriminating |
| Proteins | AN32A\_HUMAN, AN32B\_HUMAN, AN32B\_HUMAN |
| Sequence | ``` LLPQLTYLDGYDR ``` |
| Position | 138 |
| PTMs | none |
| 919\* | Confidence | Green |
| Runs | 1, 2, 4 |
| Relation | Discriminating |
| Proteins | AN32A\_HUMAN, AN32B\_HUMAN, AN32B\_HUMAN |
| Sequence | ``` SLDLFNCEVTNLNDYR ``` |
| Position | 117 |
| PTMs | Carbamidomethyl+C(7) |

  

Protein P22234

| Name | PUR6\_HUMAN | | |
| Description | Multifunctional protein ADE2 OS Homo sapiens GN PAICS PE 1 SV 3 | | |
| Sequence | ```   1 MATAEVLNIG KKLYEGKTKE VYELLDSPGK VLLQSKDQIT AGNAARKNHL EGKAAISNKI TSCIFQLLQE AGIKTAFTRK CGETAFIAPQ CEMIPIEWVC 101 RRIATGSFLK RNPGVKEGYK FYPPKVELFF KDDANNDPQW SEEQLIAAKF CFAGLLIGQT EVDIMSHATQ AIFEILEKSW LPQNCTLVDM KIEFGVDVTT 201 KEIVLADVID NDSWRLWPSG DRSQQKDKQS YRDLKEVTPE GLQMVKKNFE WVAERVELLL KSESQCRVVV LMGSTSDLGH CEKIKKACGN FGIPCELRVT 301 SAHKGPDETL RIKAEYEGDG IPTVFVAVAG RSNGLGPVMS GNTAYPVISC PPLTPDWGVQ DVWSSLRLPS GLGCSTVLSP EGSAQFAAQI FGLSNHLVWS 401 KLRASILNTW ISLKQADKKI RECNL ``` | | |
| Evidence | Indistinguishable | | |
| Peptide list | 23\*, 1416\* | | |
| Peptides | 23\* | Confidence | Green |
| Runs | 1, 2, 3, 4 |
| Relation | Discriminating |
| Proteins | PUR6\_HUMAN, PUR6\_HUMAN |
| Sequence | ``` DQITAGNAAR ``` |
| Position | 37 |
| PTMs | none |
| 1416\* | Confidence | Yellow |
| Runs | 2, 3, 4 |
| Relation | Discriminating |
| Proteins | PUR6\_HUMAN, PUR6\_HUMAN |
| Sequence | ``` EVYELLDSPGK ``` |
| Position | 20 |
| PTMs | none |

  

Protein P22234-2

| Name | PUR6\_HUMAN | | |
| Description | Isoform 2 of Multifunctional protein ADE2 OS Homo sapiens GN PAICS | | |
| Sequence | ```   1 MATAEVLNIG KKLYEGKTKE VYELLDSPGK VLLQSKDQIT AGNAARKNHL EGKAAISNKI TSCIFQLLQE AVTSYKSNRI KTAFTRKCGE TAFIAPQCEM 101 IPIEWVCRRI ATGSFLKRNP GVKEGYKFYP PKVELFFKDD ANNDPQWSEE QLIAAKFCFA GLLIGQTEVD IMSHATQAIF EILEKSWLPQ NCTLVDMKIE 201 FGVDVTTKEI VLADVIDNDS WRLWPSGDRS QQKDKQSYRD LKEVTPEGLQ MVKKNFEWVA ERVELLLKSE SQCRVVVLMG STSDLGHCEK IKKACGNFGI 301 PCELRVTSAH KGPDETLRIK AEYEGDGIPT VFVAVAGRSN GLGPVMSGNT AYPVISCPPL TPDWGVQDVW SSLRLPSGLG CSTVLSPEGS AQFAAQIFGL 401 SNHLVWSKLR ASILNTWISL KQADKKIREC NL ``` | | |
| Evidence | Indistinguishable | | |
| Peptide list | 23\*, 1416\* | | |
| Peptides | 23\* | Confidence | Green |
| Runs | 1, 2, 3, 4 |
| Relation | Discriminating |
| Proteins | PUR6\_HUMAN, PUR6\_HUMAN |
| Sequence | ``` DQITAGNAAR ``` |
| Position | 37 |
| PTMs | none |
| 1416\* | Confidence | Yellow |
| Runs | 2, 3, 4 |
| Relation | Discriminating |
| Proteins | PUR6\_HUMAN, PUR6\_HUMAN |
| Sequence | ``` EVYELLDSPGK ``` |
| Position | 20 |
| PTMs | none |

  

Protein P26599

| Name | PTBP1\_HUMAN | | |
| Description | Polypyrimidine tract binding protein 1 OS Homo sapiens GN PTBP1 PE 1 SV 1 | | |
| Sequence | ```   1 MDGIVPDIAV GTKRGSDELF STCVTNGPFI MSSNSASAAN GNDSKKFKGD SRSAGVPSRV IHIRKLPIDV TEGEVISLGL PFGKVTNLLM LKGKNQAFIE 101 MNTEEAANTM VNYYTSVTPV LRGQPIYIQF SNHKELKTDS SPNQARAQAA LQAVNSVQSG NLALAASAAA VDAGMAMAGQ SPVLRIIVEN LFYPVTLDVL 201 HQIFSKFGTV LKIITFTKNN QFQALLQYAD PVSAQHAKLS LDGQNIYNAC CTLRIDFSKL TSLNVKYNND KSRDYTRPDL PSGDSQPSLD QTMAAAFGLS 301 VPNVHGALAP LAIPSAAAAA AAAGRIAIPG LAGAGNSVLL VSNLNPERVT PQSLFILFGV YGDVQRVKIL FNKKENALVQ MADGNQAQLA MSHLNGHKLH 401 GKPIRITLSK HQNVQLPREG QEDQGLTKDY GNSPLHRFKK PGSKNFQNIF PPSATLHLSN IPPSVSEEDL KVLFSSNGGV VKGFKFFQKD RKMALIQMGS 501 VEEAVQALID LHNHDLGENH HLRVSFSKST I ``` | | |
| Evidence | Indistinguishable | | |
| Peptide list | 1035\*, 1054\*, 1579\* | | |
| Peptides | 1035\* | Confidence | Green |
| Runs | 1, 3, 4 |
| Relation | Discriminating |
| Proteins | PTBP1\_HUMAN, PTBP1\_HUMAN |
| Sequence | ``` IAIPGLAGAGNSVLLVSNLNPER ``` |
| Position | 326 |
| PTMs | none |
| 1054\* | Confidence | Yellow |
| Runs | 1, 3, 4 |
| Relation | Discriminating |
| Proteins | PTBP1\_HUMAN, PTBP1\_HUMAN |
| Sequence | ``` KLPIDVTEGEVISLGLPFGK ``` |
| Position | 65 |
| PTMs | none |
| 1579\* | Confidence | Green |
| Runs | 2, 3, 4 |
| Relation | Discriminating |
| Proteins | PTBP1\_HUMAN, PTBP1\_HUMAN |
| Sequence | ``` NNQFQALLQYADPVSAQHAK ``` |
| Position | 219 |
| PTMs | none |

  

Protein P26599-2

| Name | PTBP1\_HUMAN | | |
| Description | Isoform 2 of Polypyrimidine tract binding protein 1 OS Homo sapiens GN PTBP1 | | |
| Sequence | ```   1 MDGIVPDIAV GTKRGSDELF STCVTNGPFI MSSNSASAAN GNDSKKFKGD SRSAGVPSRV IHIRKLPIDV TEGEVISLGL PFGKVTNLLM LKGKNQAFIE 101 MNTEEAANTM VNYYTSVTPV LRGQPIYIQF SNHKELKTDS SPNQARAQAA LQAVNSVQSG NLALAASAAA VDAGMAMAGQ SPVLRIIVEN LFYPVTLDVL 201 HQIFSKFGTV LKIITFTKNN QFQALLQYAD PVSAQHAKLS LDGQNIYNAC CTLRIDFSKL TSLNVKYNND KSRDYTRPDL PSGDSQPSLD QTMAAAFASP 301 YAGAGFPPTF AIPQAAGLSV PNVHGALAPL AIPSAAAAAA AAGRIAIPGL AGAGNSVLLV SNLNPERVTP QSLFILFGVY GDVQRVKILF NKKENALVQM 401 ADGNQAQLAM SHLNGHKLHG KPIRITLSKH QNVQLPREGQ EDQGLTKDYG NSPLHRFKKP GSKNFQNIFP PSATLHLSNI PPSVSEEDLK VLFSSNGGVV 501 KGFKFFQKDR KMALIQMGSV EEAVQALIDL HNHDLGENHH LRVSFSKSTI ``` | | |
| Evidence | Indistinguishable | | |
| Peptide list | 1035\*, 1054\*, 1579\* | | |
| Peptides | 1035\* | Confidence | Green |
| Runs | 1, 3, 4 |
| Relation | Discriminating |
| Proteins | PTBP1\_HUMAN, PTBP1\_HUMAN |
| Sequence | ``` IAIPGLAGAGNSVLLVSNLNPER ``` |
| Position | 345 |
| PTMs | none |
[truncated: 330,487 more chars]
